# Azido-alkynylation of alkenes through radical-polar crossover†

**DOI:** 10.1039/d3sc03309k

**Published:** 2023-08-11

**Authors:** Julien Borrel, Jerome Waser

**Affiliations:** a Laboratory of Catalysis and Organic Synthesis, Institute of Chemical Sciences and Engineering, Ecole Polytechnique Fédérale de Lausanne EPFL SB ISIC LCSO, BCH 4306 1015 Lausanne Switzerland jerome.waser@epfl.ch

## Abstract

We report an azido-alkynylation of alkenes allowing a straightforward access to homopropargylic azides by combining hypervalent iodine reagents and alkynyl-trifluoroborate salts. The design of a photocatalytic redox-neutral radical polar crossover process was key to develop this transformation. A variety of homopropargylic azides possessing electron-rich and -poor aryls, heterocycles or ether substituents could be accessed in 34–84% yield. The products are synthetically useful building blocks that could be easily transformed into pyrroles or bioactive amines.

## Abstract 2

An azido-alkynylation of alkenes has been developed by combining hypervalent iodine reagents and alkynyl-trifluoroborate salts. This method allows the synthesis of homopropargylic azides, which could be transformed into pyrroles or bioactive amines.

## Graphical abstract

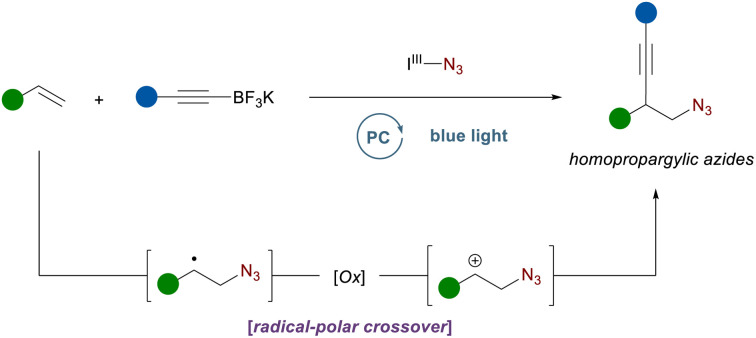

## Introduction

**Scheme 1:**
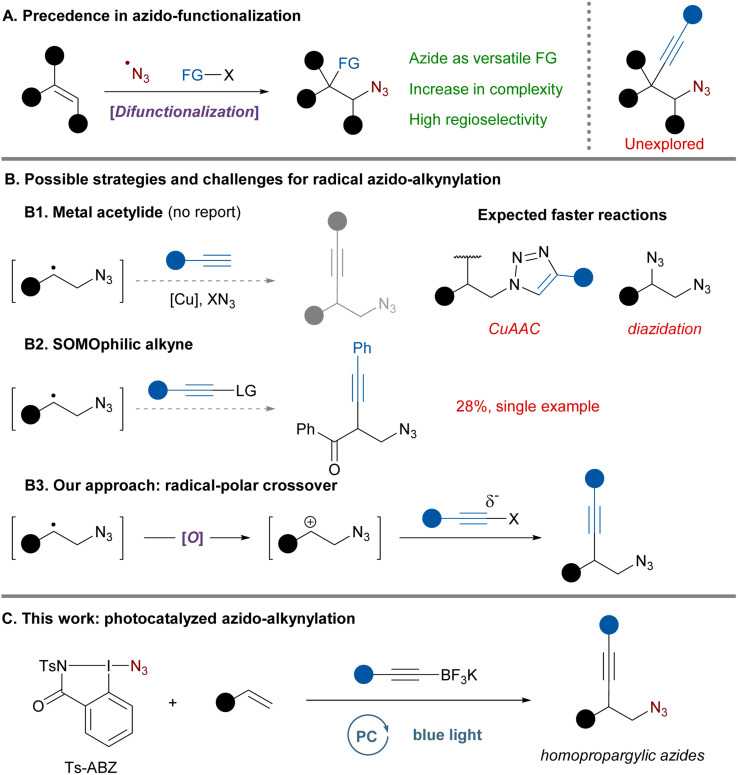
Azido-functionalization state of the art and challenges.

The azide moiety is widely recognized as a versatile functional group (FG) and it has found broad application in the pharmaceutical industry^1^ and in material science.^2^ It is both a form of protected amine and a powerful synthetic handle with a unique downstream chemistry.^3^ In recent years, the renaissance of radical chemistry triggered by photoredox catalysis led to the development of novel methods to introduce azides in organic molecules.^4^ In particular, the formation of azide radicals and their addition onto alkenes was demonstrated to be an efficient strategy for the synthesis of difunctionalized products (Scheme 1A).^4*a*,*f*^ After addition of the azide radical, different substituents including heteroatoms^5^ and aryls^6^ have been introduced on the intermediate carbon-centered radical. This represents a powerful strategy to quickly gain molecular complexity with the benefit of a highly regiospecific outcome resulting from the formation of the more stable carbon-centered radical.

Among all the difunctionalization methods developed, one of the potentially most useful – the azido-alkynylation – has surprisingly not yet been explored, except for the single example of the azido-alkynylation of phenyl-vinyl ketone in 28% yield reported as part of a mechanistic study (Scheme 1B).^7^ Alkynes are highly useful handles for further derivatization *via* cycloaddition or other triple bond functionalization methods.^8^ In this specific case, the resulting homopropargylic azides are interesting synthetic intermediates known to undergo cyclization to form pyrroles.^9^ Moreover, upon reduction they would afford homopropargylic amines which can be found in bioactive molecules.^10^ Homopropargylic azides are currently accessed from epoxides using a sequence of ring opening with lithium acetylide, mesylation of the resulting alcohol and displacement by azide anions.^9*d*,*f*^ Consequently, more direct synthetic approaches to access such motifs would be of general interest and high synthetic value.

Developing a radical azido-alkynylation of alkenes would initially involve azide radical addition to the double bond. From there multiple approaches could be envisaged to transfer the alkyne to the intermediate carbon radical (Scheme 1B).^11^ Classical strategies based on the recombination with a metal acetylide followed by reductive elimination^12^ would not be compatible in the case of azidation (Scheme 1B1). Copper acetylides are the most classical intermediates used in this chemistry but the presence of azides, free alkynes and copper would lead to cycloaddition reactions.^1*b*^ Additionally, copper catalysts and different azide sources are known to effectively promote the diazidation of alkenes, often proceeding *via* radical intermediates.^13^ It is therefore not surprising that no azido-alkynylation following this mechanism has been reported so far. A second approach solely based on open-shell species would use SOMOphilic alkynes in an addition–elimination process to provide the desired product (Scheme 1B2).^12*a*,14^ Nevertheless, this system would have major limitations as commonly used alkyne-transfer reagents (ethynylbenziodoxolone and alkynyl sulfones) often require aryl substituents to perform efficiently.^15^ Moreover, while frequently used to trap alkyl radicals, only a few examples exist for more stabilized benzylic radicals and are often associated with a lower yield,^16^ a narrow scope^17^ or a high excess of radical.^18^ All those factors could explain why there is only one report of such an approach for azido-alkynylation proceeding in 28% yield on phenyl vinyl ketone as a very activated substrate.^7^

In order to overcome this gap in existing synthetic methodologies, we thought of an alternative pathway involving the merger of radical and polar chemistry.^19^ Transformations involving radical-polar crossover (RPC) mechanisms have recently received increased attention as they enable the combination of orthogonal reagents only active in either radical or polar regime. Additionally, redox-neutral processes can be developed by careful design of the catalytic cycle. Upon oxidation of the intermediate C-centered radical, the carbocation formed could be trapped by a nucleophilic alkyne affording the desired product (Scheme 1B3). In fact, Xu^20^ and Molander^21^ elegantly demonstrated that alkene radical cations and benzylic carbocations could be trapped by nucleophilic trifluoroborate salts. In addition, rare cases of RPC reactions involving azide radicals have been reported, but the nucleophiles were limited to methanol,^13*b*^ carboxylic acids^22^ or alkyl groups during a semipinacol rearrangement.^23^

Although the envisaged 3-component synthesis of homopropargylic azides based on a RPC approach looks promising, there are still significant challenges to overcome: a non-nucleophilic azide radical precursor needs to be selected to limit diazidation and a nucleophilic alkyne efficient enough for carbocation trapping before decomposition is required. Herein, we report the photocatalyzed azido-alkynylation of styrenes using the combination of an azidoiodane reagent and alkynyl-trifluoroborate salts (Scheme 1C). Using this radical-polar crossover strategy a large variety of homopropargylic azides could be accessed in a single step.

## Results and discussion

Following optimization studies, the azido-alkynylation of styrene 1a was achieved using Ts-ABZ as azide radical source upon single electron reduction. This hypervalent iodine reagent is a safer version of the more commonly used azidobenziodoxolone, also known as Zhdankin reagent,^24^ which showed an explosion hazard.^23^ Potassium alkynyl-trifluoroborates were selected as nucleophilic alkynes as they had been previously employed for the trapping of similar carbocations.^20,21^ The reaction was performed under photoredox conditions with BF_3_·Et_2_O as additive to afford 3a in 74% yield after 1.5 hours at −20 °C (Table 1, entry 1).

**Table 1:** Reaction optimization^a^

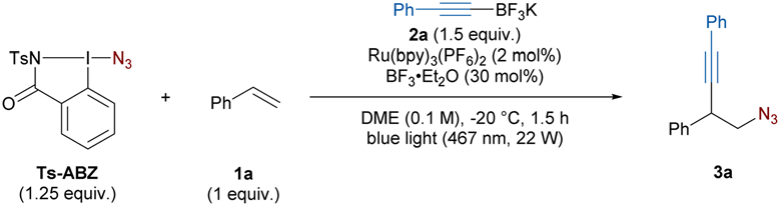		
Entry	Variation from standard conditions	Yield^b^ (%)
1	None	74
2	Ts-ABZ as limiting reagent	69
3	2a as limiting reagent	44
4	*C* = 0.05 M	74
5	*C* = 0.2 M	60
6	Room temperature	50
7	No BF_3_·Et_2_O	49
8	No light or photocatalyst	<5

a Reactions were carried out on 0.1 mmol scale. Light irradiation was carried out using a single Kessil lamp.

b NMR yield determined using CH_2_Br_2_ as internal standard.

Both styrene (1a) and Ts-ABZ can be used as the limiting reagent, but a lower yield was observed when 1.0 equivalent of 2a was used (entries 1–3). Lower and higher concentrations had little to no impact on the reaction outcome (entries 4 and 5). Raising the temperature to 21 °C led to a decrease in yield (entry 6). A similar result was observed when BF_3_·Et_2_O was not added (entry 7). Finally, control experiments in the absence of light or photocatalyst afforded only traces of the desired product (entry 8). Full optimization tables, including screening of photocatalyst, solvents, equivalents, light sources and additives can be found in the ESI (Tables S1–S8).†

**Scheme 2:**
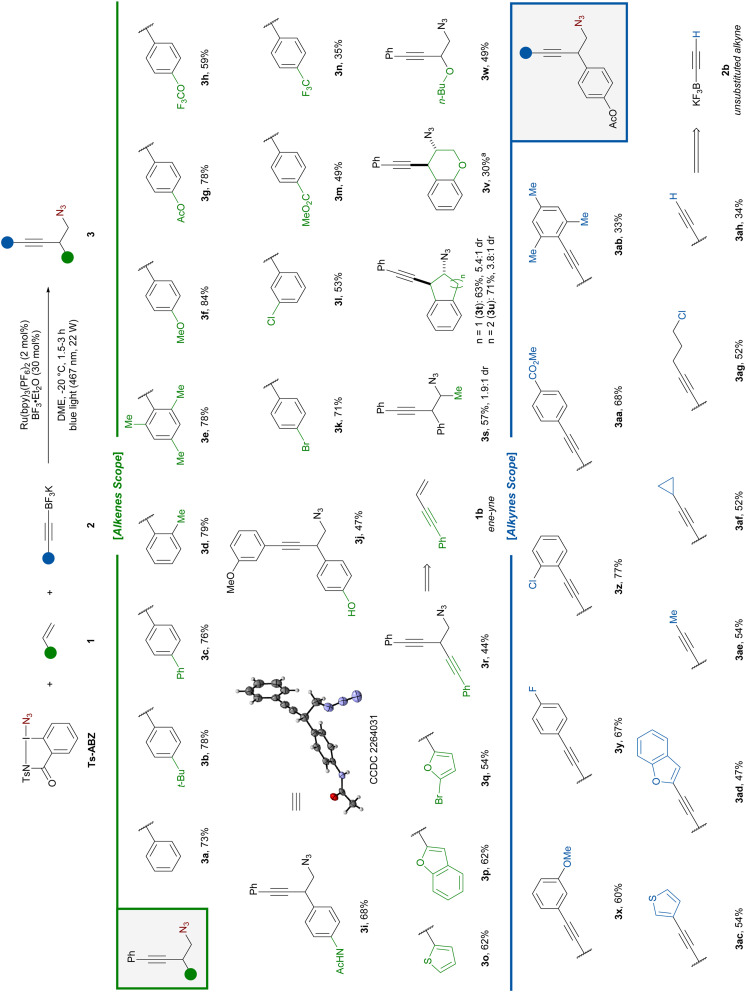
Scope of the azido-alkynylation. Reaction conditions: 1 (1 equiv.), 2 (1.5 equiv.), Ts-ABZ (1.25 equiv.), Ru(bpy)_3_(PF_6_)_2_ (2 mol%), BF_3_·Et_2_O (30 mol%), DME (0.1 M), −20 °C, blue light (467 nm, 22 W), 1.5 to 3 h. The major diastereoisomer is drawn, the dr was determined on the crude reaction mixture by ^1^H NMR. ^*a*^Only diastereoisomer observed.

With optimized conditions in hand, the scope of styrenes was investigated (Scheme 2). In all reactions, full conversion of the alkene was achieved. As no other small molecule side products were observed, we assign the different isolated yields observed to different levels of oligomerization/polymerization of the alkenes. The model substrate 3a was obtained in 73% yield on a 0.3 mmol scale. Styrenes bearing a *tert*-butyl or a phenyl group in *para* position gave products 3b and 3c in 78% and 76% yield, respectively. Steric hindrance on the aryl ring was well tolerated as 3d and 3e possessing one or two *ortho* substituents could be obtained in >78% yield. Oxygen-substituted aryls with substituents such as methoxy and acetoxy could also be used (3f–g). A slight decreased in yield was observed in the presence of the medicinal chemistry relevant trifluoromethoxy substituent (3h).^25^ Pleasingly, the presence of nucleophilic functional groups, which could have compete with 2a for the trapping of the carbocation, did not hinder the reaction: products 3i or 3j bearing an acetamide and a free phenol could be accessed in 68% and 47% yield, respectively. Additionally, an X-ray structure of 3i was obtained. Halogen-substituted arenes afforded the corresponding azido-alkynylated products 3k–l in 71% and 53% yield, respectively.

Electron-withdrawing group (EWG), which could be expected to destabilize the carbocation intermediate, were still tolerated in the reaction. Substrates bearing a *para* ester and CF_3_ group afforded the corresponding products 3m and 3n in 49% and 35% yield. Moreover, homopropargylic azides 3o–p containing electron-rich heterocycles such as thiophene and benzofuran were obtained in 62% yield. Carrying out the reaction on a sensitive bromo-substituted vinyl-furan, which quickly polymerizes after synthesis, successfully gave product 3q in 54% yield.

Eneyne 1b could be exclusively 1,2-functionalized to give diyne 3r in 44% yield. The reaction tolerated β-substitution on the styrene: product 3s bearing a methyl substituent was obtained in 57% yield as a 1.9 : 1 mixture of diastereoisomers. Using the less flexible cyclic indene, the diastereoselectivity of the reaction could be increased to 5.4 : 1 in favor of the *trans*3t isomer. Increasing the ring size slightly improved the yield, but lowered the dr (3u). When chromene was used, the azido-alkynylated product 3v was formed in 30% yield. No other diastereoisomer was observed. Gratifyingly, vinyl butyl ether could be azido-alkynylated to afford 3w in 49% yield. Unfortunately, alkenes bearing aliphatic substituents only could not be used.

Next, the scope of nucleophilic alkynes was studied. Aryl-alkynes bearing either EDG (OMe) or EWG (F, Cl, CO_2_Me) at different positions gave the corresponding products 3x–aa in 60–77% yield. In this case, the steric hindrance of the nucleophile seems to be an important factor as the use of mesityl-alkyne led to 3ab in only 33% yield. We were pleased to see that heteroaryl such at 3-thiophene or 2-benzofuran afforded the desired product 3ac and 3ad in 54% and 47% yield, respectively. Alkyl-substituted alkynes bearing methyl, cyclopropyl or a propyl chain possessing a chloride were well tolerated affording the corresponding products 3ae–ag in 52–54% yields. Finally, using unsubstituted alkynyl-BF_3_K 2b, terminal alkyne 3ah was obtained in 34% yield allowing for potential further diversification *via* cross-coupling.

**Scheme 3:**
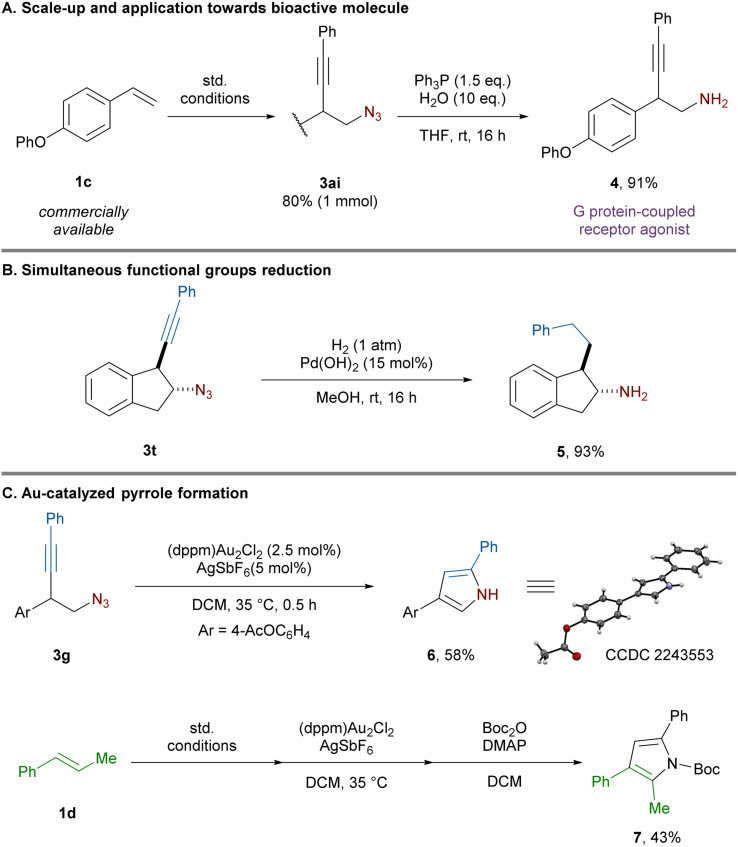
Product modifications.

To demonstrate the synthetic utility of the homopropargylic azides, various post functionalizations were carried out (Scheme 3). First, the azido-alkynylation was performed on 1 mmol scale using styrene 1c bearing a *para* phenoxy group affording the desired product 3ai in 80% yield under the same reaction conditions. Further reduction of the azide afforded primary amine 4 in high yield. The corresponding HCl salt is a known agonist for G protein-coupled receptors currently synthesized through a 4-step sequence in 19% overall yield.^10*d*^

Upon reduction of both the alkyne and azide, 5 was obtained in 93% yield affording a formal 2-step amino-alkylation. Pyrroles play a crucial role in the pharmaceutical industry as they are one of the most frequently encountered heterocycles in bioactive compounds.^26^ Applying conditions developed by Toste using gold catalysis,^9*a*^ homopropargylic azide 3g underwent 5-*endo-dig* cyclization to afford 6. Non-cyclic β-substituted alkenes afforded poor diastereoselectivity in the azido-alkynylation reaction (Scheme 2, 3s). This issue is inconsequential for pyrrole synthesis, as all stereoisomers are converted in a single product. For example, styrene 1d was effectively converted to trisubstituted pyrrole 7 in 43% yield over a 3 step-sequence of azido-alkynylation, cyclization and protection.

**Scheme 4:**
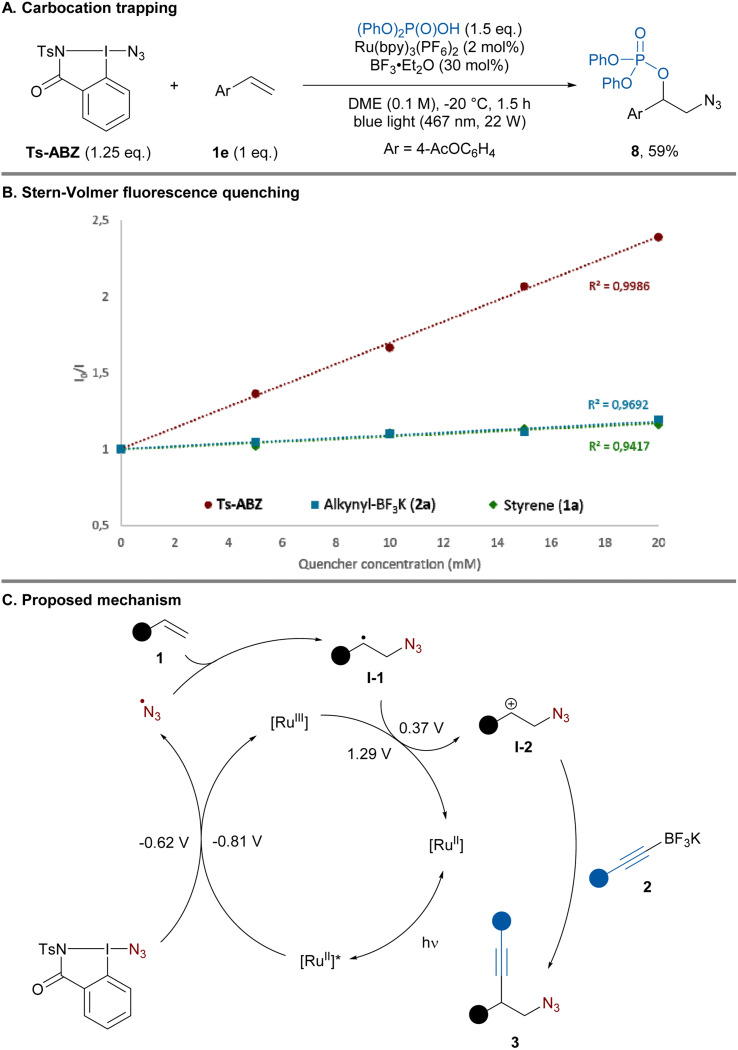
Mechanistic experiments and proposed mechanism.

To gain insight into the reaction mechanism, control experiments were performed. In the absence of light and photocatalyst only traces of the product could be obtained (Table 1, entry 8). Replacing the alkyne nucleophile by diphenyl phosphate led to the formation of azido-phosphonylated product 8 in 59% yield, presumably resulting from trapping of the carbocation intermediate (Scheme 4A).

Next, Stern–Volmer quenching experiments were performed. Ts-ABZ proved to be the most efficient quencher of the excited state photocatalyst compared to alkyne 2a and styrene 1a (Scheme 4B). Based on these experiments and literature precedents, a plausible mechanism could be proposed (Scheme 4C).^13*b*,20,21^ Under blue light irradiation, excited state Ru(bpy)_3_^2+*^ (*E*_1/2_ [Ru^III^/Ru^II*^] = −0.86 V *vs.* SCE)^27^ is capable of reducing Ts-ABZ (*E*_1/2_^red^ = −0.62 V *vs.* SCE)^28^ generating the azide radical. Addition of the latter to alkene 1 would lead to carbon-centered radical I-1 (*E*_1/2_^ox^ = 0.37 V *vs.* SCE)^29^ which can be oxidized by the previously formed Ru(bpy)^3+^ (*E*_1/2_ [Ru^III^/Ru^II^] = + 1.29 V *vs.* SCE)^27^ regenerating the ground state photocatalyst. Finally, the resulting carbocation I-2 would be trapped by the nucleophilic alkynyl-BF_3_K 2 affording homopropargylic azide 3. Establishing the mechanism of this addition step would need further studies, but a concerted C–C bond formation and C–B bond cleavage could be operative, in analogy to what has been proposed for alkenyl boronate salts.^20^ The exact role of BF_3_·Et_2_O is still unclear, it is known to abstract fluoride from alkynyl-BF_3_K to form alkynyl-BF_2_.^30^ Control experiment involving preformation of alkynyl-BF_2_ and its subsequent addition instead of BF_3_·Et_2_O led to comparable yield hinting at the potential formation of alkynyl-BF_2_ under the standard conditions (see the ESI† Section 9.3).

## Conclusions

In summary, a photocatalyzed azido-alkynylation of alkenes using Ts-ABZ as azide radical source and nucleophilic alkynyl-trifluoroborate salts was developed. The reaction proceeds in high yield for electron-rich and electron-poor styrenes. Various aryl-, alkyl- or unsubstituted alkynes were successfully transferred to generate azido-alkynylated scaffolds. Moreover, heterocycles were compatible on both the alkene and alkyne fragment. The homopropargylic azides could be further derivatized, giving access to valuable pyrroles and the efficient 2-step synthesis of a G protein-coupled receptor agonist. The reaction is proposed to proceed through an overall redox-neutral process *via* a radical-polar crossover mechanism.^31,32^

## Data availability

ESI† available: Experimental procedures, characterization data and scan of NMR spectra. Crystallographic data is available at CCDC (see note 31). Raw data for compound characterization, including NMR, IR and MS is available at zenodo.org: https://doi.org/10.5281/zenodo.8239023.

## Author contributions

J. B. conceived the project, optimized the reaction, performed the investigation on the scope of the reaction, the modification of the products and prepared the experimental parts and first draft of the manuscript. J. W. supervised the project, edited the manuscript and proofread the experimental part.

## Conflicts of interest

There are no conflicts to declare.

## Supplementary Material

SC-014-D3SC03309K-s001

SC-014-D3SC03309K-s002
