# Supplementary material for: Azido-alkynylation of alkenes through radical-polar crossover
Source: Chem Sci. 2023 Aug 11;14(35):9452–60. doi: 10.1039/d3sc03309k (PMC10498506; doi:10.1039/d3sc03309k)

Electronic Supplementary Material (ESI) for Chemical Science.
This journal is © The Royal Society of Chemistry 2023

Supporting Information for

Azido-Alkynylation of Alkenes Through Radical-Polar Crossover

Julien Borrel and Jerome Waser*

Laboratory of Catalysis and Organic Synthesis, Institute of Chemical Sciences and Engineering, Ecole Polytechnique Fédérale de Lausanne, EPFL SB ISIC LCSO, BCH 1402, 1015 Lausanne, Switzerland.

jerome.waser@epfl.ch

(108 pages)

Table of Contents

1. General Methods 3

2. Picture of the Photochemistry Set-Up 4

3. Reaction Optimization 5

4. Synthesis of Hypervalent Iodine Reagents 9

5. Synthesis of Alkenes 11

6. Synthesis of Potassium Trifluoroborate Salts 16

7. Azido-Alkynylation 23

7.1 Scope of Alkenes 23

7.1 Scope of Alkynes 35

8. Product Modifications 43

8.1 Scale-up 43

8.2 Azide reduction 43

8.3 Hydrogenation 44

8.4 Pyrrole formation 44

8.5 Telescoped protected pyrrole formation 45

9. Mechanistic Studies 46

9.1 Carbocation trapping 46

9.2 Stern-Volmer fluorescence quenching 47

9.3 Investigation of the $BF_3{\cdot}Et_2O$ effect 49

9.4 Cyclic voltammetry of Ts-ABZ 51

10. Crystal Structures 53

10.1 3i 53

10.2 6 54

11. Spectra of New Compounds 55

1. General Methods

All reactions were carried out under air unless stated otherwise. Reactions requiring heating were carried out using DrySyn heating block. For flash chromatography, distilled technical grade solvents were used. THF, toluene, Et_2O and CH_2Cl_2 were dried by passage over activated alumina under nitrogen atmosphere (H_2O content <10 ppm, Karl-Fischer titration). Solvents were degassed by bubbling with a balloon of argon. All chemicals were purchased from Acros, Aldrich, Combi-blocks, Fluka, Fluorochem, Merck, TCI or VWR and used as such unless stated otherwise.

Chromatographic purification was performed as flash chromatography using Silicycle silica 40-63 µm (230-400 mesh), using the solvents indicated as eluent with 0.1-0.5 bar pressure or using Biotage Isolera Spektra One with pre-packaged silica cartridges purchased from Buchi, models: Sepacore or GraceResolve (4 g, 12 g, 25 g, 40 g, 80 g, 120 g). When indicated purification were performed on activated neutral aluminium oxide (Brockmann activity I). TLC was performed on Merck silica gel 60 F_{254} TLC glass plates and visualized with UV light and potassium permanganate or *p*-anisaldehyde stain.

^{1}H-NMR spectra were recorded on a Bruker DPX-400 400 MHz spectrometer in chloroform-d, DMSO-d_6 or acetone-d_6. All signals are reported in ppm using the residual solvent signal as internal reference (chloroform-d: 7.26 ppm, DMSO-d_6: 2.50 ppm, acetone-d_6: 2.06 ppm). The data is being reported as (s = singlet, d = doublet, t = triplet, q = quadruplet, qi = quintet, m = multiplet or unresolved, bs = broad signal, coupling constant(s) in Hz, integration, assignment). ^{13}C-NMR spectra were recorded with {^{1}H} decoupling on a Bruker DPX-400 101 MHz spectrometer in chloroform-d, DMSO-d_6 or acetone-d_6. All signals are reported in ppm using the residual solvent signal as internal reference (chloroform-d: 77.2 ppm, DMSO-d_6: 39.5 ppm, acetone-d_6: 206.3 and 29.8 ppm). ^{19}F-NMR spectra were recorded with {^{1}H} decoupling on a Bruker DPX-400 376 MHz spectrometer in chloroform-d, DMSO-d_6 or acetone-d_6. ^{11}B-NMR spectra were recorded on a Bruker DPX-400 128 MHz spectrometer in DMSO-d_6 or acetone-d_6.

High resolution mass spectrometric measurements were performed by the mass spectrometry service of ISIC at the EPFL. Electrospray-ionisation HRMS data were acquired on a Q-Tof Ultima mass spectrometer (Waters) or a Q-Tof 6530 Accurate mass spectrometer (Agilent) operated in the positive ionization mode and fitted with a standard Z-spray ion source equipped with the Lock-Spray interface. Data from the Lock-Spray were used to calculate a correction factor for the mass scale and provide accurate mass information of the analyte. Data were processed using the MassLynx 4.1 software. Atmospheric pressure photo-ionisation (APPI) HRMS measurements were done on a LTQOrbitrap Elite instrument (Thermofisher) operated in the positive ionization mode.

Reactions under “blue LEDs irradiation” (440 nm, 40 W) were performed in test tubes (14 mL, soda-lime glass, wall thickness = 0.8 mm) which were placed at the center of a crystallization flask. On this flask were attached the blue LEDs (RUBAN LED 5MÈTRES - 60LED/M -3528 BLEU - IP65 with Transformateur pour Ruban LED 24W/2A/12V, bought directly on RubanLED.com). The distance between the LEDs and the test tubes was approximatively 3

cm. Long irradiation resulted in temperature increasing up to 35 °C during overnight reactions. Reactions using “Kessil lamp” of 467 nm or 440 nm used the corresponding models PR160L-467 or PRL160L-440.

2. Picture of the Photochemistry Set-Up

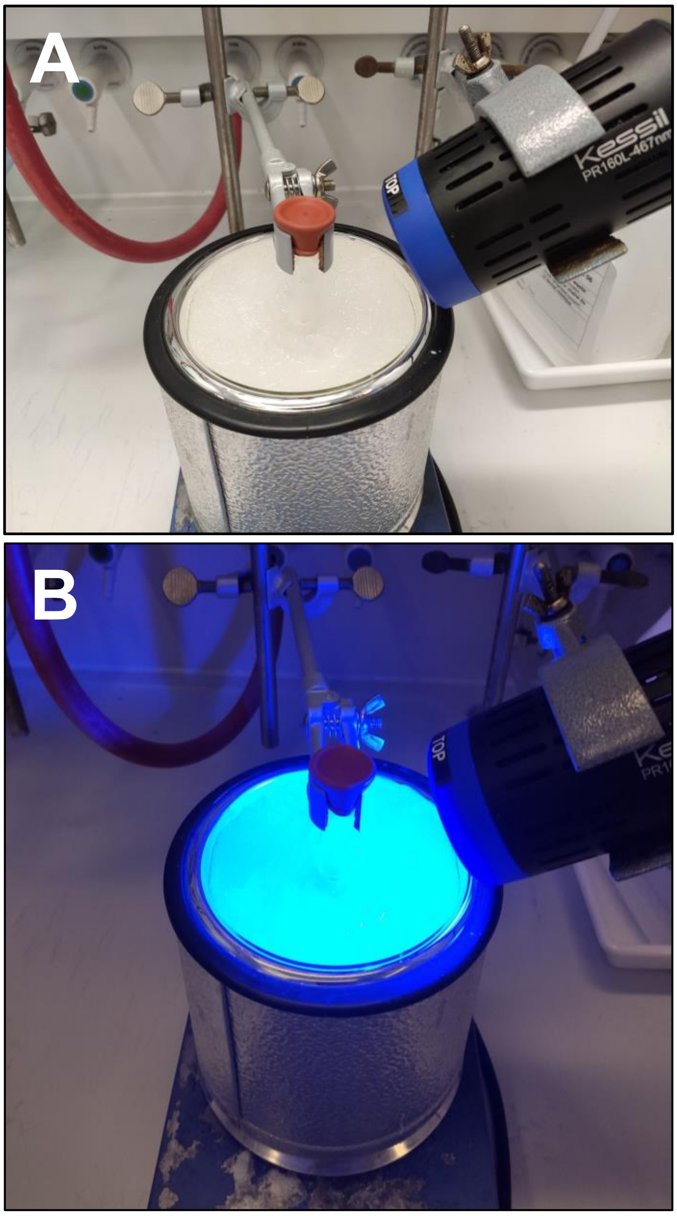

Figure S1: A) Picture of the set-up before turning on the Kessil lamp. B) Picture of the set-up during the reaction.

The Kessil lamp was placed diagonally at a distance of ~4 cm from the top of the reaction vessel (test tube or round-bottom flask). The latter was immersed in a bath of ice and salt (-20 °C) contained in a Dewar. For the optimized conditions the lamp intensity was set at 50% (22 W).

3. Reaction Optimization

Optimization protocol (0.1 mmol scale):

An oven-dried test tube charged with the solid reagents was evacuated and backfilled with N_2 (3x). Dry degassed solvent and the liquid reagents were added and the mixture was cooled to the desired temperature (if relevant). Then, additive (if relevant) was added and the reaction was stirred under light irradiation. The reaction mixture was filtered through a short plug of silica and eluted with DCM then concentrated *in vacuo*. ^{1}H NMR yield was determined by dissolving crude **3a** in $CDCl_3$ and adding CH_2Br_2 (3.5 µL, 0.049 mmol, 0.49 equiv.) as internal standard. The signal at 4.13 ppm was used to determine the yield.

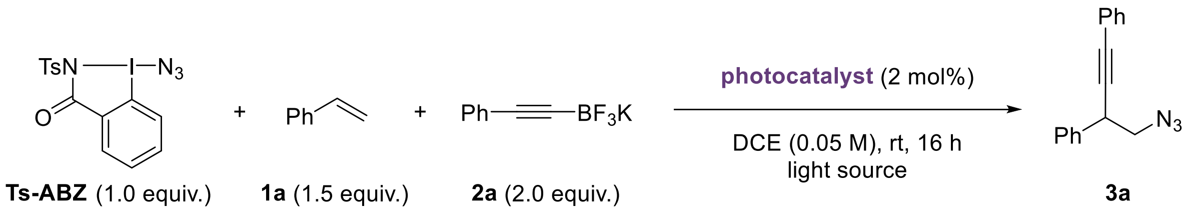

entry	photocatalyst	light source	NMR yield (%)
1	$Cu(dap)_2Cl$	blue LEDs	17
2	$Cu(dap)_2Cl$	green LEDs	21
3	$Ir(ppy)_3$	blue LEDs	n.o
4	$[Ir(dtbbpy)(ppy)_2]PF_6$	blue LEDs	<5%
5	$Ru(bpy)_3Cl_2•6H_2O$	blue LEDs	17
6	$Ru(bpz)_3(PF6)_2$	blue LEDs	n.o
7	4DPAIPN	blue LEDs	<5%
8	4ClDPAIPN	blue LEDs	10
9	4tBuCzIPN	blue LEDs	n.o
10	DPZ	blue LEDs	<5%
11	Rose Bengal	green LEDs	n.o

Table S1: Photocatalyst screening. n.o = not observed. [a]$Ru(bpy)_3Cl_2•6H_2O$ was selected as it afforded less diazidation.

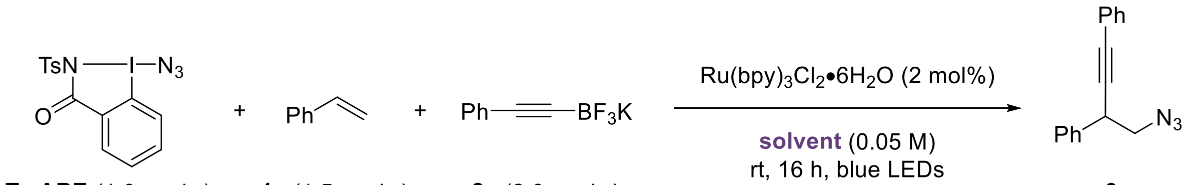

Ts-ABZ (1.0 equiv.) **1a** (1.5 equiv.) **2a** (2.0 equiv.) **3a**

entry	solvent	NMR yield (%)
1	DCE	17
2	$CHCl_3$	16
3	CH_3CN	9
4	DMF	n.o
5	DMSO	n.o
6	acetone	<5%
7	EtOAc	15
8	THF	8
9	dioxane	14
10	DME	36
11	DME/HFIP (9:1)	39
12	DME/TFE (9:1)	31

Table S2: Solvent screening. n.o = not observed.

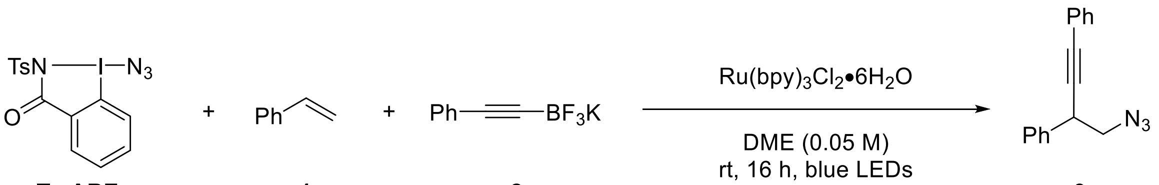

Ts-ABZ **1a** **2a** **3a**

entry	**Ts-ABZ** equiv.	**1a** equiv.	**2a** equiv.	PC (mol%)	NMR yield (%)
1	1	1.5	2	2	36
2	1.25	1	2	2	42
3	1.5	1	2	2	41
4	1.25	1	1.25	2	39
5	1.25	1	3	2	44
6	1.5	1.5	1	2	43
7	1.25	1	2	1	40
8	1.25	1	2	5	39

Table S3: Equivalent screening. PC = photocatalyst

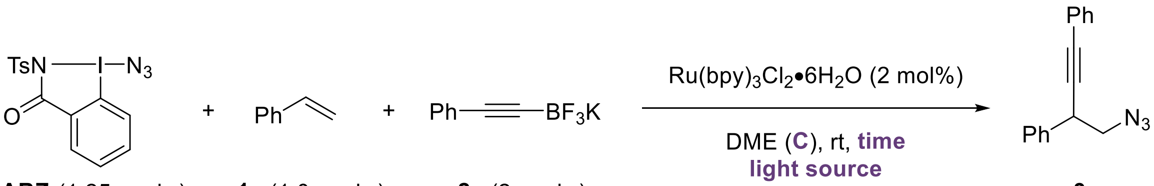

Ts-ABZ (1.25 equiv.) **1a** (1.0 equiv.) **2a** (2 equiv.) **3a**

entry	reaction time (h)	light source	Concentration (M)	NMR yield (%)
1	16	blue LEDs (40 W)	0.05	42
2	2	blue LEDs (40 W)	0.05	43
3	1	blue LEDs (40 W)	0.05	39
4	0.16	blue LEDs (40 W)	0.05	21
5	1.5	kessil 467 nm (44 W)	0.05	42
6	1.5	kessil 467 nm (22 W)	0.05	42
7	1.5	kessil 440 nm (45 W)	0.05	41
8	4	CFL (8 W)	0.05	38
9	1.5	green LEDs (40 W)	0.05	35
10	1.5	blue LEDs (40 W)	0.05	42
11	1.5	blue LEDs (40 W)	0.025	36
12	1.5	blue LEDs (40 W)	0.1	41
13	1.5	blue LEDs (40 W)	0.2	41

Table S4: Reaction time, light source and concentration screening. Blue/green LEDs refers to LEDs strip attached on a crystallization flask.

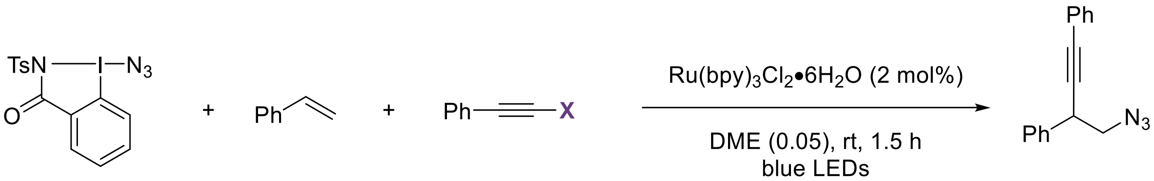

Ts-ABZ (1.25 equiv.) **1a** (1.0 equiv.) **2** (2.0 equiv.) **3a**

entry	X	NMR yield (%)
1	BF_3K	42
2	BF_3[TBA]	14
3	TMS	n.o

Table S5: Alkyne source screening. n.o = not observed.

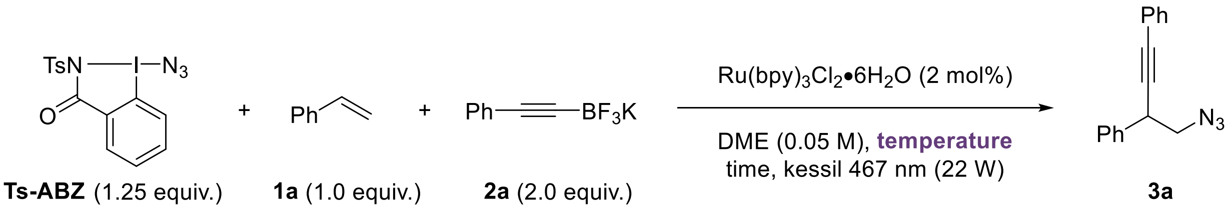

Ts-ABZ (1.25 equiv.) **1a** (1.0 equiv.) **2a** (2.0 equiv.) **3a**

entry	T (°C)	method of cooling	time (h)	NMR yield (%)
1	rt	none	1.5	42
2	0	immersion cooler	1.5	50
3	-20	immersion cooler	1.5	52
4	-20	ice and salt bath	1.5	53[a]
5	-46	immersion cooler	3	46

Table S6: Temperature screening. An immersion cooler was immersed in EtOAc. [a]Although there is little difference in yield between 0 and -20 °C we observed a better mass balance in the latter.

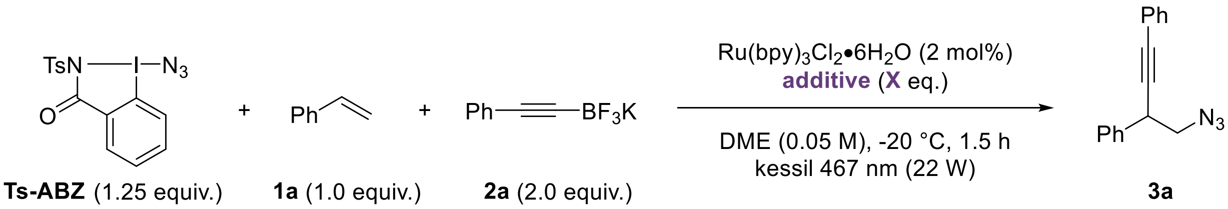

Ts-ABZ (1.25 equiv.) **1a** (1.0 equiv.) **2a** (2.0 equiv.) **3a**

entry	additive	equivalent	NMR yield (%)
1	none		53
2	MS 4Å	50 mg / 0.1 mmol	51
3	TMSCl	1 equiv.	40
4	TFAA	1 equiv.	54
5	$(TMS)_2O$	1 equiv.	48
6	$B(OTFE)_3$	1 equiv.	53
7	BF_3•Et_2O	1 equiv.	75
8	BF_3•Et_2O	2 equiv.	37
9	BF_3•Et_2O	0.5 equiv.	73
10	BF_3•Et_2O	0.3 equiv.	72

Table S7: Additive screening.

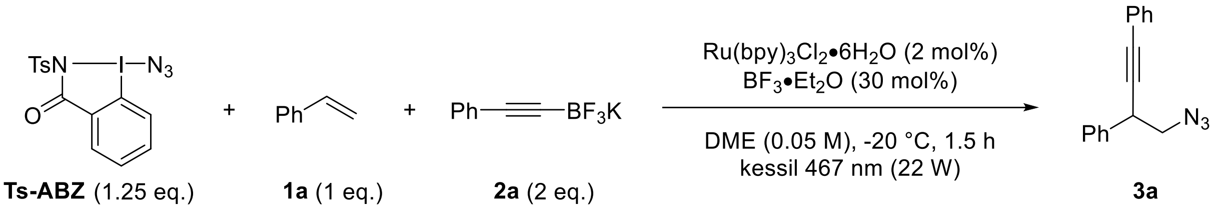

Ts-ABZ (1.25 eq.) **1a** (1 eq.) **2a** (2 eq.) **3a**

entry	change from standard condition	NMR yield (%)
1	none	72
2	C = 0.1 M	75
3	C = 0.1 M, **2a** (1.5 equiv.)	74
4	C = 0.1 M, **2a** (1.25 equiv.)	71
5	C = 0.1 M, **2a** (1.5 equiv.), no degassing	65
6	C = 0.1 M, **2a** (1.5 equiv.), $Ru(bpy)_3(PF_6)_2$ (2 mol%)	74
7	C = 0.1 M, **2a** (1.5 equiv.), $Ru(bpy)_3(PF_6)_2$ (2 mol%), DME/CH_3CN (9:1)	66
8	C = 0.1 M, **2a** (1.5 equiv.), $Ru(bpy)_3(PF_6)_2$ (2 mol%), *p*-ABSA instead of **Ts-ABZ**	n.o

Table S8: Fine-tuning of the reaction condition. n.o = not observed.

4. Synthesis of Hypervalent Iodine Reagents

3-Oxo-2-tosyl-2,3-dihydro-1*H*-1λ^3-benzo[*d*][1,2]iodazol-1-yl acetate (11):

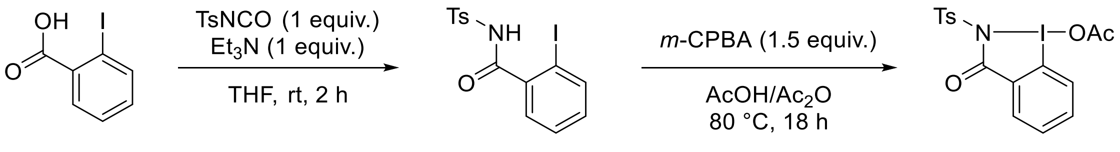

Following a reported procedure,[1] an oven dried round-bottom flask charged with 2-iodobenzoic acid (**9**) (10 g, 40 mmol, 1.0 equiv.) was evacuated and backfilled with N_2 (3x) then dry THF (115 mL) and *p*-toluenesulfonyl isocyanate (6.2 mL, 40 mmol, 1.0 equiv.) were added. Finally, triethylamine (5.6 mL, 40 mmol, 1.0 equiv.) was added dropwise under N_2, a slightly exothermic reaction began in addition to gas release. The reaction was stirred at rt for 2 h. The mixture was diluted with EtOAc (150 mL) and washed with 1 M aq. HCl (2 x 100 mL) and brine (50 mL). The organic layer was dried over $MgSO_4$, filtered and concentrated *in vacuo*. The crude product was used in the next step without further purification.

Following a reported procedure,[2] to a round-bottom flask charged with a solution of crude 2-iodo-*N*-tosylbenzamide (**10**) in a mixture of AcOH (70 mL) and Ac_2O (70 mL) was added *m*-CPBA (13.5 g, 60.5 mmol, 1.50 equiv., 77%). The reaction was stirred at 80 °C for 18 h covered from light. The mixture was left to cool to rt then pentane (100 mL) was added. The precipitate was filtered and washed with pentane (2 x 30 mL) and Et_2O (2 x 30 mL) and dried on the frit to afford 3-oxo-2-tosyl-2,3-dihydro-1*H*-1λ^3-benzo[*d*][1,2]iodazol-1-yl acetate (**11**) (7.74 g, 16.8 mmol, 42%) as an off white solid.

^{1}H NMR (400 MHz, $CDCl_3$) δ 8.12 (dd, *J* = 7.7, 1.6 Hz, 1H, Ar*H*), 8.04 (d, *J* = 8.3 Hz, 2H, Ar*H*), 7.99 (d, *J* = 8.4 Hz, 1H, Ar*H*), 7.87 – 7.81 (m, 1H, Ar*H*), 7.67 – 7.62 (m, 1H, Ar*H*), 7.33 (d, *J* = 8.1 Hz, 2H, Ar*H*), 2.42 (s, 3H, CH_3), 2.25 (s, 3H, CH_3). ^{13}C NMR (101 MHz, $CDCl_3$) δ 176.3, 162.3, 145.2, 136.4, 135.8, 133.0, 132.5, 131.3, 129.8, 129.8, 128.7, 116.8, 21.8, 20.9. Spectroscopic data was consistent with the values reported in the literature.[2]

1-Azido-2-tosyl-1,2-dihydro-3*H*-1λ^3-benzo[*d*][1,2]iodazol-3-one (Ts-ABZ):

Caution: Even though **Ts-ABZ** has a much safer safety profile than the most commonly used azidobenziodoxolone (ABX) care has to be taken when preparing it.[3] The synthesis and filtration were carried out behind a blast shield wearing anti cut gloves (HyFlex 11-541) below regular nitrile gloves. The scale described in the procedure below was the largest scale the reaction was carried on. To synthesize larger amount of the reagent we performed the reaction in multiple batches in parallel and filtered them individually. **Ts-ABZ** batches were stored in

---

[1] V. Smyrnov, B. Muriel, J. Waser, *Org. Lett.* **2021**, *23*, 5435–5439.

[2] X.-G. Yang, F.-H. Du, J.-J. Li, C. Zhang, *Chem. - Eur. J.* **2022**, *28*, e202200272.

[3] S. Alazet, J. Preindl, R. Simonet-Davin, S. Nicolai, A. Nanchen, T. Meyer, J. Waser, *J. Org. Chem.* **2018**, *83*, 12334–12356.

plastic containers and kept in the fridge at 4 °C. During the course of this project this synthesis was performed 34 times without incident with an average yield of 86%.

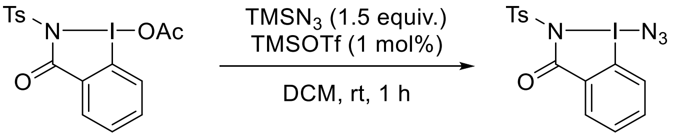

11 **Ts-ABZ**

Following a reported procedure,[1] an oven dried flask containing a solution of 3-oxo-2-tosyl-2,3-dihydro-1*H*-1λ^3-benzo[*d*][1,2]iodazol-1-yl acetate (**11**) (919 mg, 2.00 mmol, 1.0 equiv.) in dry DCM (10 mL) was cooled to 0 °C then $TMSN_3$ (0.40 mL, 3.0 mmol, 1.5 equiv.) was added dropwise followed by two drops of TMSOTf (approximation: 4.0 µL, 20 µmol, 1 mol%). The reaction was stirred at rt for 1 h and pentane (30 mL) was added to induce further precipitation (usually precipitation already starts to occur during the reaction). The solid was filtered, washed with pentane (3 x 20 mL) and dried on the frit for 2 min to afford **Ts-ABZ** (778 mg, 1.76 mmol, 88% yield) as an off white solid.

^{1}H NMR (400 MHz, $CDCl_3$) δ 8.17 (dd, *J* = 7.6, 1.6 Hz, 1H, Ar*H*), 8.07 – 8.00 (m, 3H, Ar*H*), 7.93 – 7.86 (m, 1H, Ar*H*), 7.70 (td, *J* = 7.6, 0.7 Hz, 1H, Ar*H*), 7.33 (d, *J* = 8.1 Hz, 2H, Ar*H*), 2.42 (s, 3H, C*H*$_3$). ^{13}C NMR (101 MHz, $CDCl_3$) δ 161.3, 145.1, 136.5, 136.0, 133.0, 132.8, 131.8, 129.8, 128.6, 127.3, 116.2, 21.8.

5. Synthesis of Alkenes

The following alkenes were commercially available: styrene, 4-*tert*-butylstyrene, 4-vinylbiphenyl, 2-methylstyrene, 2,4,6-trimethylstyrene, 4-vinylanisole, 4-vinylphenyl acetate, 4-bromostyrene, 3-chlorostyrene, 4-vinylbenzoate, 4-(trifluoromethyl)styrene, 2-vinylthiophene, *trans*-β-methylstyrene, indene, 1,2-dihydronaphthalene, vinyl butyl ether, 1-phenoxy-4-vinylbenzene.

General procedure A:

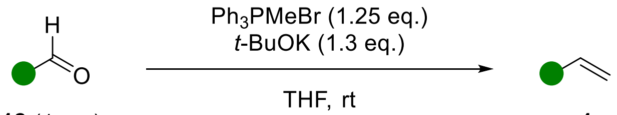

12 (1 eq.) **1**

Following a reported procedure,[4] an oven dried round-bottom flask charged with methyltriphenylphosphonium bromide (1.79 g, 5.00 mmol, 1.25 equiv.) and potassium *tert*-butoxide (584 mg, 5.20 mmol, 1.30 equiv.) was evacuated and backfilled with N_2. Dry THF (11 mL) was added and the mixture was stirred at rt for 30 min. A solution of aldehyde **12** (4.00 mmol, 1 equiv.) in dry THF (5 mL) was added dropwise over 5 min and the reaction was stirred at rt under N_2 until full conversion was observed by TLC. The reaction was quenched with 35 mL of a sat. sol. of NH_4Cl and the mixture was extracted with 3 x 40 mL of Et_2O or EtOAc. The combined organic layers were washed with brine, dried over $MgSO_4$, filtered and concentrated *in vacuo*. The crude product was purified by column chromatography to obtain alkene **1**.

But-3-en-1-yn-1-ylbenzene (1b):

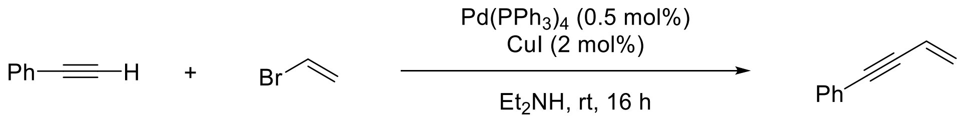

13 **14** (1.3 eq.) **1b**

Compound 1b was synthesized following a reported procedure.[5] An oven dried round-bottom flask charged with CuI (19 mg, 0.10 mmol, 2 mol%) and $Pd(PPh_3)_4$ (30 mg, 25 μmol, 0.5 mol%) was evacuated and backfilled with N_2 (3x) then degassed Et_2NH (2.5 mL) was added. The mixture was cooled to 0 °C and phenylacetylene (**13**) (0.55 mL, 5.0 mmol, 1.00 equiv.) and a solution of vinyl bromide (**14**) (6.5 mL, 6.5 mmol, 1 M in THF, 1.30 equiv.) were added. The reaction was stirred at rt for 16 h under N_2 atmosphere. The reaction was quenched with water (~15 mL) then extracted with 3 x 15 mL of a mixture of pentane/Et_2O (1:1). The combined

[4] R. J. Maza, E. Davenport, N. Miralles, J. J. Carbó, E. Fernández, *Org. Lett.* **2019**, *21*, 2251–2255.
[5] Y. Zhang, B. Yu, B. Gao, T. Zhang, H. Huang, *Org. Lett.* **2019**, *21*, 535–539.

organic layers were washed with 20 mL of 1 M aq. HCl, dried over $MgSO_4$, filtered and concentrated *in vacuo*. The crude product was loaded on celite and purified by column chromatography (pentane) to afford but-3-en-1-yn-1-ylbenzene (**1b**) (566 mg, 4.42 mmol, 88%) as a colorless oil.

^{1}H NMR (400 MHz, $CDCl_3$) δ 7.49 – 7.41 (m, 2H, Ar*H*), 7.35 – 7.29 (m, 3H, Ar*H*), 6.03 (dd, *J* = 17.5, 11.1 Hz, 1H, *H*C=CH_2), 5.74 (dd, *J* = 17.5, 2.1 Hz, 1H, HC=CH_2), 5.55 (dd, *J* = 11.1, 2.1 Hz, 1H, HC=CH_2). ^{13}C NMR (101 MHz, $CDCl_3$) δ 131.7, 128.4, 128.4, 127.0, 123.3, 117.3, 90.1, 88.2. Spectroscopic data was consistent with the values reported in the literature.[5]

1-(Trifluoromethoxy)-4-vinylbenzene (1f):

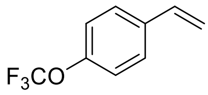

1f

Synthesized following **general procedure A** starting from 4-(trifluoromethoxy)benzaldehyde (0.57 mL, 4.0 mmol). The reaction was carried out for 1 h and extractions were performed with Et_2O. The crude product was loaded on celite and purified by column chromatography (pentane) to afford 1-(trifluoromethoxy)-4-vinylbenzene (**1f**) (441 mg, 2.34 mmol, 59%) as a colorless oil.

^{1}H NMR (400 MHz, $CDCl_3$) δ 7.46 – 7.39 (m, 2H, Ar*H*), 7.17 (d, *J* = 8.1 Hz, 2H, Ar*H*), 6.70 (dd, *J* = 17.6, 10.9 Hz, 1H, *H*C=CH_2), 5.73 (dd, *J* = 17.6, 0.5 Hz, 1H, HC=CH_2), 5.29 (dd, *J* = 10.9, 0.4 Hz, 1H, HC=CH_2).^{13}C NMR (101 MHz, $CDCl_3$) δ 148.8 (m), 136.4, 135.6, 127.6, 121.2, 120.6 (q, *J* = 257.0 Hz), 115.0. ^{19}F NMR (376 MHz, $CDCl_3$) δ -57.9. Spectroscopic data was consistent with the values reported in the literature.[6]

***N*-(4-Vinylphenyl)acetamide (1g):**

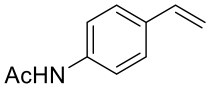

1g

Synthesized following **general procedure A** starting from 4-acetamidobenzaldehyde (653 mg, 4.00 mmol). The reaction was carried out for 3.5 h and extractions were performed with EtOAc. The crude product was purified by column chromatography (pentane/EtOAc, 6:4) to afford *N*-(4-vinylphenyl)acetamide (**1g**) (520 mg, 3.00 mmol, 75%) as a white solid.

^{1}H NMR (400 MHz, $CDCl_3$) δ 7.47 (d, *J* = 8.5 Hz, 2H, Ar*H*), 7.39 – 7.30 (m, 3H, Ar*H* + N*H*Ac), 6.67 (dd, *J* = 17.6, 10.9 Hz, 1H, *H*C=CH_2), 5.68 (d, *J* = 17.6 Hz, 1H, HC=CH_2), 5.19 (d, *J* = 10.9 Hz, 1H, HC=CH_2), 2.17 (s, 3H, CH_3). ^{13}C NMR (101 MHz, $CDCl_3$) δ 168.4, 137.6, 136.2,

[6] M. Su, X. Huang, C. Lei, J. Jin, *Org. Lett.* **2022**, *24*, 354–358.

133.9, 127.0, 119.9, 113.2, 24.8. Spectroscopic data was consistent with the values reported in the literature.[7]

4-Vinylphenol (1h):

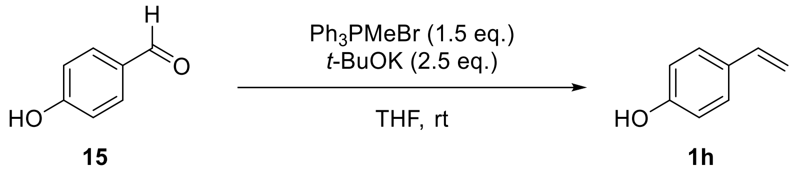

Compound **1f** was synthesized following a reported procedure.[8] An oven dried round-bottom flask charged with methyltriphenylphosphonium bromide (2.14 g, 6.00 mmol, 1.50 equiv.) and potassium *tert*-butoxide (1.12 g, 10.0 mmol, 2.50 equiv.) was evacuated and backfilled with N_2. Dry THF (11 mL) was added and the mixture was stirred at rt for 30 min. A solution of 4-hydroxybenzaldehyde (**15**) (488 mg, 4.00 mmol, 1.00 equiv.) in dry THF (5 mL) was added dropwise over 5 min and the reaction was stirred at rt for 3.5 h under N_2. The reaction was quenched with 35 mL of a sat. sol. of NH_4Cl and the mixture was extracted with 3 x 40 mL of Et_2O or EtOAc. The combined organic layers were washed with brine, dried over $MgSO_4$, filtered and concentrated *in vacuo*. The crude product was purified by column chromatography (pentane/EtOAc, 9:1) to afford 4-vinylphenol (**1f**) (388 mg, 3.23 mmol, 81%) as a white solid.

^{1}H NMR (400 MHz, $CDCl_3$) δ 7.34 – 7.28 (m, 2H, Ar*H*), 6.82 – 6.77 (m, 2H, Ar*H*), 6.65 (dd, *J* = 17.6, 10.9 Hz, 1H, *H*C=CH_2), 5.61 (dd, *J* = 17.6, 0.8 Hz, 1H, HC=CH_2), 5.13 (dd, *J* = 10.9, 0.8 Hz, 1H, HC=CH_2), 4.86 (s, 1H, O*H*). ^{13}C NMR (101 MHz, $CDCl_3$) δ 155.4, 136.3, 130.8, 127.8, 115.5, 111.8. Spectroscopic data was consistent with the values reported in the literature.[7]

2-Vinylbenzofuran (1i):

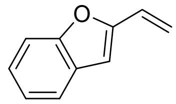

1i

Synthesized following **general procedure A** starting from 2-benzofurancarboxaldehyde (0.49 mL, 4.0 mmol). The reaction was carried out for 1 h and extractions were performed with Et_2O. The crude product was loaded on celite and purified by column chromatography (pentane) to afford 2-vinylbenzofuran (**1i**) (465 mg, 3.23 mmol, 81%) as a colorless oil.

^{1}H NMR (400 MHz, $CDCl_3$) δ 7.55 – 7.50 (m, 1H, Ar*H*), 7.48 – 7.44 (m, 1H, Ar*H*), 7.31 – 7.24 (m, 1H, Ar*H*), 7.20 (td, *J* = 7.5, 1.0 Hz, 1H, Ar*H*), 6.65 (dd, *J* = 17.5, 11.3 Hz, 1H, *H*C=CH_2), 6.60 (s, 1H, OC=C*H*), 5.97 (dd, *J* = 17.5, 0.7 Hz, 1H, HC=CH_2), 5.39 (dd, *J* = 11.2, 1.0 Hz, 1H,

[7] M.-J. Zhou, L. Zhang, G. Liu, C. Xu, Z. Huang, *J. Am. Chem. Soc.* **2021**, *143*, 16470–16485.
[8] F. C. Demidoff, F. P. de Souza, C. D. Netto, *Synthesis* **2017**, *49*, 5217–5223.

HC=CH_2). ^{13}C NMR (101 MHz, $CDCl_3$) δ 155.0, 154.9, 129.0, 125.4, 124.8, 122.9, 121.1, 115.9, 111.2, 104.9. Spectroscopic data was consistent with the values reported in the literature.[9]

Note: Product was stored at -20 °C after isolation.

2-Bromo-5-vinylfuran (1j):

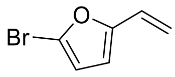

1j

Synthesized following **general procedure A** starting from 5-bromo-2-furaldehyde (700 mg, 4.00 mmol). The reaction was carried out for 1 h and extractions were performed with Et_2O. The crude product was loaded on celite and purified by column chromatography (pentane) to afford 2-bromo-5-vinylfuran (**1j**) (491 mg, 2.84 mmol, 71%) as a light orange oil.

^{1}H NMR (400 MHz, $CDCl_3$) δ 6.40 (dd, *J* = 17.5, 11.3 Hz, 1H, *H*C=CH_2), 6.29 (d, *J* = 3.3 Hz, 1H, Ar*H*), 6.21 (d, *J* = 3.3 Hz, 1H, Ar*H*), 5.65 (dd, *J* = 17.4, 0.7 Hz, 1H, HC=CH_2), 5.17 (dd, *J* = 11.3, 0.9 Hz, 1H, HC=CH_2). ^{13}C NMR (101 MHz, $CDCl_3$) δ 155.3, 124.3, 121.8, 113.1, 113.0, 110.4. Spectroscopic data was consistent with the values reported in the literature.[10]

Note: Product was stored at -20 °C after isolation. It was used the next day in the azido-alkynylation reaction. We observed substantial degradation after 2-3 days of storage.

2*H*-Chromene (1k):

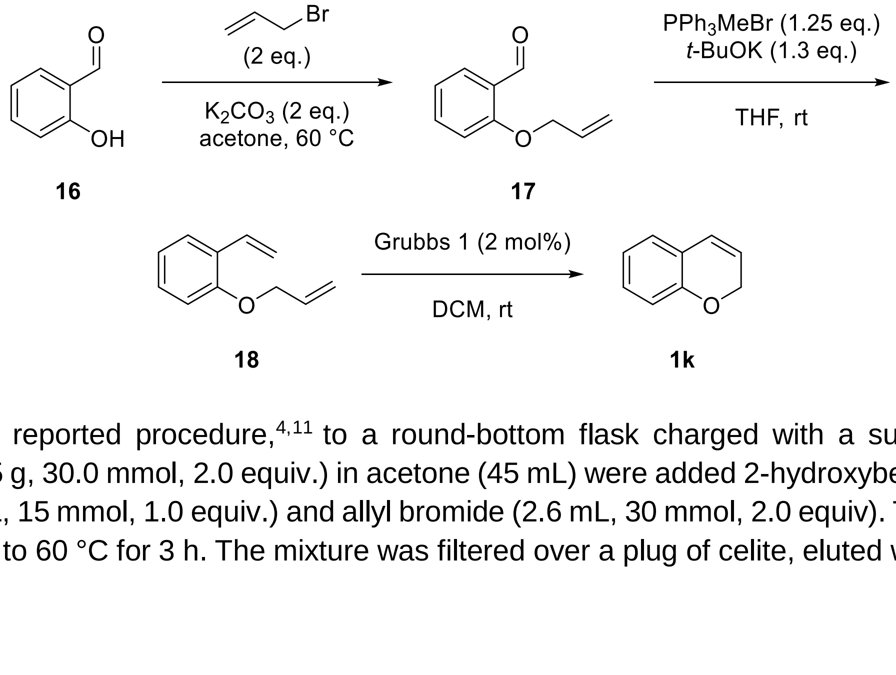

Following a reported procedure,[4,11] to a round-bottom flask charged with a suspension of K_2CO_3 (4.15 g, 30.0 mmol, 2.0 equiv.) in acetone (45 mL) were added 2-hydroxybenzaldehyde (**16**) (1.6 mL, 15 mmol, 1.0 equiv.) and allyl bromide (2.6 mL, 30 mmol, 2.0 equiv). The reaction was heated to 60 °C for 3 h. The mixture was filtered over a plug of celite, eluted with acetone

[9] H. Seo, A. Liu, T. F. Jamison, *J. Am. Chem. Soc.* **2017**, *139*, 13969–13972.
[10] Y. Yamamoto, Y. Yamada, H. Sajiki, Y. Sawama, *Bull. Chem. Soc. Jpn.* **2020**, *93*, 1419–1423.
[11] X.-S. Liang, R.-D. Li, W. Sun, Z. Liu, X.-C. Wang, *ACS Catal.* **2022**, *12*, 9153–9158.

and concentrated *in vacuo* to afford 2-(allyloxy)benzaldehyde (**17**). The crude product was used in the next step without further purification.

An oven dried round-bottom flask charged with methyltriphenylphosphonium bromide (6.7 g, 19 mmol, 1.25 equiv.) and potassium *tert*-butoxide (2.2 g, 20 mmol, 1.30 equiv.) was evacuated and backfilled with N_2. Dry THF (45 mL) was added and the mixture was stirred at rt for 30 min. A solution of crude aldehyde **17** previously prepared in dry THF (15 mL) was added dropwise over 5 min and the reaction was stirred at rt under N_2 for 1 h. The reaction was quenched with 60 mL of a sat. sol. of NH_4Cl and the mixture was extracted with 3 x 60 mL of EtOAc. The combined organic layers were washed with brine, dried over $MgSO_4$, filtered and concentrated *in vacuo*. The crude product was loaded on celite and purified by column chromatography (pentane) to afford 1-(allyloxy)-2-vinylbenzene (**18**) (1.78 g, 11.1 mmol, 74% over 2 steps) as a colorless oil.

^{1}H NMR (400 MHz, $CDCl_3$) δ 7.49 (dd, *J* = 7.6, 1.7 Hz, 1H, Ar*H*), 7.21 (ddd, *J* = 8.3, 7.5, 1.7 Hz, 1H, Ar*H*), 7.10 (dd, *J* = 17.8, 11.2 Hz, 1H, ArC*H*=CH_2), 6.97 – 6.91 (m, 1H, Ar*H*), 6.87 (dd, *J* = 8.3, 0.8 Hz, 1H, Ar*H*), 6.14 – 6.03 (m, 1H, CH_2C*H*=CH_2), 5.75 (dd, *J* = 17.8, 1.5 Hz, 1H, ArCH=CH_2), 5.43 (dq, *J* = 17.3, 1.7 Hz, 1H, CH_2CH=CH_2), 5.32 – 5.23 (m, 2H, CH_2CH=CH_2 + ArCH=CH_2), 4.57 (dt, *J* = 5.1, 1.6 Hz, 2H, OCH_2CH). ^{13}C NMR (101 MHz, $CDCl_3$) δ 155.9, 133.5, 131.8, 128.9, 127.2, 126.7, 121.0, 117.5, 114.5, 112.5, 69.3. Spectroscopic data was consistent with the values reported in the literature.[12]

Following a reported procedure,[11] to an oven-dried round-bottom flask containing a solution of 1-(allyloxy)-2-vinylbenzene (**18**) (320 mg, 2.00 mmol, 1.0 equiv.) in dry DCM (10 mL) was added Grubbs 1 catalyst (33 mg, 40 μmol, 0.02 equiv.). The reaction was stirred at rt for 2 h. The crude mixture was concentrated *in vacuo*, loaded on celite and purified by column chromatography (pentane/Et_2O, 100:0 to 97:3) to afford 2*H*-chromene (**1k**) (180 mg, 1.36 mmol, 68%) as a colorless oil.

^{1}H NMR (400 MHz, $CDCl_3$) δ 7.10 (td, *J* = 7.8, 1.7 Hz, 1H, Ar*H*), 6.96 (dd, *J* = 7.4, 1.7 Hz, 1H, Ar*H*), 6.86 (td, *J* = 7.4, 1.1 Hz, 1H, Ar*H*), 6.77 (d, *J* = 8.1 Hz, 1H, Ar*H*), 6.42 (dt, *J* = 9.9, 1.6 Hz, 1H, ArC*H*=CH), 5.77 (dt, *J* = 9.8, 3.6 Hz, 1H,CH=C*H*CH_2), 4.82 (dd, *J* = 3.6, 1.9 Hz, 2H, OCH_2CH). ^{13}C NMR (101 MHz, $CDCl_3$) δ 154.2, 129.3, 126.7, 124.7, 122.5, 122.1, 121.5, 115.9, 65.7. Spectroscopic data was consistent with the values reported in the literature.[12]

[12] F. Yang, K. Rauch, K. Kettelhoit, L. Ackermann, *Angew. Chem. Int. Ed.* **2014**, *53*, 11285–11288.

6. Synthesis of Potassium Trifluoroborate Salts

General note: It is known that carbons linked to the boron atom are difficult to be observed by ^{13}C NMR due to a broadening of the signal caused by the quadrupole moment of ^{11}B nuclei. This implies that the two carbons of the alkyne (in alkynyl-BF_3K) are too broad to be properly visible.[13] Therefore, they are not listed in the characterization data.

General procedure B:

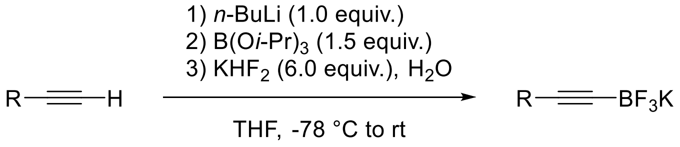

Following a reported procedure,[14,15] an oven-dried round-bottom flask (PFA), charged with alkyne (1.0 equiv.) if solid, was evacuated and backfilled with N_2 (3x). Then, alkyne (if liquid) and dry THF (0.3 M) were added. The mixture was cooled to -78 °C and a solution of *n*-BuLi (2.5 M, 1.0 equiv.) in hexane was added dropwise under N_2. The reaction was stirred at -78 °C for 1 h and B(O*i*-Pr)$_3$ (1.5 equiv.) was added quickly. The reaction was stirred 10 min at -78 °C then 2 h at rt. The mixture was cooled to 0 °C and a saturated solution of KHF_2 (6.0 equiv.) in water (40% of THF volume + additional 40% to rinse the remaining solid) was added. The reaction was stirred at rt open to air for 2 h then concentrated *in vacuo*. The wet solid obtained was further dried by co-evaporation with toluene (3x). To the dry solid was added acetone (~50 mL) and the resulting mixture was placed on a rotary evaporator and rotated rapidly at atmospheric pressure with the bath set at 45 °C for 15 minutes. The flask was removed and the mixture carefully filtered taking care to leave the insoluble material (KHF_2) in the reaction flask. Acetone was once again added and the process (heating for 15 min then collection of the liquid) was repeated 2 more times. The combined acetone filtrates were concentrated *in vacuo* to approximately 1/3 of the initial volume. Et_2O (~60 mL) was added causing a white solid to precipitate. The mixture was cooled to 0 °C for 10 min then filtered. The solid obtained was washed with Et_2O and dried in vacuo to afford the desired potassium alkynyl-trifluoroborate.

Note: This purification procedure usually affords the pure desired product. If it is not the case a more classical recrystallization from acetone/Et_2O can be performed.

Potassium trifluoro(phenylethynyl)borate (2a):

---

[13] R. A. Oliveira, R. O. Silva, G. A. Molander, P. H. Menezes, *Magn. Reson. Chem.* **2009**, *47*, 873–878.
[14] D. A. Mundal, K. E. Lutz, R. J. Thomson, *J. Am. Chem. Soc.* **2012**, *134*, 5782–5785.
[15] J. Borrel, J. Waser, *Org. Lett.* **2022**, *24*, 142–146.

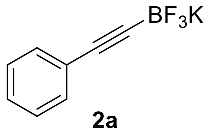

2a

Synthesized following **general procedure B** starting from phenylacetylene (1.53 g, 1.65 mL, 15.0 mmol). Potassium trifluoro(phenylethynyl)borate (**2a**) (2.60 g, 12.5 mmol, 83%) was obtained as a white solid.

^{1}H NMR (400 MHz, acetone-d$_6$) δ 7.35 – 7.29 (m, 2H, Ar*H*), 7.27 – 7.17 (m, 3H, Ar*H*). ^{13}C NMR (101 MHz, acetone-d$_6$) δ 132.1, 128.8, 127.4, 127.2. ^{19}F NMR (376 MHz, acetone-d$_6$) δ -135.0. Spectroscopic data was consistent with the values reported in the literature.[16]

Potassium ethynyltrifluoroborate (2b):

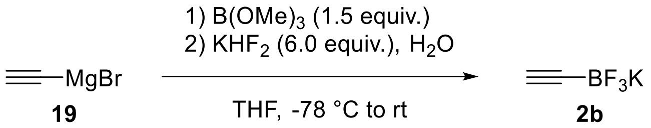

Compound **2b** was synthesized following a reported procedure.[17] An oven-dried round-bottom flask (PFA) was evacuated and backfilled with N_2 (3x). Then, a solution of ethynylmagnesium bromide (**19**) (30.0 mL, 15.0 mmol, 0.5 M, 1.0 equiv.) in THF and dry THF (30 mL) were added. The solution was cooled to -78 °C and $B(OMe)_3$ (2.5 mL, 22 mmol, 1.5 equiv.) was added quickly under N_2. The reaction was stirred 1 h at -78 °C then 1.5 h at -20 °C. A saturated solution of KHF_2 (7.03 g, 90.0 mmol, 6.0 equiv.) in water (20 mL + additional 20 mL to rinse the remaining solid) was added. The reaction was stirred at rt open air for 2 h then concentrated *in vacuo*. The wet solid obtained was further dried by co-evaporation with acetone. To the dry solid was added acetone (~30 mL) and the resulting mixture was placed on a rotary evaporator and rotated rapidly at atmospheric pressure with the bath set at 45 °C for 15 minutes. The flask was removed and the mixture carefully filtered taking care to leave the insoluble material in the reaction flask. Acetone was once again added and the process (heating for 15 min then collection of the liquid) was repeated 2 more times. The combined acetone filtrates were concentrated *in vacuo* to approximately 1/3 of the initial volume. Et_2O (~30 mL) was added causing a white solid to precipitate. The mixture was cooled to 0 °C for 10 min then filtered. The solid obtained was washed with Et_2O and dried in vacuo to afford potassium ethynyltrifluoroborate (**2b**) (1.17 g, 8.86 mmol, 59%) as a white solid.

^{1}H NMR (400 MHz, acetone-d$_6$) δ 1.67 (d, *J* = 5.4 Hz, 1H, C≡C*H*). ^{13}C NMR (101 MHz, acetone-d$_6$) not observed. ^{19}F NMR (376 MHz, acetone-d$_6$) δ -135.5. Spectroscopic data was consistent with the values reported in the literature.[15]

---

[16] G. A. Molander, B. W. Katona, F. Machrouhi, *J. Org. Chem.* **2002**, *67*, 8416–8423.
[17] P. B. Brady, E. M. Carreira, *Org. Lett.* **2015**, *17*, 3350–3353.

Potassium trifluoro((3-methoxyphenyl)ethynyl)borate (2c):

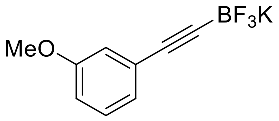

2c

Synthesized following **general procedure B** starting from 1-ethynyl-3-methoxybenzene (1.0 g, 0.97 mL, 7.5 mmol). Potassium trifluoro((3-methoxyphenyl)ethynyl)borate (**2c**) (0.91 g, 3.8 mmol, 51%) was obtained as a white solid.

^{1}H NMR (400 MHz, acetone-d$_6$) δ 7.15 (t, *J* = 8.0 Hz, 1H, Ar*H*), 6.93 – 6.84 (m, 2H, Ar*H*), 6.78 (ddd, *J* = 8.3, 2.7, 1.0 Hz, 1H, Ar*H*), 3.76 (s, 3H, OC*H$_3$*). ^{13}C NMR (101 MHz, acetone-d$_6$) δ 160.3, 129.8, 128.3, 124.5, 117.1, 113.7, 55.4. ^{19}F NMR (377 MHz, acetone-d$_6$) δ -135.0 (dd, *J* = 70.9, 30.5 Hz). Spectroscopic data was consistent with the values reported in the literature.[18]

Potassium trifluoro((4-fluorophenyl)ethynyl)borate (2d):

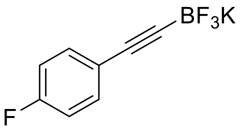

2d

Synthesized following **general procedure B** starting from 1-ethynyl-4-fluorobenzene (0.90 g, 0.86 mL, 7.5 mmol). Potassium trifluoro((4-fluorophenyl)ethynyl)borate (**2d**) (1.11 g, 4.91 mmol, 65%) was obtained as a white solid.

^{1}H NMR (400 MHz, DMSO-d$_6$) δ 7.36 – 7.28 (m, 2H, Ar*H*), 7.14 – 7.07 (m, 2H, Ar*H*). ^{1}H NMR (400 MHz, acetone-d$_6$) δ 7.38 – 7.30 (m, 2H, Ar*H*), 7.06 – 6.98 (m, 2H). ^{13}C NMR (101 MHz, acetone-d$_6$) δ 162.2 (d, *J* = 244.5 Hz), 134.0 (d, *J* = 8.0 Hz), 123.7, 115.8 (d, *J* = 21.9 Hz). ^{19}F NMR (376 MHz, acetone-d$_6$) δ -115.9, -135.1. Spectroscopic data was consistent with the values reported in the literature.[14]

Potassium ((2-chlorophenyl)ethynyl)trifluoroborate (2e):

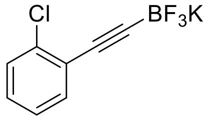

2e

[18] J. H. Song, P. Choi, S. E. Lee, K. H. Jeong, T. Kim, K. S. Kang, Y. S. Choi, J. Ham, *Eur. J. Org. Chem.* **2013**, *2013*, 6249–6253.

Synthesized following **general procedure B** starting from 1-chloro-2-ethynylbenzene (1.0 g, 0.91 mL, 7.5 mmol). Potassium ((2-chlorophenyl)ethynyl)trifluoroborate (**2e**) (712 mg, 2.94 mmol, 39%) was obtained as a white solid.

Mp (Dec.): 272 °C; ^{1}H NMR (400 MHz, Acetone) δ 7.45 – 7.39 (m, 1H, Ar*H*), 7.39 – 7.33 (m, 1H, Ar*H*), 7.23 – 7.16 (m, 2H, Ar*H*). ^{13}C NMR (101 MHz, Acetone) δ 135.6, 134.4, 129.7, 128.4, 127.3, 127.0. ^{19}F NMR (376 MHz, Acetone) δ -135.1. ^{11}B NMR (128 MHz, Acetone) δ -1.3 (q, *J* = 35.3 Hz). HRMS (ESI/QTOF) m/z: [M-K]$^-$ Calcd for $C_8H_4BClF_3^-$ 203.0052; Found 203.0053.

Potassium trifluoro((4-(methoxycarbonyl)phenyl)ethynyl)borate (2f):

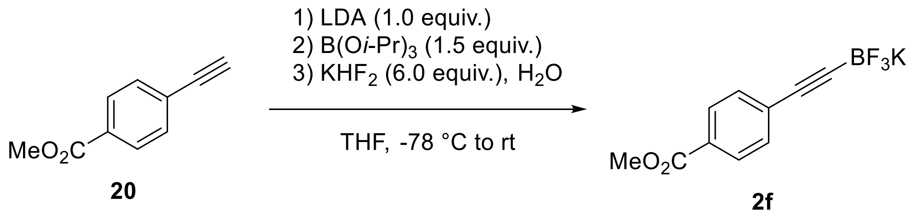

Compound **2f** was synthesized following a reported procedure.[15,19] An oven-dried round-bottom flask (PFA) was evacuated and backfilled with N_2 (3x). Then, freshly distilled diisopropylamine (1.05 mL, 7.50 mmol, 1.0 equiv.) and dry THF (15 mL) were added. The mixture was cooled to 0 °C and a solution of *n*-BuLi (3.0 mL, 7.5 mmol, 2.5 M, 1.0 equiv.) in hexane was added dropwise under N_2. The reaction was stirred at 0 °C for 0.5 h then cooled to -78 °C. A solution of methyl 4-ethynylbenzoate (**20**) (1.2 g, 7.5 mmol, 1.0 equiv.) in dry THF (10 mL) was added dropwise. The reaction was stirred at -78 °C for 0.5 h then B(O*i*-Pr)$_3$ (2.60 mL, 11.3 mmol, 1.5 equiv.) was added quickly. The reaction was stirred 10 min at -78 °C then 2 h at rt. The mixture was cooled to 0 °C and a saturated solution of KHF_2 (3.52 g, 45.0 mmol, 6.0 equiv.) in water (10 mL + additional 10 mL to rinse the remaining solid) was added. The reaction was stirred at rt open to air for 2 h then concentrated *in vacuo*. The wet solid obtained was further dried by co-evaporation with acetone. To the dry solid was added acetone (~30 mL) and the resulting mixture was placed on a rotary evaporator and rotated rapidly at atmospheric pressure with the bath set at 45 °C for 15 minutes. The flask was removed and the mixture carefully filtered taking care to leave the insoluble material in the reaction flask. Acetone was once again added and the process (heating for 15 min then collection of the liquid) was repeated 2 more times. The combined acetone filtrates were concentrated *in vacuo* to approximately 1/3 of the initial volume. Et_2O (~40 mL) was added causing a white solid to precipitate. The mixture was cooled to 0 °C for 10 min then filtered. The solid obtained was washed with Et_2O and dried in vacuo to afford potassium trifluoro((4-(methoxycarbonyl)phenyl)ethynyl)borate (**2f**) (0.80 g, 3.0 mmol, 40%) as a beige solid.

^{1}H NMR (400 MHz, acetone-d$_6$) δ 7.92 – 7.85 (m, 2H, Ar*H*), 7.45 – 7.38 (m, 2H, Ar*H*), 3.86 (s, 3H, OC*H$_3$*). ^{13}C NMR (101 MHz, acetone-d$_6$) δ 167.0, 132.5, 132.1, 129.9, 128.7, 52.3. ^{19}F

[19] For LDA preparation see: S. Jansone-Popova, J. A. May, *J. Am. Chem. Soc.* **2012**, *134*, 17877–17880.

NMR (376 MHz, acetone-d_6) δ -135.4. Spectroscopic data was consistent with the values reported in the literature.[15]

Potassium trifluoro(mesitylethynyl)borate (2g):

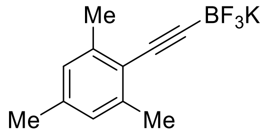

2g

Synthesized following **general procedure B** starting from 2-ethynyl-1,3,5-trimethylbenzene (0.950 g, 1.03 mL, 6.3 mmol). Potassium trifluoro(mesitylethynyl)borate (**2g**) (1.23 g, 4.94 mmol, 78%) was obtained as a white solid.

^{1}H NMR (400 MHz, acetone-d_6) δ 6.79 (s, 2H, Ar*H*), 2.34 (s, 6H, C*H$_3$*), 2.20 (s, 3H, C*H$_3$*). ^{13}C NMR (101 MHz, acetone-d_6) δ 140.0, 135.9, 127.9, 124.0, 21.3, 21.2. ^{19}F NMR (377 MHz, acetone-d_6) δ -134.3. Spectroscopic data was consistent with the values reported in the literature.[15]

Potassium trifluoro(thiophen-3-ylethynyl)borate (2h):

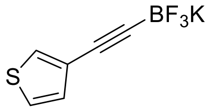

2h

Synthesized following **general procedure B** starting from 3-ethynylthiophene (0.85 g, 0.77 mL, 7.5 mmol). Potassium trifluoro(thiophen-3-ylethynyl)borate (**2h**) (1.29 g, 6.01 mmol, 80%) was obtained as a light brown solid.

Mp (Dec.): 248 °C; ^{1}H NMR (400 MHz, Acetone) δ 7.33 (dd, J = 4.9, 3.0 Hz, 1H, Ar*H*), 7.29 (dd, J = 3.0, 1.2 Hz, 1H, Ar*H*), 7.00 (dd, J = 4.9, 1.2 Hz, 1H, Ar*H*). ^{13}C NMR (101 MHz, Acetone) δ 131.0, 126.9, 126.4, 125.5. ^{19}F NMR (376 MHz, Acetone) δ -135.0. ^{11}B NMR (128 MHz, Acetone) δ -1.3 (q, J = 36.4 Hz). HRMS (ESI/QTOF) m/z: [M-K]$^-$ Calcd for $C_6H_3BF_3S^-$ 175.0006; Found 175.0012.

2-Ethynylbenzofuran (19):

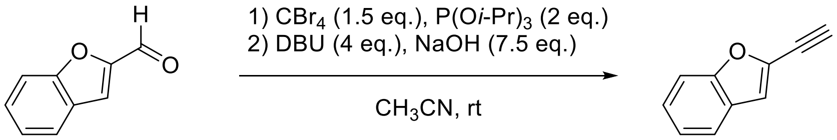

21 **22**

Compound **22** was synthesized following a reported procedure.[20] An oven-dried round-bottom flask charged with CBr_4 (5.0 g, 15 mmol, 1.5 equiv.) was evacuated and backfilled with N_2 (3x) then dry CH_3CN (20 mL) and 2-benzofurancarboxaldehyde (**21**) (1.2 mL, 10 mmol, 1.0 equiv.) were added. The mixture was cooled to 0 °C and triisopropyl phosphite (4.9 mL, 20 mmol, 2.0 equiv) was added dropwise over 5 min then DBU (6.0 mL, 40 mmol, 4.0 equiv) was added dropwise over 15 minutes. The mixture was stirred at 0 °C for 10 min then at rt for 20 min. Grinded NaOH (3.0 g, 75 mmol, 7.5 equiv) was added and the reaction was stirred at rt for 4 h under N_2 atmosphere. Water (30 mL) and brine (50 mL) were added and the mixture was extracted with 3 x 70 mL of EtOAc. The combined organic layers were washed with brine (100 mL), dried over $MgSO_4$, filtered and concentrated *in vacuo*. The crude product was loaded on celite and purified by column chromatography (pentane) to afford 2-ethynylbenzofuran (**22**) (516 mg, 3.63 mmol, 36%) as an orange oil.

^{1}H NMR (400 MHz, $CDCl_3$) δ 7.59 – 7.54 (m, 1H, Ar*H*), 7.49 – 7.44 (m, 1H, Ar*H*), 7.36 (td, *J* = 7.8, 1.3 Hz, 1H, Ar*H*), 7.30 – 7.21 (m, 1H, Ar*H*), 7.01 (d, *J* = 0.5 Hz, 1H, OC=C*H*), 3.50 (s, 1H, C≡C*H*). ^{13}C NMR (101 MHz, $CDCl_3$) δ 154.9, 137.8, 127.3, 126.1, 123.5, 121.5, 112.8, 111.5, 83.5, 74.2. Spectroscopic data was consistent with the values reported in the literature.[20]

Potassium (benzofuran-2-ylethynyl)trifluoroborate (2i):

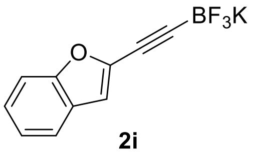

2i

Synthesized following **general procedure B** starting from 2-ethynylbenzofuran (**22**) (500 mg, 3.52 mmol). After recrystallization (acetone/Et_2O) potassium (benzofuran-2-ylethynyl)trifluoroborate (**2i**) (568 mg, 2.29 mmol, 65%) was obtained as a white solid.

^{1}H NMR (400 MHz, DMSO) δ 7.59 – 7.54 (m, 1H, Ar*H*), 7.51 – 7.45 (m, 1H, Ar*H*), 7.30 (dd, *J* = 6.0, 1.4 Hz, 1H, Ar*H*), 7.22 (td, *J* = 7.6, 1.0 Hz, 1H, Ar*H*), 6.92 (s, 1H, OC=C*H*). ^{13}C NMR (101 MHz, DMSO) δ 153.5, 140.4, 127.7, 124.8, 123.1, 120.9, 110.7, 108.6. Spectroscopic data was consistent with the values reported in the literature.[21]

Potassium trifluoro(prop-1-yn-1-yl)borate (2j):

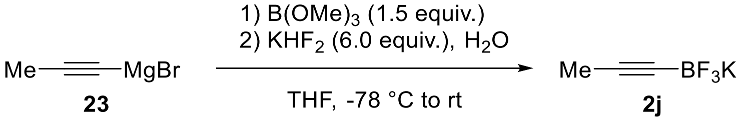

Compound **2j** was synthesized following a reported procedure.[15] An oven-dried round-bottom flask (PFA) was evacuated and backfilled with N_2 (3x). Then, a solution of 1-

---

[20] Y. Thummala, G. V. Karunakar, V. R. Doddi, *Adv. Synth. Catal.* **2019**, *361*, 611–616.

[21] J.-F. Wang, X. Meng, C.-H. Zhang, C.-M. Yu, B. Mao, *Org. Lett.* **2020**, *22*, 7427–7432.

propynylmagnesium bromide (**23**) (15 mL, 7.5 mmol, 0.5 M, 1.0 equiv.) in THF and dry THF (15 mL) were added. The solution was cooled to -78 °C and $B(OMe)_3$ (1.25 mL, 11.3 mmol, 1.5 equiv.) was added quickly under N_2. The reaction was stirred 1 h at -78 °C then 1.5 h at -20 °C. A saturated solution of KHF_2 (3.5 g, 45 mmol, 6.0 equiv.) in water (10 mL + additional 10 mL to rinse the remaining solid) was added. The reaction was stirred at rt open air for 2 h then concentrated *in vacuo*. The wet solid obtained was further dried by co-evaporation with acetone. To the dry solid was added acetone (~30 mL) and the resulting mixture was placed on a rotary evaporator and rotated rapidly at atmospheric pressure with the bath set at 45 °C for 15 minutes. The flask was removed and the mixture carefully filtered taking care to leave the insoluble material in the reaction flask. Acetone was once again added and the process (heating for 15 min then collection of the liquid) was repeated 2 more times. The combined acetone filtrates were concentrated *in vacuo* to approximately 1/3 of the initial volume. Et_2O (~30 mL) was added causing a white solid to precipitate. The mixture was cooled to 0 °C for 10 min then filtered. The solid obtained was washed with Et_2O and dried in vacuo to afford potassium trifluoro(prop-1-yn-1-yl)borate (**2j**) (0.95 g, 6.5 mmol, 87%) as a white solid.

^1H NMR (400 MHz, acetone-d_6) δ 1.64 – 1.58 (m, 3H, C*H*$_3$). ^{13}C NMR (101 MHz, acetone-d_6) δ 4.0. ^{19}F NMR (376 MHz, acetone-d_6) δ -134.7. Spectroscopic data was consistent with the values reported in the literature.[15]

Potassium (cyclopropylethynyl)trifluoroborate (2k):

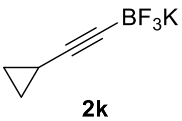

2k

Synthesized following **general procedure B** starting from ethynylcyclopropane (0.50 g, 0.64 mL, 7.5 mmol). Potassium (cyclopropylethynyl)trifluoroborate (**2k**) (0.86 g, 5.0 mmol, 67%) was obtained as a white solid.

^1H NMR (400 MHz, DMSO-d_6) δ 1.12 – 1.01 (m, 1H, C*H*), 0.61 – 0.54 (m, 2H, C*H*$_2$), 0.42 – 0.36 (m, 2H, C*H*$_2$). ^{13}C NMR (101 MHz, DMSO-d_6) δ 7.4, 0.1. ^{19}F NMR (377 MHz, DMSO-d_6) δ -131.1. Spectroscopic data was consistent with the values reported in the literature.[15]

Potassium (5-chloropent-1-yn-1-yl)trifluoroborate (2l):

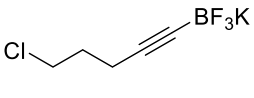

2l

Synthesized following **general procedure B** starting from 5-chloropent-1-yne (0.77 g, 0.80 mL, 7.5 mmol). Potassium (5-chloropent-1-yn-1-yl)trifluoroborate (**2l**) (1.28 g, 6.14 mmol, 82%) was obtained as a white solid.

^1H NMR (400 MHz, acetone-d_6) δ 3.70 (t, *J* = 6.6 Hz, 2H, C*H*$_2$Cl), 2.24 – 2.17 (m, 2H, C≡C-C*H*$_2$), 1.85 (p, *J* = 6.7 Hz, 2H, CH_2C*H*$_2$$CH_2$). ^{13}C NMR (101 MHz, acetone-d_6) δ 44.9, 33.1,

17.3. ^{19}F NMR (376 MHz, acetone-d_6) δ -134.6. Spectroscopic data was consistent with the values reported in the literature.[16]

7. Azido-Alkynylation

General Note: We observed that the homopropargylic azides synthetized in this work tend to slowly decompose even when stored in the fridge at 4 °C

7.1 Scope of Alkenes

General procedure C:

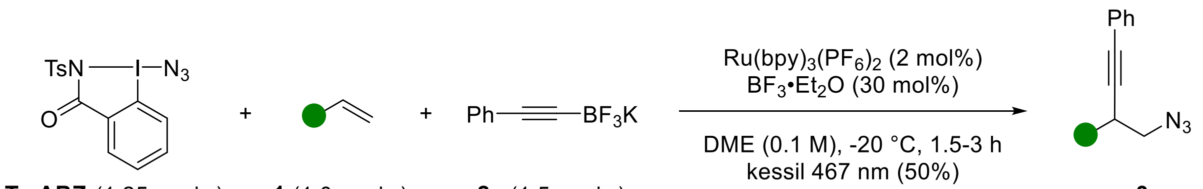

Ts-ABZ (1.25 equiv.) **1** (1.0 equiv.) **2a** (1.5 equiv.) **3**

An oven-dried test tube charged with $Ru(bpy)_3(PF_6)_2$ (5.2 mg, 6.0 μmol, 0.02 equiv.), **Ts-ABZ** (166 mg, 0.375 mmol, 1.25 equiv.), potassium trifluoro(phenylethynyl)borate (**2a**) (94 mg, 0.45 mmol, 1.50 equiv.) and alkene (**1**) (if solid, 0.30 mmol, 1.00 equiv.) was evacuated and backfilled with N_2 (3x). Dry degassed DME (2.7 mL) and alkene (**1**) (if liquid, 0.30 mmol, 1.00 equiv.) were added and the mixture was cooled to -20 °C. Then, a stock solution of BF_3•Et_2O (11 μL, 90 μmol, 0.30 equiv.) in dry degassed DME (0.34 mL) was added and the reaction was stirred under blue LEDs irradiation (1 x Kessil 467 nm, 50% intensity 22 W) at -20 °C until full conversion was observed (1.5-3 h). The reaction mixture was filtered through a short plug of silica and eluted with DCM or EtOAc then concentrated *in vacuo*. The crude product was purified by column chromatography to afford **3**.

Note: Commercially available liquid alkenes were eluted through a short plug of basic Al_2O_3 before use. DME was sparged with argon for 0.5 h before use. Cooling was performed using a Dewar filled with a mixture of ice and salt. We did not observe significant rise in temperature after 1.5 h (which is enough in most cases to reach full conversion). In the case of longer reactions, the cold bath was replaced with a new one after 1.5 h. For further details on the photochemistry set-up see Figure S1.

(4-Azidobut-1-yne-1,3-diyl)dibenzene (3a):

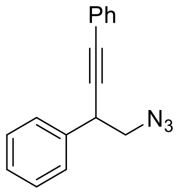

3a

Synthesized following **general procedure C** starting from styrene (**1a**) (35 µL, 0.30 mmol, 1.00 equiv.). The reaction was carried out for 1.5 h, DCM was used for the silica plug. The crude product was purified by column chromatography (pentane/toluene, 85:15) to afford (4-azidobut-1-yne-1,3-diyl)dibenzene (**3a**) (55 mg, 0.22 mmol, 73%) as a yellow oil.

R_f (pentane/toluene, 7:3): 0.45; ^{1}H NMR (400 MHz, $CDCl_3$) δ 7.52 – 7.45 (m, 4H, Ar*H*), 7.42 – 7.36 (m, 2H, Ar*H*), 7.35 – 7.30 (m, 4H, Ar*H*), 4.13 (t, *J* = 6.8 Hz, 1H, C*H*C≡C), 3.63 (dd, *J* = 12.0, 7.6 Hz, 1H, C$H_2$$N_3$), 3.53 (dd, *J* = 12.0, 6.2 Hz, 1H, C$H_2$$N_3$). ^{13}C NMR (101 MHz, $CDCl_3$) δ 138.1, 131.7, 128.8, 128.3, 128.3, 127.9, 127.8, 123.0, 87.9, 84.9, 57.3, 39.6. HRMS (APPI/LTQ-Orbitrap) m/z: $[M-N_2+H]^+$ Calcd for $C_{16}H_{14}N^+$ 220.1121; Found 220.1120.

1-(1-Azido-4-phenylbut-3-yn-2-yl)-4-(tert-butyl)benzene (3b):

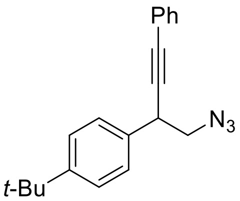

3b

Synthesized following **general procedure C** starting from 4-*tert*-butylstyrene (55 µL, 0.30 mmol, 1.00 equiv.). The reaction was carried out for 1.5 h, DCM was used for the silica plug. The crude product was purified by column chromatography (pentane/toluene, 95:5 to 90:10) to afford 1-(1-azido-4-phenylbut-3-yn-2-yl)-4-(tert-butyl)benzene (**3b**) (71 mg, 0.24 mmol, 78%) as a colorless oil.

R_f (pentane/toluene, 85 :15) : 0.27; ^{1}H NMR (400 MHz, $CDCl_3$) δ 7.54 – 7.48 (m, 2H, Ar*H*), 7.45 – 7.39 (m, 4H, Ar*H*), 7.36 – 7.30 (m, 3H, Ar*H*), 4.12 (dd, *J* = 7.7, 6.1 Hz, 1H, C*H*C≡C), 3.63 (dd, *J* = 12.0, 7.8 Hz, 1H, C$H_2$$N_3$), 3.53 (dd, *J* = 12.0, 6.1 Hz, 1H, C$H_2$$N_3$), 1.35 (s, 9H, *t*-Bu). ^{13}C NMR (101 MHz, $CDCl_3$) δ 150.8, 135.2, 131.8, 128.4, 128.3, 127.6, 125.9, 123.2, 88.3, 84.8, 57.4, 39.3, 34.7, 31.5. HRMS (ESI/QTOF) m/z: $[M+H]^+$ Calcd for $C_{20}H_{22}N_3^+$ 304.1808; Found 304.1804.

4-(1-Azido-4-phenylbut-3-yn-2-yl)-1,1'-biphenyl (3c):

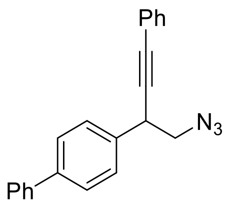

3c

Synthesized following **general procedure C** starting from 4-vinylbiphenyl (54 mg, 0.30 mmol, 1.00 equiv.). The reaction was carried out for 1.5 h, DCM was used for the silica plug. The crude product was purified by column chromatography (pentane/toluene, 90:10 to 80:20) to afford 4-(1-azido-4-phenylbut-3-yn-2-yl)-1,1'-biphenyl (**3c**) (74 mg, 0.23 mmol, 76%) as a yellow oil.

R_f (pentane/toluene, 7:3): 0.48; ^{1}H NMR (400 MHz, $CDCl_3$) δ 7.66 – 7.60 (m, 4H, Ar*H*), 7.58 – 7.51 (m, 4H, Ar*H*), 7.49 – 7.44 (m, 2H, Ar*H*), 7.41 – 7.32 (m, 4H, Ar*H*), 4.19 (t, *J* = 6.6 Hz, 1H, C*H*C≡C), 3.69 (dd, *J* = 12.0, 7.6 Hz, 1H, CH_2N_3), 3.59 (dd, *J* = 12.0, 6.2 Hz, 1H, CH_2N_3). ^{13}C NMR (101 MHz, $CDCl_3$) δ[22] 140.9, 140.7, 137.3, 131.9, 128.9, 128.4, 128.4, 127.7, 127.5, 127.2, 123.1, 88.0, 85.1, 57.3, 39.4. HRMS (ESI/QTOF) m/z: $[M+H]^+$ Calcd for $C_{22}H_{18}N_3^+$ 324.1495; Found 324.1497.

1-(1-Azido-4-phenylbut-3-yn-2-yl)-2-methylbenzene (3d):

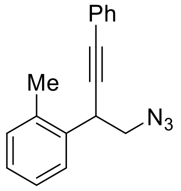

3d

Synthesized following **general procedure C** starting from 2-methylstyrene (39 μL, 0.30 mmol, 1.00 equiv.). The reaction was carried out for 1.5 h, DCM was used for the silica plug. The crude product was purified by column chromatography (pentane/toluene, 90:10 to 85:15) to afford 1-(1-azido-4-phenylbut-3-yn-2-yl)-2-methylbenzene (**3d**) (62 mg, 0.24 mmol, 79%) as a yellow oil.

R_f (pentane/toluene, 7:3): 0.57; ^{1}H NMR (400 MHz, $CDCl_3$) δ 7.65 – 7.61 (m, 1H, Ar*H*), 7.51 – 7.45 (m, 2H, Ar*H*), 7.34 – 7.29 (m, 3H, Ar*H*), 7.29 – 7.18 (m, 3H, Ar*H*), 4.35 (dd, *J* = 8.1, 5.8 Hz, 1H, C*H*C≡C), 3.60 (dd, *J* = 12.0, 8.2 Hz, 1H, CH_2N_3), 3.49 (dd, *J* = 12.0, 5.8 Hz, 1H, CH_2N_3), 2.42 (s, 3H, CH_3). ^{13}C NMR (101 MHz, $CDCl_3$) δ 136.4, 135.4, 131.8, 130.9, 128.4, 128.3, 128.2, 127.8, 126.8, 123.2, 88.7, 84.3, 56.2, 36.2, 19.4. HRMS (Sicrit plasma/LTQ-Orbitrap) m/z: $[M-N_2+H]^+$ Calcd for $C_{17}H_{16}N^+$ 234.1277; Found 234.1277.

2-(1-Azido-4-phenylbut-3-yn-2-yl)-1,3,5-trimethylbenzene (3e):

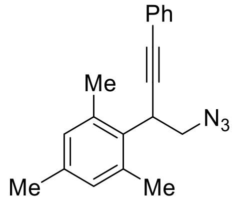

3e

Synthesized following **general procedure C** starting from 2,4,6-trimethylstyrene (48 μL, 0.30 mmol, 1.00 equiv.). The reaction was carried out for 1.5 h, DCM was used for the silica plug. The crude product was purified by column chromatography (pentane/Et_2O, 97.5:2.5) to

[22] One aromatic carbon was not resolved.

afford 2-(1-azido-4-phenylbut-3-yn-2-yl)-1,3,5-trimethylbenzene (**3e**) (68 mg, 0.24 mmol, 78%) as a yellow oil.

R_f (pentane/Et_2O, 95:5): 0.5; ^1H NMR (400 MHz, $CDCl_3$) δ 7.48 – 7.43 (m, 2H, Ar*H*), 7.35 – 7.30 (m, 3H, Ar*H*), 6.91 (s, 2H, Ar*H*), 4.61 (dd, *J* = 8.6, 7.0 Hz, 1H, C*H*C≡C), 3.83 (dd, *J* = 12.0, 8.8 Hz, 1H, C*H*$_2$N$_3$), 3.49 (dd, *J* = 12.0, 6.9 Hz, 1H, C*H*$_2$N$_3$), 2.54 (s, 6H, C*H*$_3$), 2.30 (s, 3H, C*H*$_3$). ^{13}C NMR (101 MHz, $CDCl_3$) δ 137.2, 136.8, 131.6, 131.0, 130.4, 128.4, 128.1, 123.4, 88.5, 84.1, 53.9, 34.1, 21.0, 20.9. HRMS (APPI/LTQ-Orbitrap) m/z: $[M-N_2+H]^+$ Calcd for $C_{19}H_{20}N^+$ 262.1590; Found 262.1590.

1-(1-Azido-4-phenylbut-3-yn-2-yl)-4-methoxybenzene (3f):

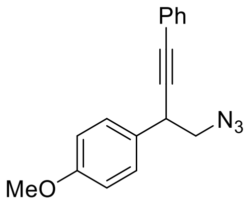

3f

Synthesized following **general procedure C** starting from 4-vinylanisole (40 µL, 0.30 mmol, 1.00 equiv.). The reaction was carried out for 1.5 h, DCM was used for the silica plug. The crude product was purified by column chromatography (pentane/Et_2O, 97.5:2.5 to 95:5) to afford 1-(1-azido-4-phenylbut-3-yn-2-yl)-4-methoxybenzene (**3f**) (70 mg, 0.25 mmol, 84%) as a yellow oil.

R_f (pentane/Et_2O, 9:1): 0.35; ^1H NMR (400 MHz, $CDCl_3$) δ 7.51 – 7.46 (m, 2H, Ar*H*), 7.41 – 7.36 (m, 2H, Ar*H*), 7.34 – 7.30 (m, 3H, Ar*H*), 6.95 – 6.89 (m, 2H, Ar*H*), 4.10 – 4.05 (m, 1H, C*H*C≡C), 3.82 (s, 3H, OC*H*$_3$), 3.60 (dd, *J* = 12.0, 7.6 Hz, 1H, C*H*$_2$N$_3$), 3.50 (dd, *J* = 12.0, 6.3 Hz, 1H, C*H*$_2$N$_3$). ^{13}C NMR (101 MHz, $CDCl_3$) δ 159.3, 131.8, 130.3, 129.0, 128.4, 128.4, 123.1, 114.3, 88.4, 84.8, 57.5, 55.5, 38.9. HRMS (APPI/LTQ-Orbitrap) m/z: $[M-N_2+H]^+$ Calcd for $C_{17}H_{16}NO^+$ 250.1226; Found 250.1226.

4-(1-Azido-4-phenylbut-3-yn-2-yl)phenyl acetate (3g):

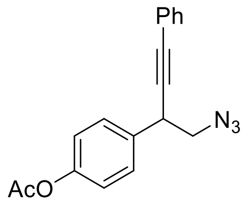

3g

Synthesized following **general procedure C** starting from 4-vinylphenyl acetate (**1e**) (46 µL, 0.30 mmol, 1.00 equiv.). The reaction was carried out for 1.5 h, DCM was used for the silica plug. The crude product was purified by column chromatography (pentane/Et_2O, 90:10 to 85:15) to afford 4-(1-azido-4-phenylbut-3-yn-2-yl)phenyl acetate (**3g**) (71 mg, 0.23 mmol, 78%) as a yellow oil.

R_f (pentane/Et_2O, 8:2): 0.33; ^1H NMR (400 MHz, $CDCl_3$) δ 7.52 – 7.44 (m, 4H, Ar*H*), 7.36 – 7.30 (m, 3H, Ar*H*), 7.14 – 7.08 (m, 2H, Ar*H*), 4.13 (dd, *J* = 7.5, 6.2 Hz, 1H, C*H*C≡C), 3.62 (dd, *J* = 12.0, 7.6 Hz, 1H, CH_2N_3), 3.52 (dd, *J* = 12.0, 6.1 Hz, 1H, CH_2N_3), 2.31 (s, 3H, CH_3). ^{13}C NMR (101 MHz, $CDCl_3$) δ 169.5, 150.2, 135.8, 131.8, 129.0, 128.5, 128.4, 122.9, 122.0, 87.7, 85.2, 57.3, 39.2, 21.2. HRMS (ESI/QTOF) m/z: $[M+Na]^+$ Calcd for $C_{18}H_{15}N_3NaO_2^+$ 328.1056; Found 328.1056.

1-(1-Azido-4-phenylbut-3-yn-2-yl)-4-(trifluoromethoxy)benzene (3h):

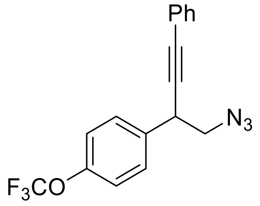

3h

Synthesized following **general procedure C** starting from a solution of 1-(trifluoromethoxy)-4-vinylbenzene (**1f**) (56 mg, 0.30 mmol, 1.00 equiv.) in dry degassed DME (0.5 mL). The reaction was carried out for 1.5 h, DCM was used for the silica plug. The crude product was purified by column chromatography (pentane/toluene, 9:1) to afford 1-(1-azido-4-phenylbut-3-yn-2-yl)-4-(trifluoromethoxy)benzene (**3h**) (59 mg, 0.18 mmol, 59%) as a colorless oil.

R_f (pentane/toluene, 85:15): 0.35; ^1H NMR (400 MHz, $CDCl_3$) δ 7.52 – 7.46 (m, 4H, Ar*H*), 7.35 – 7.31 (m, 3H, Ar*H*), 7.24 (d, *J* = 8.0 Hz, 2H, Ar*H*), 4.14 (t, *J* = 6.8 Hz, 1H, C*H*C≡C), 3.63 (dd, *J* = 12.0, 7.2 Hz, 1H, CH_2N_3), 3.53 (dd, *J* = 12.0, 6.3 Hz, 1H, CH_2N_3). ^{13}C NMR (101 MHz, $CDCl_3$) δ 148.9 (q, *J* = 1.8 Hz), 137.0, 131.8, 129.4, 128.6, 128.5, 122.8, 121.4, 120.6 (q, *J* = 257.2 Hz), 87.3, 85.5, 57.3, 39.1. ^{19}F NMR (376 MHz, CDCl3) δ -57.9. HRMS (APPI/LTQ-Orbitrap) m/z: $[M-N_2+H]^+$ Calcd for $C_{17}H_{13}F_3NO^+$ 304.0944; Found 304.0946

***N*-(4-(1-Azido-4-phenylbut-3-yn-2-yl)phenyl)acetamide (3i):**

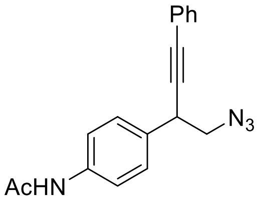

3i

Synthesized following **general procedure C** starting from *N*-(4-vinylphenyl)acetamide (**1g**) (52 mg, 0.30 mmol, 1.00 equiv.). The reaction was carried out for 1.5 h, EtOAc was used for the silica plug. The crude product was dissolved in DCM (15 mL), the solution was washed with 3 x 10 mL of a sat. sol. of $NaHCO_3$. The organic layer was dried over $MgSO_4$, filtered and concentrated *in vacuo*. The crude product was purified by column chromatography (pentane/EtOAc, 6:4 to 5:5) to afford *N*-(4-(1-azido-4-phenylbut-3-yn-2-yl)phenyl)acetamide (**3i**) (62 mg, 0.20 mmol, 68%) as a yellow solid.

R_f (pentane/EtOAc, 4:6): 0.45; Mp (Dec.): 139 °C; ^{1}H NMR (400 MHz, $CDCl_3$) δ 7.55 – 7.44 (m, 4H, Ar*H*), 7.41 (d, *J* = 8.4 Hz, 2H, Ar*H*), 7.35 – 7.29 (m, 3H, Ar*H*), 7.22 (bs, 1H, N*H*Ac), 4.09 (t, *J* = 6.8 Hz, 1H, C*H*C≡C), 3.60 (dd, *J* = 12.0, 7.5 Hz, 1H, C$H_2$$N_3$), 3.50 (dd, *J* = 12.0, 6.2 Hz, 1H, C$H_2$$N_3$), 2.18 (s, 3H, C$H_3$). ^{13}C NMR (101 MHz, $CDCl_3$) δ[23] 168.4, 137.5, 134.1, 131.8, 128.6, 128.4, 123.0, 120.3, 87.9, 85.1, 57.4, 39.2, 24.8. HRMS (ESI/QTOF) m/z: $[M+Na]^+$ Calcd for $C_{18}H_{16}N_4NaO^+$ 327.1216; Found 327.1212.

4-(1-Azido-4-(3-methoxyphenyl)but-3-yn-2-yl)phenol (3j):

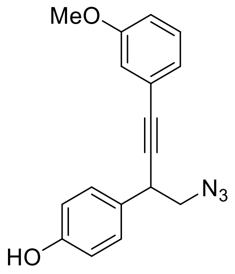

3j

An oven-dried test tube charged with $Ru(bpy)_3(PF_6)_2$ (5.2 mg, 6.0 μmol, 2 mol%), **Ts-ABZ** (166 mg, 0.375 mmol, 1.25 equiv.), potassium trifluoro((3-methoxyphenyl)ethynyl)borate (**2c**) (107 mg, 0.450 mmol, 1.50 equiv.) and 4-vinylphenol (**1h**) (36 mg, 0.30 mmol, 1.00 equiv.) was evacuated and backfilled with N_2 (3x). Dry degassed DME (2.7 mL) was added and the mixture was cooled to -20 °C. Then, a stock solution of BF_3•Et_2O (11.1 μL, 90.0 μmol, 0.30 equiv.) in dry degassed DME (0.34 mL) was added and the reaction was stirred under blue LEDs irradiation (1 x Kessil 467 nm 50% intensity, 22W) at -20 °C for 1.5 h. The reaction mixture was filtered through a short plug of silica and eluted with DCM then concentrated *in vacuo*. The crude product was purified by column chromatography (pentane/EtOAc, 85:15 to 80:20) to afford 4-(1-azido-4-(3-methoxyphenyl)but-3-yn-2-yl)phenol (**3j**) (41 mg, 0.14 mmol, 47%) as an orange oil.

R_f (pentane/EtOAc, 75:25): 0.32; ^{1}H NMR (400 MHz, $CDCl_3$) δ 7.35 – 7.30 (m, 2H, Ar*H*), 7.25 – 7.19 (m, 1H Ar*H*), 7.11 – 7.06 (m, 1H Ar*H*), 7.02 (dd, *J* = 2.4, 1.4 Hz, 1H Ar*H*), 6.89 (ddd, *J* = 8.4, 2.6, 0.8 Hz, 1H Ar*H*), 6.87 – 6.81 (m, 2H Ar*H*), 5.19 (bs, 1H, O*H*), 4.06 (t, *J* = 6.9 Hz, 1H, C*H*C≡C), 3.80 (s, 3H, OCH_3), 3.59 (dd, *J* = 12.0, 7.5 Hz, 1H, C$H_2$$N_3$), 3.49 (dd, *J* = 12.0, 6.2 Hz, 1H, C$H_2$$N_3$). ^{13}C NMR (101 MHz, $CDCl_3$) δ 159.4, 155.2, 130.4, 129.5, 129.2, 124.4, 124.1, 116.6, 115.8, 115.0, 88.2, 84.8, 57.5, 55.4, 38.9. HRMS (APPI/LTQ-Orbitrap) m/z: $[M-N_2+H]^+$ Calcd for $C_{17}H_{16}NO_2^+$ 266.1176; Found 266.1176.

1-(1-Azido-4-phenylbut-3-yn-2-yl)-4-bromobenzene (3k):

[23] One aromatic carbon was not resolved.

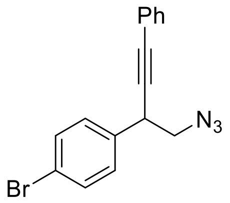

3k

Synthesized following **general procedure C** starting from 4-bromostyrene (41 µL, 0.30 mmol, 1.00 equiv.). The reaction was carried out for 1.5 h, DCM was used for the silica plug. The crude product was purified by column chromatography (pentane/toluene, 90:10 to 85:15) to afford 1-(1-azido-4-phenylbut-3-yn-2-yl)-4-bromobenzene (**3k**) (69 mg, 0.21 mmol, 71%) as a yellow oil.

R_f (pentane/toluene, 7:3): 0.48; ^{1}H NMR (400 MHz, $CDCl_3$) δ 7.54 – 7.46 (m, 4H, Ar*H*), 7.38 – 7.30 (m, 5H, Ar*H*), 4.08 (t, *J* = 6.8 Hz, 1H, C*H*C≡C), 3.62 (dd, *J* = 12.0, 7.3 Hz, 1H, C$H_2$$N_3$), 3.52 (dd, *J* = 12.0, 6.3 Hz, 1H, C$H_2$$N_3$). ^{13}C NMR (101 MHz, $CDCl_3$) δ 137.3, 132.0, 131.8, 129.7, 128.6, 128.5, 122.8, 121.8, 87.3, 85.4, 57.1, 39.2. HRMS (Sicrit plasma/LTQ-Orbitrap) m/z: $[M+H]^+$ Calcd for $C_{16}H_{13}BrN_3^+$ 326.0287; Found 326.0286.

1-(1-Azido-4-phenylbut-3-yn-2-yl)-3-chlorobenzene (3l):

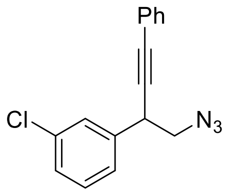

3l

Synthesized following **general procedure C** starting from 3-chlorostyrene (38 µL, 0.30 mmol, 1.00 equiv.). The reaction was carried out for 1.5 h, DCM was used for the silica plug. The crude product was purified by column chromatography (pentane/toluene, 90:10 to 85:15) to afford 1-(1-azido-4-phenylbut-3-yn-2-yl)-3-chlorobenzene (**3l**) (45 mg, 0.16 mmol, 53%) as a yellow oil.

R_f (pentane/toluene, 8:2): 0.24; ^{1}H NMR (400 MHz, $CDCl_3$) δ 7.52 – 7.45 (m, 3H, Ar*H*), 7.37 – 7.28 (m, 6H, Ar*H*), 4.10 (t, *J* = 6.8 Hz, 1H, C*H*C≡C), 3.63 (dd, *J* = 12.0, 7.3 Hz, 1H, C$H_2$$N_3$), 3.54 (dd, *J* = 12.0, 6.3 Hz, 1H, C$H_2$$N_3$). ^{13}C NMR (101 MHz, $CDCl_3$) δ 140.3, 134.8, 131.9, 130.2, 128.6, 128.5, 128.2, 128.1, 126.2, 122.8, 87.1, 85.5, 57.2, 39.4. HRMS (APPI/LTQ-Orbitrap) m/z: $[M-N_2+H]^+$ Calcd for $C_{16}H_{13}ClN^+$ 254.0731; Found 254.0737.

Methyl 4-(1-azido-4-phenylbut-3-yn-2-yl)benzoate (3m):

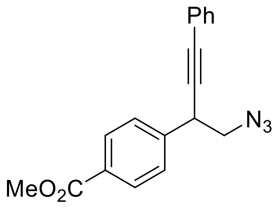

3m

Synthesized following **general procedure C** starting from methyl 4-vinylbenzoate (49 mg, 0.30 mmol, 1.00 equiv.). The reaction was carried out for 3 h, DCM was used for the silica plug. The crude product was purified by column chromatography (pentane/Et_2O, 9:1) to afford methyl 4-(1-azido-4-phenylbut-3-yn-2-yl)benzoate (**3m**) (45 mg, 0.18 mmol, 49%) as a yellow oil.

R_f (pentane/Et_2O, 8:2): 0.45; ^{1}H NMR (400 MHz, $CDCl_3$) δ 8.08 – 8.03 (m, 2H, Ar*H*), 7.57 – 7.52 (m, 2H, Ar*H*), 7.51 – 7.46 (m, 2H, Ar*H*), 7.36 – 7.30 (m, 3H, Ar*H*), 4.18 (t, *J* = 6.7 Hz, 1H, C*H*C≡C), 3.93 (s, 3H, C*H*$_3$), 3.65 (dd, *J* = 12.0, 7.3 Hz, 1H, C*H*$_2$N$_3$), 3.56 (dd, *J* = 12.0, 6.3 Hz, 1H, C*H*$_2$N$_3$). ^{13}C NMR (101 MHz, $CDCl_3$) δ 166.8, 143.4, 131.8, 130.2, 129.8, 128.6, 128.5, 128.1, 122.8, 87.1, 85.5, 57.1, 52.3, 39.7. HRMS (ESI/QTOF) m/z: $[M+Na]^+$ Calcd for $C_{18}H_{15}N_3NaO_2^+$ 328.1056; Found 328.1061.

1-(1-Azido-4-phenylbut-3-yn-2-yl)-4-(trifluoromethyl)benzene (3n):

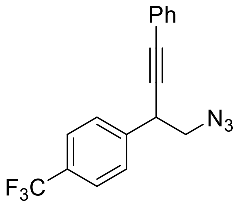

3n

Synthesized following **general procedure C** starting from 4-(trifluoromethyl)styrene (46 µL, 0.30 mmol, 1.00 equiv.). The reaction was carried out for 3 h, DCM was used for the silica plug. The crude product was purified by column chromatography (pentane/toluene, 90:10 to 85:15) to afford 1-(1-azido-4-phenylbut-3-yn-2-yl)-4-(trifluoromethyl)benzene (**3n**) (33 mg, 0.11 mmol, 35%) as a yellow oil.

R_f (pentane/toluene, 85:15): 0.38; ^{1}H NMR (400 MHz, $CDCl_3$) δ 7.65 (d, *J* = 8.3 Hz, 2H, Ar*H*), 7.59 (d, *J* = 8.4 Hz, 2H, Ar*H*), 7.50 – 7.46 (m, 2H, Ar*H*), 7.36 – 7.31 (m, 3H, Ar*H*), 4.18 (t, *J* = 6.7 Hz, 1H, C*H*C≡C), 3.66 (dd, *J* = 12.1, 7.1 Hz, 1H, C*H*$_2$N$_3$), 3.57 (dd, *J* = 12.1, 6.3 Hz, 1H, C*H*$_2$N$_3$). ^{13}C NMR (101 MHz, $CDCl_3$) δ 142.3, 131.9, 130.2 (q, *J* = 32.7 Hz), 128.7, 128.5, 128.5, 125.9 (q, *J* = 3.6 Hz), 124.2 (q, *J* = 272.1 Hz), 122.7, 86.9, 85.7, 57.1, 39.5. ^{19}F NMR (376 MHz, $CDCl_3$) δ -62.6. HRMS (APPI/LTQ-Orbitrap) m/z: $[M-N_2+H]^+$ Calcd for $C_{17}H_{13}F_3N^+$ 288.0995; Found 288.0995.

2-(1-Azido-4-phenylbut-3-yn-2-yl)thiophene (3o):

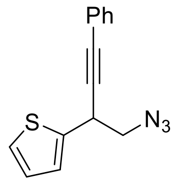

3o

Synthesized following **general procedure C** starting from a solution of 2-vinylthiophene (35 mg, 0.30 mmol, 1.00 equiv.) in dry degassed DME (0.5 mL). The reaction was carried out for 1.5 h, DCM was used for the silica plug. The crude product was purified by column chromatography (pentane/toluene, 90:10 to 85:15) to afford 2-(1-azido-4-phenylbut-3-yn-2-yl)thiophene (**3o**) (47 mg, 0.19 mmol, 62%) as a yellow oil.

R_f (pentane/toluene, 7:3): 0.55; ^{1}H NMR (400 MHz, $CDCl_3$) δ 7.53 – 7.47 (m, 2H, Ar*H*), 7.37 – 7.31 (m, 3H, Ar*H*), 7.28 (dd, *J* = 5.1, 1.2 Hz, 1H, Ar*H*), 7.16 – 7.11 (m, 1H, Ar*H*), 7.01 (dd, *J* = 5.1, 3.5 Hz, 1H, Ar*H*), 4.39 (t, *J* = 6.6 Hz, 1H, C*H*C≡C), 3.70 (dd, *J* = 12.0, 7.2 Hz, 1H, CH_2N_3), 3.61 (dd, *J* = 12.0, 6.2 Hz, 1H, CH_2N_3). ^{13}C NMR (101 MHz, $CDCl_3$) δ 141.3, 131.9, 128.6, 128.4, 127.1, 125.8, 125.2, 122.7, 87.4, 84.7, 57.4, 35.0. HRMS (APPI/LTQ-Orbitrap) m/z: $[M-N_2+H]^+$ Calcd for $C_{14}H_{12}NS^+$ 226.0685; Found 226.0687.

2-(1-Azido-4-phenylbut-3-yn-2-yl)benzofuran (3p):

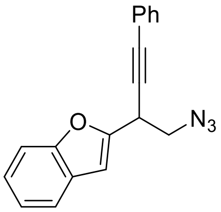

3p

Synthesized following **general procedure C** starting from a solution of 2-vinylbenzofuran (**1i**) (43 mg, 0.30 mmol, 1.00 equiv.) in dry degassed DME (0.5 mL). The reaction was carried out for 1.5 h, DCM was used for the silica plug. The crude product was purified by column chromatography (pentane/toluene, 85:15) to afford 2-(1-azido-4-phenylbut-3-yn-2-yl)benzofuran (**3p**) (54 mg, 0.19 mmol, 62%) as a yellow oil.

R_f (pentane/toluene, 8:2): 0.35; ^{1}H NMR (400 MHz, $CDCl_3$) δ 7.59 – 7.45 (m, 4H, Ar*H*), 7.37 – 7.32 (m, 3H, Ar*H*), 7.33 – 7.19 (m, 2H, Ar*H*), 6.84 (s, 1H, OC=C*H*), 4.37 (t, *J* = 6.0 Hz, 1H, C*H*C≡C), 3.83 (dd, *J* = 11.1, 4.7 Hz, 1H, CH_2N_3), 3.79 (dd, *J* = 12.1, 4.9 Hz, 1H, CH_2N_3). ^{13}C NMR (101 MHz, $CDCl_3$) δ 155.2, 153.9, 132.0, 128.7, 128.5, 128.3, 124.4, 123.1, 122.6, 121.1, 111.3, 105.0, 84.9, 84.8, 54.1, 34.2. HRMS (APPI/LTQ-Orbitrap) m/z: $[M-N_2+H]^+$ Calcd for $C_{18}H_{14}NO^+$ 260.1070; Found 260.1071.

2-(1-Azido-4-phenylbut-3-yn-2-yl)-5-bromofuran (3q):

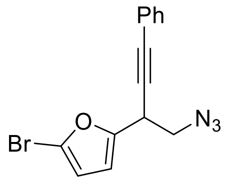

3q

Synthesized following **general procedure C** starting from a solution of 2-bromo-5-vinylfuran (**1j**) (52 mg, 0.30 mmol, 1.00 equiv.) in dry degassed DME (0.5 mL). The reaction was carried out for 1.5 h, DCM was used for the silica plug. The crude product was purified by column chromatography (pentane/toluene, 9:1) to afford 2-(1-azido-4-phenylbut-3-yn-2-yl)-5-bromofuran (**3q**) (52 mg, 0.16 mmol, 54%) as an orange oil.

R_f (pentane/toluene, 8:2): 0.43; ^{1}H NMR (400 MHz, $CDCl_3$) δ 7.50 – 7.45 (m, 2H, Ar*H*), 7.36 – 7.30 (m, 3H, Ar*H*), 6.40 (dd, *J* = 3.3, 0.9 Hz, 1H, Ar*H*), 6.30 (d, *J* = 3.3 Hz, 1H, Ar*H*), 4.20 (t, *J* = 6.0 Hz, 1H, C*H*C≡C), 3.68 (dd, *J* = 6.2, 2.5 Hz, 2H, CH_2N_3). ^{13}C NMR (101 MHz, $CDCl_3$) δ 152.8, 131.9, 128.7, 128.5, 122.5, 121.5, 112.4, 110.7, 84.7, 84.5, 54.2, 33.8. HRMS (APPI/LTQ-Orbitrap) m/z: $[M-N_2+H]^+$ Calcd for $C_{14}H_{11}BrNO^+$ 288.0019; Found 288.0025.

(3-(Azidomethyl)penta-1,4-diyne-1,5-diyl)dibenzene (3r):

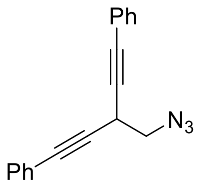

3r

Synthesized following **general procedure C** starting from a solution of but-3-en-1-yn-1-ylbenzene (**1b**) (39 mg, 0.30 mmol, 1.00 equiv.) in dry degassed DME (0.5 mL). The reaction was carried out for 1.5 h, DCM was used for the silica plug. The crude product was purified by column chromatography (pentane/toluene, 85:15) to afford (3-(azidomethyl)penta-1,4-diyne-1,5-diyl)dibenzene (**3r**) (36 mg, 0.13 mmol, 44%) as a yellow oil.

R_f (pentane/toluene, 8:2): 0.32; ^{1}H NMR (400 MHz, $CDCl_3$) δ 7.52 – 7.44 (m, 4H, Ar*H*), 7.37 – 7.29 (m, 6H, Ar*H*), 4.09 (t, *J* = 6.7 Hz, 1H, C*H*C≡C), 3.64 (d, *J* = 6.7 Hz, 2H, CH_2N_3). ^{13}C NMR (101 MHz, $CDCl_3$) δ 132.0, 128.7, 128.4, 122.6, 84.4, 83.2, 55.1, 26.8. HRMS (APPI/LTQ-Orbitrap) m/z: $[M-N_2+H]^+$ Calcd for $C_{18}H_{14}N^+$ 244.1121; Found 244.1121.

(4-Azidopent-1-yne-1,3-diyl)dibenzene (3s):

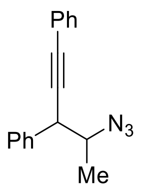

3s

Synthesized following **general procedure C** starting from *trans*-β-methylstyrene (**1d**) (36 μL, 0.30 mmol, 1.00 equiv.). The reaction was carried out for 1.5 h, DCM was used for the silica plug. Crude ^{1}H NMR of the mixture showed a diastereomeric ratio of 1.9:1 using peaks at 4.08 and 3.98 ppm. The crude product was purified by column chromatography (pentane/Et_2O, 99:1 to 98:2) to afford an inseparable mixture of diastereoisomers (4-azidopent-1-yne-1,3-diyl)dibenzene (**3s**) (45 mg, 0.17 mmol, 57%) as a yellow oil.

R_f (pentane/Et_2O, 97.5:2.5): 0.54; ^{1}H NMR (400 MHz, $CDCl_3$) δ 7.53 – 7.44 (m, 8H, Ar*H* major + minor), 7.42 – 7.36 (m, 4H, Ar*H* major + minor), 7.35 – 7.30 (m, 8H, Ar*H* major + minor), 4.08 (d, *J* = 6.1 Hz, 1H, C*H*C≡C major), 3.98 (d, *J* = 5.4 Hz, 1H, C*H*C≡C minor), 3.83 – 3.75 (m, 1H, C*H*N_3 minor), 3.71 (p, *J* = 6.5 Hz, 1H, C*H*N_3 major), 1.40 (d, *J* = 6.6 Hz, 3H, C*H₃* major), 1.38 (d, *J* = 6.7 Hz, 3H, CH_3 minor). ^{13}C NMR (101 MHz, $CDCl_3$) δ 138.1, 138.1, 131.8, 131.8, 128.8, 128.7, 128.6, 128.5, 128.4, 128.4, 128.3, 128.3, 127.8, 127.7, 123.2, 123.2, 87.6, 87.4, 85.7, 85.4, 62.1, 62.0, 45.4, 45.2, 17.7, 16.0. HRMS (APPI/LTQ-Orbitrap) m/z: $[M-N_2+H]^+$ Calcd for $C_{17}H_{16}N^+$ 234.1277; Found 234.1288.

2-Azido-1-(phenylethynyl)-2,3-dihydro-1*H*-indene (3t/3t'):

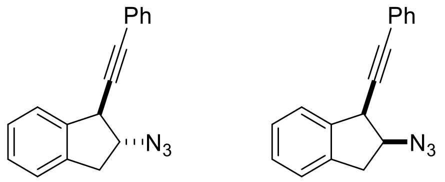

3t **3t'**

Synthesized following **general procedure C** starting from indene (35 μL, 0.30 mmol, 1.00 equiv.). The reaction was carried out for 1.5 h, DCM was used for the silica plug. Crude ^{1}H NMR of the mixture showed a diastereomeric ratio of 5.4:1 (*trans*:*cis*) using peaks at 4.46 and 4.38 ppm. The crude product was purified by column chromatography (pentane/toluene, 9:1 to 8:2).

trans diastereoisomer (major): 1,2-*trans*-2-azido-1-(phenylethynyl)-2,3-dihydro-1*H*-indene (**3t**) (49 mg, 0.19 mmol, 63%) yellow oil. The *trans* configuration was determined using ^{1}H-^{1}H NOESY experiment on the reduced product (see product **5** in section 8.3)

R_f (pentane/toluene, 7:3): 0.46; ^{1}H NMR (400 MHz, $CDCl_3$) δ 7.50 – 7.42 (m, 3H, Ar*H*), 7.35 – 7.21 (m, 6H, Ar*H*), 4.38 (q, *J* = 8.3 Hz, 1H, C*H*N_3), 4.22 (d, *J* = 8.1 Hz, 1H, C*H*C≡C), 3.32 (dd, *J* = 15.5, 7.4 Hz, 1H, ArCH_2), 2.95 (dd, *J* = 15.5, 8.6 Hz, 1H, ArCH_2). ^{13}C NMR (101 MHz, $CDCl_3$) δ 140.5, 138.7, 131.9, 128.4, 128.3, 128.1, 127.7, 124.8, 124.6, 123.1, 88.0, 83.9, 68.7, 43.7, 37.4. HRMS (APPI/LTQ-Orbitrap) m/z: $[M-N_2]^+$ Calcd for $C_{17}H_{13}N^+$ 231.1043; Found 231.1043.

cis diastereoisomer (minor): 1,2-*cis*-2-azido-1-(phenylethynyl)-2,3-dihydro-1*H*-indene (**3t'**), the yield was determined only by crude ^{1}H NMR (12%) using CH_2Br_2 (10.6 μL, 0.150 mmol, 0.50 equiv.) as internal standard. An analytically pure sample was obtained by preparative TLC (pentane/toluene, 1:1).

R_f (pentane/toluene, 7:3): 0.25; ^{1}H NMR (400 MHz, $CDCl_3$) δ 7.54 – 7.47 (m, 3H, Ar*H*), 7.34 – 7.30 (m, 3H, Ar*H*), 7.28 – 7.25 (m, 3H, Ar*H*), 4.49 – 4.43 (m, 2H, C*H*C≡C + C*H*N_3), 3.26 – 3.16 (m, 1H, ArC*H₂*), 3.13 – 3.06 (m, 1H, ArC*H₂*). ^{13}C NMR (101 MHz, $CDCl_3$) δ 140.5, 139.3, 132.0, 128.4, 128.3, 128.0, 127.7, 124.9, 124.7, 123.2, 85.6, 85.5, 65.4, 43.6, 38.1. HRMS (nanochip-ESI/LTQ-Orbitrap) m/z: $[M-N_2+H]^+$ Calcd for $C_{17}H_{14}N^+$ 232.1121; Found 232.1122.

2-Azido-1-(phenylethynyl)-1,2,3,4-tetrahydronaphthalene (3u/3u'):

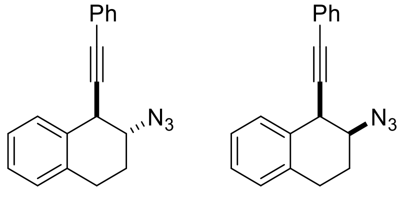

Synthesized following **general procedure C** starting from 1,2-dihydronaphthalene (39 µL, 0.30 mmol, 1.00 equiv.). The reaction was carried out for 1.5 h, DCM was used for the silica plug. Crude ^{1}H NMR of the mixture showed a diastereomeric ratio of 3.8:1 (*trans*:*cis*) using peaks at 2.08 and 1.89 ppm. The crude product was purified by column chromatography (pentane/toluene, 9:1 to 8:2). Relative configuration of the diastereoisomers were determined by analogy to **3t/3t'** where the major product is *trans*.

trans diastereoisomer (major): 1,2-*trans*-2-azido-1-(phenylethynyl)-1,2,3,4-tetrahydronaphthalene (**3u**) (50 mg, 0.18 mmol, 61%) yellow oil.

R_f (pentane/toluene, 8:2): 0.42; ^{1}H NMR (400 MHz, $CDCl_3$) δ 7.62 – 7.58 (m, 1H, Ar*H*), 7.50 – 7.44 (m, 2H, Ar*H*), 7.34 – 7.29 (m, 3H, Ar*H*), 7.25 – 7.19 (m, 2H, Ar*H*), 7.12 (d, *J* = 6.8 Hz, 1H, Ar*H*), 4.04 (d, *J* = 8.2 Hz, 1H, C*H*C≡C), 4.01 – 3.95 (m, 1H, C*H*N_3), 3.00 – 2.93 (m, 2H, ArC*H₂*), 2.36 – 2.27 (m, 1H, $ArCH_2CH_2$), 1.96 – 1.83 (m, 1H, $ArCH_2CH_2$). ^{13}C NMR (101 MHz, $CDCl_3$) δ 134.6, 133.7, 131.9, 129.3, 128.8, 128.4, 128.3, 127.3, 126.7, 123.2, 89.5, 83.8, 62.5, 39.2, 27.4, 27.2. HRMS (APPI/LTQ-Orbitrap) m/z: $[M-N_2+H]^+$ Calcd for $C_{18}H_{16}N^+$ 246.1277; Found 246.1269.

cis diastereoisomer (minor): 1,2-*cis*-2-azido-1-(phenylethynyl)-1,2,3,4-tetrahydronaphthalene (**3u'**) (8 mg, 0.03 mmol, 10%) orange oil.

R_f (pentane/toluene, 8:2): 0.32; ^{1}H NMR (400 MHz, $CDCl_3$) δ 7.51 – 7.43 (m, 3H, Ar*H*), 7.31 – 7.27 (m, 3H, Ar*H*), 7.24 – 7.18 (m, 2H, Ar*H*), 7.15 – 7.10 (m, 1H, Ar*H*), 4.27 (d, *J* = 4.3 Hz, 1H, C*H*C≡C), 4.01 (ddd, *J* = 8.5, 4.3, 2.8 Hz, 1H, C*H*N_3), 3.08 (dt, *J* = 17.2, 6.5 Hz, 1H, ArC*H₂*), 2.87 (dt, *J* = 17.2, 6.9 Hz, 1H, ArC*H₂*), 2.37 – 2.27 (m, 1H, $ArCH_2CH_2$), 2.13 – 2.04 (m, 1H, $ArCH_2CH_2$). ^{13}C NMR (101 MHz, $CDCl_3$) δ 134.5, 133.9, 131.9, 129.6, 129.0, 128.3, 128.2, 127.5, 126.6, 123.3, 88.2, 85.0, 59.6, 38.3, 26.5, 25.6. HRMS (APPI/LTQ-Orbitrap) m/z: $[M-N_2+H]^+$ Calcd for $C_{18}H_{16}N^+$ 246.1277; Found 246.1275.

3,4-*trans*-3-Azido-4-(phenylethynyl)chromane (3v):

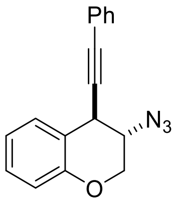

3v

Synthesized following **general procedure C** starting from a solution of 2*H*-chromene (**1k**) (40 mg, 0.30 mmol, 1.00 equiv.) in dry degassed DME (0.5 mL). The reaction was carried out for 1.5 h, DCM was used for the silica plug. The crude product was purified by column chromatography (pentane/toluene, 85:15 to 80:20) to afford 3,4-*trans*-3-azido-4-(phenylethynyl)chromane (**3v**) (24 mg, 0.090 mmol, 30%) as a yellow oil. Relative configuration of the diastereoisomer was determined by analogy to **3t/3t'** where the major product is *trans*.

R_f (Pentane/Toluene, 75:25): 0.23; ^{1}H NMR (400 MHz, $CDCl_3$) δ 7.49 – 7.42 (m, 3H, Ar*H*), 7.34 – 7.29 (m, 3H, Ar*H*), 7.25 – 7.18 (m, 1H, Ar*H*), 6.99 (td, *J* = 7.5, 1.2 Hz, 1H, Ar*H*), 6.88 (dd, *J* = 8.2, 1.1 Hz, 1H, Ar*H*), 4.43 (dd, *J* = 10.9, 2.0 Hz, 1H, OCH_2CH), 4.13 – 4.01 (m, 3H, OCH_2CH + C*H*N_3 + C*H*C≡C). ^{13}C NMR (101 MHz, $CDCl_3$) δ 152.8, 131.9, 130.0, 129.1, 128.6, 128.4, 122.7, 121.7, 119.3, 117.0, 87.9, 84.4, 65.9, 58.3, 35.2. HRMS (APPI/LTQ-Orbitrap) m/z: [M-N_2+H]$^+$ Calcd for $C_{17}H_{14}NO^+$ 248.1070; Found 248.1080.

(4-Azido-3-butoxybut-1-yn-1-yl)benzene (3w):

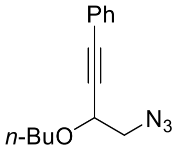

3w

Synthesized following **general procedure C** starting from vinyl butyl ether (39 µL, 0.30 mmol, 1.00 equiv.). The reaction was carried out for 1.5 h, DCM was used for the silica plug. The crude product was purified by column chromatography (pentane/Et_2O, 99:1 to 98:2) to afford (4-azido-3-butoxybut-1-yn-1-yl)benzene (**3w**) (36 mg, 0.15 mmol, 49%) as a slightly yellow oil.

R_f (pentane/Et_2O, 95:5): 0.55; ^{1}H NMR (400 MHz, $CDCl_3$) δ 7.47 – 7.43 (m, 2H, Ar*H*), 7.35 – 7.30 (m, 3H, Ar*H*), 4.43 (dd, *J* = 7.6, 4.0 Hz, 1H, C*H*C≡C), 3.86 (dt, *J* = 9.1, 6.5 Hz, 1H, CH_2O), 3.61 – 3.47 (m, 2H, CH_2O + CH_2N_3), 3.38 (dd, *J* = 12.8, 4.0 Hz, 1H, CH_2N_3), 1.68 – 1.60 (m, 2H, CH_2CH_2O), 1.49 – 1.39 (m, 2H, CH_2CH_3), 0.95 (t, *J* = 7.4 Hz, 3H, CH_2CH_3). ^{13}C NMR (101 MHz, $CDCl_3$) δ 131.9, 128.9, 128.5, 122.3, 87.1, 85.3, 69.9, 69.5, 54.7, 31.8, 19.4, 14.0. HRMS (ESI/QTOF) m/z: [M+Na]$^+$ Calcd for $C_{14}H_{17}N_3NaO^+$ 266.1264; Found 266.1260.

7.1 Scope of Alkynes

General procedure D:

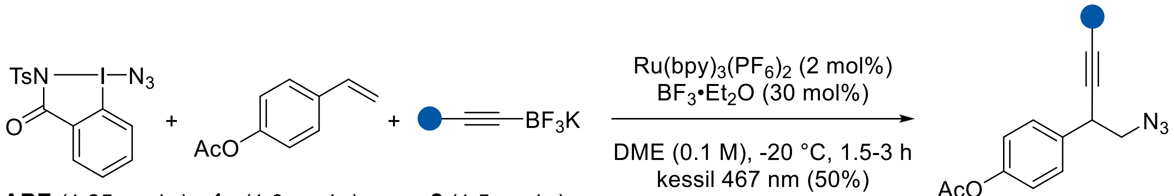

Ts-ABZ (1.25 equiv.) **1e** (1.0 equiv.) **2** (1.5 equiv.) **3**

An oven-dried test tube charged with $Ru(bpy)_3(PF_6)_2$ (5.2 mg, 6.0 µmol, 0.02 equiv.), **Ts-ABZ** (166 mg, 0.375 mmol, 1.25 equiv.) and potassium trifluoroborate **2** (0.45 mmol, 1.50 equiv.) was evacuated and backfilled with N_2 (3x). Dry degassed DME (2.7 mL) and 4-acetoxystyrene (**1e**) (46 µL, 0.30 mmol, 1.00 equiv.) were added and the mixture was cooled to -20 °C. Then, a stock solution of BF_3•Et_2O (11 µL, 90 µmol, 0.30 equiv.) in dry degassed DME (0.34 mL) was added and the reaction was stirred under blue LEDs irradiation (1 x Kessil 467 nm 50% intensity, 22 W) at -20 °C for 1.5-3 h. The reaction mixture was filtered through a short plug of silica and eluted with DCM then concentrated *in vacuo*. The crude product was purified by column chromatography to afford **3**.

Note: 4-Acetoxystyrene (**1e**) was eluted through a short plug of basic Al_2O_3 before use. DME was sparged with argon for 0.5 h before use. Cooling was performed using a Dewar filled with a mixture of ice and salt. We did not observe significant rise in temperature after 1.5 h (which is enough in most cases to reach full conversion). In the case of longer reaction, the cold bath was replaced with a new one after 1.5 h. For further details on the photochemistry set-up see Figure S1.

4-(1-Azido-4-(3-methoxyphenyl)but-3-yn-2-yl)phenyl acetate (3x):

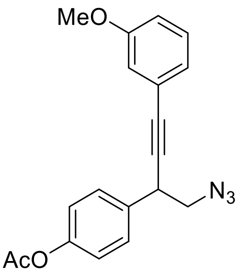

3x

Synthesized following **general procedure D** starting from potassium trifluoro((3-methoxyphenyl)ethynyl)borate (**2c**) (107 mg, 0.450 mmol, 1.50 equiv.). The reaction was carried out for 1.5 h, DCM was used for the silica plug. The crude product was purified by column chromatography (pentane/Et_2O, 85:15) to afford 4-(1-azido-4-(3-methoxyphenyl)but-3-yn-2-yl)phenyl acetate (**3x**) (60 mg, 0.18 mmol, 60%) as a yellow oil.

R_f (pentane/Et_2O, 8:2): 0.19; ^{1}H NMR (400 MHz, $CDCl_3$) δ 7.50 – 7.45 (m, 2H, Ar*H*), 7.27 – 7.20 (m, 1H, Ar*H*), 7.14 – 7.05 (m, 3H, Ar*H*), 7.01 (dd, *J* = 2.4, 1.4 Hz, 1H, Ar*H*), 6.89 (ddd, *J* = 8.3, 2.6, 0.8 Hz, 1H, Ar*H*), 4.16 – 4.09 (m, 1H, C*H*C≡C), 3.81 (s, 3H, OC*H*$_3$), 3.62 (dd, *J* = 12.0, 7.7 Hz, 1H, C*H*$_2$N$_3$), 3.52 (dd, *J* = 12.0, 6.1 Hz, 1H, C*H*$_2$N$_3$), 2.31 (s, 3H, C*H*$_3$). ^{13}C NMR (101 MHz, $CDCl_3$) δ 169.6, 159.4, 150.3, 135.7, 129.5, 129.1, 124.3, 123.9, 122.1, 116.6,

115.2, 87.6, 85.1, 57.3, 55.4, 39.2, 21.3. HRMS (ESI/QTOF) m/z: $[M+Na]^+$ Calcd for $C_{19}H_{17}N_3NaO_3^+$ 358.1162; Found 358.1161.

4-(1-Azido-4-(4-fluorophenyl)but-3-yn-2-yl)phenyl acetate (3y):

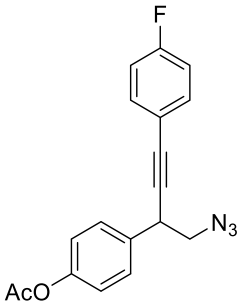

3y

Synthesized following **general procedure D** starting from potassium trifluoro((4-fluorophenyl)ethynyl)borate (**2d**) (102 mg, 0.450 mmol, 1.50 equiv.). The reaction was carried out for 1.5 h, DCM was used for the silica plug. The crude product was purified by column chromatography (pentane/Et_2O, 90:10 to 85:15) to afford 4-(1-azido-4-(4-fluorophenyl)but-3-yn-2-yl)phenyl acetate (**3y**) (65 mg, 0.20 mmol, 67%) as a yellow oil.

R_f (pentane/Et_2O, 8:2): 0.3; ^1H NMR (400 MHz, $CDCl_3$) δ 7.45 (dd, *J* = 8.6, 4.7 Hz, 4H, Ar*H*), 7.14 – 7.08 (m, 2H, Ar*H*), 7.06 – 6.97 (m, 2H, Ar*H*), 4.14 – 4.08 (m, 1H, C*H*C≡C), 3.60 (dd, *J* = 12.0, 7.7 Hz, 1H, CH_2N_3), 3.51 (dd, *J* = 12.0, 6.0 Hz, 1H, CH_2N_3), 2.31 (s, 3H, CH_3). ^{13}C NMR (101 MHz, $CDCl_3$) δ 169.5, 162.7 (d, *J* = 249.5 Hz), 150.3, 135.7, 133.7 (d, *J* = 8.4 Hz), 129.0, 122.1, 119.0 (d, *J* = 3.5 Hz), 115.7 (d, *J* = 22.1 Hz), 87.5 (d, *J* = 1.3 Hz), 84.2, 57.3, 39.2, 21.3. ^{19}F NMR (376 MHz, $CDCl_3$) δ -110.9. HRMS (ESI/QTOF) m/z: $[M+Na]^+$ Calcd for $C_{18}H_{14}FN_3NaO_2^+$ 346.0962; Found 346.0953.

4-(1-Azido-4-(2-chlorophenyl)but-3-yn-2-yl)phenyl acetate (3z):

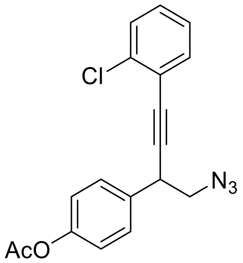

3z

Synthesized following **general procedure D** starting from potassium ((2-chlorophenyl)ethynyl)trifluoroborate (**2e**) (109 mg, 0.450 mmol, 1.50 equiv.). The reaction was carried out for 1.5 h, DCM was used for the silica plug. The crude product was purified by column chromatography (pentane/Et_2O, 90:10 to 85:15) to afford 4-(1-azido-4-(2-chlorophenyl)but-3-yn-2-yl)phenyl acetate (**3z**) (79 mg, 0.23 mmol, 77%) as a yellow oil.

R_f (pentane/Et_2O, 8:2): 0.3; ^1H NMR (400 MHz, $CDCl_3$) δ 7.56 – 7.47 (m, 3H, Ar*H*), 7.41 (dd, *J* = 7.9, 1.4 Hz, 1H, Ar*H*), 7.30 – 7.17 (m, 2H, Ar*H*), 7.15 – 7.07 (m, 2H, Ar*H*), 4.16 (t, *J* = 6.8 Hz, 1H, C*H*C≡C), 3.66 (dd, *J* = 12.0, 7.6 Hz, 1H, CH_2N_3), 3.56 (dd, *J* = 12.0, 6.3 Hz, 1H, CH_2N_3), 2.30 (s, 3H, CH_3). ^{13}C NMR (101 MHz, $CDCl_3$) δ 169.5, 150.3, 136.3, 135.4, 133.6, 129.5, 129.4, 129.2, 126.6, 122.9, 122.1, 93.2, 82.1, 57.4, 39.3, 21.3. HRMS (ESI/QTOF) m/z: $[M+Na]^+$ Calcd for $C_{18}H_{14}ClN_3NaO_2^+$ 362.0667; Found 362.0654.

Methyl 4-(3-(4-acetoxyphenyl)-4-azidobut-1-yn-1-yl)benzoate (3aa):

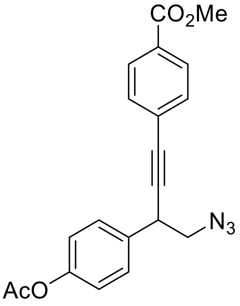

3aa

Synthesized following **general procedure D** starting from potassium trifluoro((4-(methoxycarbonyl)phenyl)ethynyl)borate (**2f**) (120 mg, 0.450 mmol, 1.50 equiv.). The reaction was carried out for 1.5 h, DCM was used for the silica plug. The crude product was purified by column chromatography (pentane/EtOAc, 85:15 to 80:20) to afford methyl 4-(3-(4-acetoxyphenyl)-4-azidobut-1-yn-1-yl)benzoate (**3aa**) (74 mg, 0.20 mmol, 68%) as a yellow oil.

R_f (pentane/EtOAc, 8:2): 0.33; ^1H NMR (400 MHz, $CDCl_3$) δ 8.02 – 7.96 (m, 2H, Ar*H*), 7.55 – 7.51 (m, 2H, Ar*H*), 7.49 – 7.42 (m, 2H, Ar*H*), 7.15 – 7.08 (m, 2H, Ar*H*), 4.17 – 4.11 (m, 1H, C*H*C≡C), 3.92 (s, 3H, OCH_3), 3.63 (dd, *J* = 12.0, 7.7 Hz, 1H, CH_2N_3), 3.53 (dd, *J* = 12.0, 6.1 Hz, 1H, CH_2N_3), 2.31 (s, 3H, CH_3). ^{13}C NMR (101 MHz, $CDCl_3$) δ 169.6, 166.7, 150.4, 135.4, 131.8, 129.8, 129.6, 129.0, 127.6, 122.2, 90.9, 84.5, 57.2, 52.4, 39.3, 21.3. HRMS (ESI/QTOF) m/z: $[M+Na]^+$ Calcd for $C_{20}H_{17}N_3NaO_4^+$ 386.1111; Found 386.1101.

4-(1-Azido-4-mesitylbut-3-yn-2-yl)phenyl acetate (3ab):

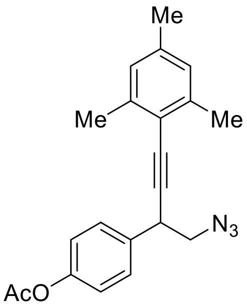

3ab

Synthesized following **general procedure D** starting from potassium trifluoro(mesitylethynyl)borate (**2g**) (113 mg, 0.450 mmol, 1.50 equiv.). The reaction was carried out for 3 h, DCM was used for the silica plug. The crude product was purified by column

chromatography (pentane/Et_2O, 95:5 to 90:10) to afford 4-(1-azido-4-mesitylbut-3-yn-2-yl)phenyl acetate (**3ab**) (35 mg, 99 μmol, 33%) as a yellow oil.

R_f (pentane/Et_2O, 9:1): 0.26; ^{1}H NMR (400 MHz, $CDCl_3$) δ 7.53 – 7.48 (m, 2H, Ar*H*), 7.14 – 7.08 (m, 2H, Ar*H*), 6.87 (s, 2H, Ar*H*), 4.20 (t, *J* = 6.9 Hz, 1H, C*H*C≡C), 3.64 (dd, *J* = 12.0, 7.4 Hz, 1H, CH_2N_3), 3.58 (dd, *J* = 12.0, 6.4 Hz, 1H, CH_2N_3), 2.42 (s, 6H, ArCH_3), 2.31 (s, 3H, CH_3C(O)), 2.28 (s, 3H, ArCH_3). ^{13}C NMR (101 MHz, $CDCl_3$) δ 169.5, 150.2, 140.4, 137.8, 136.2, 129.1, 127.7, 122.0, 119.7, 95.2, 83.1, 57.9, 39.3, 21.4, 21.3, 21.2. HRMS (ESI/QTOF) m/z: $[M+Na]^+$ Calcd for $C_{21}H_{21}N_3NaO_2^+$ 370.1526; Found 370.1536.

4-(1-Azido-4-(thiophen-3-yl)but-3-yn-2-yl)phenyl acetate (3ac):

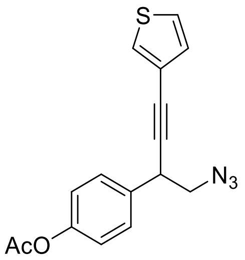

3ac

Synthesized following **general procedure D** starting from potassium trifluoro(thiophen-3-ylethynyl)borate (**2h**) (97 mg, 0.45 mmol, 1.50 equiv.). The reaction was carried out for 3 h, DCM was used for the silica plug. The crude product was purified by column chromatography (pentane/Et_2O, 85:15) to afford 4-(1-azido-4-(thiophen-3-yl)but-3-yn-2-yl)phenyl acetate (**3ac**) (50 mg, 0.16 mmol, 54%) as a yellow oil.

R_f (pentane/Et_2O, 8:2): 0.29; ^{1}H NMR (400 MHz, $CDCl_3$) δ 7.50 – 7.44 (m, 3H, Ar*H*), 7.28 (dd, *J* = 5.0, 3.0 Hz, 1H, Ar*H*), 7.17 – 7.09 (m, 3H, Ar*H*), 4.14 – 4.08 (m, 1H, C*H*C≡C), 3.61 (dd, *J* = 12.0, 7.7 Hz, 1H, CH_2N_3), 3.51 (dd, *J* = 12.0, 6.1 Hz, 1H, CH_2N_3), 2.31 (s, 3H, CH_3). ^{13}C NMR (101 MHz, $CDCl_3$) δ 169.5, 150.3, 135.7, 130.0, 129.0, 129.0, 125.4, 122.0, 121.9, 87.3, 80.4, 57.2, 39.2, 21.3. HRMS (ESI/QTOF) m/z: $[M+Na]^+$ Calcd for $C_{16}H_{13}N_3NaO_2S^+$ 334.0621; Found 334.0624.

4-(1-Azido-4-(benzofuran-2-yl)but-3-yn-2-yl)phenyl acetate (3ad):

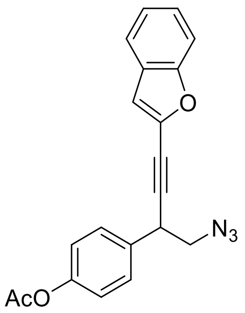

3ad

Synthesized following **general procedure D** starting from potassium (benzofuran-2-ylethynyl)trifluoroborate (**2i**) (112 mg, 0.450 mmol, 1.50 equiv.). The reaction was carried out for 3 h, DCM was used for the silica plug. The crude product was purified by column chromatography (pentane/Et_2O, 8:2) to afford 4-(1-azido-4-(benzofuran-2-yl)but-3-yn-2-yl)phenyl acetate (**3ad**) (49 mg, 0.14 mmol, 47%) as a yellow oil.

R_f (pentane/Et_2O, 6:4): 0.45; ^{1}H NMR (400 MHz, $CDCl_3$) δ 7.55 (d, *J* = 7.8 Hz, 1H, Ar*H*), 7.46 (t, *J* = 8.3 Hz, 3H, Ar*H*), 7.33 (t, *J* = 7.2 Hz, 1H, Ar*H*), 7.24 (t, *J* = 7.5 Hz, 1H, Ar*H*), 7.12 (d, *J* = 8.6 Hz, 2H, Ar*H*), 6.95 (s, 1H, OC=C*H*), 4.18 (t, *J* = 6.9 Hz, 1H, C*H*C≡C), 3.68 (dd, *J* = 12.0, 7.5 Hz, 1H, CH_2N_3), 3.58 (dd, *J* = 12.0, 6.3 Hz, 1H, CH_2N_3), 2.31 (s, 3H, CH_3). ^{13}C NMR (101 MHz, $CDCl_3$) δ 169.5, 154.8, 150.5, 138.3, 134.7, 129.1, 127.6, 125.7, 123.4, 122.2, 121.4, 111.8, 111.4, 94.1, 75.7, 56.8, 39.2, 21.3. HRMS (ESI/QTOF) m/z: $[M+Na]^+$ Calcd for $C_{20}H_{15}N_3NaO_3^+$ 368.1006; Found 368.1022.

4-(1-Azidopent-3-yn-2-yl)phenyl acetate (3ae)

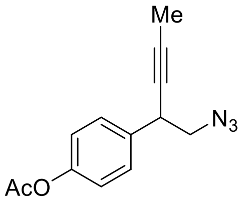

3ae

Synthesized following **general procedure D** starting from potassium trifluoro(prop-1-yn-1-yl)borate (**2j**) (66 mg, 0.45 mmol, 1.50 equiv.). The reaction was carried out for 3 h, DCM was used for the silica plug. The crude product was purified by column chromatography (pentane/Et_2O, 8:2) to afford 4-(1-azidopent-3-yn-2-yl)phenyl acetate (**3ae**) (40 mg, 0.16 mmol, 54%) as a slightly yellow oil.

R_f (pentane/Et_2O, 8:2): 0.46; ^{1}H NMR (400 MHz, $CDCl_3$) δ 7.41 – 7.36 (m, 2H, Ar*H*), 7.10 – 7.04 (m, 2H, Ar*H*), 3.87 – 3.80 (m, 1H, C*H*C≡C), 3.47 (dd, *J* = 11.9, 7.6 Hz, 1H, CH_2N_3), 3.39 (dd, *J* = 11.9, 6.2 Hz, 1H, CH_2N_3), 2.29 (s, 3H, CH_3), 1.88 (d, *J* = 2.4 Hz, 3H, C≡CCH_3). ^{13}C NMR (101 MHz, $CDCl_3$) δ[24] 169.5, 150.1, 136.3, 128.9, 121.9, 81.1, 57.5, 38.5, 21.2, 3.7. HRMS (ESI/QTOF) m/z: $[M+Na]^+$ Calcd for $C_{13}H_{13}N_3NaO_2^+$ 266.0900; Found 266.0902.

4-(1-Azido-4-cyclopropylbut-3-yn-2-yl)phenyl acetate (3af):

[24] One carbon of the alkyne is overlapping with the $CDCl_3$ signal.

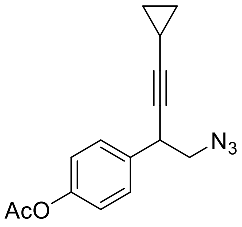

3af

Synthesized following **general procedure D** starting from potassium (cyclopropylethynyl)trifluoroborate (**2k**) (77 mg, 0.45 mmol, 1.50 equiv.). The reaction was carried out for 1.5 h, DCM was used for the silica plug. The crude product was purified by column chromatography (pentane/Et_2O, 9:1) to afford 4-(1-azido-4-cyclopropylbut-3-yn-2-yl)phenyl acetate (**3af**) (42 mg, 0.16 mmol, 52%) as a colorless oil.

R_f (pentane/Et_2O, 8:2): 0.29; ^1H NMR (400 MHz, $CDCl_3$) δ 7.40 – 7.34 (m, 2H, Ar*H*), 7.09 – 7.04 (m, 2H, Ar*H*), 3.87 – 3.80 (m, 1H, C*H*C≡C), 3.45 (dd, *J* = 11.9, 7.6 Hz, 1H, CH_2N_3), 3.36 (dd, *J* = 12.0, 6.1 Hz, 1H, CH_2N_3), 2.29 (s, 3H, CH_3), 1.34 – 1.25 (m, 1H, C*H*CH_2), 0.81 – 0.68 (m, 4H, CHCH_2). ^{13}C NMR (101 MHz, $CDCl_3$) δ 169.5, 150.1, 136.4, 128.9, 121.8, 88.6, 73.6, 57.5, 38.6, 21.2, 8.1, -0.3. HRMS (ESI/QTOF) m/z: $[M+Na]^+$ Calcd for $C_{15}H_{15}N_3NaO_2^+$ 292.1056; Found 292.1055.

4-(1-Azido-7-chlorohept-3-yn-2-yl)phenyl acetate (3ag):

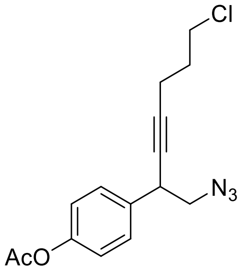

3ag

Synthesized following **general procedure D** starting from potassium (5-chloropent-1-yn-1-yl)trifluoroborate (**2l**) (94 mg, 0.45 mmol, 1.50 equiv.). The reaction was carried out for 1.5 h, DCM was used for the silica plug. The crude product was purified by column chromatography (pentane/Et_2O, 90:10 to 85:15) to afford 4-(1-azido-7-chlorohept-3-yn-2-yl)phenyl acetate (**3ag**) (47 mg, 0.16 mmol, 52%) as a yellow oil.

R_f (pentane/Et_2O, 8:2): 0.2; ^1H NMR (400 MHz, $CDCl_3$) δ 7.41 – 7.35 (m, 2H, Ar*H*), 7.10 – 7.05 (m, 2H, Ar*H*), 3.90 – 3.83 (m, 1H, C*H*C≡C), 3.66 (t, *J* = 6.3 Hz, 2H, CH_2Cl), 3.47 (dd, *J* = 12.0, 7.7 Hz, 1H, CH_2N_3), 3.40 (dd, *J* = 12.0, 6.1 Hz, 1H, CH_2N_3), 2.46 (td, *J* = 6.9, 2.2 Hz, 2H, C≡CCH_2CH_2), 2.30 (s, 3H, CH_3), 2.00 (p, *J* = 6.6 Hz, 2H, C≡CCH_2CH_2). ^{13}C NMR (101 MHz, $CDCl_3$) δ 169.5, 150.1, 136.1, 128.9, 121.9, 83.6, 79.5, 57.5, 43.8, 38.5, 31.4, 21.2, 16.4. HRMS (ESI/QTOF) m/z: $[M+Na]^+$ Calcd for $C_{15}H_{16}ClN_3NaO_2^+$ 328.0823; Found 328.0830.

4-(1-Azidobut-3-yn-2-yl)phenyl acetate (3ah):

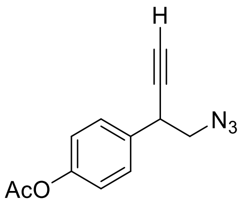

3ah

Synthesized following **general procedure D** starting from potassium ethynyltrifluoroborate (**2b**) (59 mg, 0.45 mmol, 1.50 equiv.). The reaction was carried out for 1.5 h, DCM was used for the silica plug. The crude product was purified by column chromatography (pentane/Et_2O, 90:10 to 85:15) to afford 4-(1-azidobut-3-yn-2-yl)phenyl acetate (**3ah**) (23 mg, 0.10 mmol, 34%) as a yellow oil.

R_f (pentane/Et_2O, 8:2): 0.35; ^{1}H NMR (400 MHz, $CDCl_3$) δ 7.44 – 7.39 (m, 2H, Ar*H*), 7.12 – 7.07 (m, 2H, Ar*H*), 3.89 (td, *J* = 7.3, 2.5 Hz, 1H, C*H*C≡C), 3.56 (dd, *J* = 12.0, 7.5 Hz, 1H, CH_2N_3), 3.48 (dd, *J* = 12.0, 6.3 Hz, 1H, CH_2N_3), 2.39 (d, *J* = 2.5 Hz, 1H, C≡C*H*), 2.30 (s, 3H, CH_3). ^{13}C NMR (101 MHz, $CDCl_3$) δ 169.5, 150.4, 135.1, 129.0, 122.1, 82.5, 73.3, 57.1, 38.1, 21.3. HRMS (ESI/QTOF) m/z: $[M+Na]^+$ Calcd for $C_{12}H_{11}N_3NaO_2^+$ 252.0743; Found 252.0734.

8. Product Modifications

8.1 Scale-up

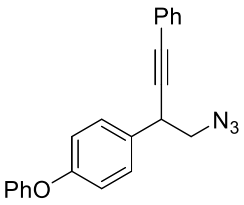

3ai

An oven-dried round-bottom flask charged with $Ru(bpy)_3(PF_6)_2$ (17.2 mg, 20.0 μmol, 2 mol%), **Ts-ABZ** (553 mg, 1.25 mmol, 1.25 equiv.), potassium trifluoro(phenylethynyl)borate (**2a**) (312 mg, 1.50 mmol, 1.50 equiv.) was evacuated and backfilled with N_2 (3x). Dry degassed DME (7.8 mL) and a solution of 1-phenoxy-4-vinylbenzene (**1c**) (207 mg, 1.00 mmol, 1.00 equiv.) in dry degassed DME (1.0 mL) were added and the mixture was cooled to -20 °C. Then, a stock solution of BF_3•Et_2O (37 μL, 0.30 mmol, 0.30 equiv.) in dry degassed DME (1.2 mL) was added and the reaction was stirred under blue LEDs irradiation (1 x Kessil 467 nm 50% intensity, 22W) at -20 °C for 1.5 h. The reaction mixture was filtered through a short plug of silica and eluted with DCM then concentrated *in vacuo*. The crude product was purified by column chromatography (pentane/Et_2O, 97.5:2.5) to afford 1-(1-azido-4-phenylbut-3-yn-2-yl)-4-phenoxybenzene (**3ai**) (271 mg, 0.799 mmol, 80%) as a yellow oil.

R_f (pentane/Et_2O, 9:1): 0.52; ^1H NMR (400 MHz, $CDCl_3$) δ 7.51 – 7.46 (m, 2H, Ar*H*), 7.45 – 7.40 (m, 2H, Ar*H*), 7.38 – 7.30 (m, 5H, Ar*H*), 7.14 – 7.09 (m, 1H, Ar*H*), 7.05 – 6.99 (m, 4H, Ar*H*), 4.11 (t, J = 6.8 Hz, 1H, C*H*C≡C), 3.62 (dd, J = 12.0, 7.4 Hz, 1H, C$H_2$$N_3$), 3.53 (dd, J = 12.0, 6.3 Hz, 1H, C$H_2$$N_3$). ^{13}C NMR (101 MHz, CDCl3) δ[25] 157.2, 157.0, 133.0, 131.8, 129.9, 129.3, 128.4, 123.6, 123.0, 119.2, 119.1, 88.0, 85.1, 57.5, 39.1. HRMS (Sicrit plasma/LTQ-Orbitrap) m/z: $[M-N_2+H]^+$ Calcd for $C_{22}H_{18}NO^+$ 312.1383; Found 312.1384.

8.2 Azide reduction

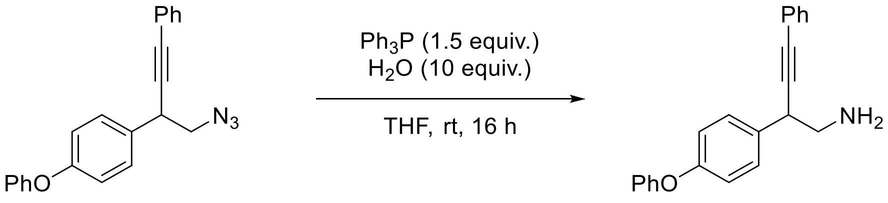

3ai **4**

To a microwave vial containing a solution of 1-(1-azido-4-phenylbut-3-yn-2-yl)-4-phenoxybenzene (**3ai**) (34 mg, 0.10 mmol, 1.00 equiv) in THF (1 mL) were added Ph_3P (39 mg, 0.15 mmol, 1.50 equiv) and water (18 μL, 1.0 mmol, 10.0 equiv) under air. The vial was

[25] One aromatic carbon was not resolved.

capped and the reaction was stirred at rt for 16 h. The mixture was concentrated *in vacuo* then was loaded on a small plug of silica using DCM. The plug was washed with DCM then the crude product was collected using DCM/MeOH (9:1) and concentrated *in vacuo*. The crude product was purified by reverse phase column chromatography (H_2O/CH_3CN, 95:5 to 5:95) to afford 2-(4-phenoxyphenyl)-4-phenylbut-3-yn-1-amine (**4**) (29 mg, 91 μmol, 91%) as an orange oil.

^{1}H NMR (400 MHz, $CDCl_3$) δ 7.50 – 7.44 (m, 2H, Ar*H*), 7.42 – 7.37 (m, 2H, Ar*H*), 7.37 – 7.29 (m, 5H, Ar*H*), 7.14 – 7.08 (m, 1H, Ar*H*), 7.05 – 6.98 (m, 4H, Ar*H*), 3.96 – 3.90 (m, 1H, C*H*C≡C), 3.08 (dd, *J* = 12.8, 5.6 Hz, 1H, CH_2NH_2), 3.01 (dd, *J* = 12.8, 7.1 Hz, 1H, CH_2NH_2), 1.44 (bs, 2H, NH_2). ^{13}C NMR (101 MHz, $CDCl_3$) δ 157.3, 156.5, 134.3, 131.9, 129.9, 129.2, 128.4, 128.2, 123.4, 123.4, 119.1, 119.0, 89.6, 84.7, 49.8, 42.5. HRMS (ESI/QTOF) m/z: $[M+H]^+$ Calcd for $C_{22}H_{20}NO^+$ 314.1539; Found 314.1534.

8.3 Hydrogenation

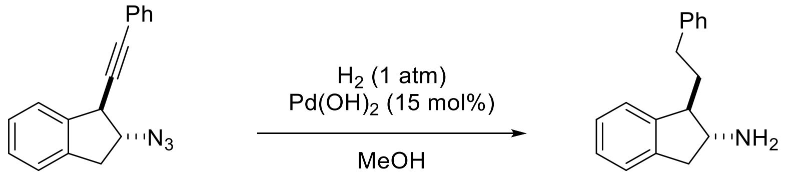

3t **5**

A capped oven-dried microwave vial charged with 2-azido-1-(phenylethynyl)-2,3-dihydro-1*H*-indene (**3t**) (31 mg, 0.12 mmol, 1.00 equiv.) was evacuated and backfilled with N_2 (3x). Then, dry MeOH (2 mL) was added. The vial was opened and $Pd(OH)_2$ (13 mg, 18 μmol, 20 wt.% on C, 0.15 equiv.) was added. The vial was capped and the mixture was sparged with H_2 for 15 min using a balloon. The reaction was stirred at rt for 16 h under H_2 atmosphere (1 atm, balloon). The mixture was sparged with N_2 for 15 min then was filtered over a syringe filter (PTFE, 0.22 μm), eluted with DCM and concentrated in vacuo to afford 1-phenethyl-2,3-dihydro-1*H*-inden-2-amine (**5**) (26 mg, 0.11 mmol, 93%) as a dark orange oil.

^{1}H NMR (400 MHz, $CDCl_3$) δ 7.32 – 7.27 (m, 2H, Ar*H*), 7.25 – 7.15 (m, 7H, Ar*H*), 3.53 (dt, *J* = 6.7, 4.8 Hz, 1H, C*H*NH_2), 3.27 (dd, *J* = 15.9, 6.8 Hz, 1H, $ArCH_2CHNH_2$), 2.88 (q, *J* = 6.4 Hz, 1H, ArC*H*), 2.82 – 2.75 (m, 2H, $ArCH_2CH_2$), 2.66 (dd, *J* = 15.9, 4.9 Hz, 1H, $ArCH_2CHNH_2$), 2.05 – 1.81 (m, 4H, $ArCH_2CH_2$ + NH_2). ^{13}C NMR (101 MHz, $CDCl_3$) δ 145.3, 142.4, 141.2, 128.6, 128.5, 126.9, 126.7, 126.0, 125.1, 124.6, 58.6, 54.4, 41.7, 35.1, 33.8. HRMS (Nanochip-based ESI/LTQ-Orbitrap) m/z: $[M+H]^+$ Calcd for $C_{17}H_{20}N^+$ 238.1590; Found 238.1590.

8.4 Pyrrole formation

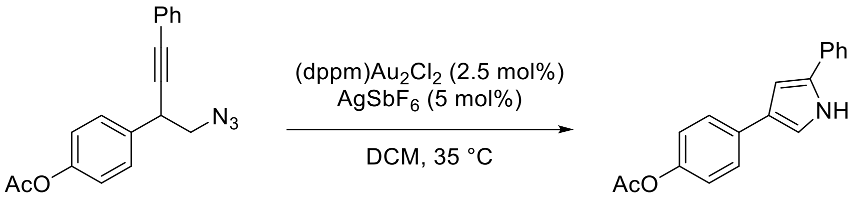

3g **6**

Adapting from a known methodology,[26] a capped oven-dried microwave vial charged with $AgSbF_6$ (1.7 mg, 5.0 µmol, 5 mol%) and (dppm)Au_2Cl_2 (2.1 mg, 2.5 µmol, 2.5 mol%) was evacuated and backfilled with N_2 (3x). Then, dry DCM (1 mL) was added and the mixture was stirred at rt for 2 min. A solution of 4-(1-azido-4-phenylbut-3-yn-2-yl)phenyl acetate (**3g**) (31 mg, 0.10 mmol, 1.00 equiv.) in dry DCM (1 mL) was added under N_2 and the vial was placed in a preheated heating block at 35 °C. The reaction was stirred at 35 °C for 30 min. The mixture was allowed to cool to rt, filtered over activated neutral alumina, eluted with DCM and concentrated *in vacuo*. The crude product was purified by column chromatography on activated neutral alumina (pentane/EtOAc, 95:5 to 80:20) to afford 4-(5-phenyl-1H-pyrrol-3-yl)phenyl acetate (**6**) (16 mg, 58 µmol, 58%) as a slightly yellow solid.

R_f (SiO_2, pentane/EtOAc, 7 :3): 0.43; Mp (Dec.): 176 °C; ^{1}H NMR (400 MHz, $CDCl_3$) δ 8.47 (s, 1H, N*H*), 7.58 – 7.49 (m, 4H, Ar*H*), 7.42 – 7.36 (m, 2H, Ar*H*), 7.26 – 7.22 (m, 1H, Ar*H*), 7.11 – 7.06 (m, 3H, Ar*H*), 6.78 (dd, *J* = 2.7, 1.7 Hz, 1H, Ar*H*), 2.31 (s, 3H, C*H3*). ^{13}C NMR (101 MHz, $CDCl_3$) δ 169.9, 148.9, 133.6, 133.3, 132.5, 129.1, 126.7, 126.3, 126.0, 124.0, 121.8, 115.7, 104.2, 21.3. HRMS (ESI/QTOF) m/z: $[M+Na]^+$ Calcd for $C_{18}H_{15}NNaO_2^+$ 300.0995; Found 300.0994.

8.5 Telescoped protected pyrrole formation

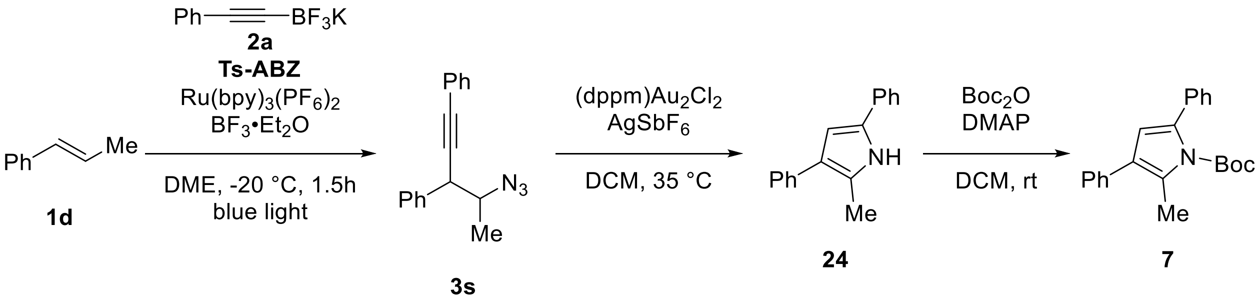

An oven-dried test tube charged with $Ru(bpy)_3(PF_6)_2$ (5.2 mg, 6.0 µmol, 0.02 equiv.), **Ts-ABZ** (166 mg, 0.375 mmol, 1.25 equiv.) and potassium trifluoro(phenylethynyl)borate (**2a**) (94 mg, 0.45 mmol, 1.50 equiv.) was evacuated and backfilled with N_2 (3x). Dry degassed DME (2.7 mL) and *trans-β*-methylstyrene (**1d**) (39 µL, 0.30 mmol, 1.00 equiv.) were added and the mixture was cooled to -20 °C. Then, a stock solution of BF_3•Et_2O (11 µL, 90 µmol, 0.30 equiv.) in dry degassed DME (0.34 mL) was added and the reaction was stirred under blue LEDs irradiation (1 x Kessil 467 nm 50% intensity, 22W) at -20 °C for 1.5 h. The reaction mixture

[26] D. J. Gorin, N. R. Davis, F. D. Toste, *J. Am. Chem. Soc.* **2005**, *127*, 11260–11261.

was concentrated *in vacuo*. The crude product was dissolved in DCM, filtered through a short plug of silica and eluted with DCM then concentrated *in vacuo* to afford crude **3s**.

A capped oven-dried microwave vial charged with $AgSbF_6$ (5.2 mg, 15 µmol, 0.050 equiv) and (dppm)Au_2Cl_2 (6.4 mg, 7.5 µmol, 0.025 equiv.) was evacuated and backfilled with N_2 (3x). Then, dry DCM (3 mL) was added and the mixture was stirred at rt for 2 min. A solution of previously obtained crude **3s** in dry DCM (3 mL) was added under N_2 and the vial was placed in a preheated heating block at 35 °C. The reaction was stirred at 35 °C for 1.5 h. The mixture was allowed to cool to rt, filtered over activated neutral alumina, eluted with DCM and concentrated *in vacuo* to afford crude **24**.

A capped microwave vial charged with previously obtained crude **24** and DMAP (7.3 mg, 60 µmol, 0.20 equiv) was evacuated and backfilled with N_2 (3x). Then, dry DCM (2 mL) was added followed by the dropwise addition of a solution of Boc_2O (131 mg, 0.600 mmol, 2.00 equiv.) in dry DCM (1 mL). The reaction was stirred at rt for 1.5 h then was concentrated *in vacuo*. The crude product was purified by column chromatography (pentane/Et_2O, 98:2 to 96:4) to afford *tert*-butyl 2-methyl-3,5-diphenyl-1*H*-pyrrole-1-carboxylate (**7**) (43 mg, 0.13 mmol, 43%) as a yellow oil.

R_f (pentane/Et_2O, 95:5): 0.25; ^{1}H NMR (400 MHz, $CDCl_3$) δ 7.43 – 7.33 (m, 8H, Ar*H*), 7.32 – 7.27 (m, 2H, Ar*H*), 6.28 (s, 1H, NC=C*H*), 2.54 (s, 3H, ArC*H₃*), 1.28 (s, 9H, Boc). ^{13}C NMR (101 MHz, $CDCl_3$) δ 150.4, 136.0, 135.2, 134.3, 128.8, 128.7, 128.5, 128.5, 128.0, 127.0, 126.3, 125.0, 113.6, 83.8, 27.5, 13.5. HRMS (ESI/QTOF) m/z: $[M+Na]^+$ Calcd for $C_{22}H_{23}NNaO_2^+$ 356.1621; Found 356.1620.

9. Mechanistic Studies

9.1 Carbocation trapping

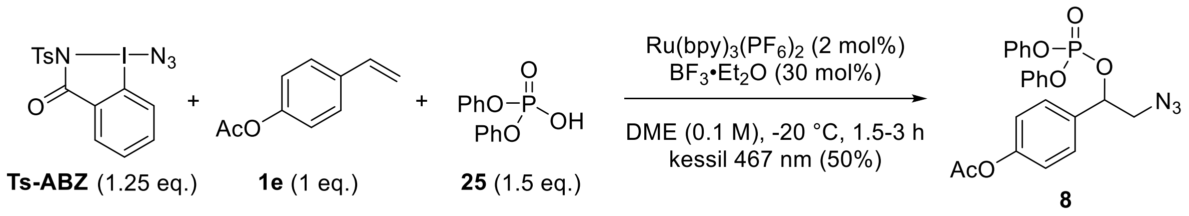

An oven-dried test tube charged with $Ru(bpy)_3(PF_6)_2$ (1.7 mg, 2.0 µmol, 0.02 equiv.), **Ts-ABZ** (55 mg, 0.13 mmol, 1.25 equiv.) and diphenyl phosphate (**25**) (38 mg, 0.15 mmol, 1.50 equiv.) was evacuated and backfilled with N_2 (3x). Dry degassed DME (0.9 mL) and 4-acetoxystyrene (**1e**) (15 µL, 0.10 mmol, 1.00 equiv.) were added and the mixture was cooled to -20 °C. Then, a stock solution of BF_3•Et_2O (3.7 µL, 30 µmol, 0.30 equiv.) in dry degassed DME (0.12 mL) was added and the reaction was stirred under blue LEDs irradiation (1 x Kessil 467 nm 50% intensity, 22 W) at -20 °C for 2 h. The reaction mixture was filtered through a short plug of silica and eluted with EtOAc then concentrated *in vacuo*. The crude product was dissolved in DCM (10 mL), the solution was washed with 3 x 10 mL of a sat. sol. of $NaHCO_3$. The organic layer was dried over $MgSO_4$, filtered and concentrated *in vacuo*. The crude product was purified by

preparative TLC (pentane/EtOAc, 65:35) to afford 4-(2-azido-1-((diphenoxyphosphoryl)oxy)ethyl)phenyl acetate (**8**) (27 mg, 59 μmol, 59%) as a colorless oil.

R_f (pentane/EtOAc, 7:3): 0.25; ^{1}H NMR (400 MHz, $CDCl_3$) δ 7.37 – 7.30 (m, 4H, Ar*H*), 7.27 – 7.16 (m, 5H, Ar*H*), 7.15 – 7.10 (m, 1H, Ar*H*), 7.09 – 7.04 (m, 2H, Ar*H*), 7.03 – 6.98 (m, 2H, Ar*H*), 5.62 (td, *J* = 7.6, 4.3 Hz, 1H, P(O)OC*H*), 3.64 (dd, *J* = 13.2, 7.4 Hz, 1H, CH_2N_3), 3.53 (ddd, *J* = 13.2, 4.2, 2.5 Hz, 1H, CH_2N_3), 2.30 (s, 3H, CH_3). ^{13}C NMR (101 MHz, $CDCl_3$) δ 169.3, 151.4, 150.5 (d, *J* = 7.1 Hz), 150.4 (d, *J* = 7.2 Hz), 133.9 (d, *J* = 3.0 Hz), 129.9, 129.8, 128.0, 125.6 (d, *J* = 1.1 Hz), 125.5 (d, *J* = 1.1 Hz), 122.1, 120.2 (d, *J* = 5.0 Hz), 120.1 (d, *J* = 4.9 Hz), 79.8 (d, *J* = 5.8 Hz), 56.3 (d, *J* = 7.6 Hz), 21.2. HRMS (Nanochip-based ESI/LTQ-Orbitrap) m/z: $[M+H]^+$ Calcd for $C_{22}H_{21}N_3O_6P^+$ 454.1162; Found 454.1163.

9.2 Stern-Volmer fluorescence quenching

Due to the low solubility of the different components of the reaction in pure DME a mixture of DME/CH_3CN (9:1) was chosen for the study. We observed only a very slight decreased in yield when the reaction was carried out in this solvent mixture (Table S8, entry 7). All the solvent used during the study was dry and sparged with argon for 30 minutes before use. Stern-Volmer fluorescence quenching experiments were conducted on a Varian Cary Eclipse machine.

Preparation of the solutions:

Solution **A**: A 20 mL volumetric flask charged with $Ru(bpy)_3(PF_6)_2$ (4.3 mg, 5.0 μmol) was evacuated and backfilled with N_2 (3x). The solvent was added (~16 mL) and the flask was vigorously shaken to ensure solubilization. Solvent was further added until the line and the flask was further shaken. The solution was stored under argon atmosphere throughout the experiments.

Solution **B**: A 25 mL volumetric flask charged with potassium trifluoro(phenylethynyl)borate (**2a**) (130 mg, 0.625 mmol) was evacuated and backfilled with N_2 (3x). The solvent was added (~20 mL) and the flask was vigorously shaken to ensure solubilization. Solvent was further added until the line and the flask was further shaken. The solution was stored under argon atmosphere throughout the experiments.

Solution **C**: A 10 mL volumetric flask charged with **Ts-ABZ** (111 mg, 0.250 mmol) was evacuated and backfilled with N_2 (3x). The solvent was added (~7 mL) and the flask was vigorously shaken to ensure solubilization. Solvent was further added until the line and the flask was further shaken. The solution was stored under argon atmosphere throughout the experiments.

Solution **D**: A 5 mL volumetric flask charged with styrene (**1a**) (36 μL, 0.31 mmol) was evacuated and backfilled with N_2 (3x). The solvent was added (~3 mL) and the flask was vigorously shaken. Solvent was further added until the line and the flask was further shaken. The solution was stored under argon atmosphere throughout the experiments.

Preparation of a sample:

A quartz cuvette fitted with a septum was evacuated and backfilled with N_2 (3x). Then, 0.5 mL of solution **A**, the appropriate amount of quencher solution and solvent were added under N_2. The final volume of the solution was always 2.5 mL with a final concentration of the photocatalyst of $5x10^{-5}$ M. The mixture was vortexed for 5 s and the analysis was performed.

V_A (mL)	V_B (mL)	$V_{solvent}$	Quencher concentration (mM)
0.5	0	2	0
0.5	0.5	1.5	5
0.5	1	1	10
0.5	1.5	0.5	15
0.5	2	0	20

Table S9: Alkynyl-BF_3K **2a** as quencher (solution **B**).

V_A (mL)	V_C (mL)	$V_{solvent}$	Quencher concentration (mM)
0.5	0	2	0
0.5	0.5	1.5	5
0.5	1	1	10
0.5	1.5	0.5	15
0.5	2	0	20

Table S10: Ts-ABZ as quencher (solution **C**).

V_A (mL)	V_D (mL)	$V_{solvent}$	Quencher concentration (mM)
0.5	0	2	0
0.5	0.2	1.8	5
0.5	0.4	1.6	10
0.5	0.6	1.4	15
0.5	0.8	1.2	20

Table S11: Styrene **1a** as quencher (solution **D**).

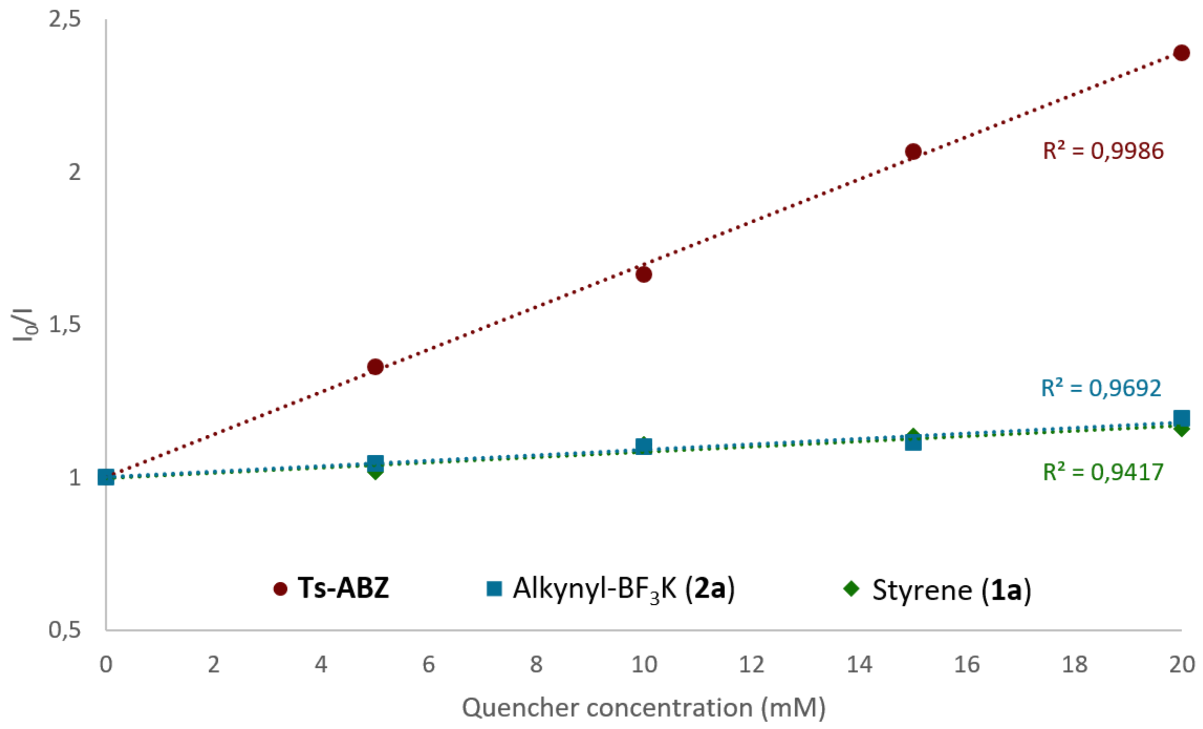

Figure S2: Stern-Volmer fluorescence quenching results.

9.3 Investigation of the BF_3•Et_2O effect

As described in the manuscript we did not reach a proper conclusion on the role of BF_3. We investigated different hypothesis for its effect:

1) It could scavenge water from the reaction mixture:

Since the reaction is occurring via the formation of a carbocation intermediate we usually observed small amount of water addition (oxy-azidation). When BF_3 is added the formation of this by-product occurs similarly.

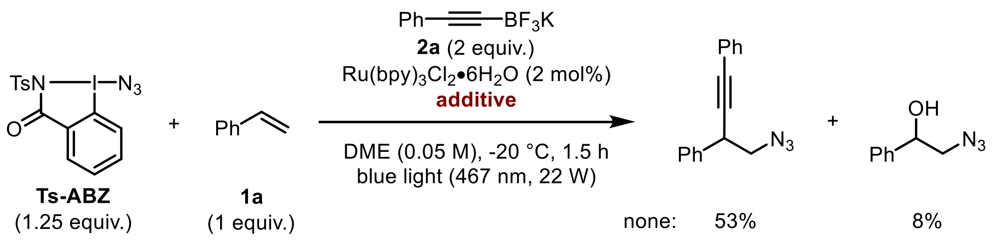

none:	53%	8%
BF_3•Et_2O (30 mol%):	72%	9%
MS 4A (50 mg/0.1 mmol):	51%	trace

Additionally, when the reaction was performed in the presence of molecular sieves almost no oxy-azidation product was observed but the yield did not increase.

2) Activation of Ts-ABZ:

BF_3 is known to activate different hypervalent iodine reagent by coordinating the ligands.[27] In our case, NMR studies show no significant shift of the signals corresponding to Ts-ABZ in the presence of BF_3 in DME.

3) Abstraction of fluoride from alkynyl-BF_3K:

BF_3 is known to abstract fluoride from trifluoroborate salts to form BF_4^- and neutral R-BF_2 compounds. This reactivity is known for alkynyl-BF_3K[28] and a control experiment in which alkynyl-BF_2 was preformed and then added to the reaction afforded similar results as the standard conditions:

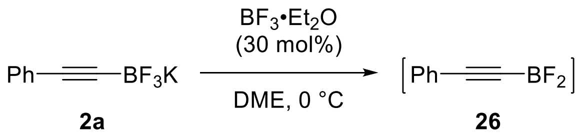

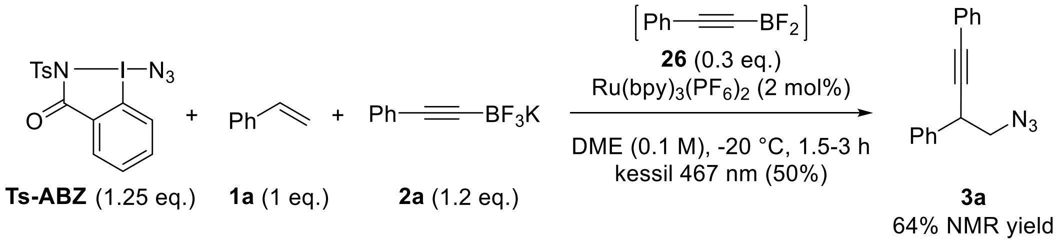

A capped oven-dried microwave vial charged with potassium trifluoro(phenylethynyl)borate (**2a**) (19 mg, 90 µmol, 0.9 equiv.) was evacuated and backfilled with N_2 (3x). Dry degassed DME (0.54 mL) was added and the solution was cooled to 0 °C. Then, a stock solution of BF_3•Et_2O (11 µL, 90 µmol, 0.9 equiv.) in dry DME (0.36 mL) was added and the mixture was stirred at 0 °C for 30 min.

An oven-dried test tube charged with Ru(bpy)$_3$(PF$_6$)$_2$ (1.7 mg, 2.0 µmol, 0.02 equiv.), **Ts-ABZ** (55 mg, 0.13 mmol, 1.25 equiv.) and potassium trifluoro(phenylethynyl)borate (**2a**) (25 mg, 0.12 mmol, 1.20 equiv.) was evacuated and backfilled with N_2 (3x). Dry degassed DME (0.7 mL) and styrene (**1a**) (11.5 µL, 0.100 mmol, 1.00 equiv.) were added and the mixture was cooled to -20 °C. Then, the stock solution of **26** in dry degassed DME (0.34 mL, 0.3 equiv.) was added and the reaction was stirred under blue LEDs irradiation (1 x Kessil 467 nm 50% intensity, 22 W) at -20 °C for 1.5 h. The reaction mixture was filtered through a short plug of silica and eluted with DCM then concentrated *in vacuo*. Crude NMR yield of **3a** was determined to be 64% using CH_2Br_2 (3.5 µL, 50 µmol, 0.5 equiv.) as internal standard.

A control reaction was carried out in parallel using the standard conditions on 0.1 mmol scale and afforded **3a** in 71% NMR yield.

[27] A. Dasgupta, C. Thiehoff, P. D. Newman, T. Wirth, R. L. Melen, *Org. Biomol. Chem.* **2021**, *19*, 4852–4865.
[28] T. A. Mitchell, J. W. Bode, *J. Am. Chem. Soc.* **2009**, *131*, 18057–18059.

This result suggest that the 30 mol% of BF_3 are consumed to form alknyl-BF_2. The implication of this species in the reaction is not yet clear. A highly speculative explanation would rely on a potential coordination with the reactive azide intermediate (either radical or carbocation) leading to a pseudo intramolecular delivery of the alkyne, which could explain the higher yield. Similar intermolecular deliveries using oxygen anchors have been proposed.[29]

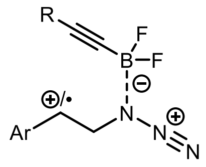

To try to probe this interaction we added a solution of alkynyl-BF_2 to benzyl azide. No NMR shift of the benzylic proton were observed.

Alknyl-BF_2 could also be directly quenched by another nucleophile in the mixture, such as 2-iodotosylbenzamide, leading to the formation of a potentially more nucleophilic tetravalent alkynyl-BF_2X^- salt.

With all the observation listed above we cannot properly conclude on the exact role of BF_3 although evidence suggest the formation of alknyl-BF_2 but its effect is still unclear. Those species are known to be in equilibrium in solution with other tetravalent boron compound which often lead to complex mechanistic scenarios.[30]

9.4 Cyclic voltammetry of Ts-ABZ

Cyclic voltammetry (CV) was performed using a BioLogic Potentiostat SP-150. Electrochemical cell consisted of a 10 mL glass sample vial (VC-4 type, ALS Co., LTD) equipped with a corresponding Teflon cap, an Ag wire coated with AgCl in 3 M NaCl as a reference electrode, a Pt wire as a counter electrode and a Pt surface with a diameter of 6 mm as a working electrode. Dry degassed solution of 0.1 M *n*-Bu_4NPF_6 in MeCN was used as a support electrolyte. A ferrocene/ferrocenium (Fc/Fc^+) couple was used as an internal reference. Conversion from Fc/Fc^+ to SCE was carried out using the reference therein.[31]

[29] (a) R. William, S. Wang, A. Mallick, X.-W. Liu, *Org. Lett.* **2016**, *18*, 4458–4461. (b) S. Roscales, V. Ortega, A. G. Csákÿ, *J. Org. Chem.* **2018**, *83*, 11425–11436.
[30] C.-V. T. Vo, T. A. Mitchell, J. W. Bode, *J. Am. Chem. Soc.* **2011**, *133*, 14082–14089.
[31] V. V. Pavlishchuk, A. W. Addison, *Inorganica Chim. Acta* **2000**, *298*, 97–102.

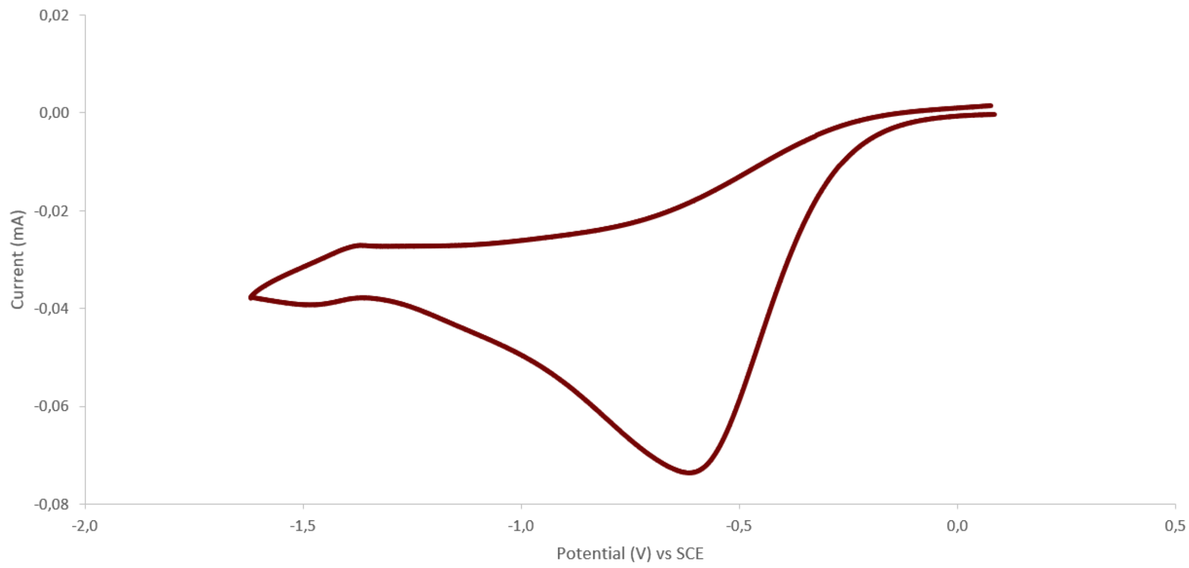

Figure S3: CV of **Ts-ABZ** vs SCE (0.1 M [TBA]PF_6 in CH_3CN), scan rate = 100 mV/s, $E_{1/2}^{red}$ = -0.62 V.

10. Crystal Structures

10.1 3i

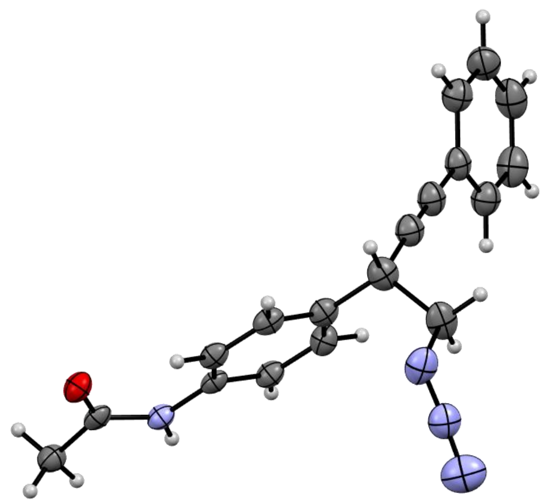

Figure S4: Ellipsoid plot (probability level 50%) of **3i**.

**Compound**	**3i**
Formula	$C_{18}H_{16}N_4O$
$D_{calc.}$/ g cm^{-3}	1.295
μ/mm^{-1}	0.672
Formula Weight	304.35
Colour	clear pale colourless
Shape	plate-shaped
Size/mm^3	0.24×0.20×0.07
T/K	140.00(10)
Crystal System	orthorhombic
Space Group	*Pccn*
a/Å	43.729(2)
b/Å	9.4992(4)
c/Å	7.5184(3)
α/°	90
β/°	90
γ/°	90
V/Å^3	3123.1(3)
Z	8
Z'	1
Wavelength/Å	1.54184
Radiation type	Cu K_α
Θ_{min}/°	4.044
Θ_{max}/°	74.799
Measured Refl's.	11322
Indep't Refl's	3060
Refl's I≥2 σ(I)	2117
R_{int}	0.0431
Parameters	250
Restraints	122
Largest Peak	0.223
Deepest Hole	-0.196
GooF	1.041
wR_2 (all data)	0.1460
wR_2	0.1309
R_1 (all data)	0.0855
R_1	0.0552
CCDC number	2264031

Crystals were grown by dissolving **3i** in a small amount of DCM (~1mL), addition of hexane (~10 mL) and slow evaporation of this mixture over several days.

Analysis of the crystal: A suitable crystal with dimensions 0.24 × 0.20 × 0.07 mm^3 was selected and mounted on a XtaLAB Synergy R, DW system, HyPix-Arc 150 diffractometer. The crystal was kept at a steady T = 140.00(10) K during data collection. The structure was solved with the **ShelXT** (Sheldrick, 2015) solution program using dual methods and by using **Olex2** 1.5 (Dolomanov et al., 2009) as the graphical interface. The model was refined with **ShelXL** 2018/3 (Sheldrick, 2015) using full matrix least squares minimisation on ***F*^{2}**.

10.2 6

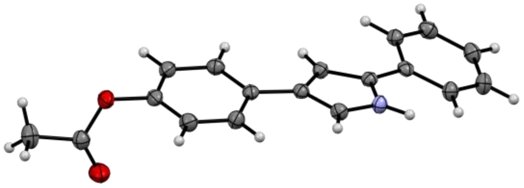

Figure S5: Ellipsoid plot (probability level 50%) of **6**.

Crystals were grown by dissolving **6** in a small amount of DCM (~1mL), addition of hexane (~10 mL) and slow evaporation of this mixture over several days.

Analysis of the crystal: A suitable crystal with dimensions 0.18 × 0.14 × 0.02 mm^3 was selected and mounted on an XtaLAB Synergy R, DW system, HyPix-Arc 150 diffractometer. The crystal was kept at a steady *T* = 140.00(10) K during data collection. The structure was solved with the **ShelXT** 2018/2 (Sheldrick, 2015) solution program using dual methods and by using **Olex2** 1.5 (Dolomanov et al., 2009) as the graphical interface. The model was refined with **ShelXL** 2018/3 (Sheldrick, 2015) using full-matrix least-squares minimisation on F^2.

**Compound**	**6**
Formula	$C_{18}H_{15}NO_2$
$D_{calc.}$/ g cm^{-3}	1.294
μ/mm^{-1}	0.677
Formula Weight	277.31
Colour	colourless
Shape	plate-shaped
Size/mm^3	0.18×0.14×0.02
T/K	140.00(10)
Crystal System	orthorhombic
Flack Parameter	0.04(9)
Space Group	$P2_12_12_1$
a/Å	5.80631(11)
b/Å	7.77611(12)
c/Å	31.5189(6)
α/°	90
β/°	90
γ/°	90
V/Å^3	1423.09(4)
Z	4
Z'	1
Wavelength/Å	1.54184
Radiation type	Cu*Kα*
Θ_{min}/°	2.804
Θ_{max}/°	75.554
Measured Refl's.	12451
Indep't Refl's	2932
Refl's I≥2σ(I)	2786
R_{int}	0.0183
Parameters	197
Restraints	0
Largest Peak/e Å^{-3}	0.211
Deepest Hole/e Å^{-3}	-0.148
GooF	1.034
wR_2 (all data)	0.0782
wR_2	0.0770
R_1 (all data)	0.0318
R_1	0.0297
CCDC number	2243553

11. Spectra of New Compounds

^{1}H NMR (400 MHz, $CDCl_3$) of **Ts-ABZ**:

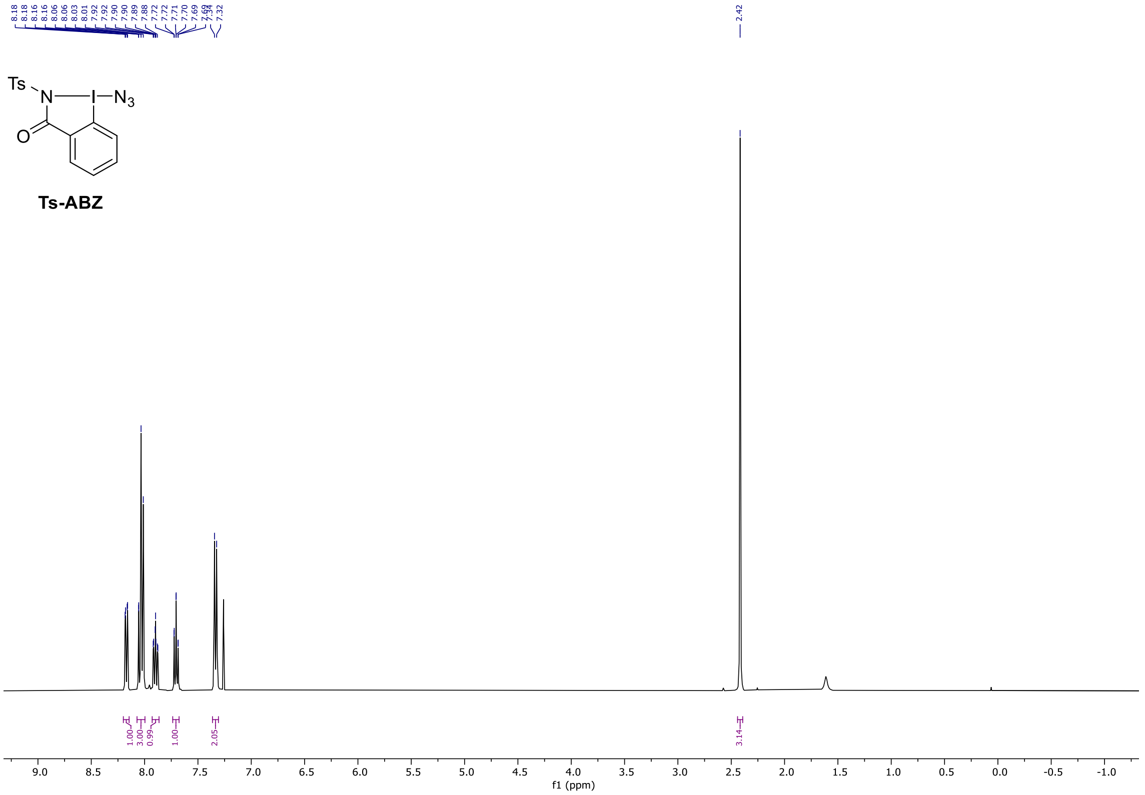

^{13}C NMR (101 MHz, $CDCl_3$) of **Ts-ABZ**:

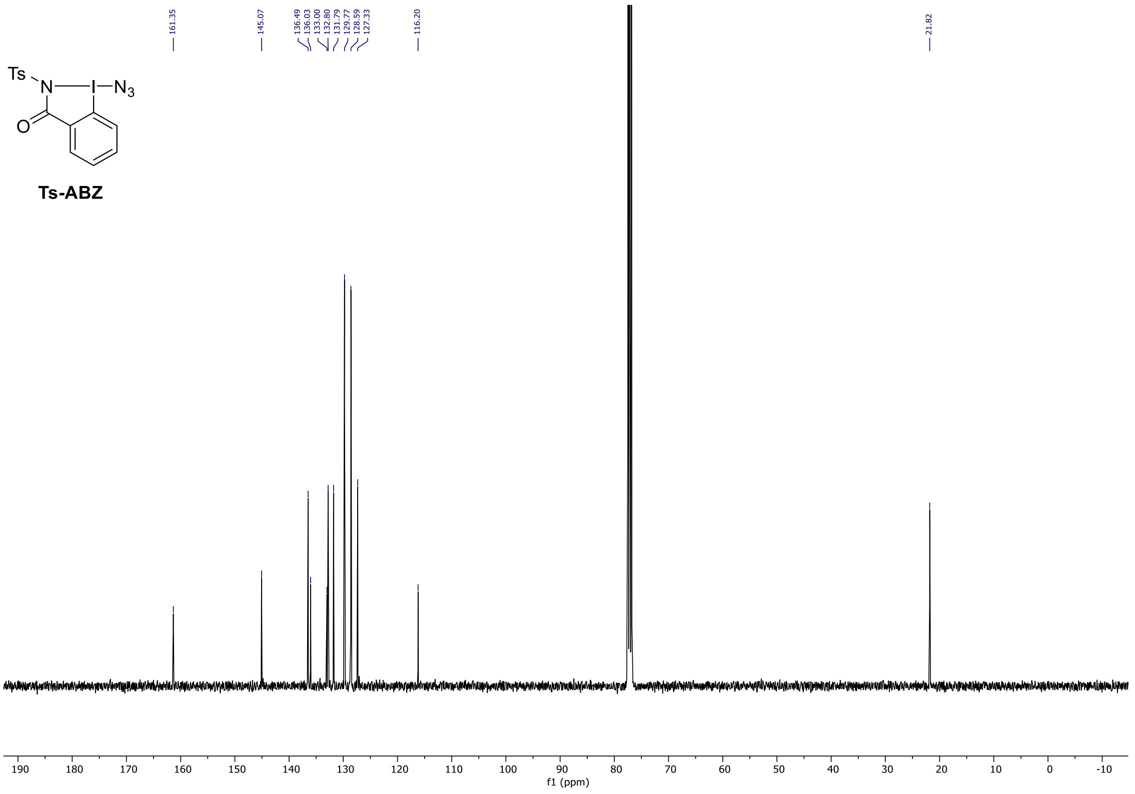

^1H NMR (400 MHz, acetone-d_6) of compound **2e**:

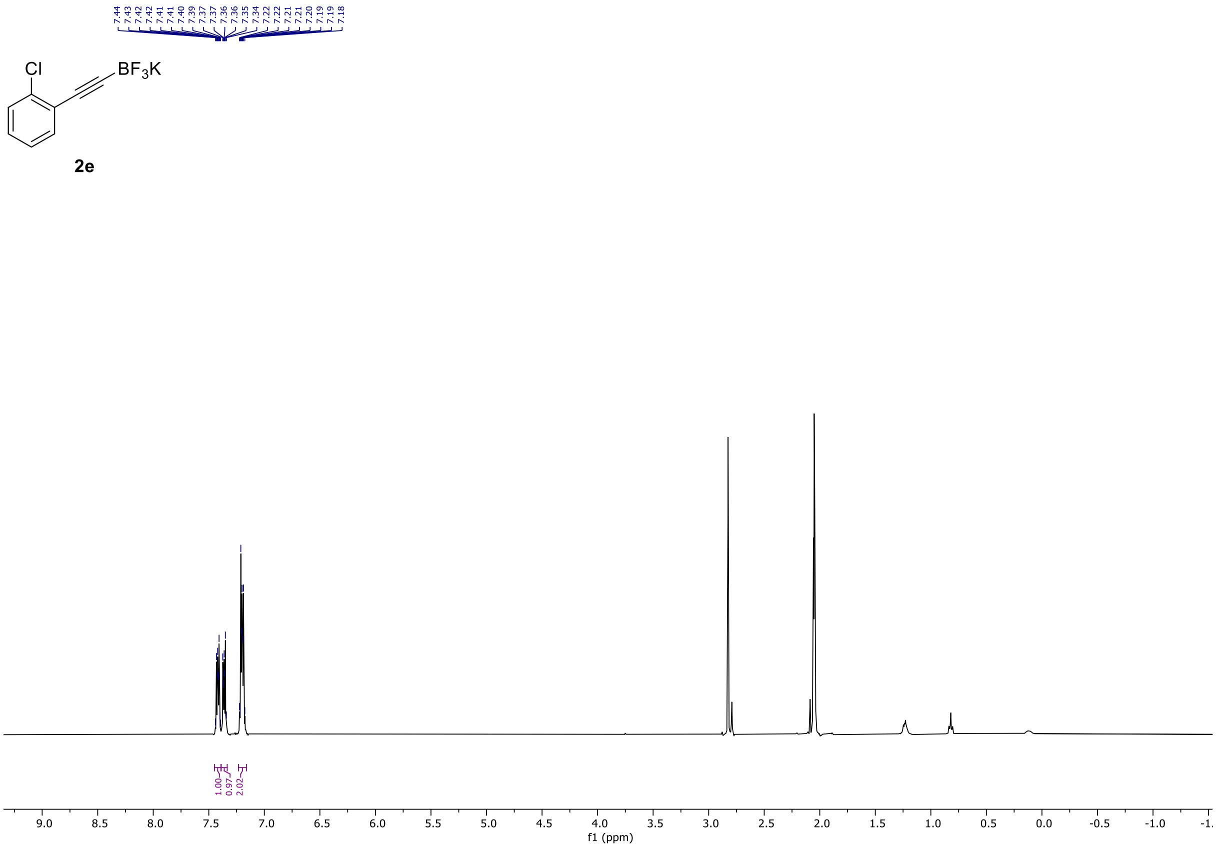

^{13}C NMR (101 MHz, acetone-d_6) of compound **2e**:

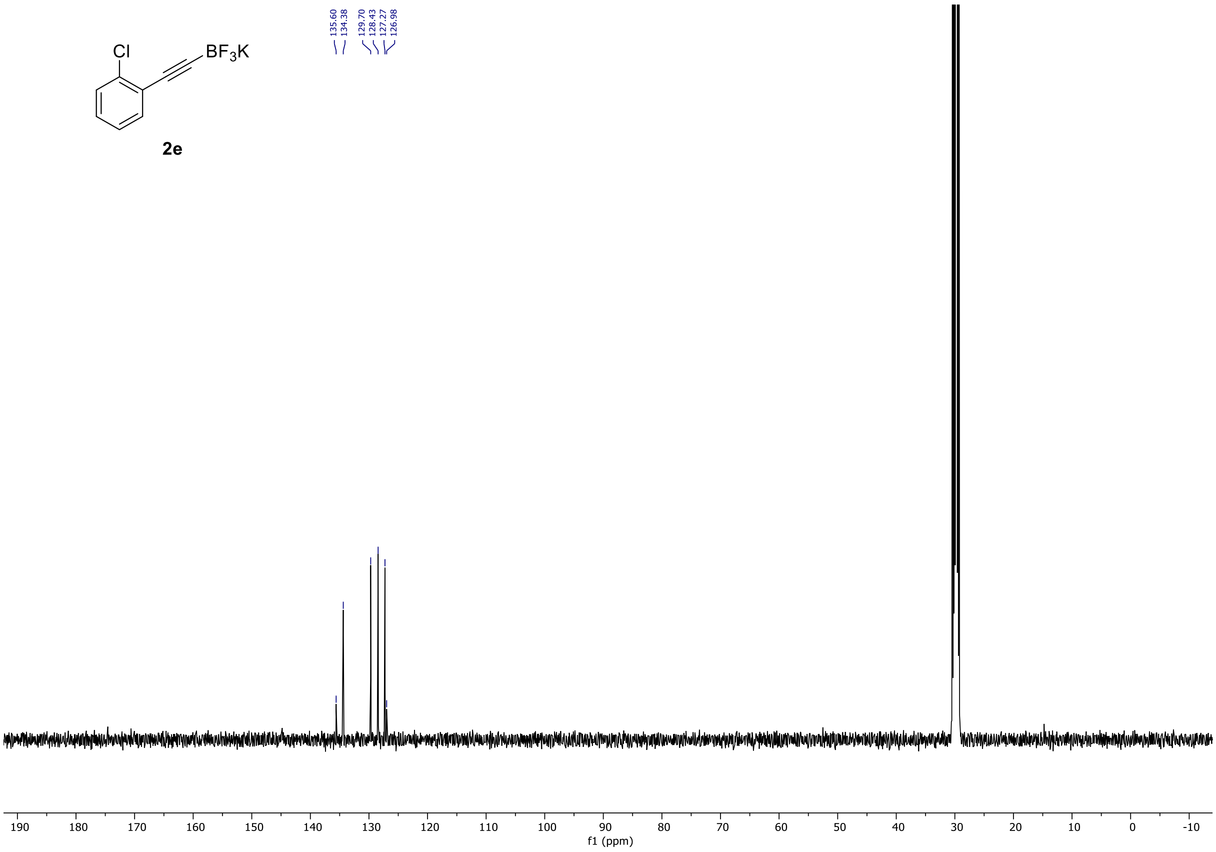

^{19}F NMR (376 MHz, acetone-d$_6$) of compound **2e**:

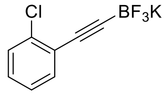

2e

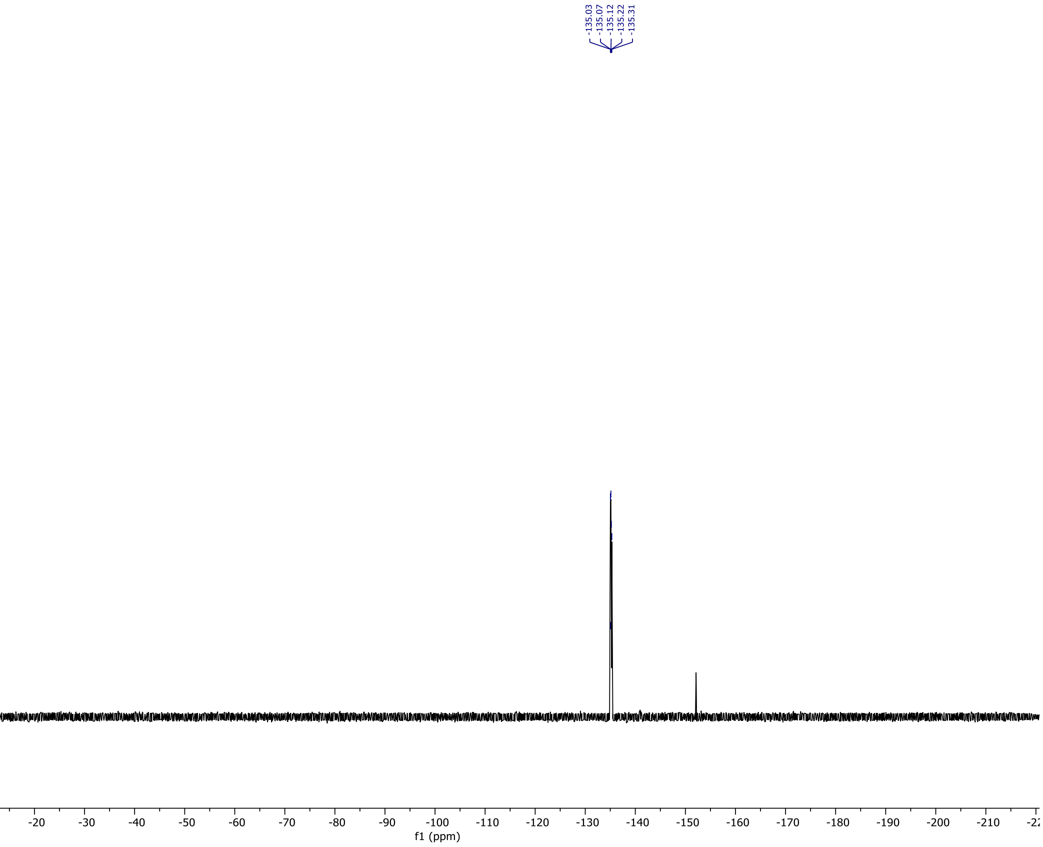

^{11}B NMR (128 MHz, acetone-d$_6$) of compound **2e**:

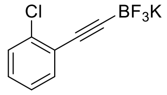

2e

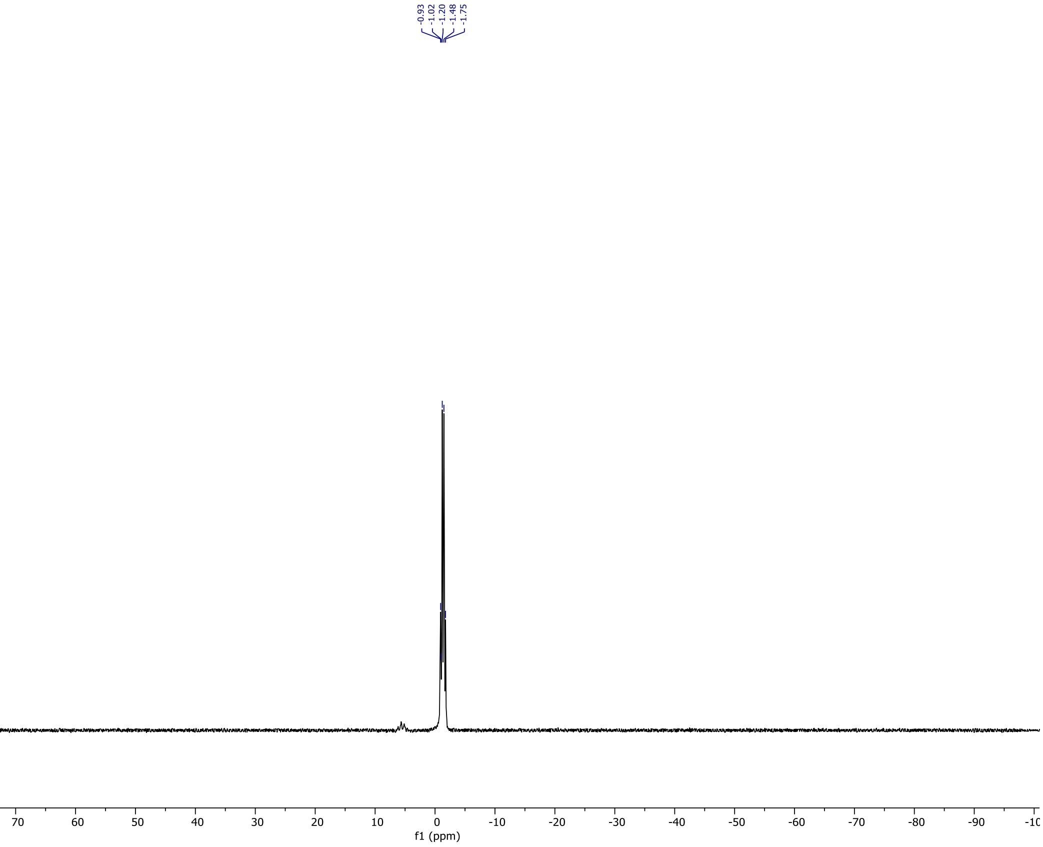

^{1}H NMR (400 MHz, acetone-d$_6$) of compound **2h**:

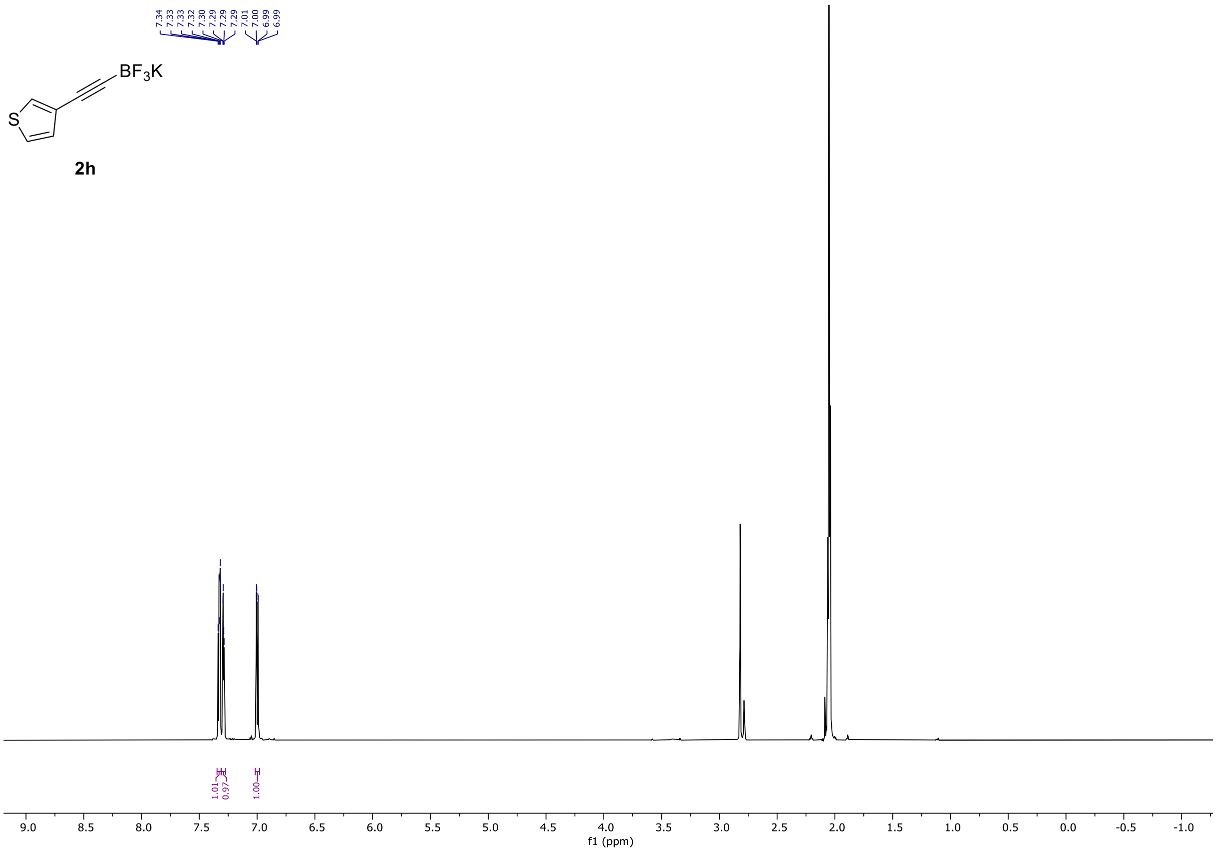

^{13}C NMR (101 MHz, acetone-d$_6$) of compound **2h**:

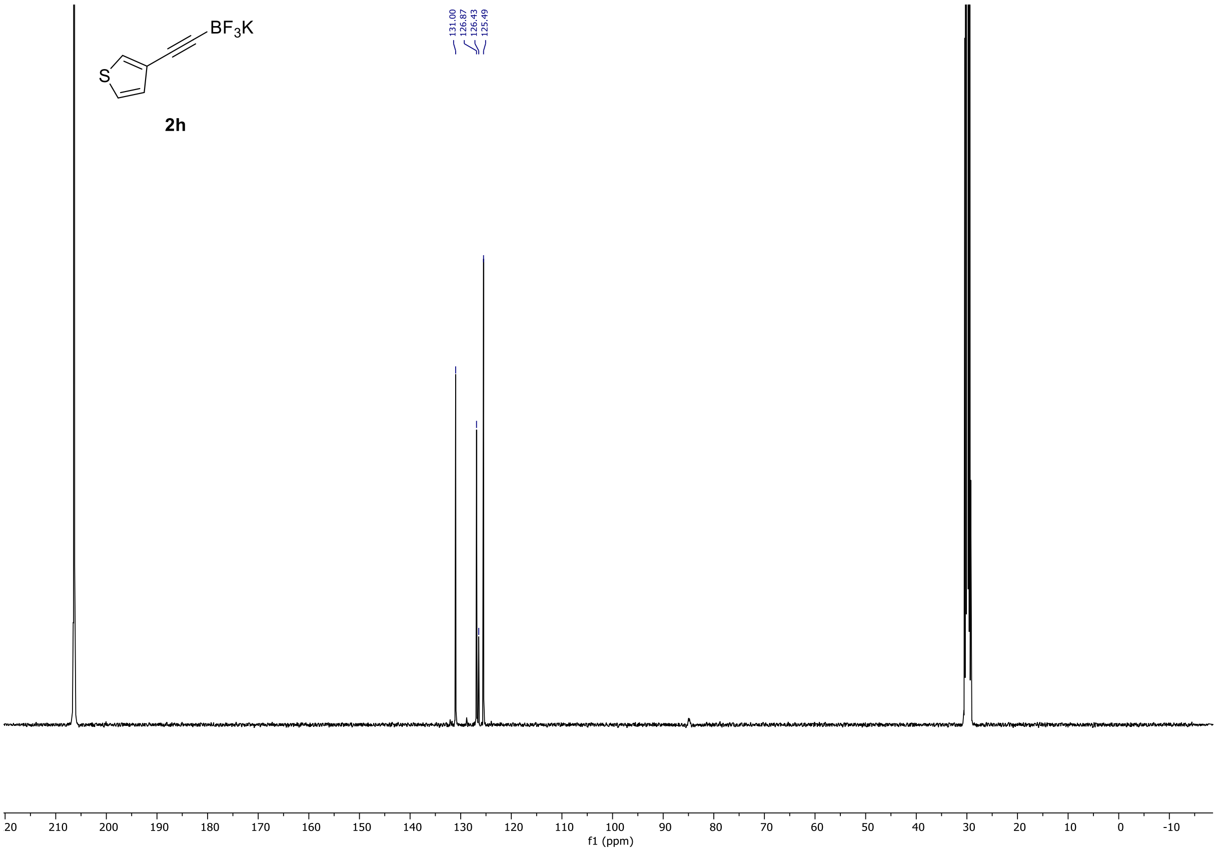

^{19}F NMR (376 MHz, acetone-d$_6$) of compound **2h**:

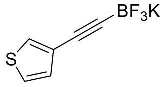

2h

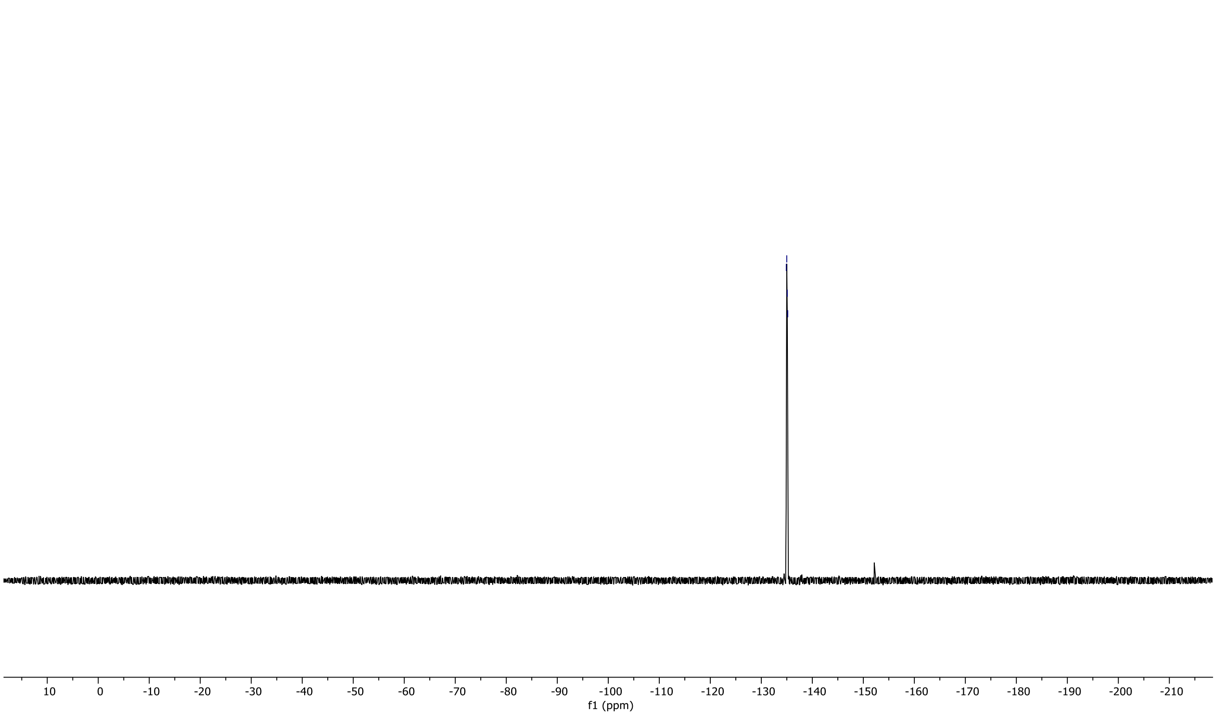

^{11}B NMR (128 MHz, acetone-d$_6$) of compound **2h**:

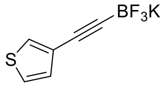

2h

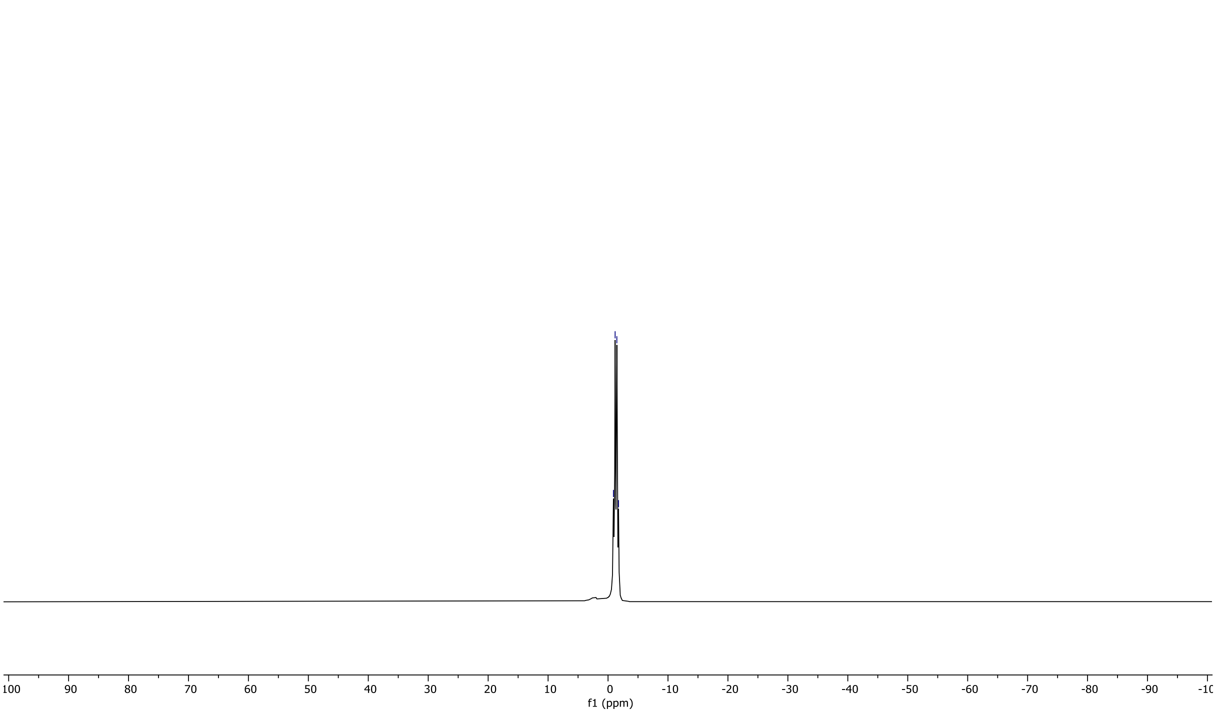

^{1}H NMR (400 MHz, $CDCl_3$) of compound **3a**:

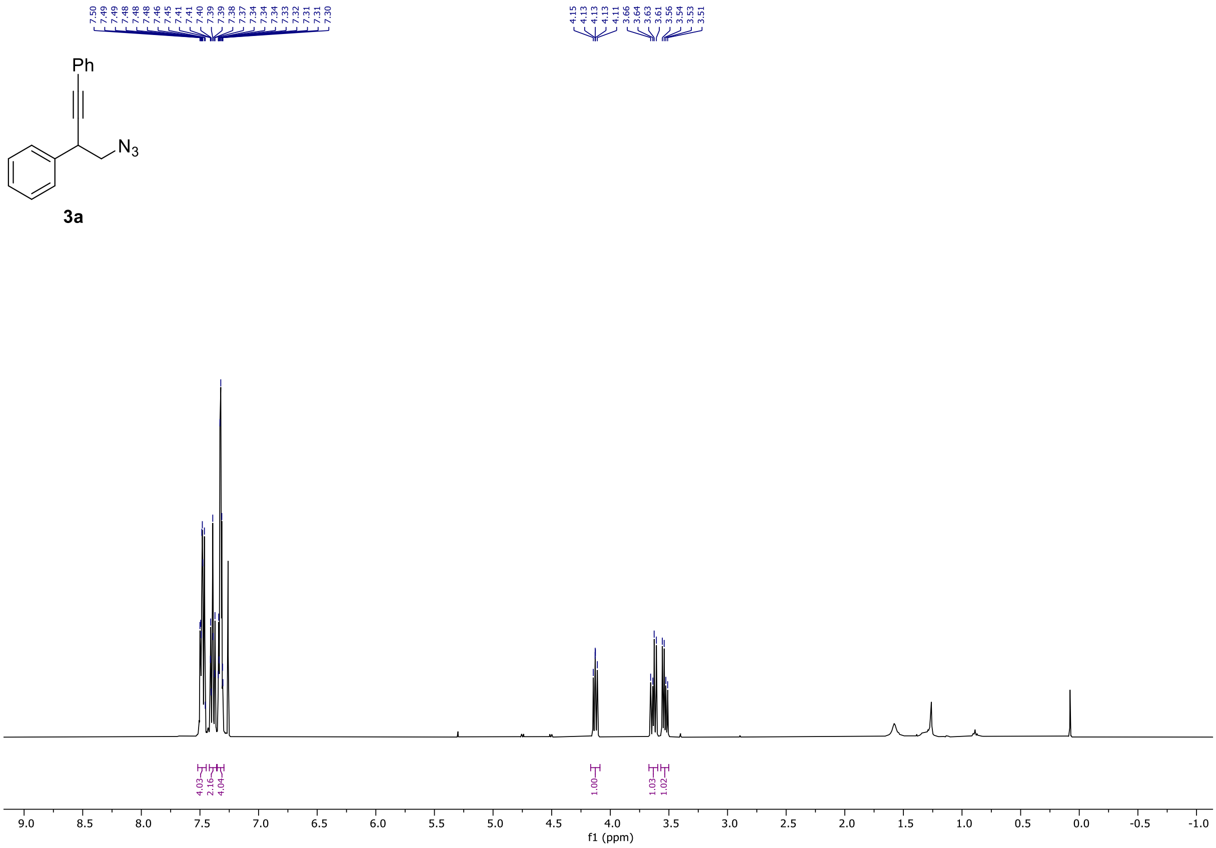

^{13}C NMR (101 MHz, $CDCl_3$) of compound **3a**:

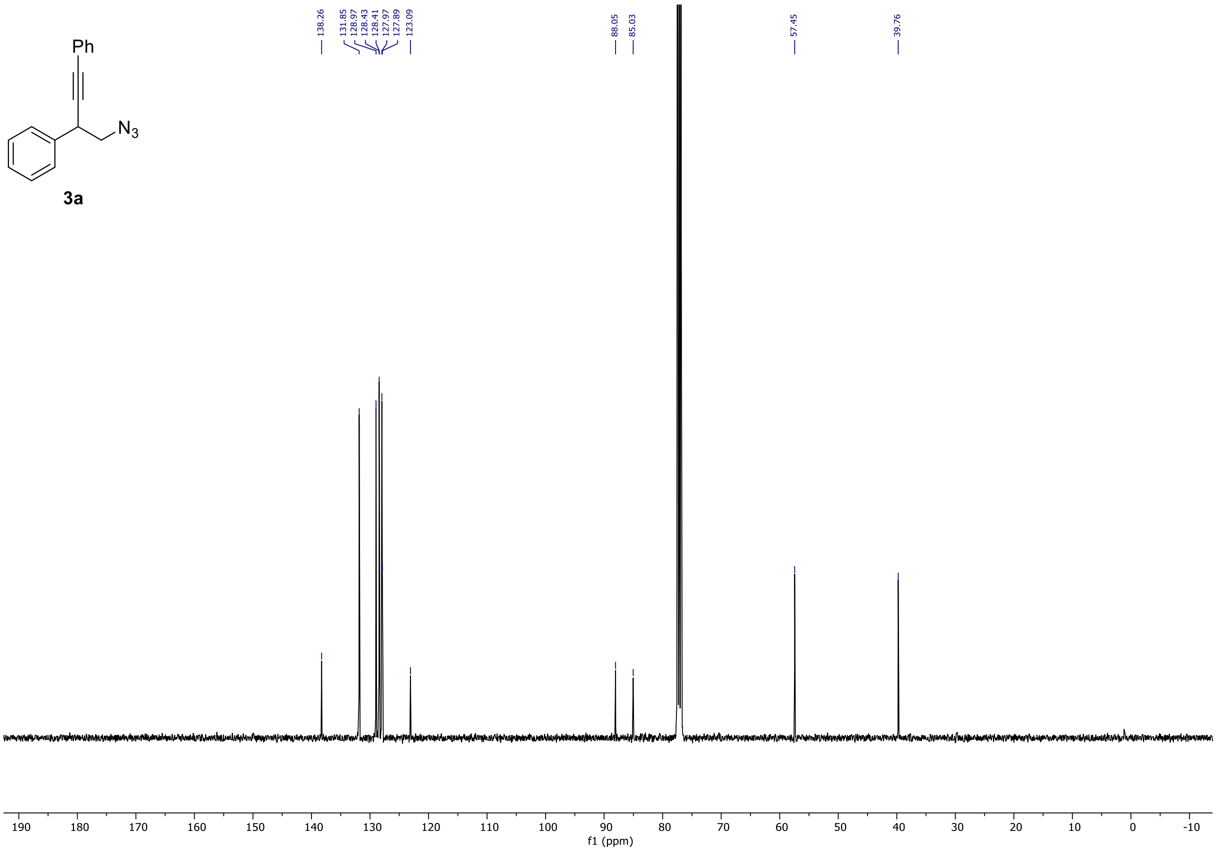

^{1}H NMR (400 MHz, $CDCl_3$) of compound **3b**:

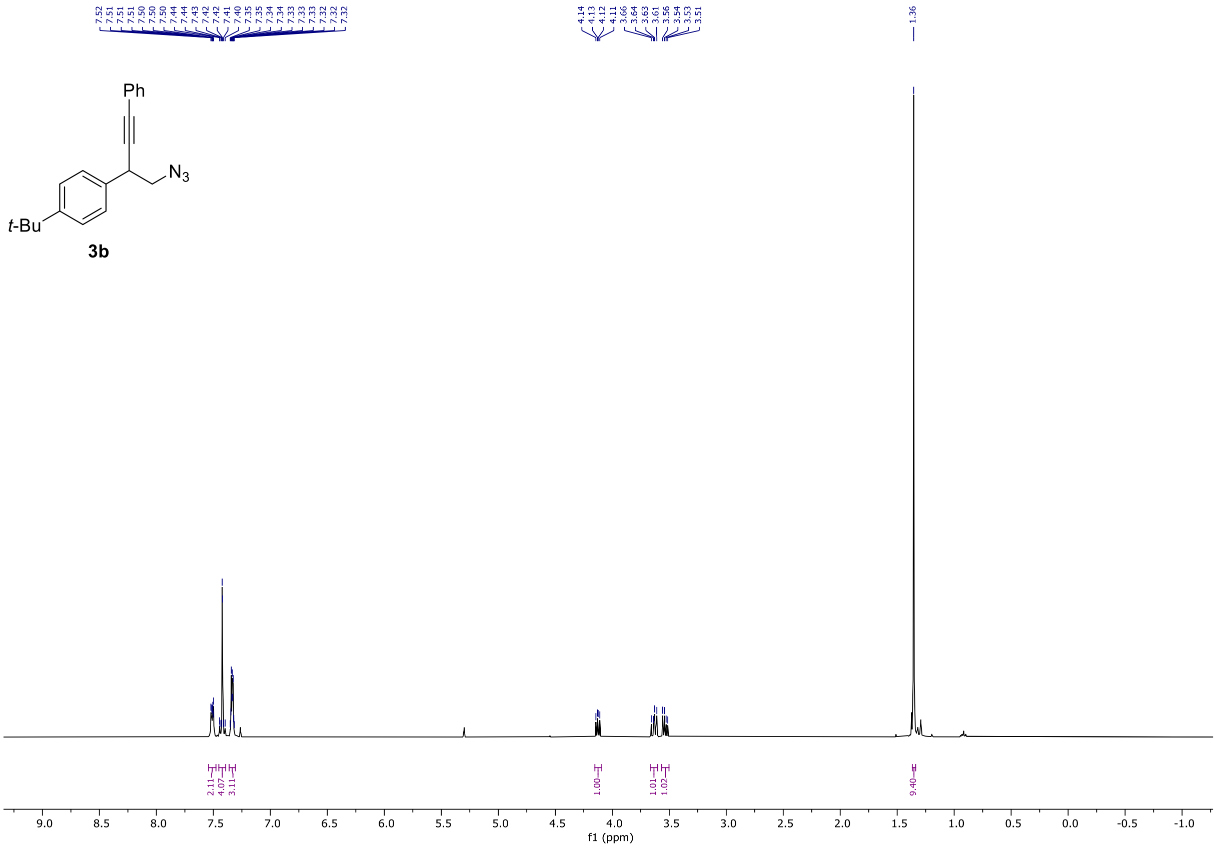

^{13}C NMR (101 MHz, $CDCl_3$) of compound **3b**:

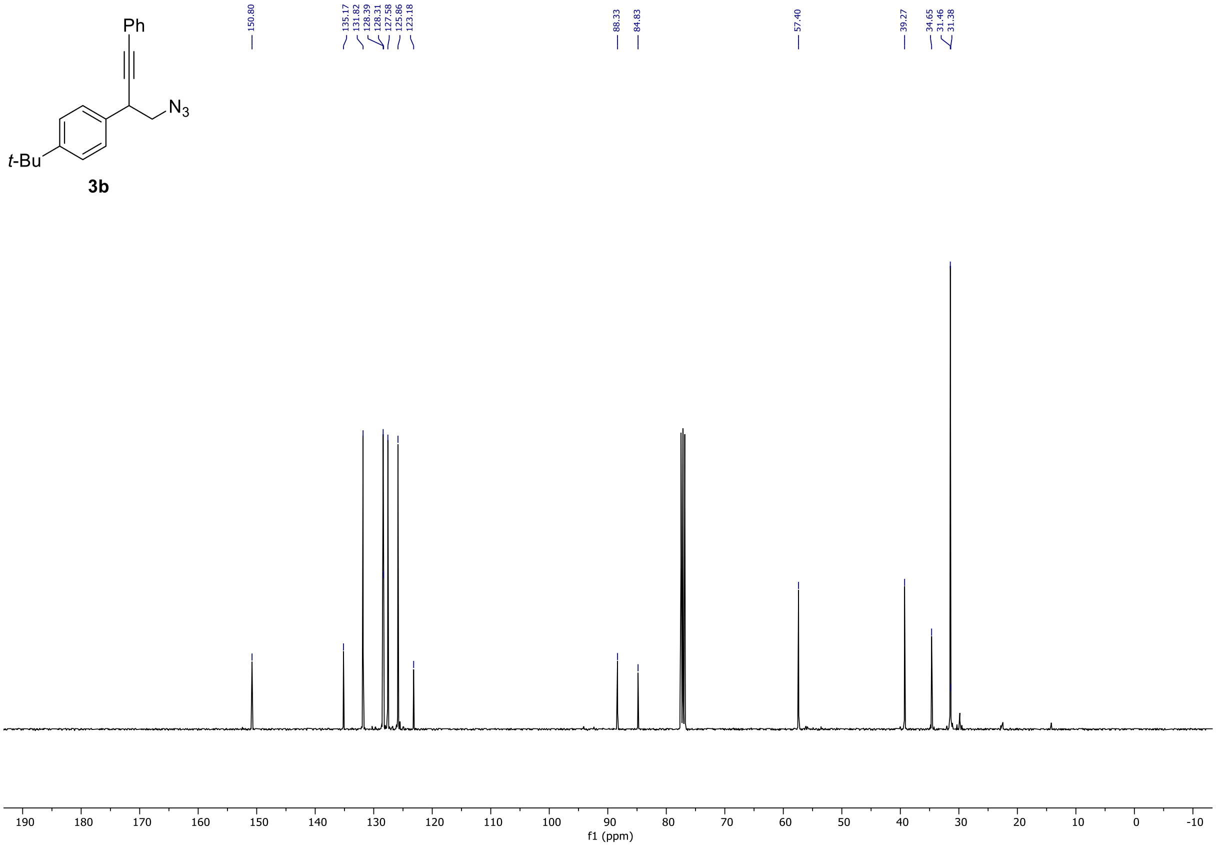

^{1}H NMR (400 MHz, $CDCl_3$) of compound **3c**:

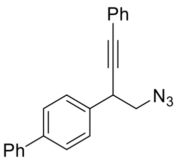

3c

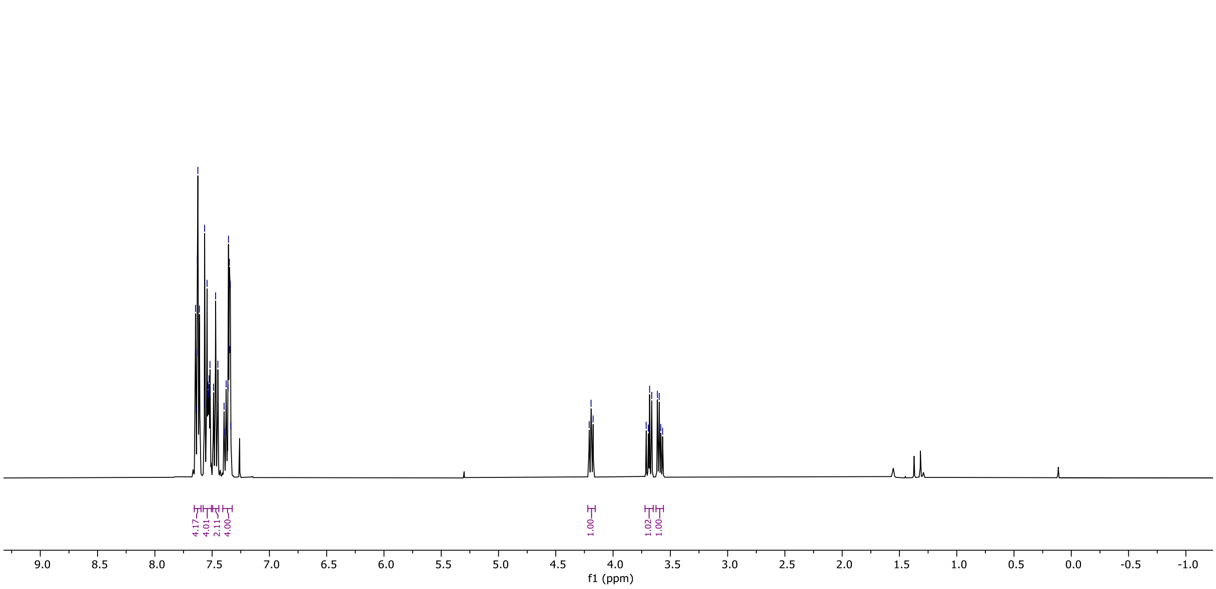

^{13}C NMR (101 MHz, $CDCl_3$) of compound **3c**:

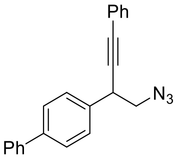

3c

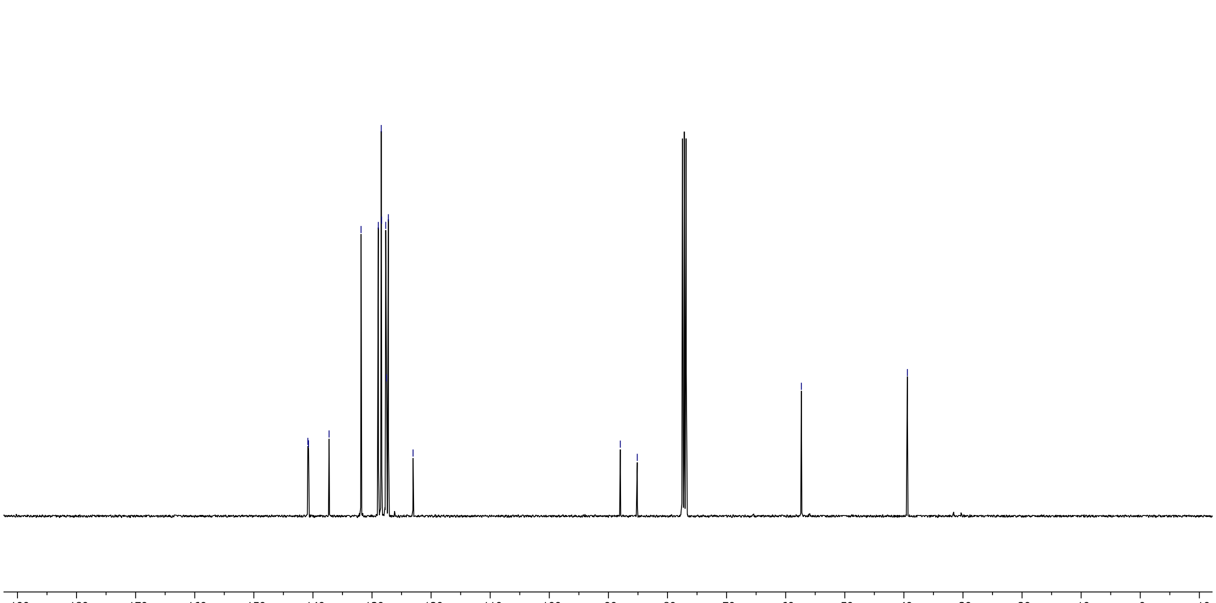

^{1}H NMR (400 MHz, $CDCl_3$) of compound **3d**:

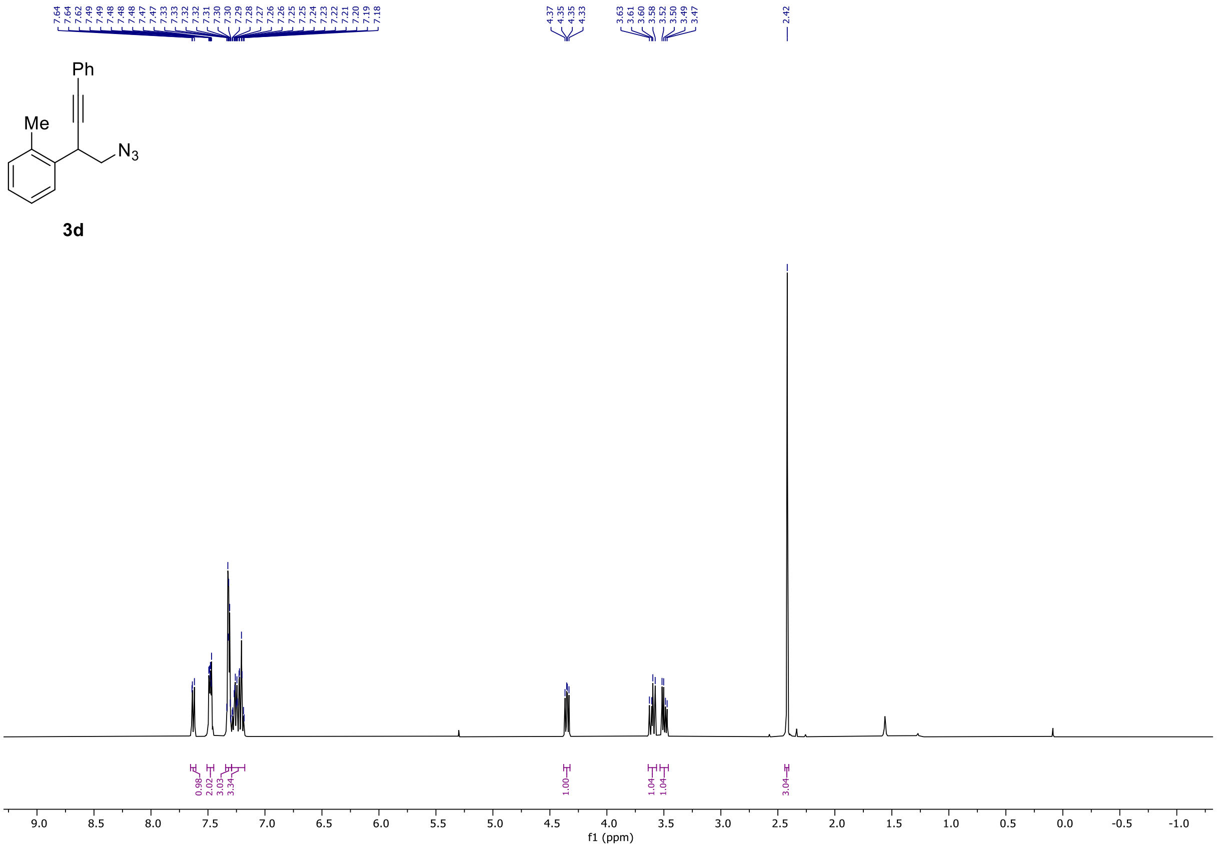

^{13}C NMR (101 MHz, $CDCl_3$) of compound **3d**:

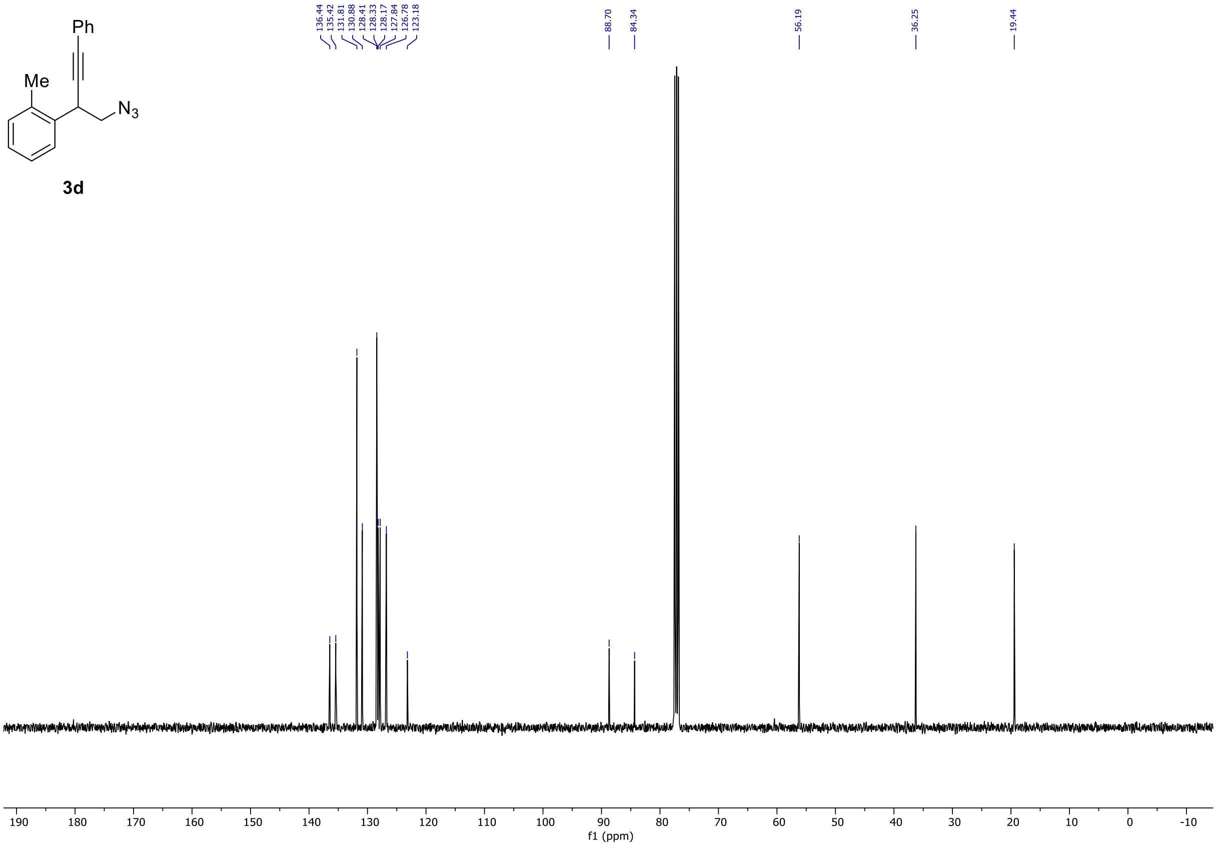

^{1}H NMR (400 MHz, $CDCl_3$) of compound **3e**:

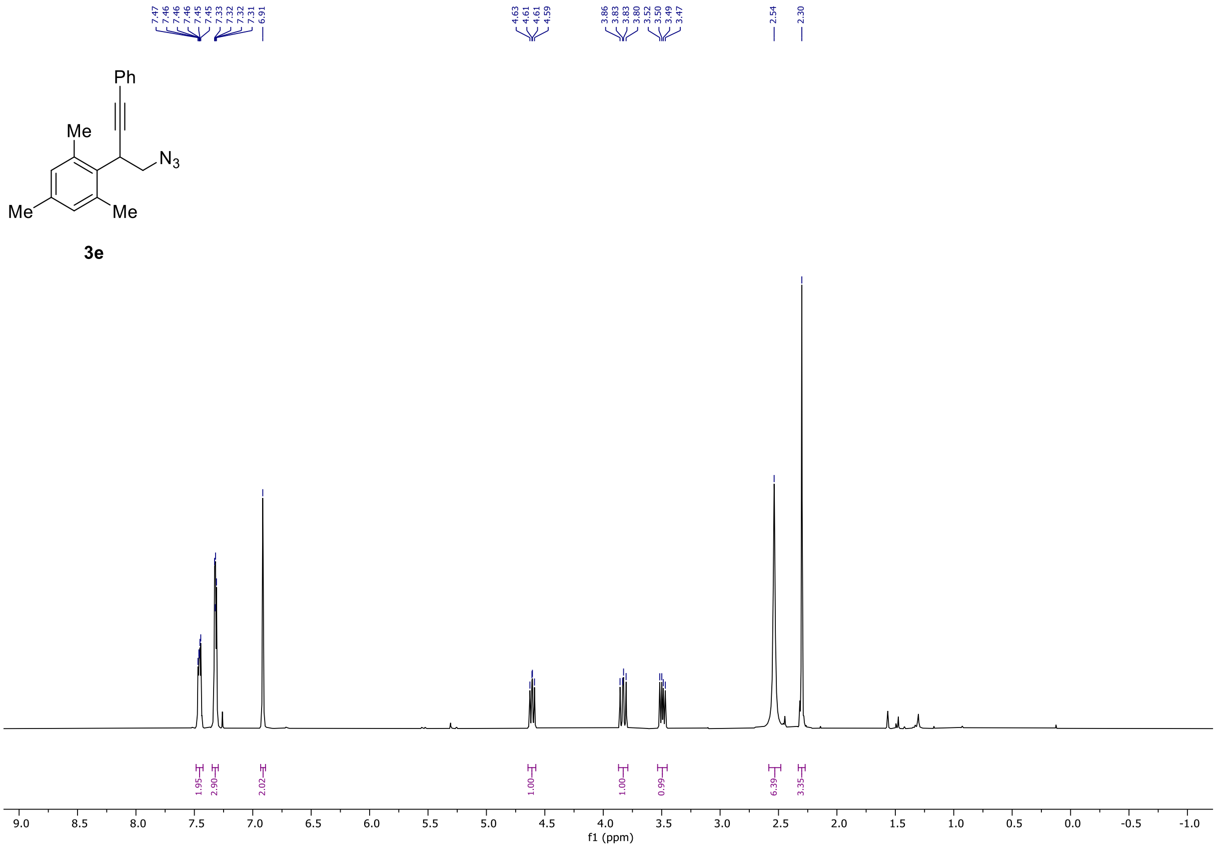

^{13}C NMR (101 MHz, $CDCl_3$) of compound **3e**:

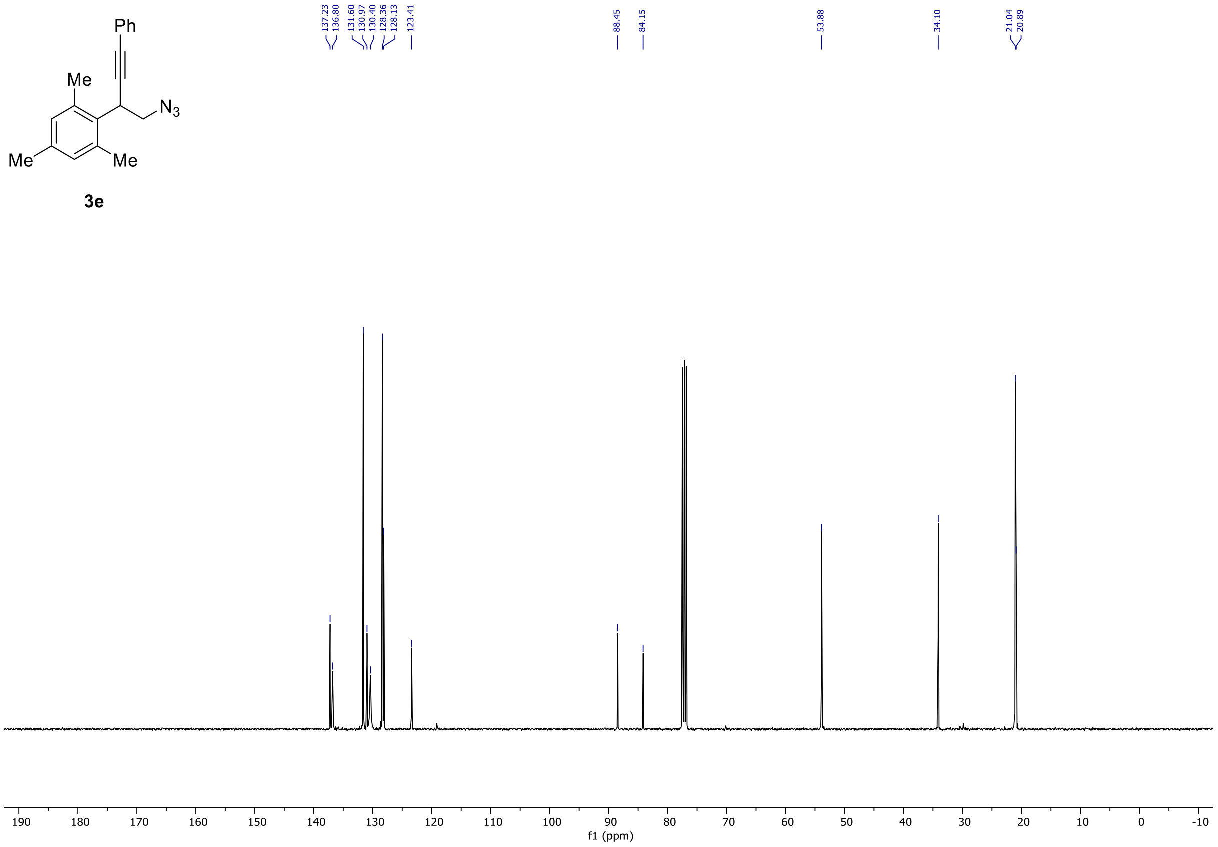

^{1}H NMR (400 MHz, $CDCl_3$) of compound **3f**:

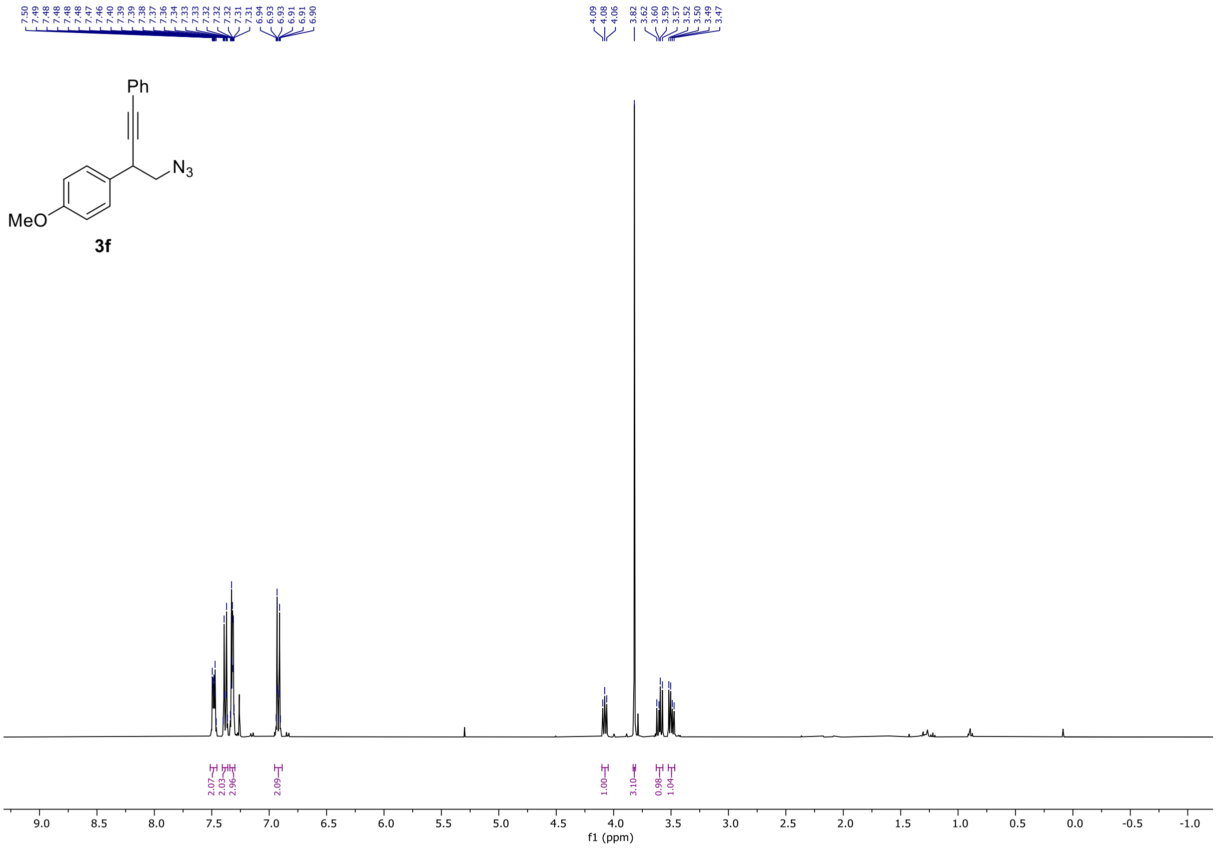

^{13}C NMR (101 MHz, $CDCl_3$) of compound **3f**:

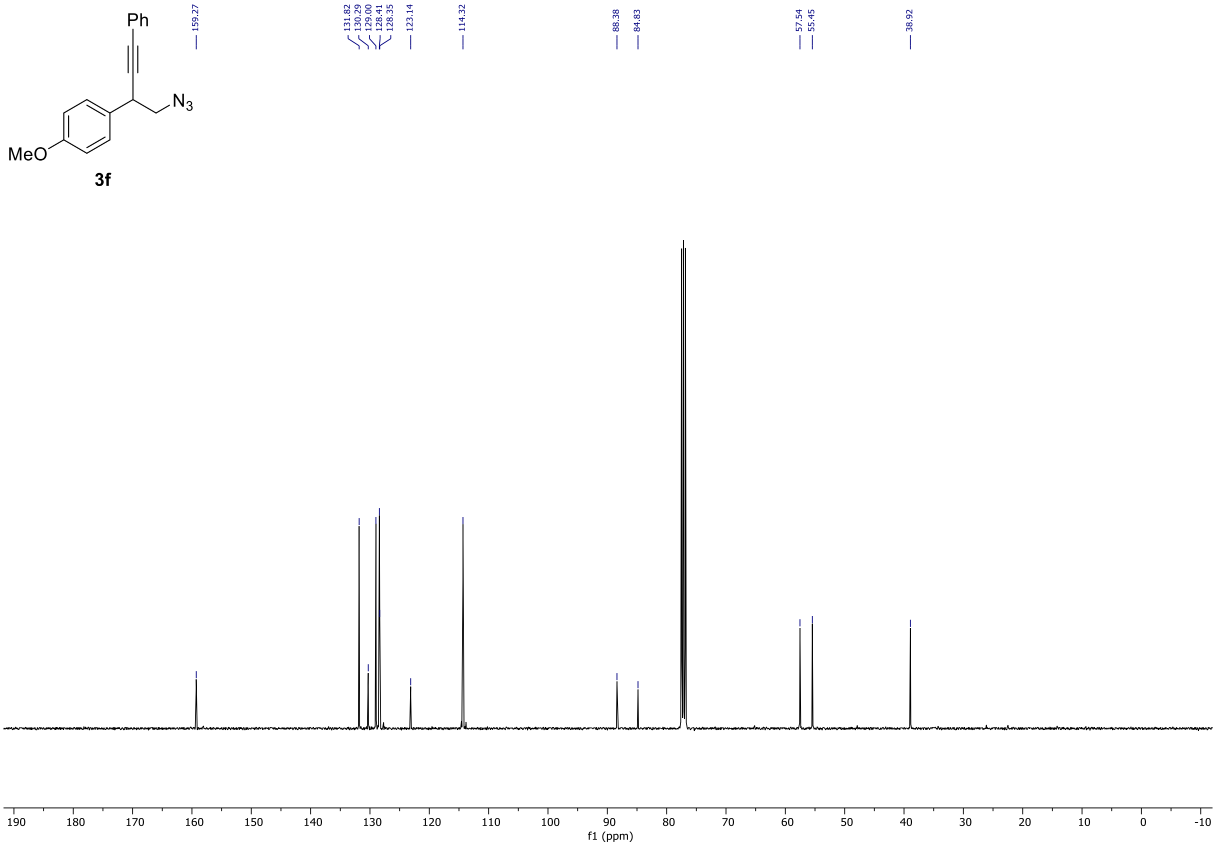

^{1}H NMR (400 MHz, $CDCl_3$) of compound **3g**:

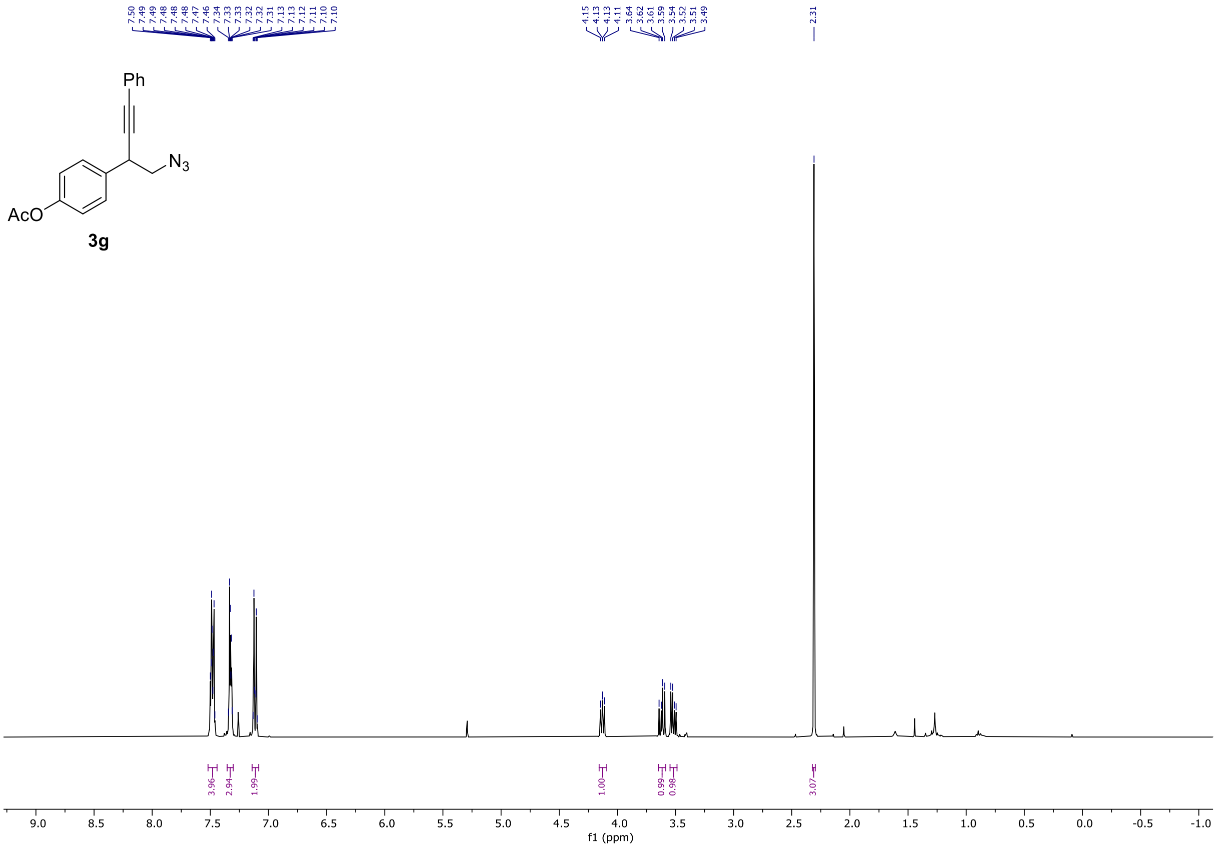

^{13}C NMR (101 MHz, $CDCl_3$) of compound **3g**:

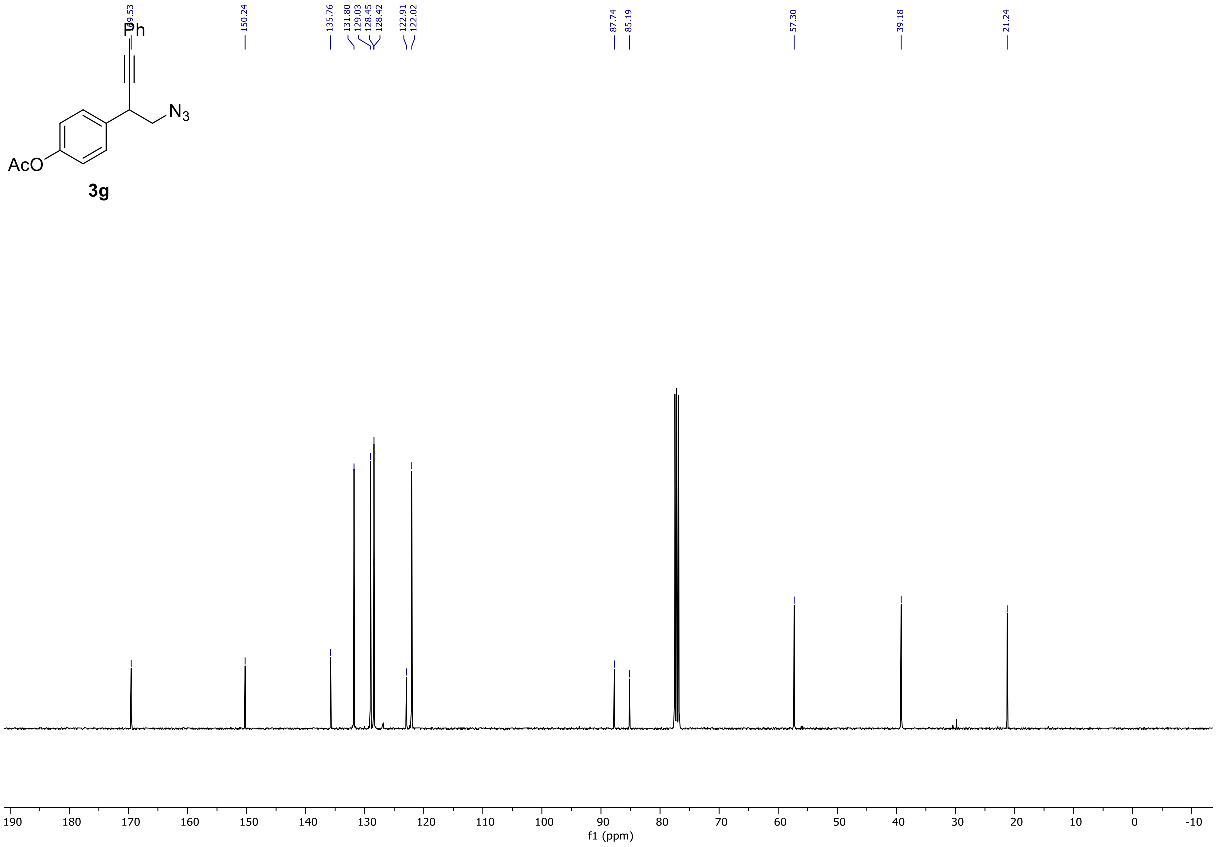

^{1}H NMR (400 MHz, $CDCl_3$) of compound **3h**:

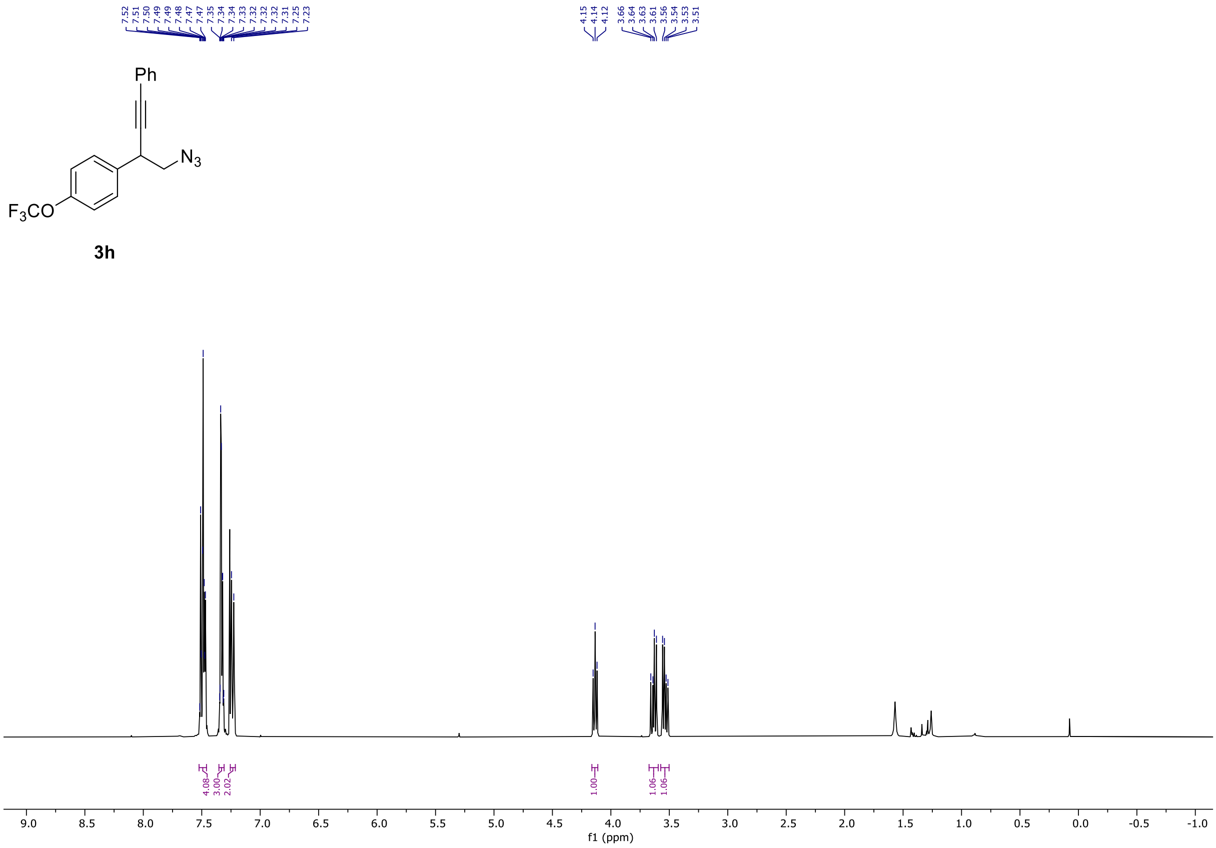

^{13}C NMR (101 MHz, $CDCl_3$) of compound **3h**:

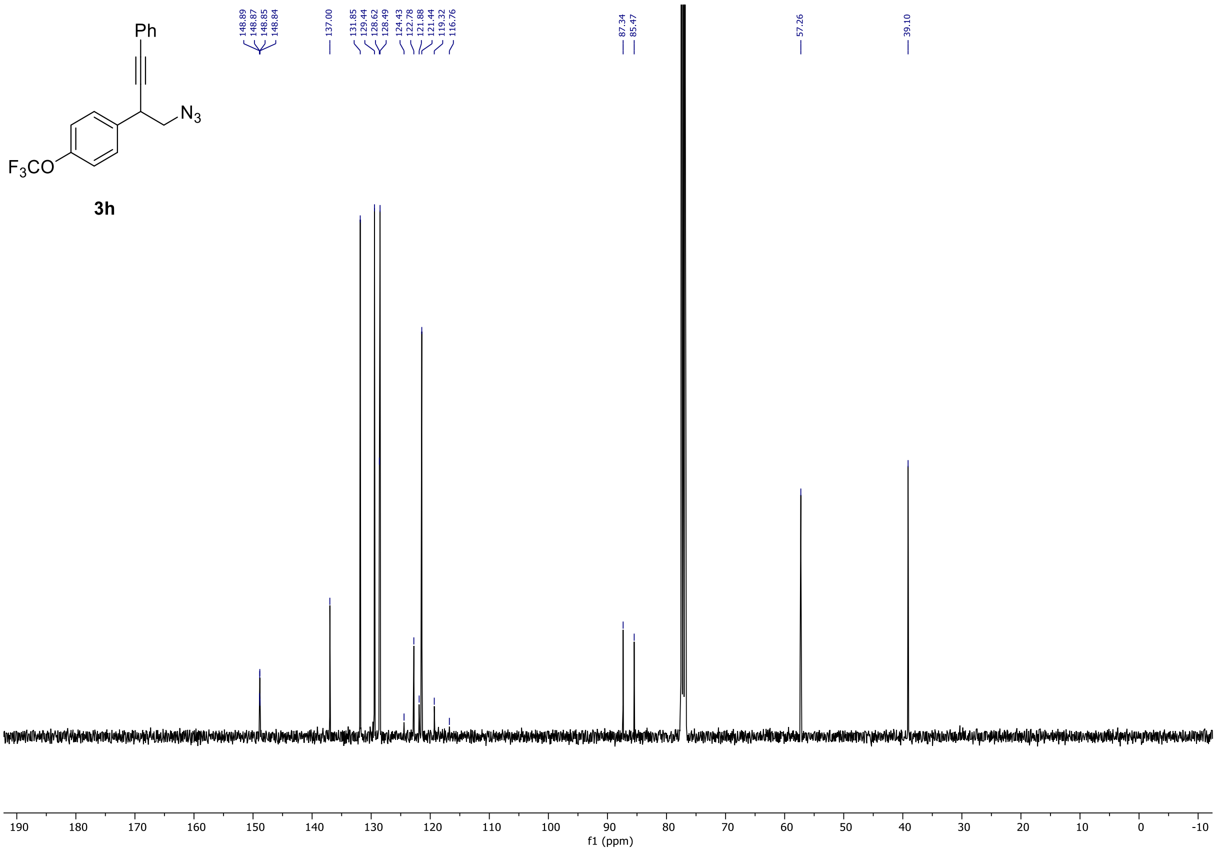

^{19}F NMR (376 MHz, $CDCl_3$) of compound **3h**:

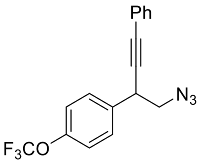

3h

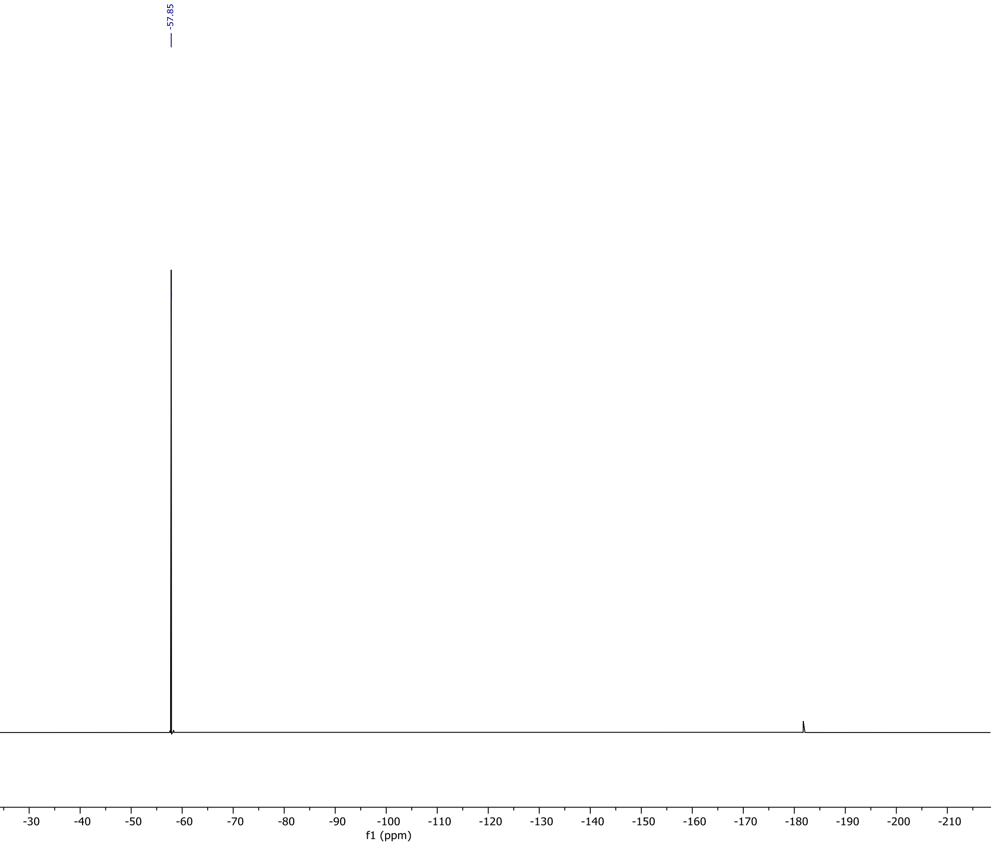

^1H NMR (400 MHz, $CDCl_3$) of compound **3i**:

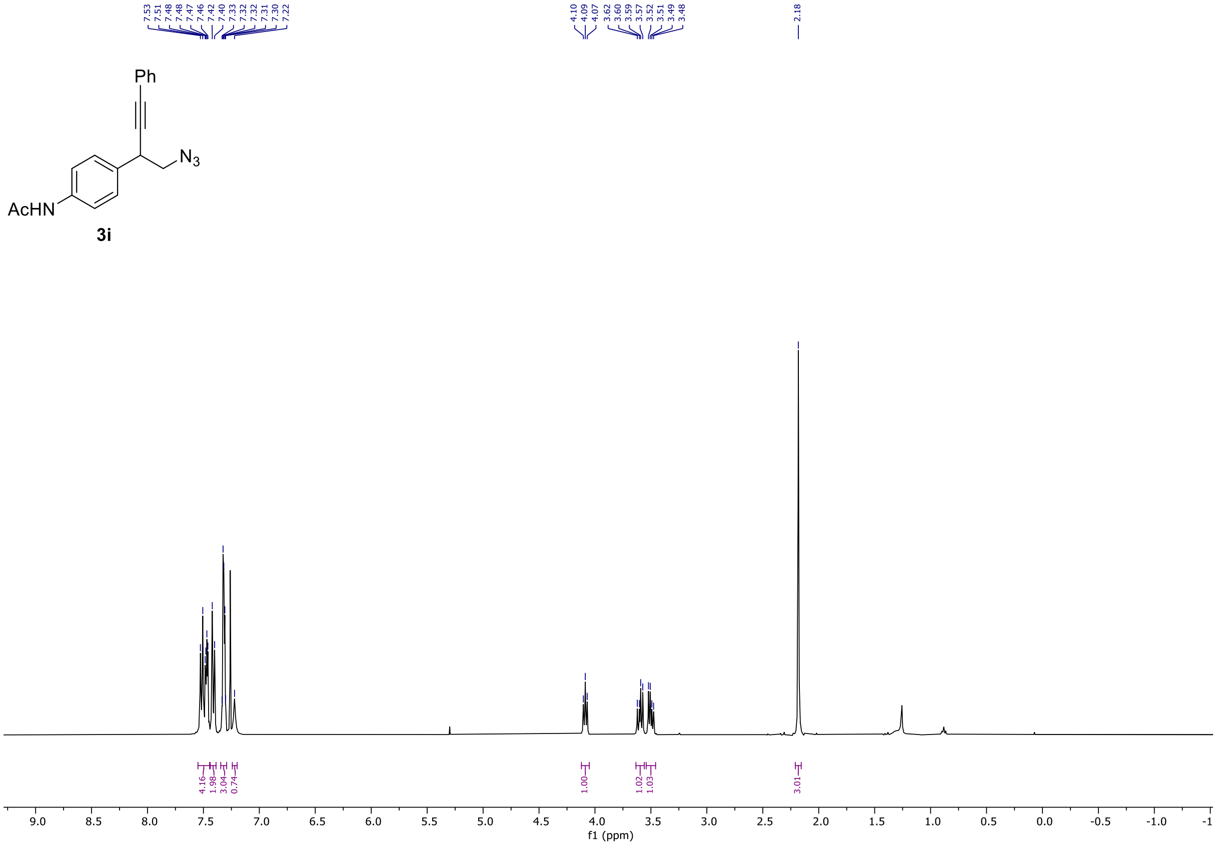

3i

^{13}C NMR (101 MHz, $CDCl_3$) of compound **3i**:

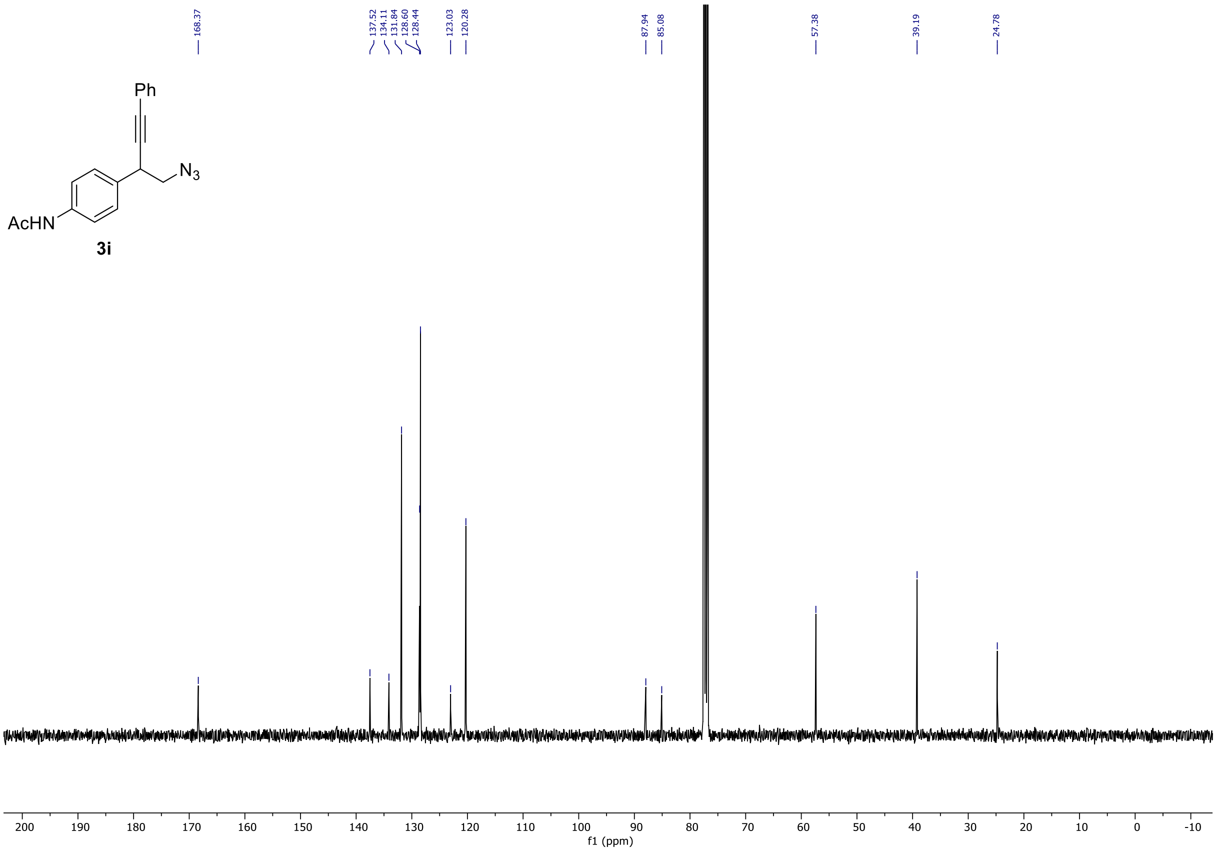

3i

^{1}H NMR (400 MHz, $CDCl_3$) of compound **3j**:

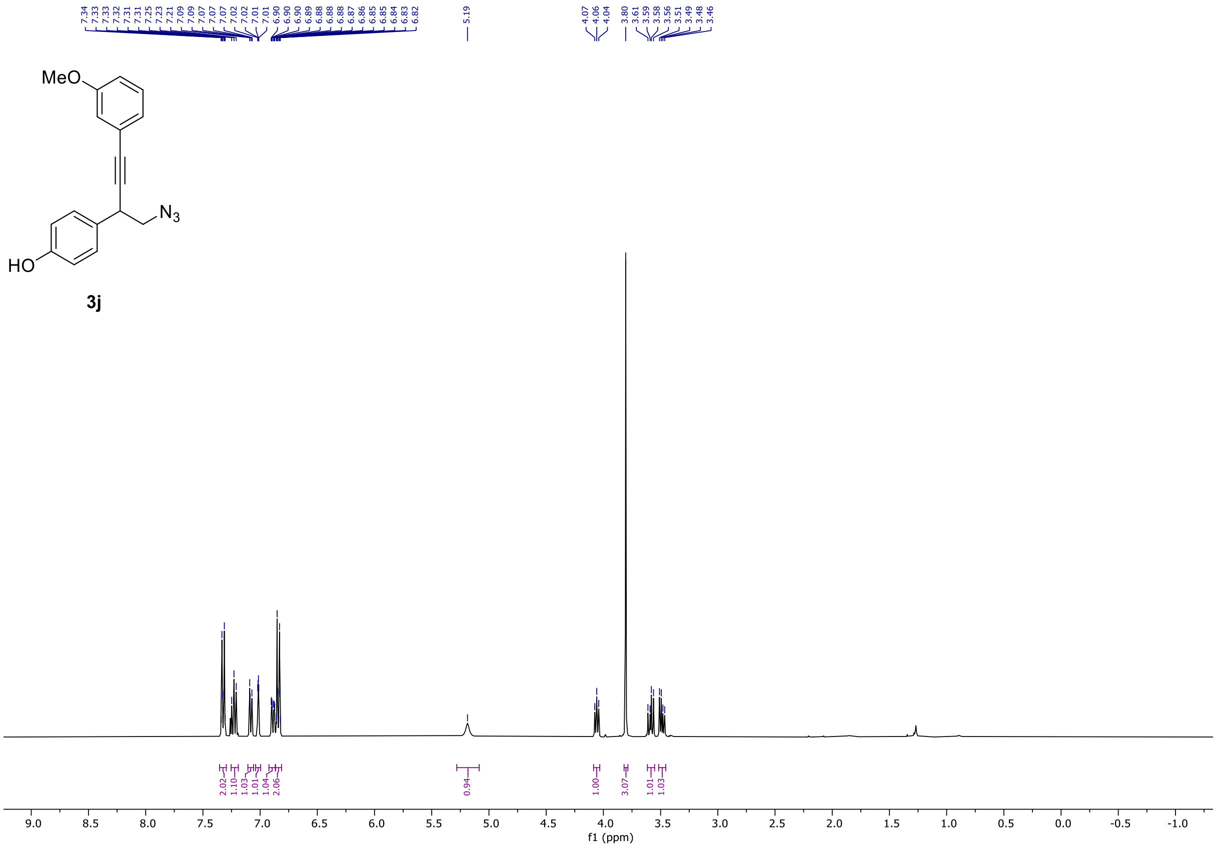

^{13}C NMR (101 MHz, $CDCl_3$) of compound **3j**:

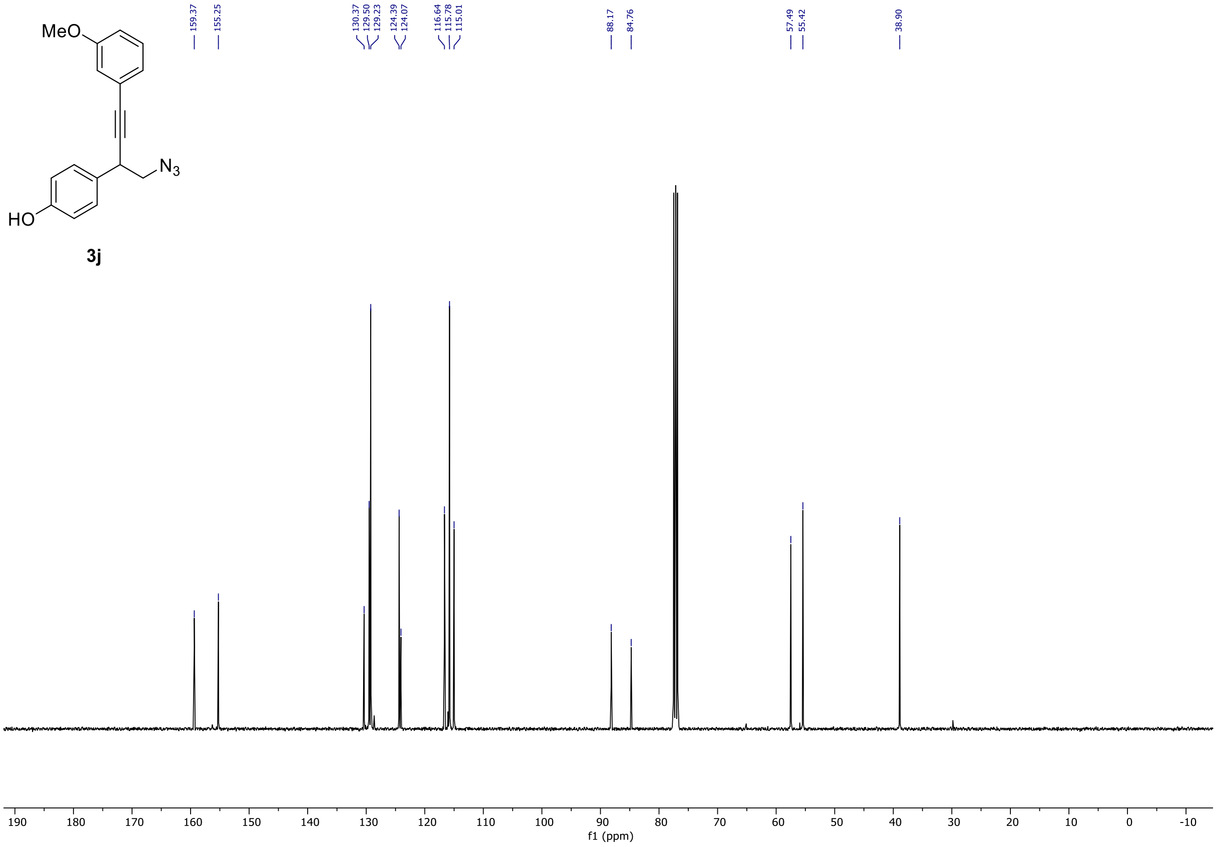

^{1}H NMR (400 MHz, $CDCl_3$) of compound **3k**:

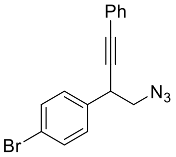

3k

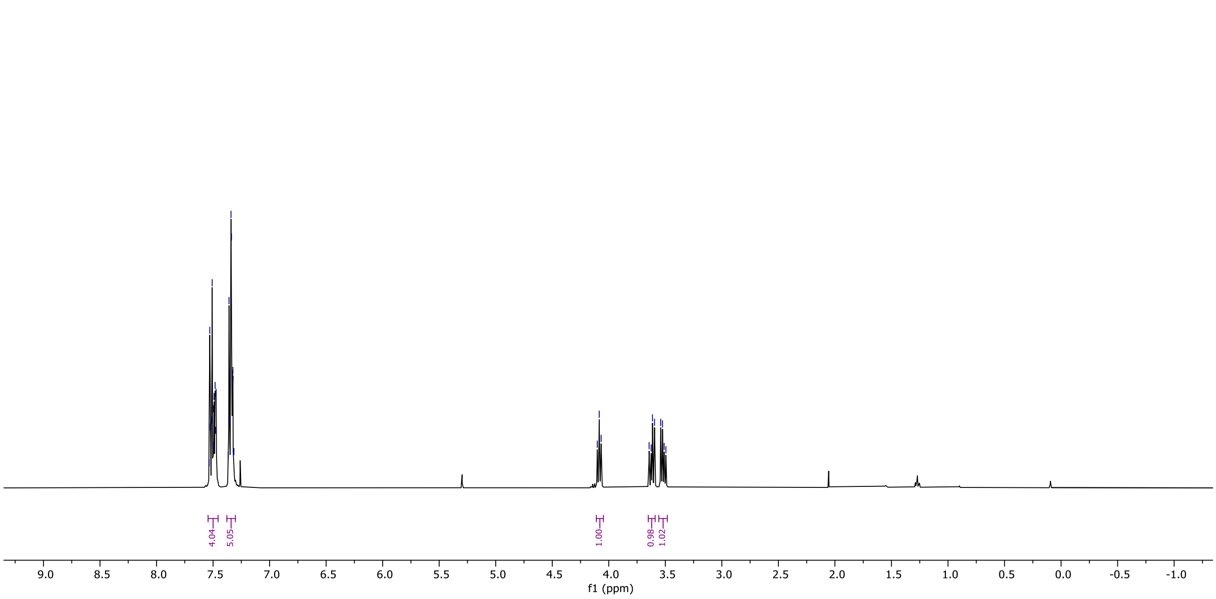

^{13}C NMR (101 MHz, $CDCl_3$) of compound **3k**:

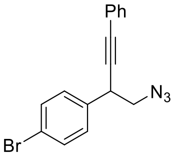

3k

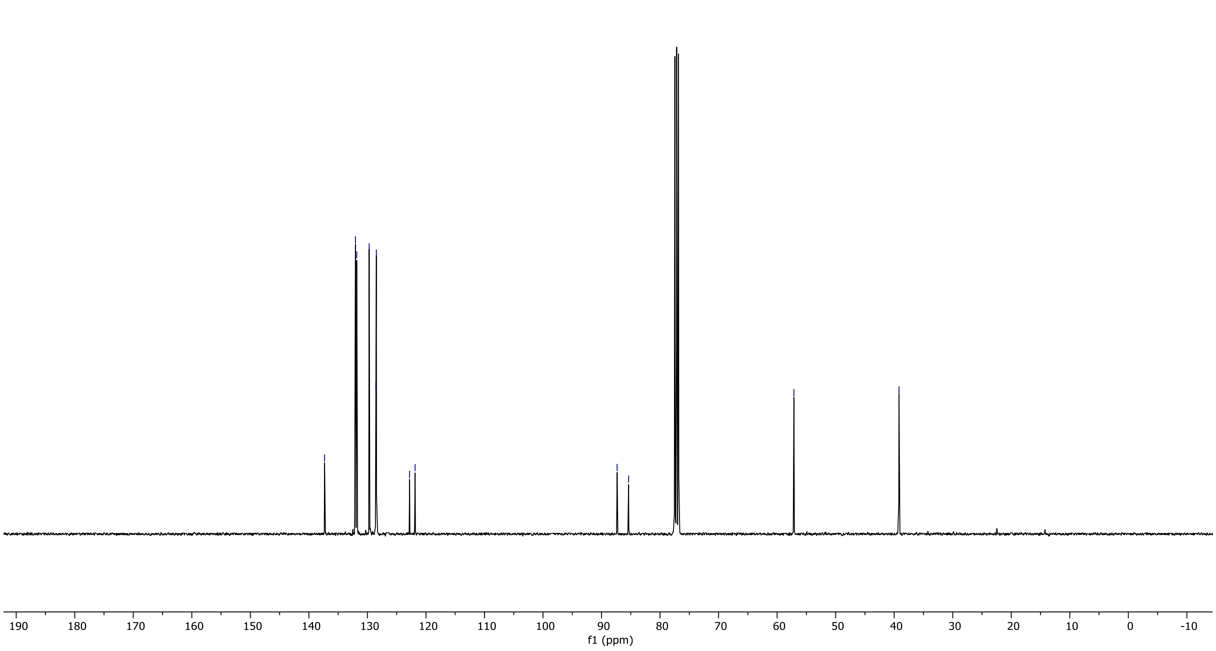

^{1}H NMR (400 MHz, $CDCl_3$) of compound **3l**:

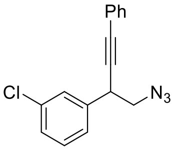

3l

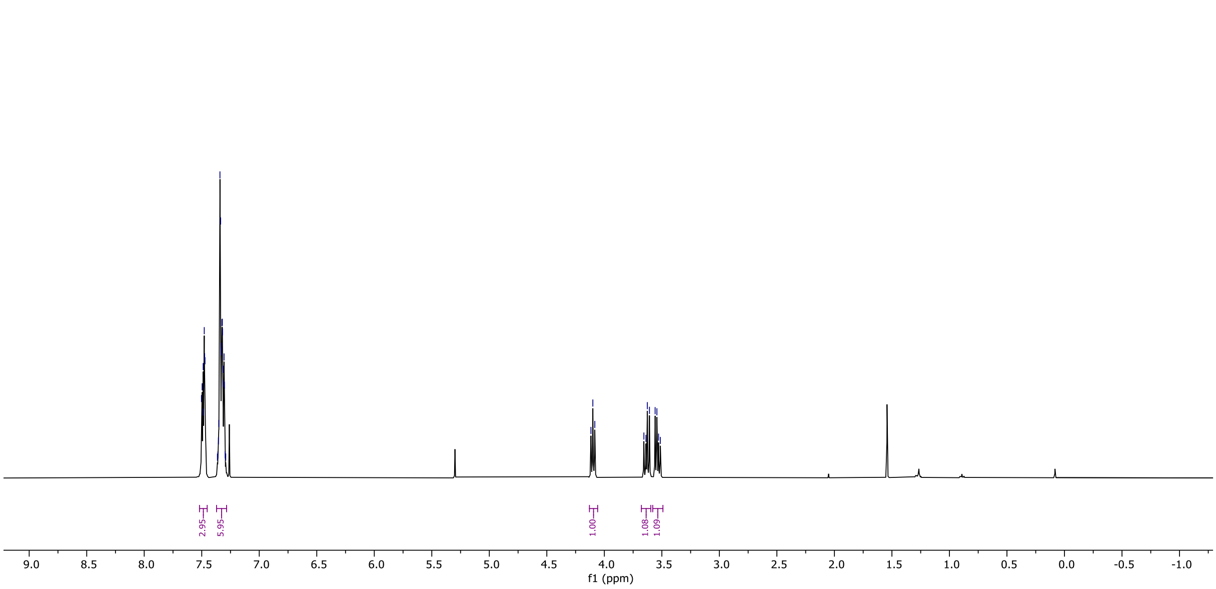

^{13}C NMR (101 MHz, $CDCl_3$) of compound **3l**:

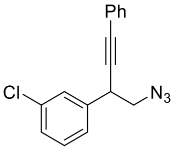

3l

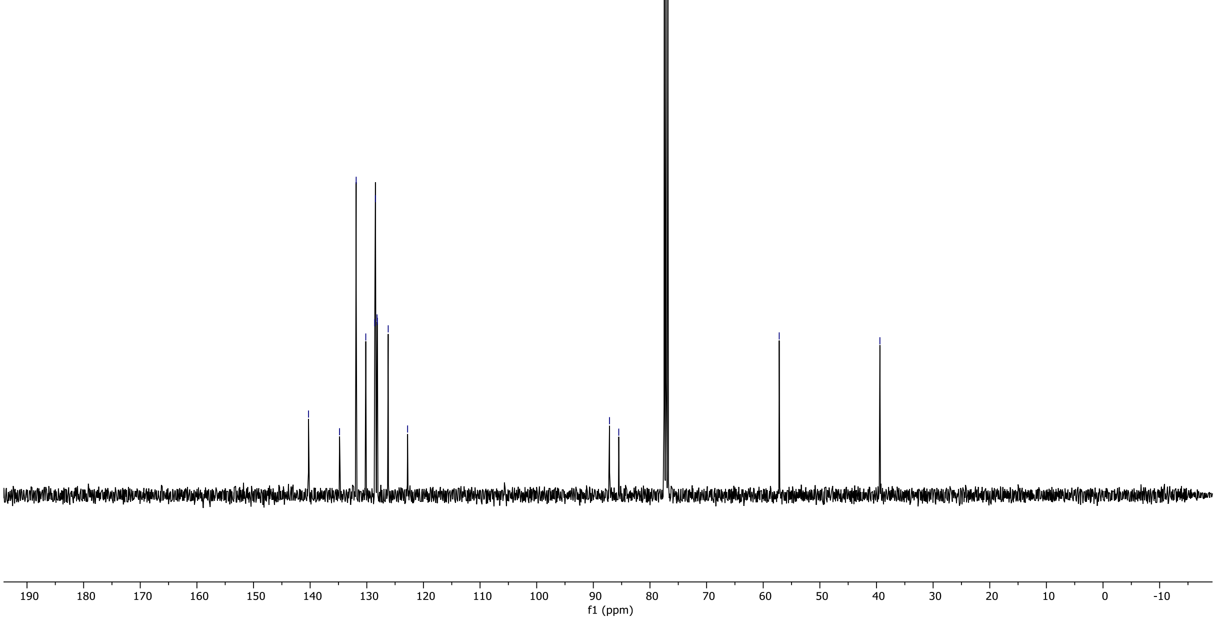

^{1}H NMR (400 MHz, $CDCl_3$) of compound **3m**:

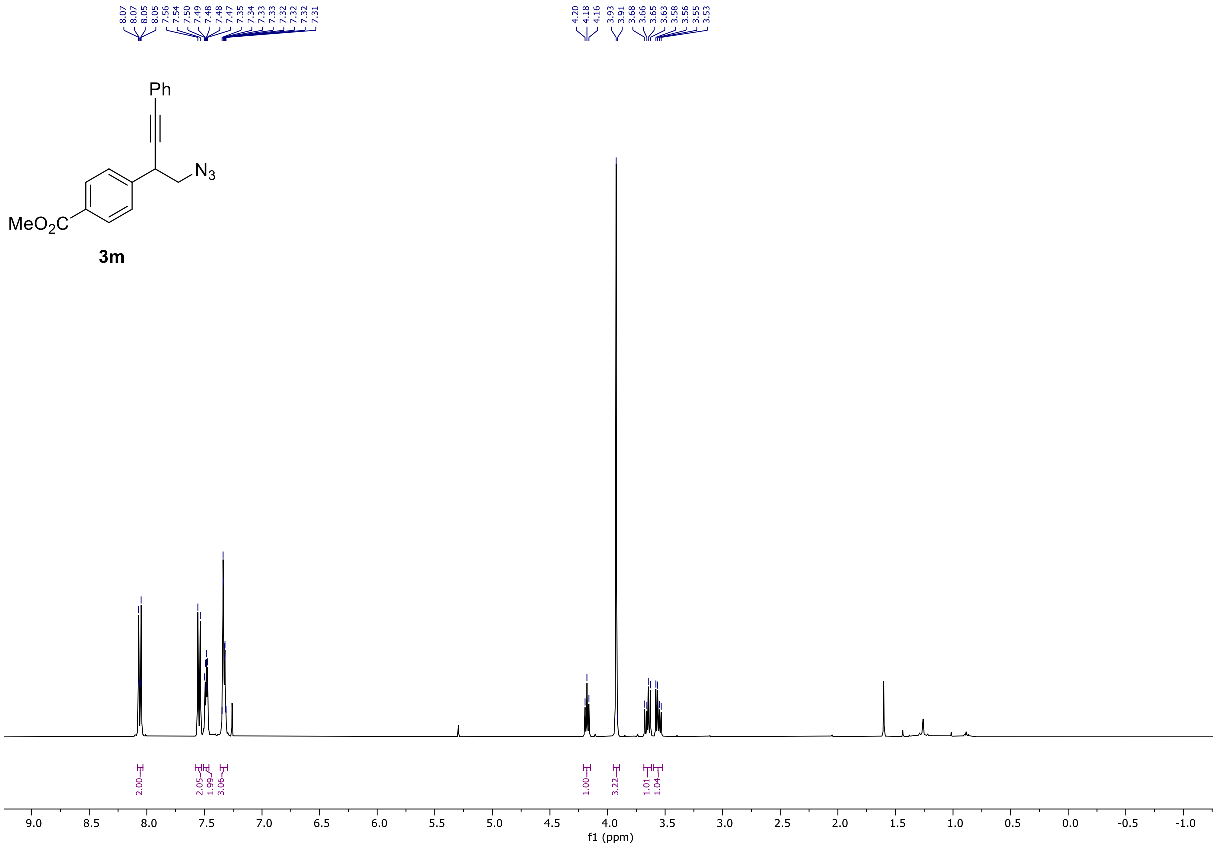

^{13}C NMR (101 MHz, $CDCl_3$) of compound **3m**:

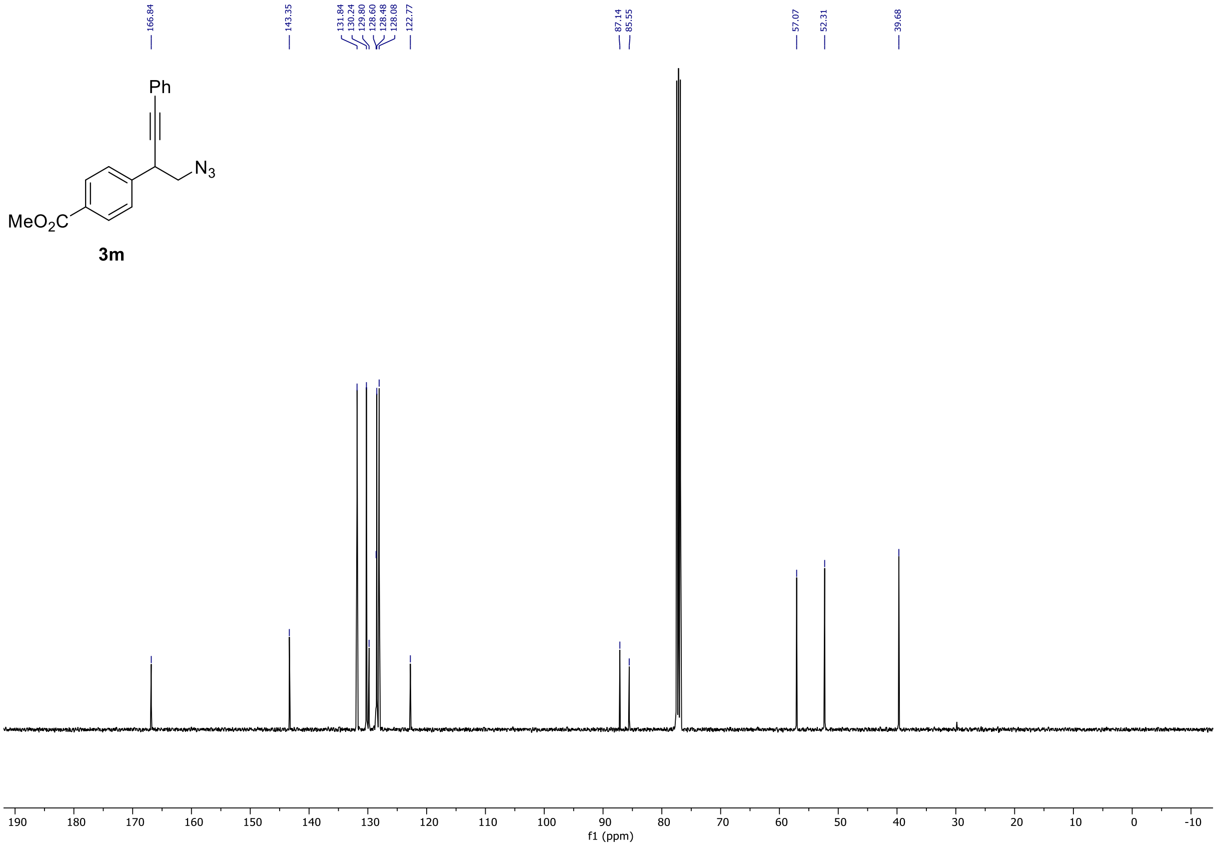

^{1}H NMR (400 MHz, $CDCl_3$) of compound **3n**:

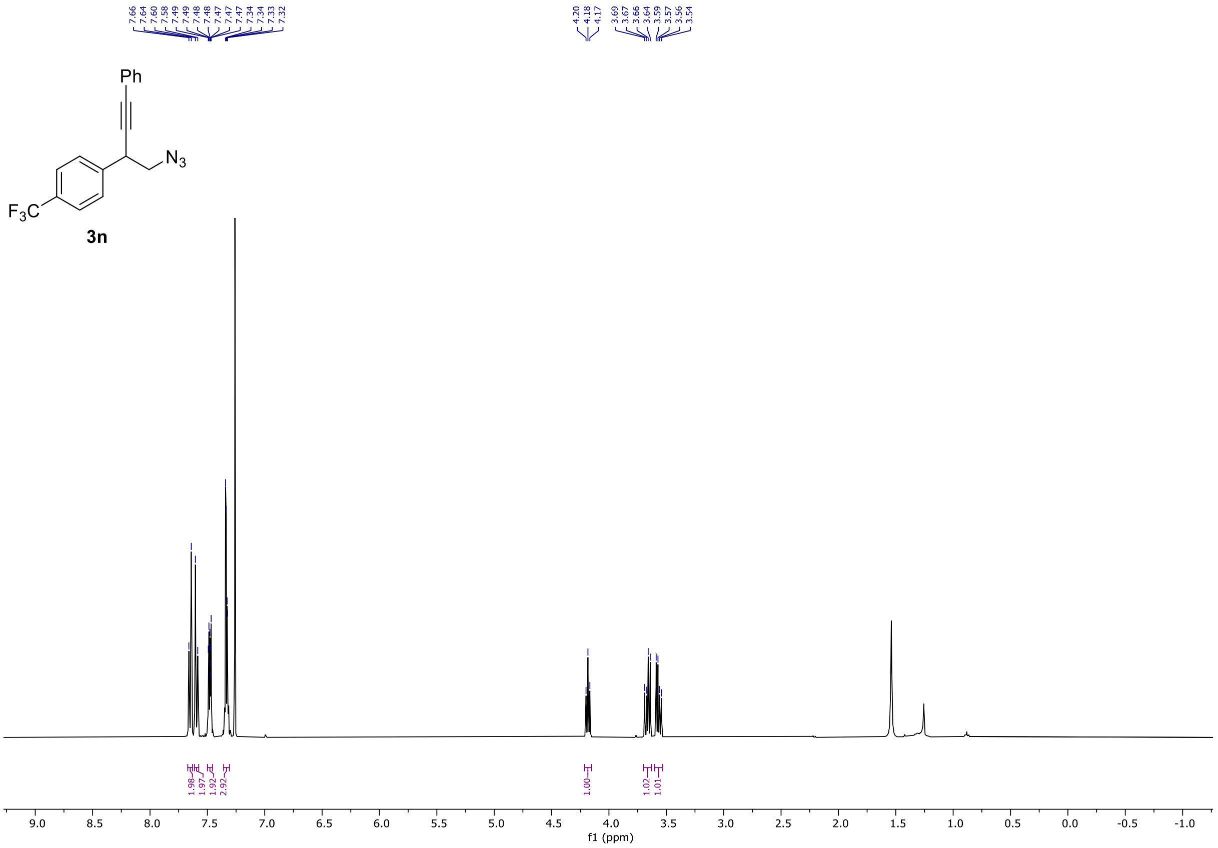

^{13}C NMR (101 MHz, $CDCl_3$) of compound **3n**:

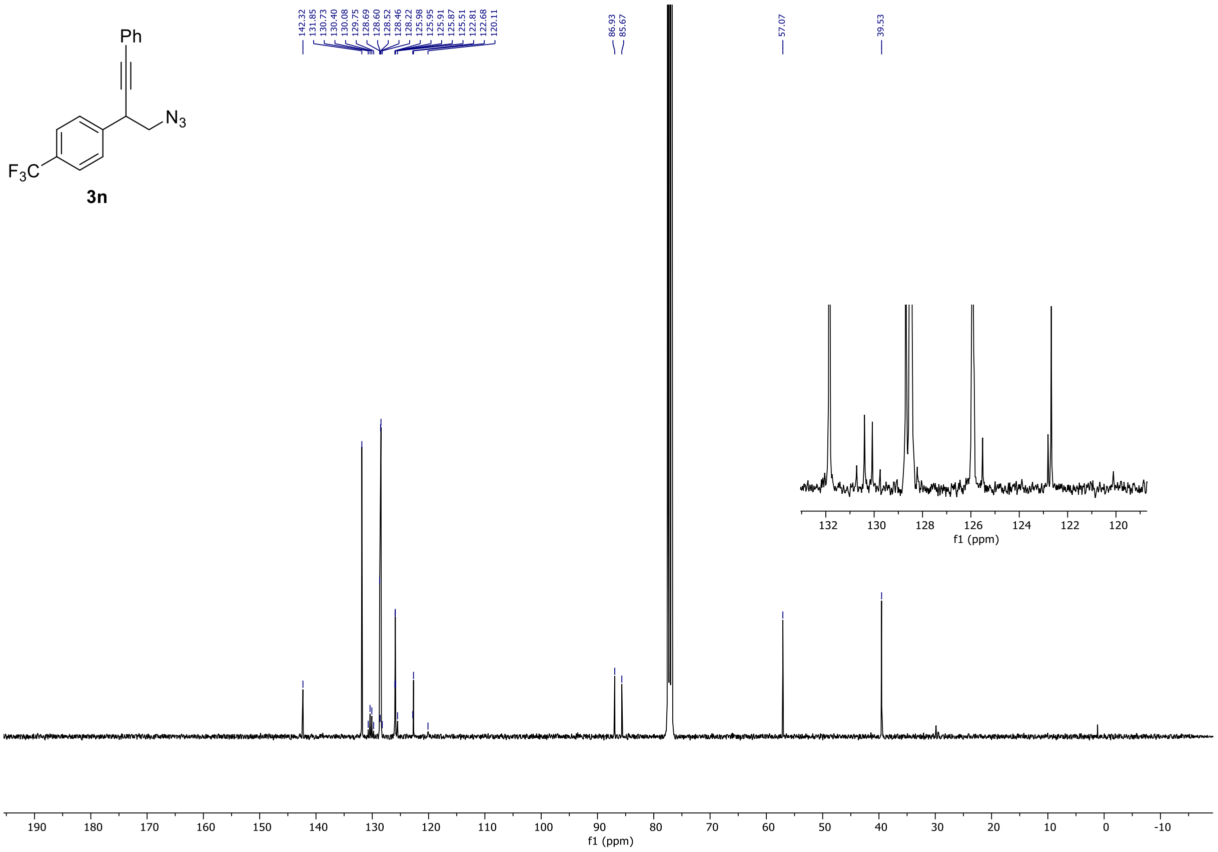

^{19}F NMR (376 MHz, $CDCl_3$) of compound **3n**:

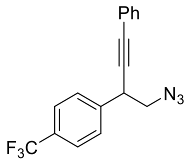

3n

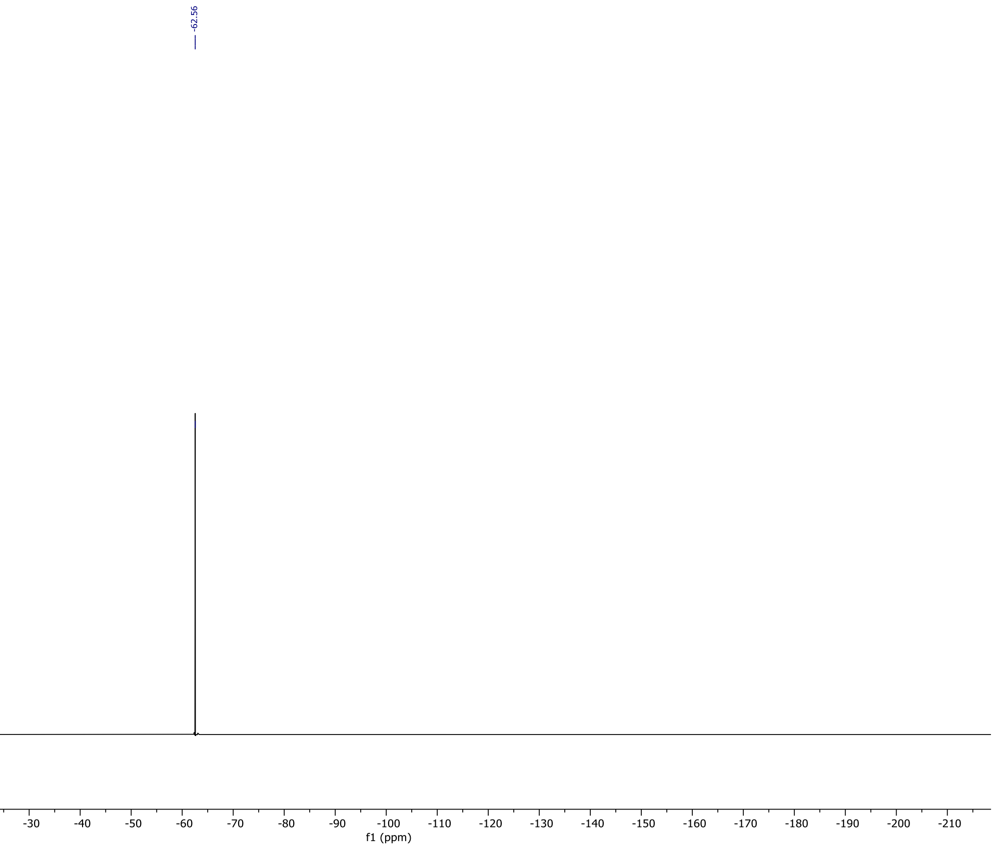

^{1}H NMR (400 MHz, $CDCl_3$) of compound **3o**:

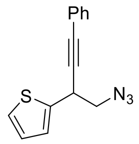

3o

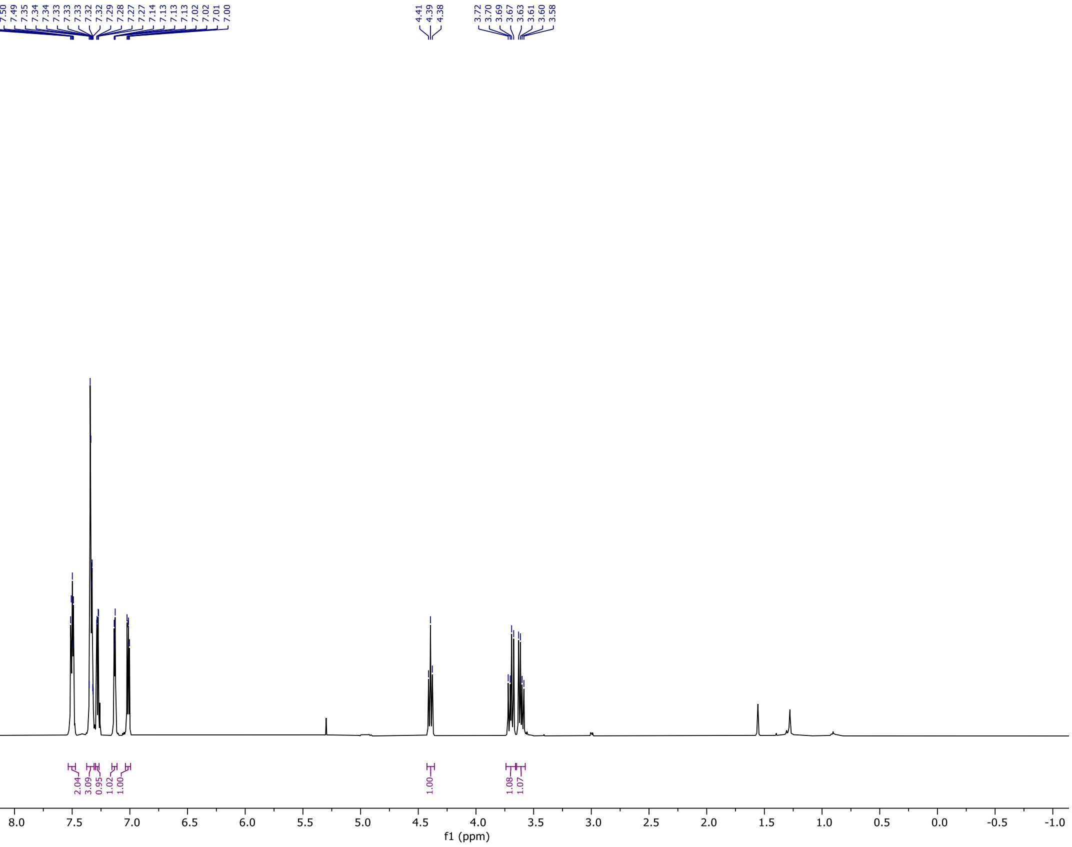

^{13}C NMR (101 MHz, $CDCl_3$) of compound **3o**:

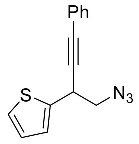

3o

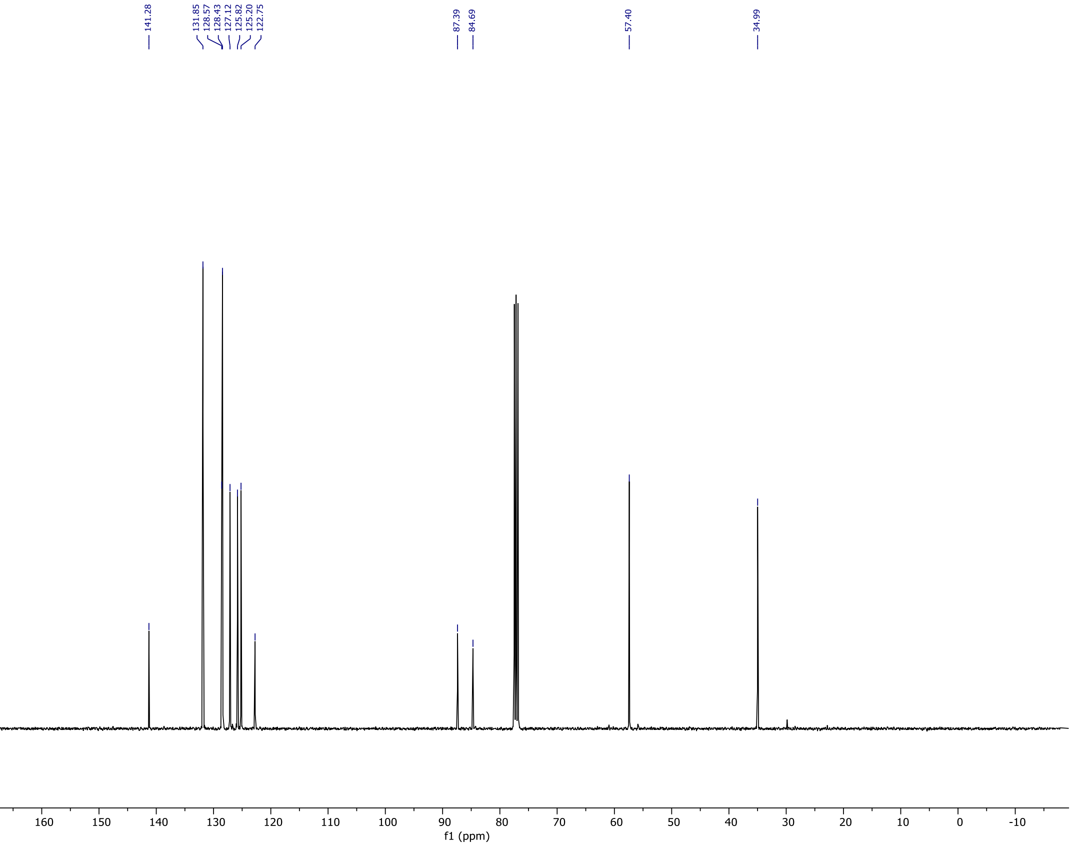

^{1}H NMR (400 MHz, $CDCl_3$) of compound **3p**:

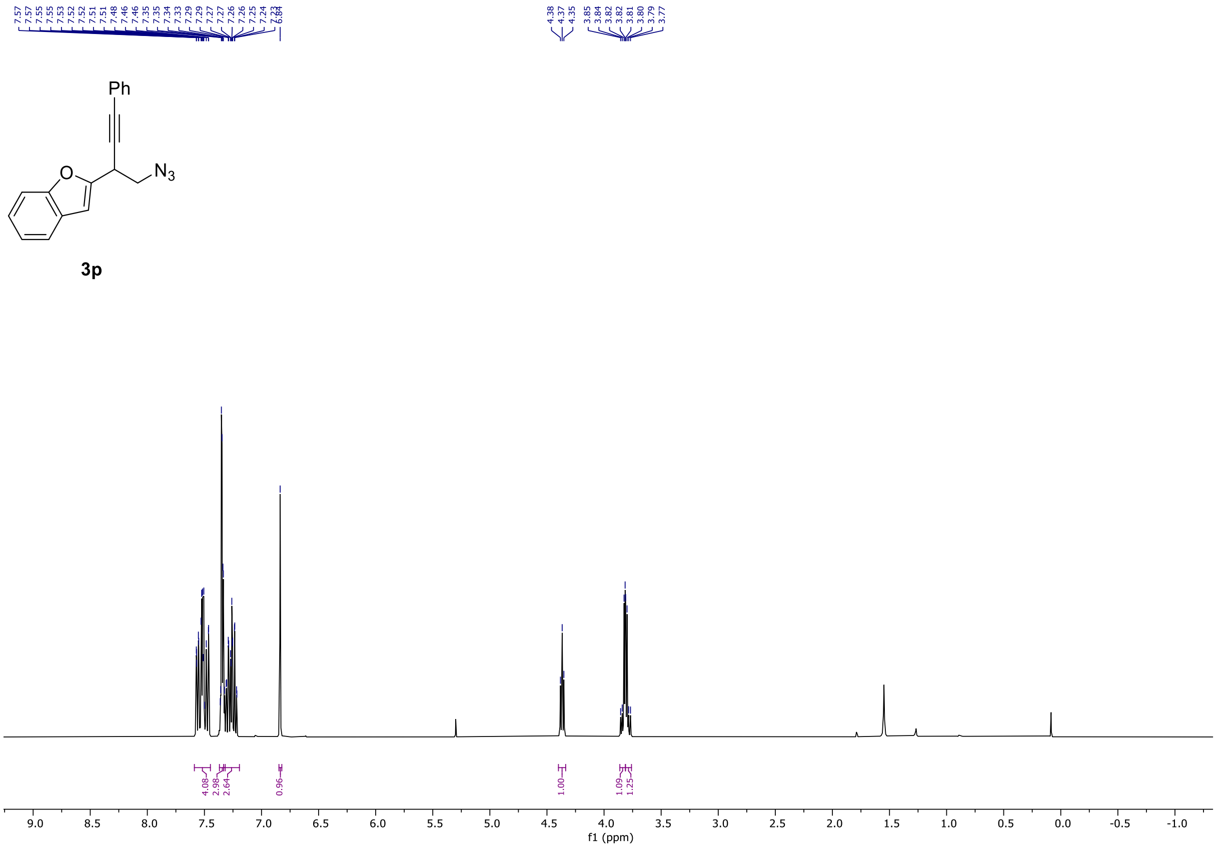

^{13}C NMR (101 MHz, $CDCl_3$) of compound **3p**:

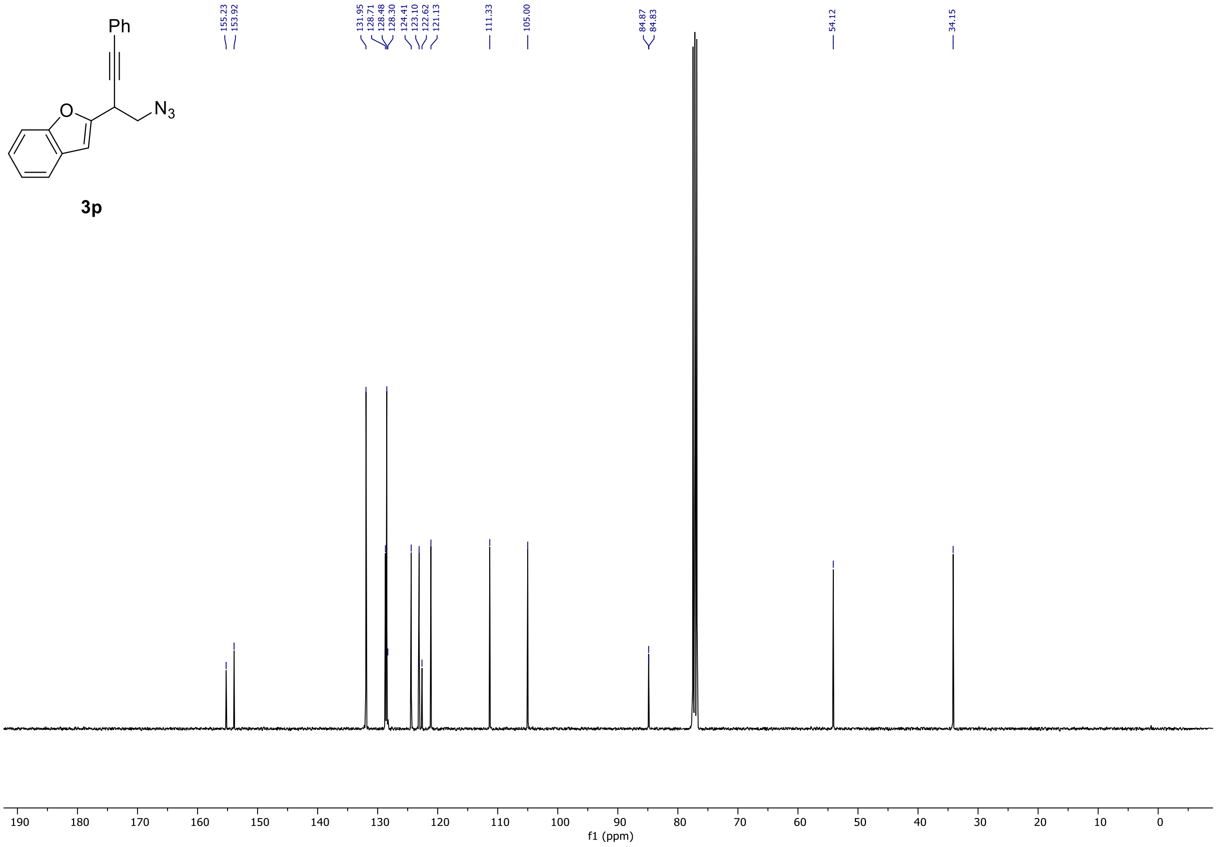

^{1}H NMR (400 MHz, $CDCl_3$) of compound **3q**:

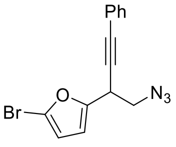

3q

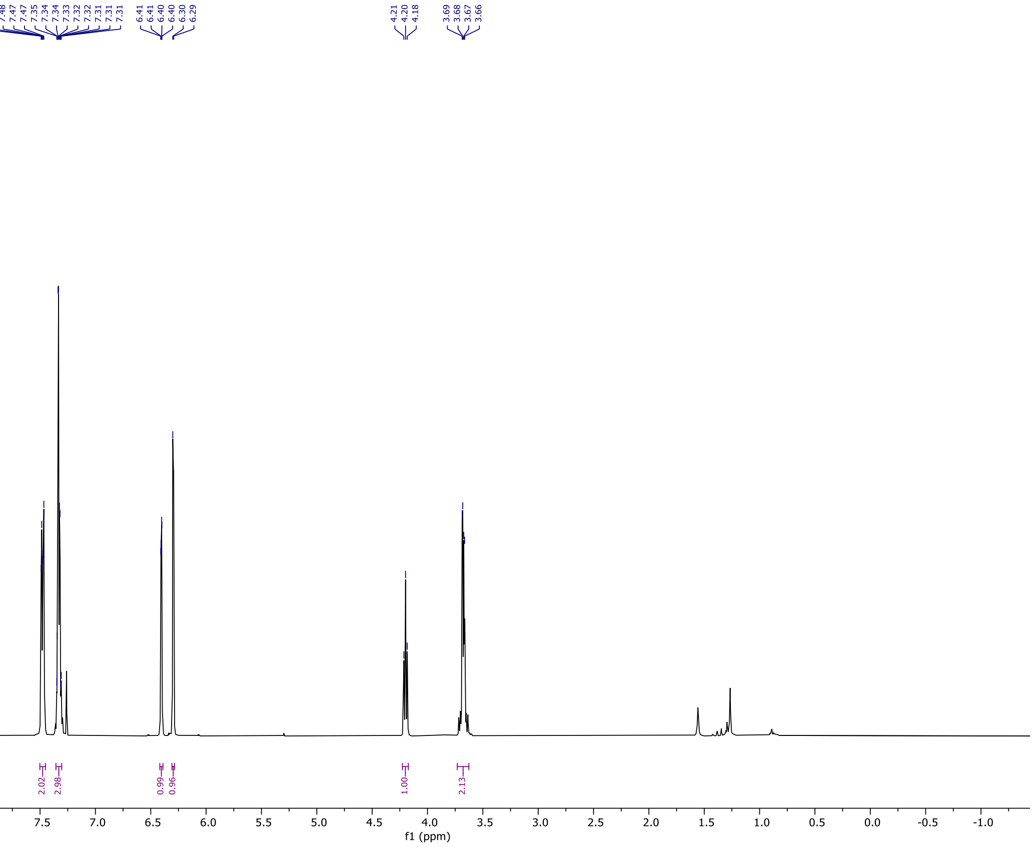

^{13}C NMR (101 MHz, $CDCl_3$) of compound **3q**:

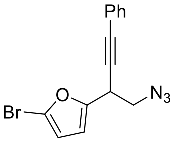

3q

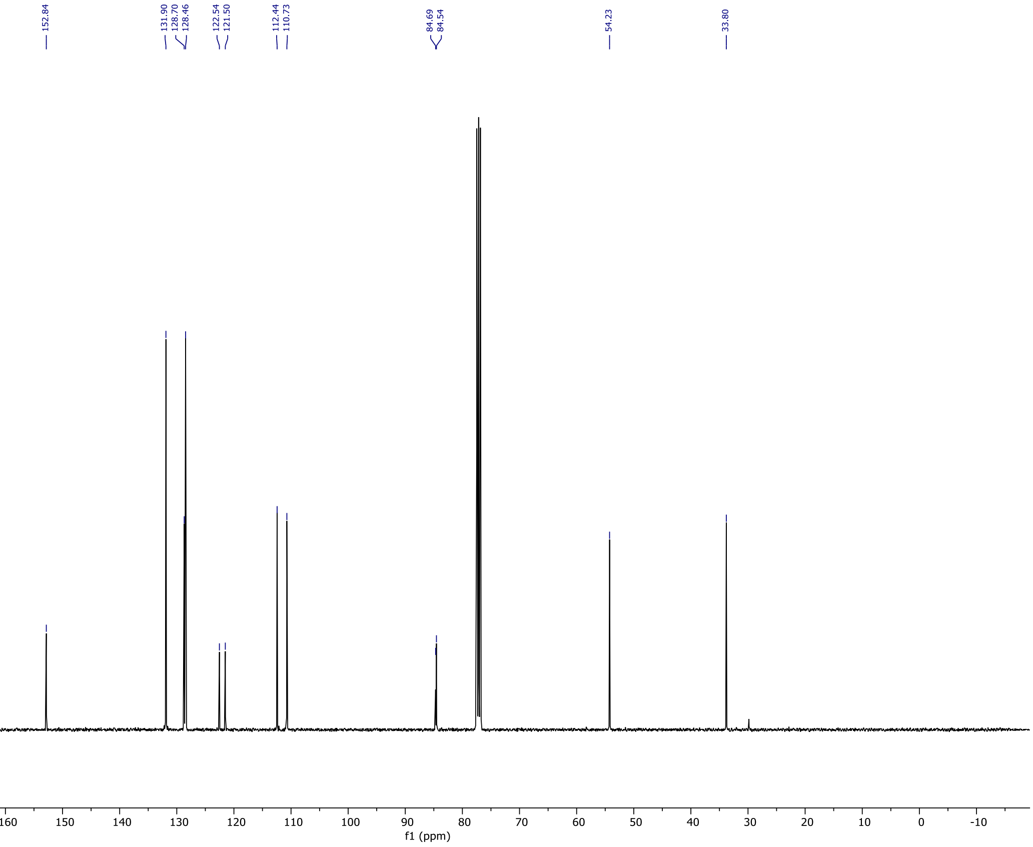

^{1}H NMR (400 MHz, $CDCl_3$) of compound **3r**:

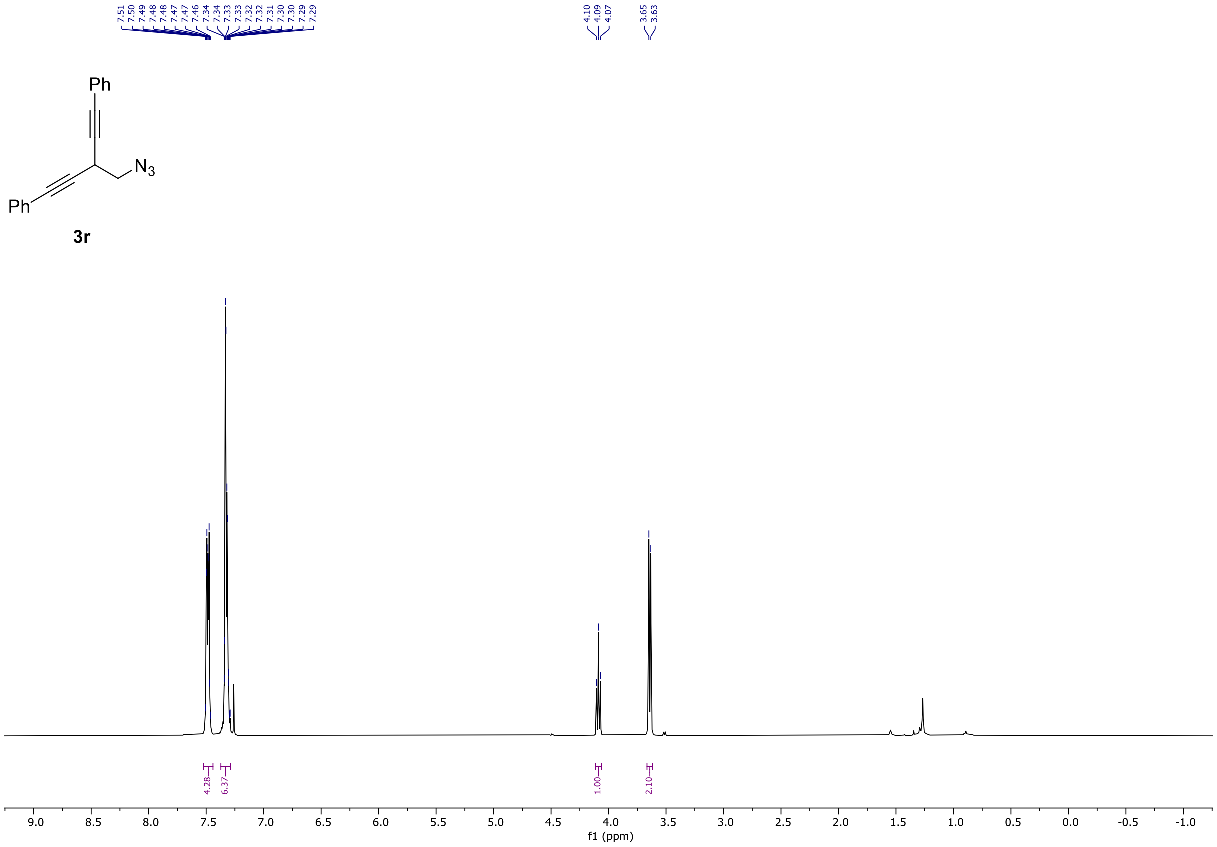

^{13}C NMR (101 MHz, $CDCl_3$) of compound **3r**:

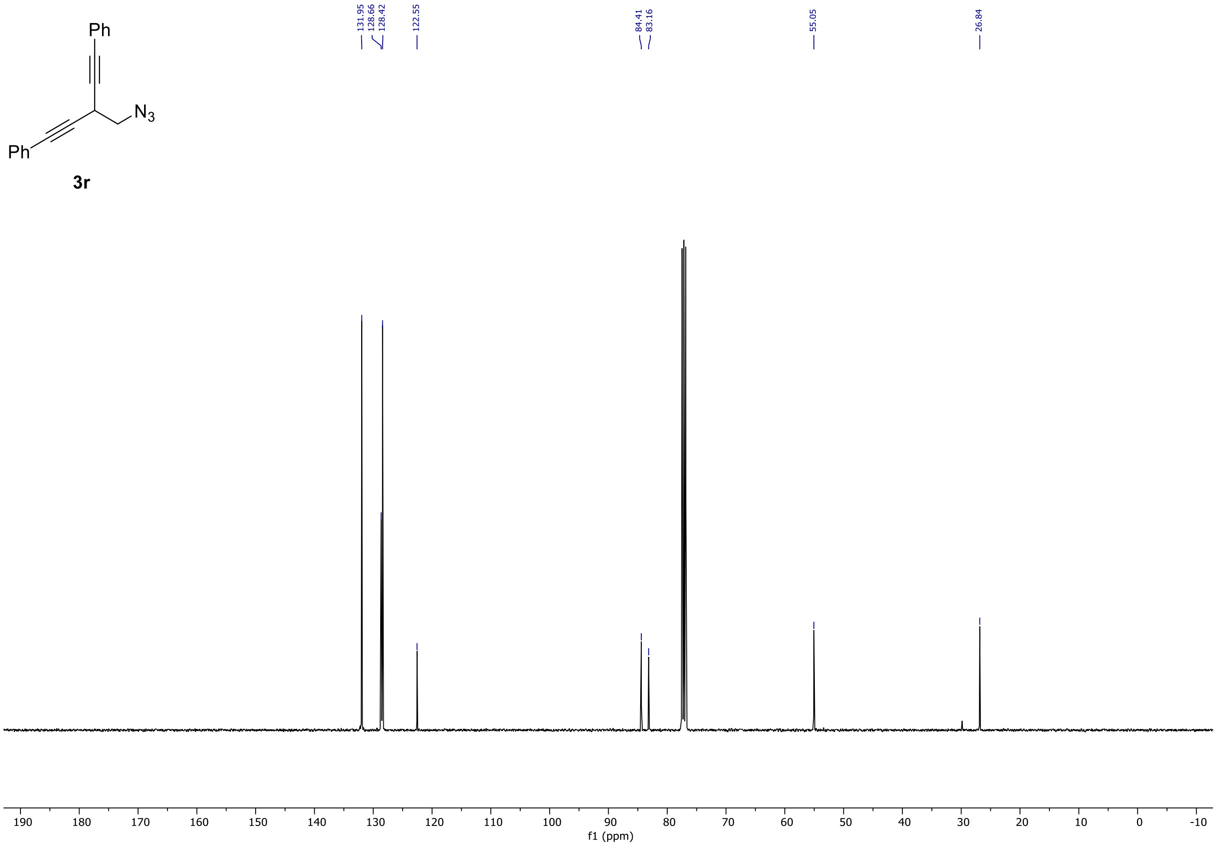

^{1}H NMR (400 MHz, $CDCl_3$) of crude compound **3s** (dr determination):

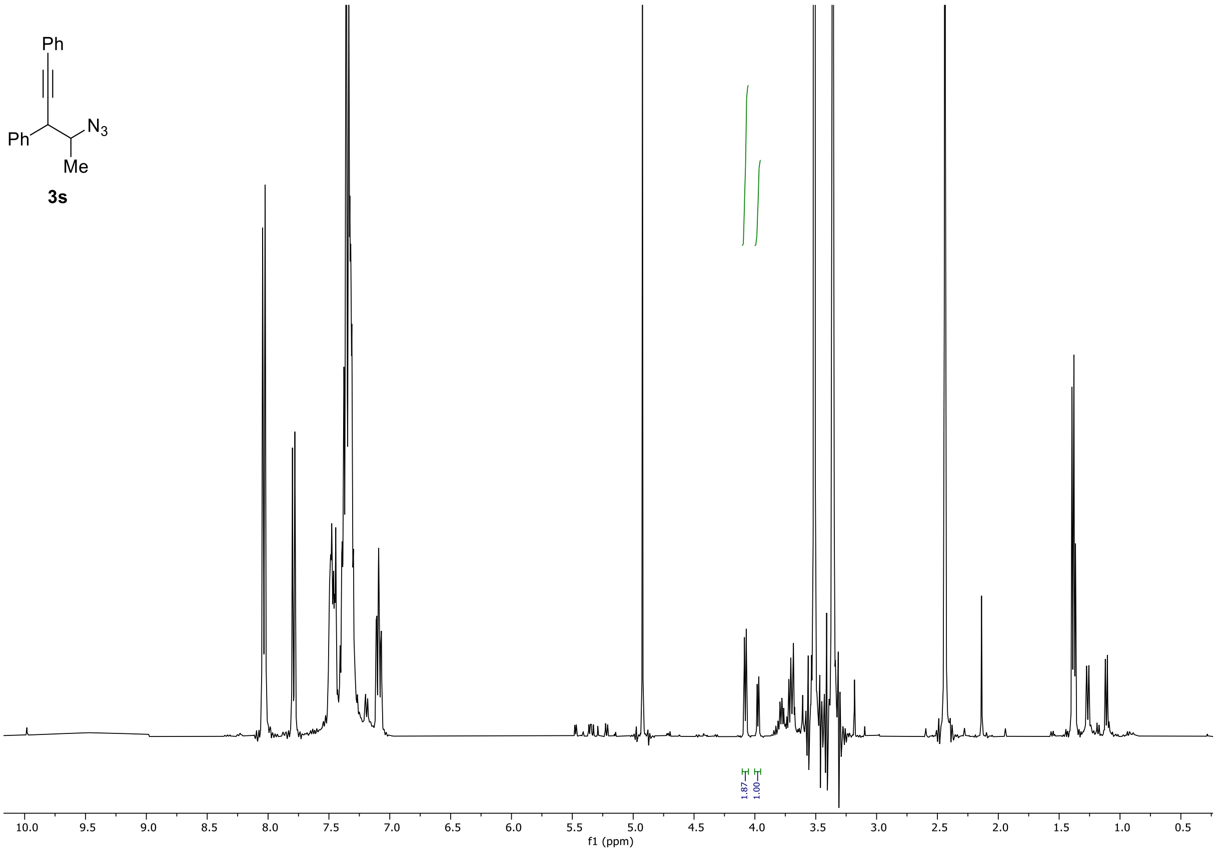

^{1}H NMR (400 MHz, $CDCl_3$) of compound **3s**:

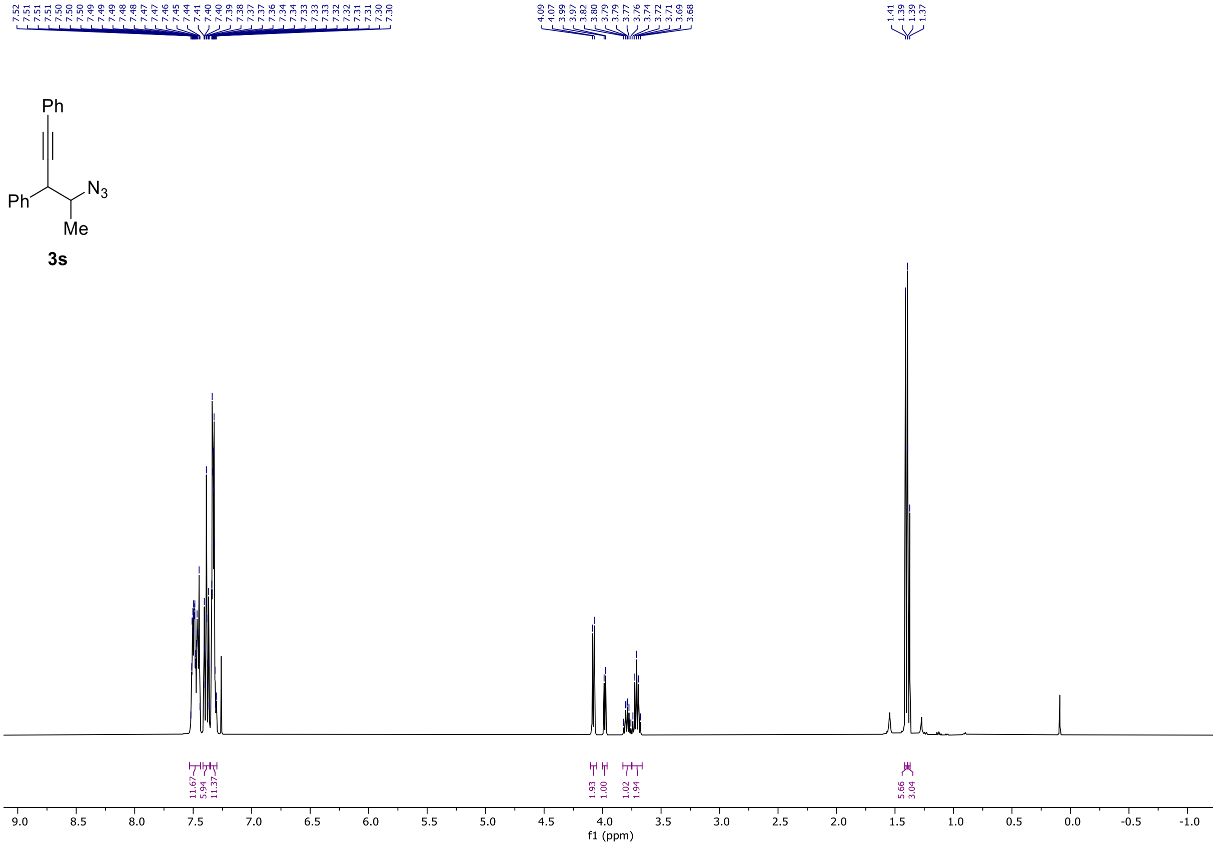

^{13}C NMR (101 MHz, $CDCl_3$) of compound **3s**:

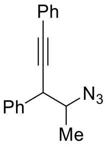

3s

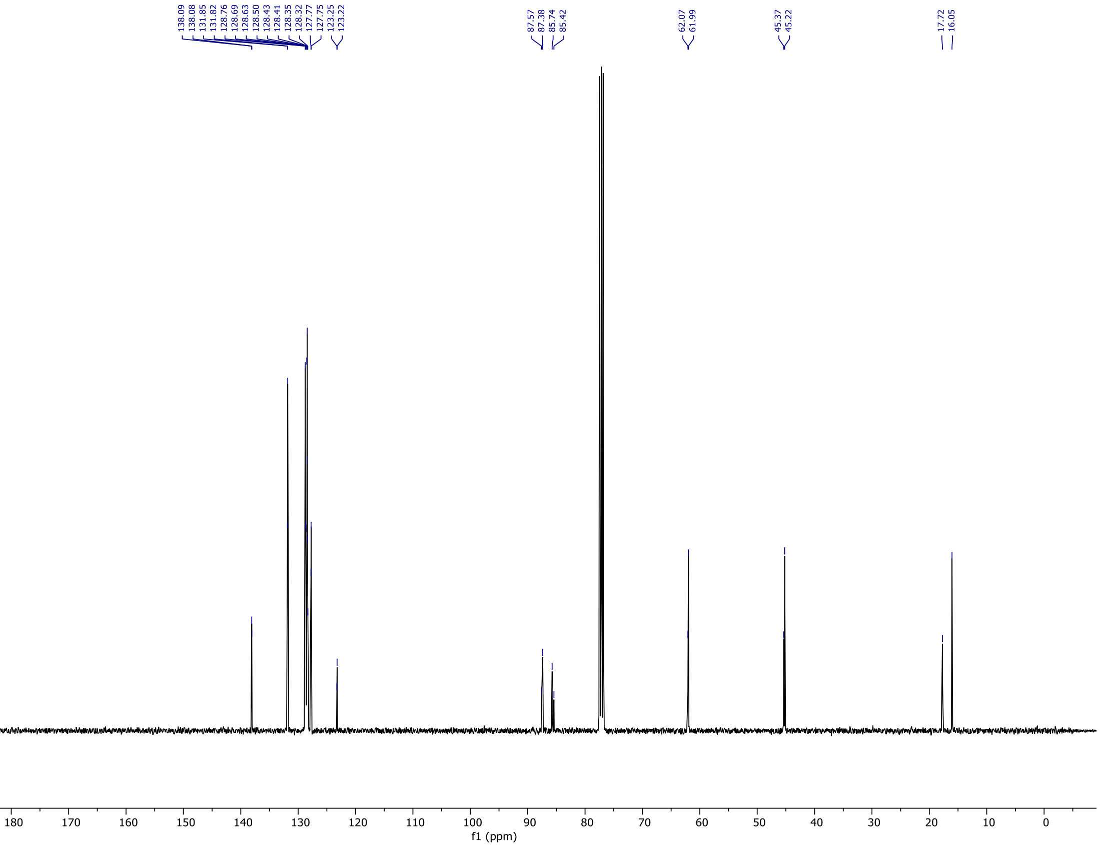

^{1}H NMR (400 MHz, $CDCl_3$) of compound **3t/3t’** (yield/dr determination):

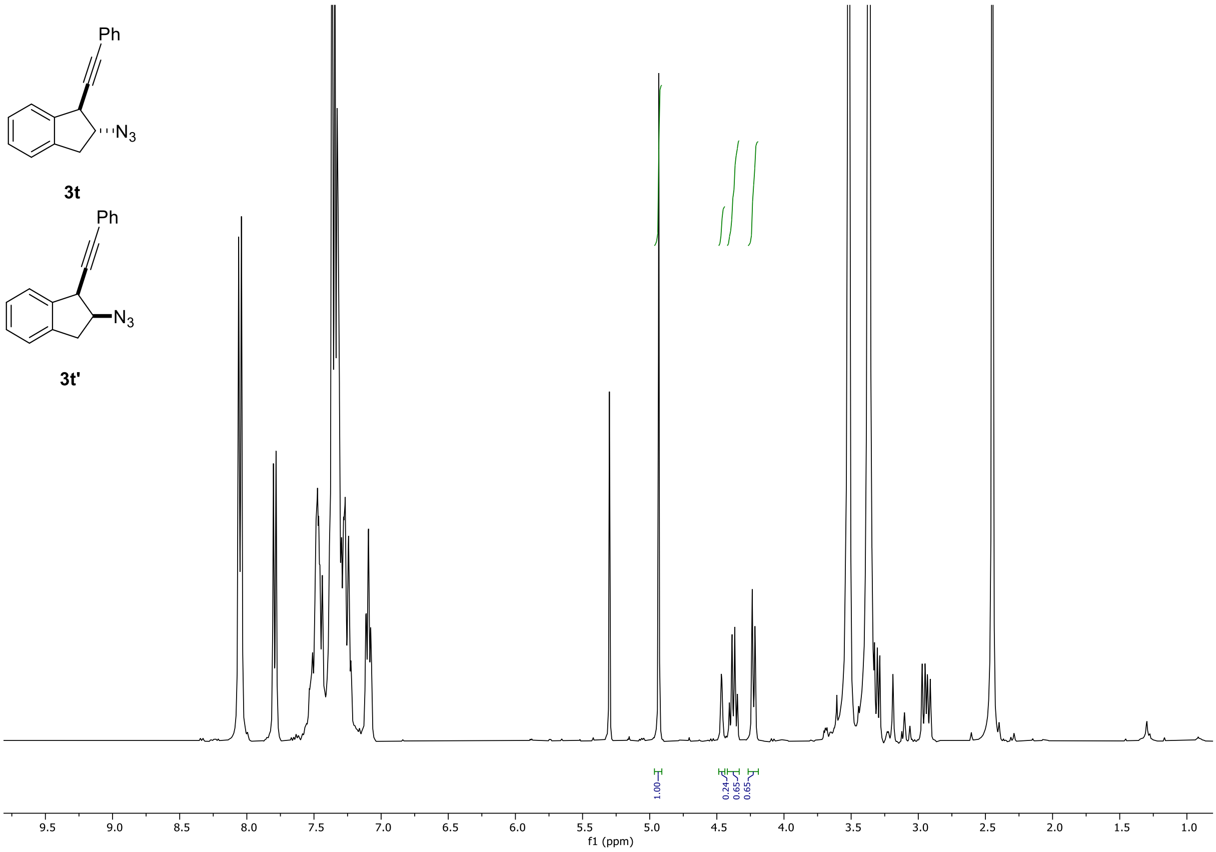

^{1}H NMR (400 MHz, $CDCl_3$) of compound **3t**:

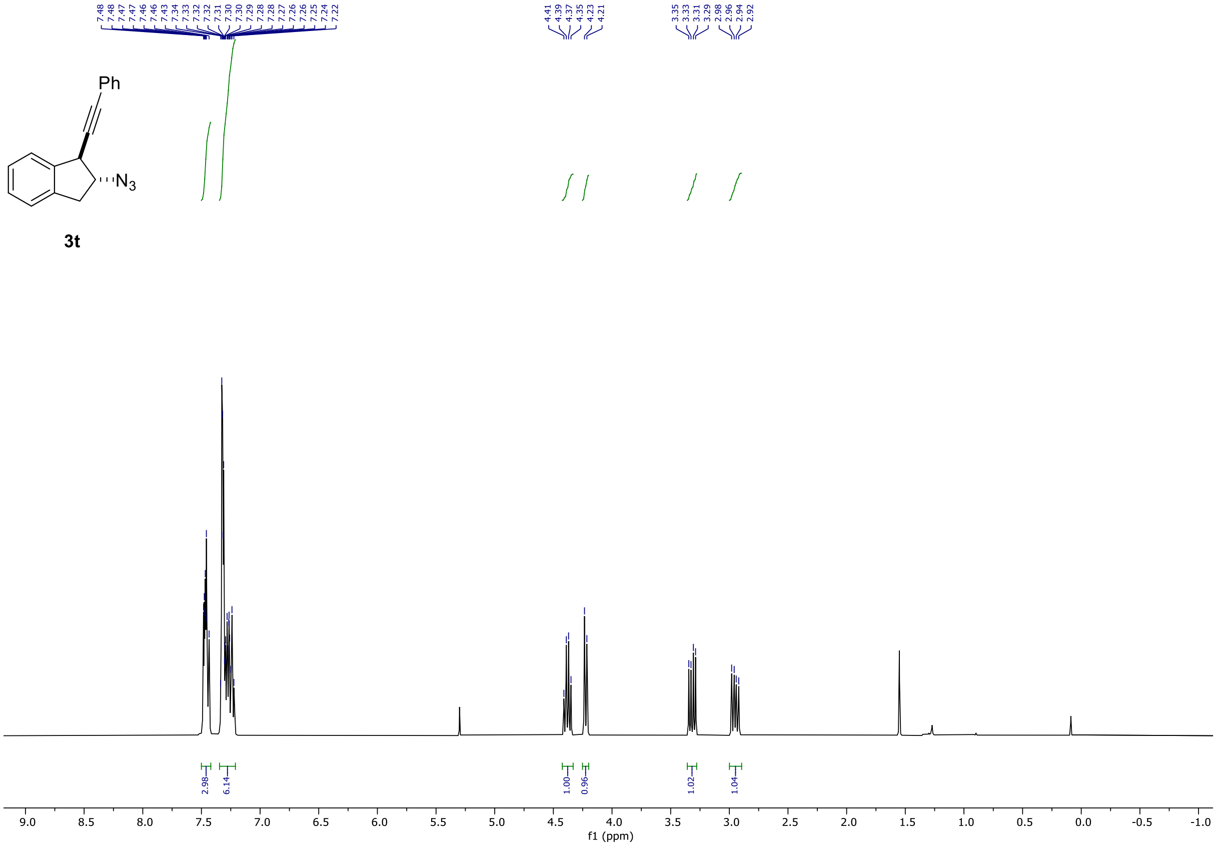

^{13}C NMR (101 MHz, $CDCl_3$) of compound **3t**:

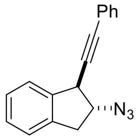

3t

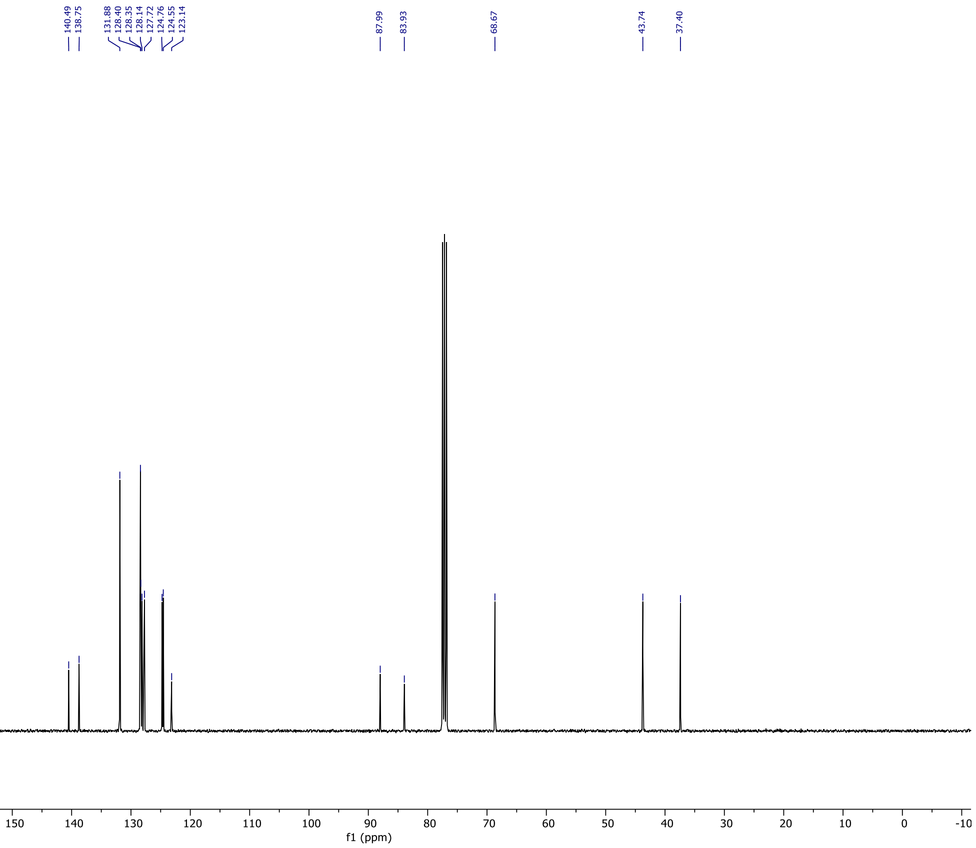

^{1}H NMR (400 MHz, $CDCl_3$) of compound **3t’**:

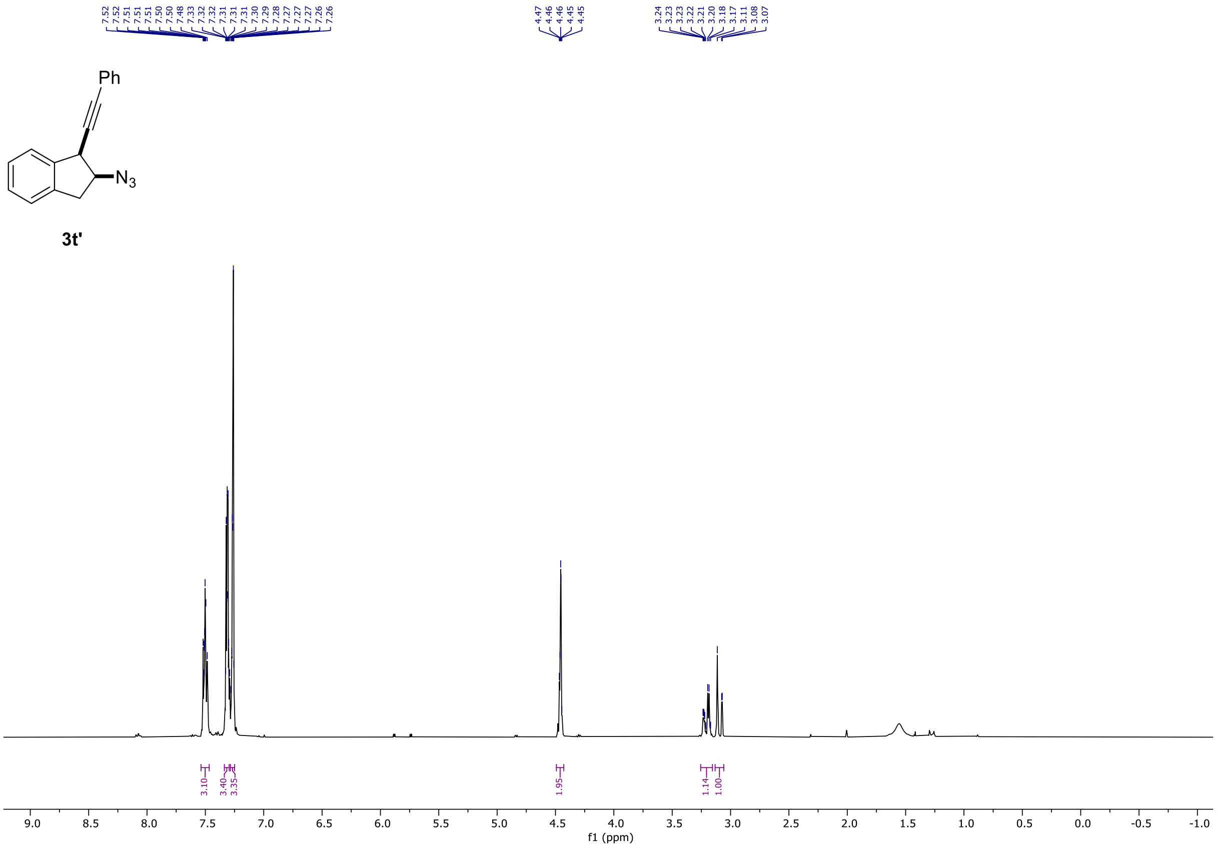

^{13}C NMR (101 MHz, $CDCl_3$) of compound **3t’**:

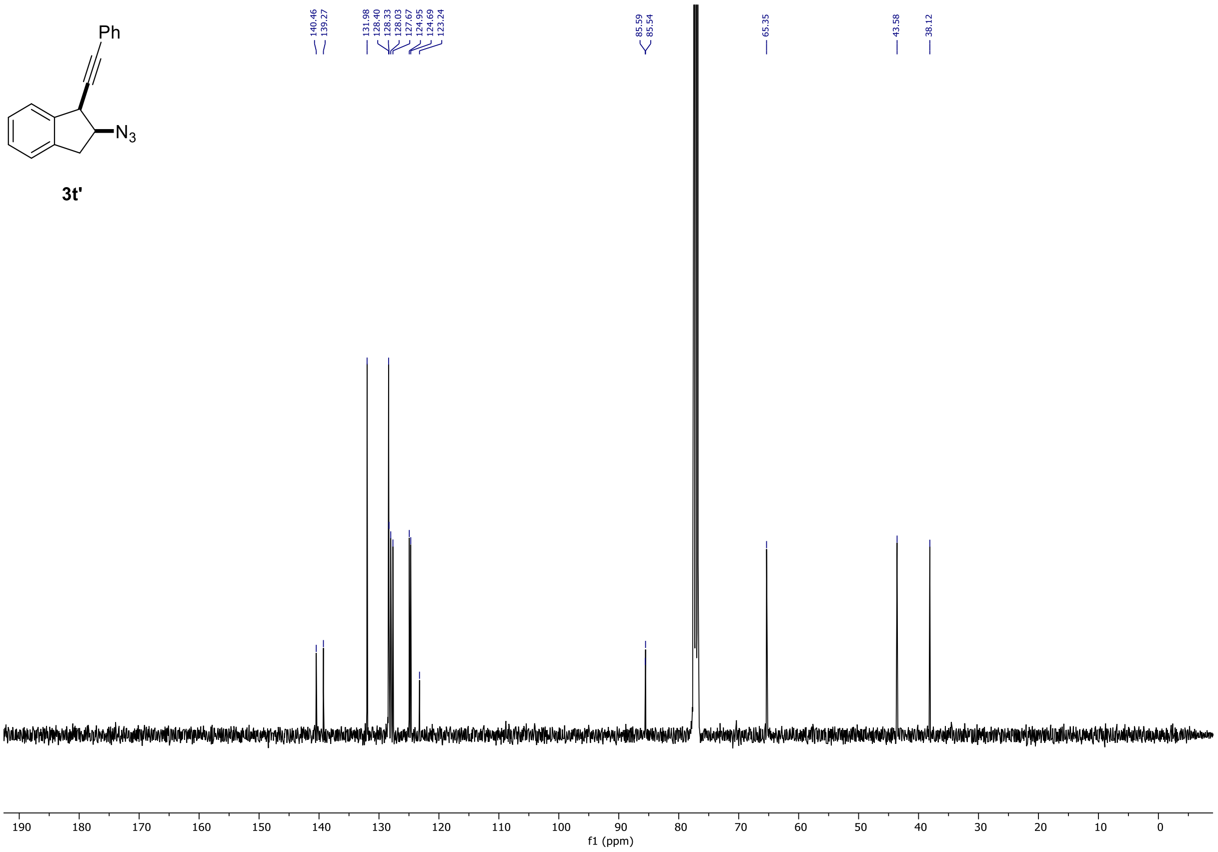

^{1}H NMR (400 MHz, $CDCl_3$) of compound **3u/3u'** (dr determination):

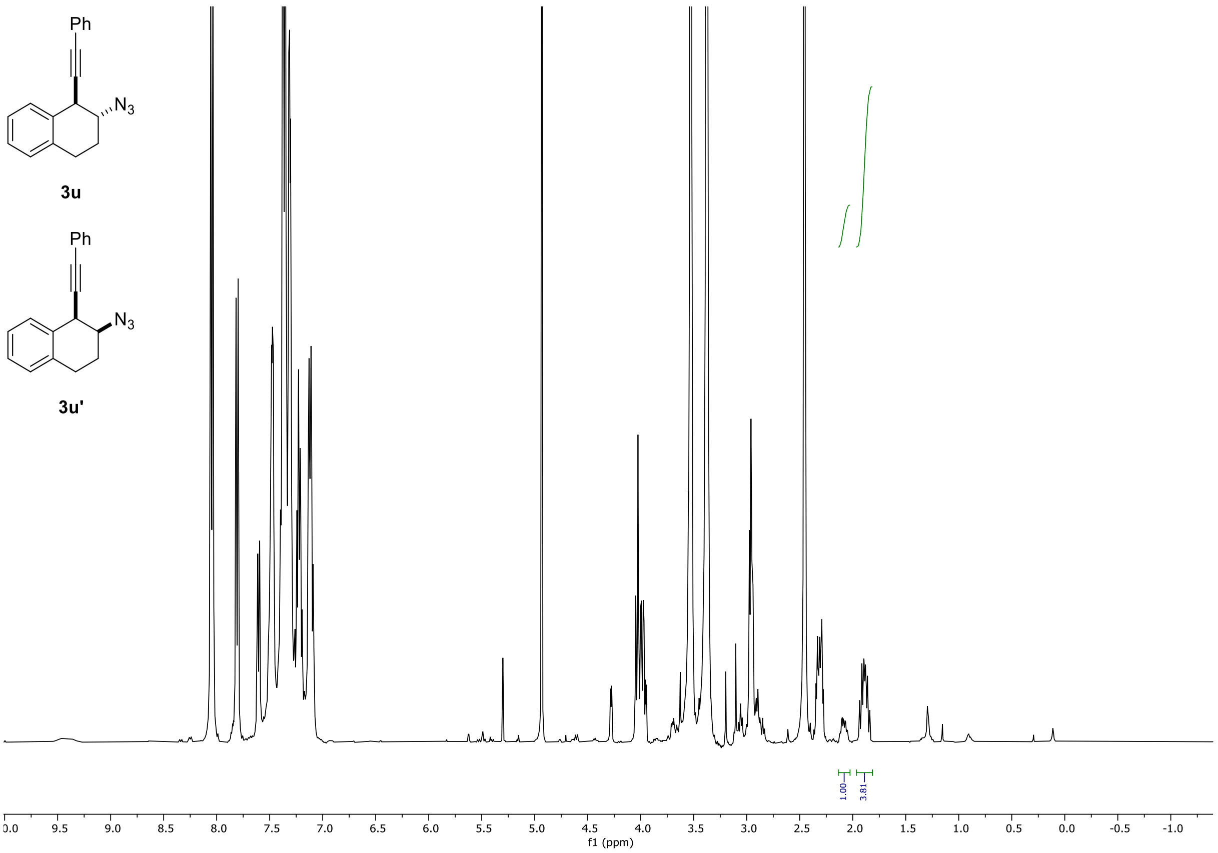

^{1}H NMR (400 MHz, $CDCl_3$) of compound **3u**:

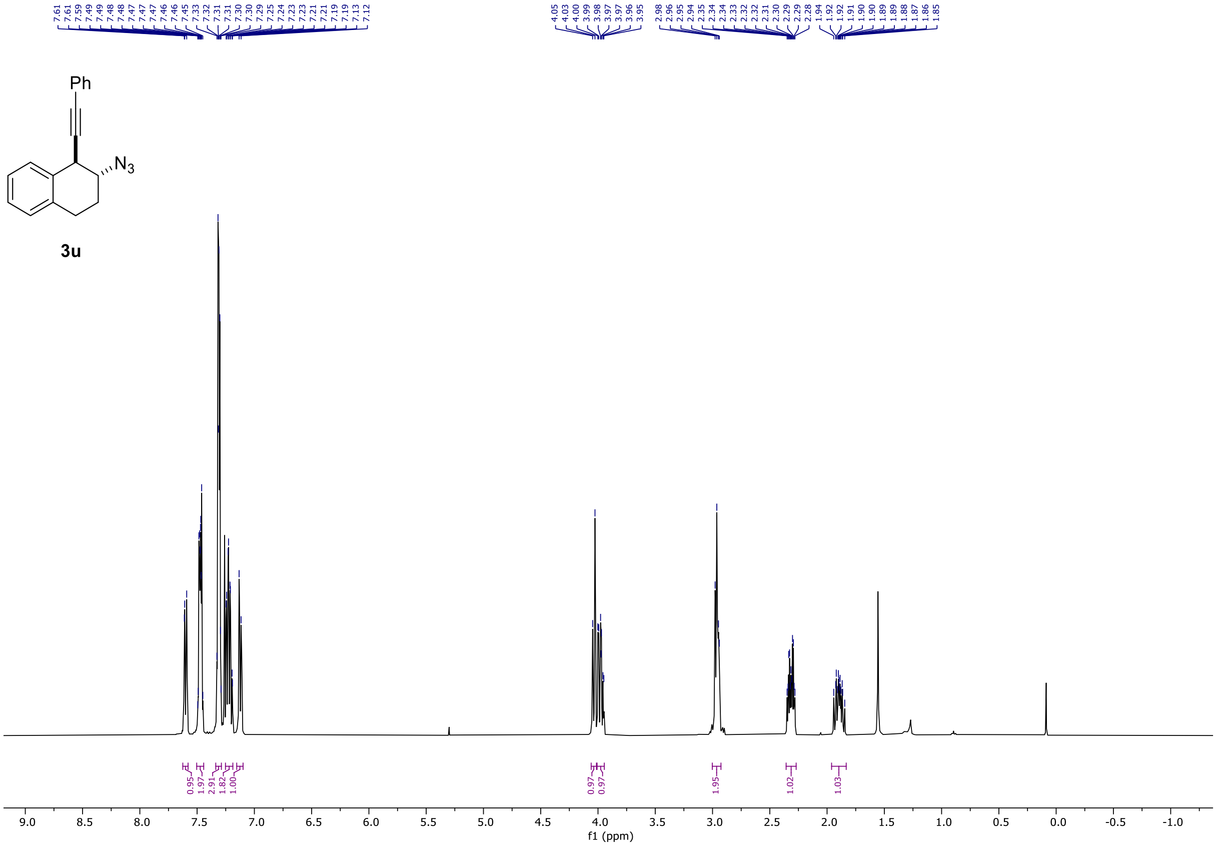

^{13}C NMR (101 MHz, $CDCl_3$) of compound **3u**:

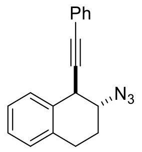

3u

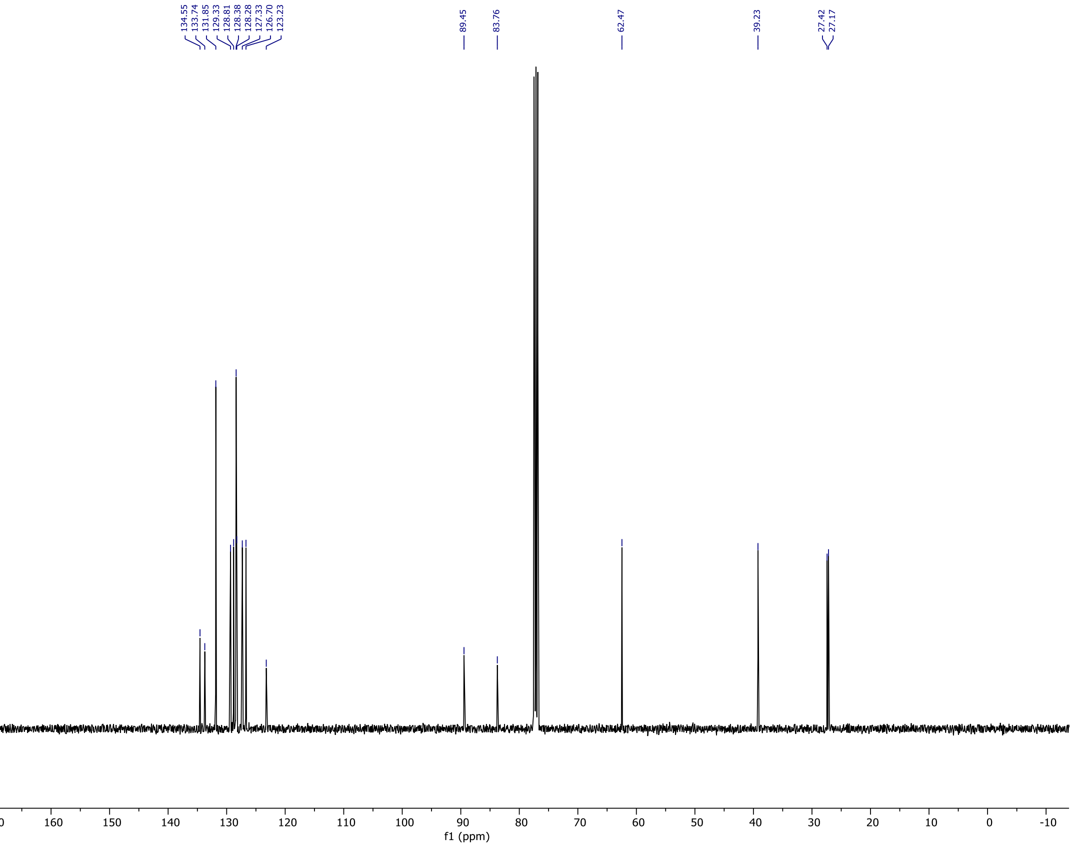

^{1}H NMR (400 MHz, $CDCl_3$) of compound **3u'**:

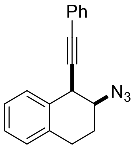

3u'

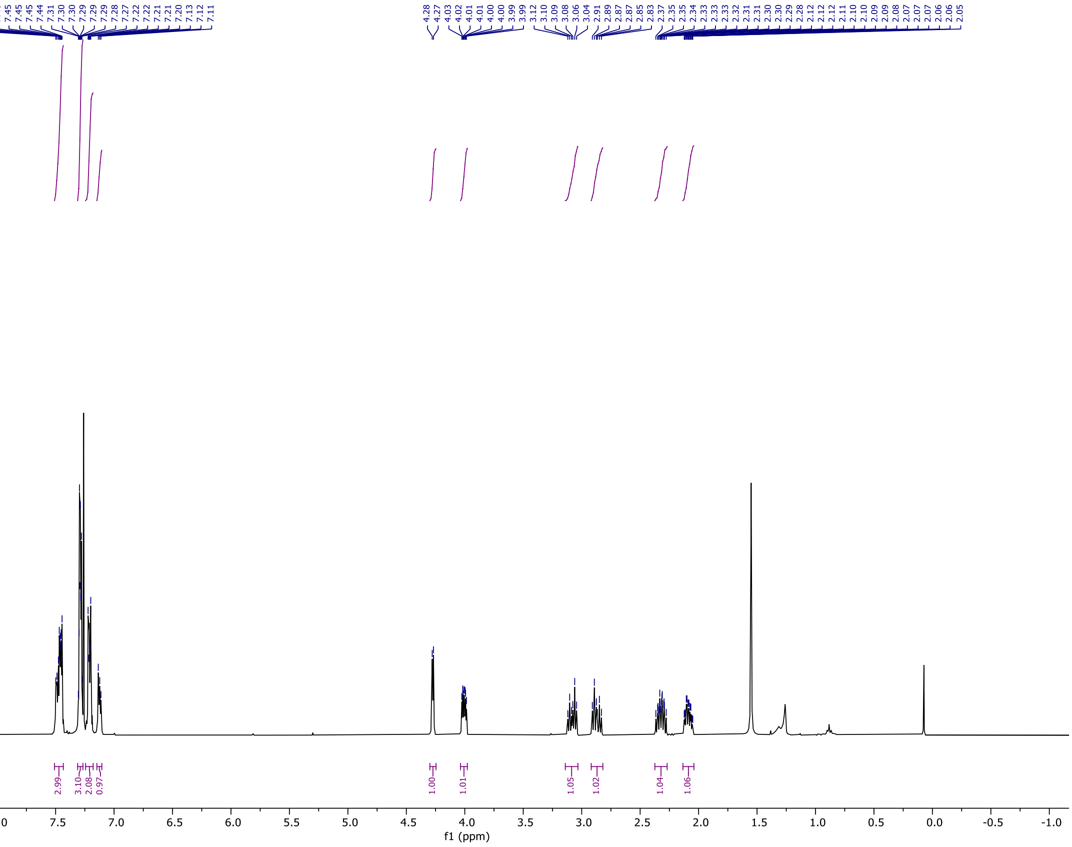

^{13}C NMR (101 MHz, $CDCl_3$) of compound **3u'**:

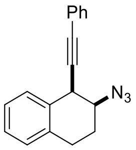

3u'

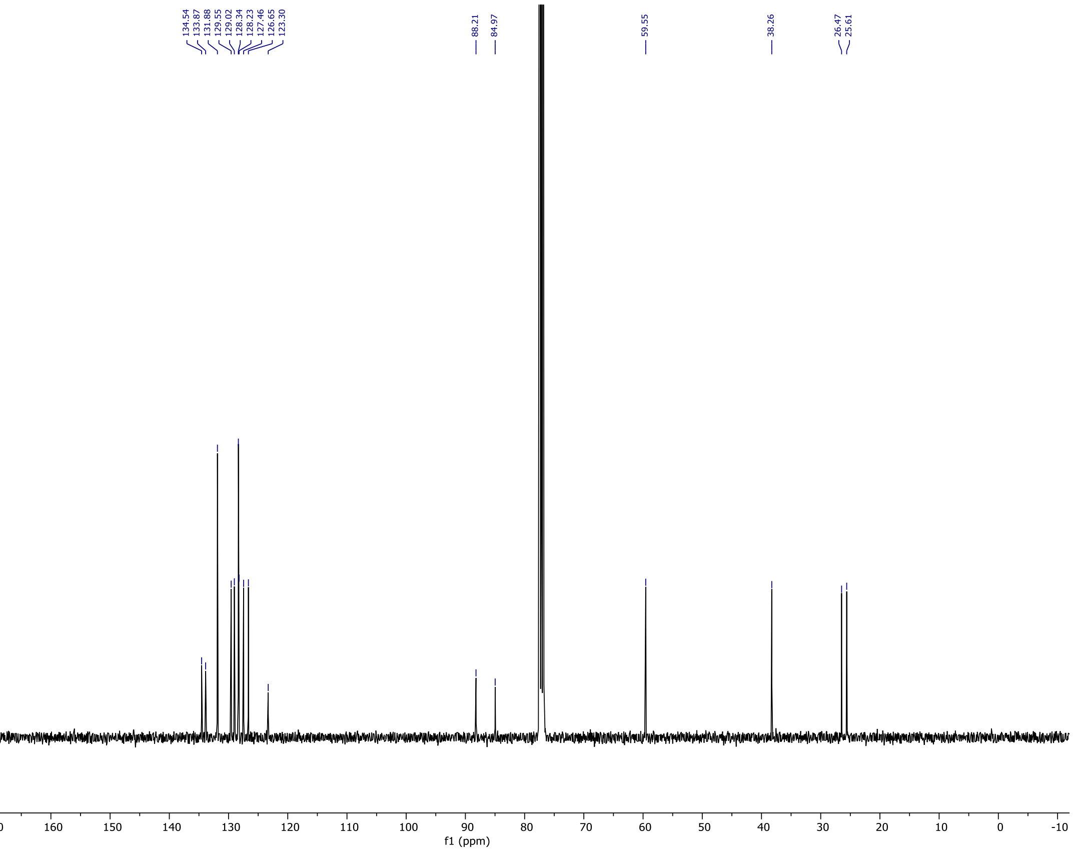

^{1}H NMR (400 MHz, $CDCl_3$) of compound **3v**:

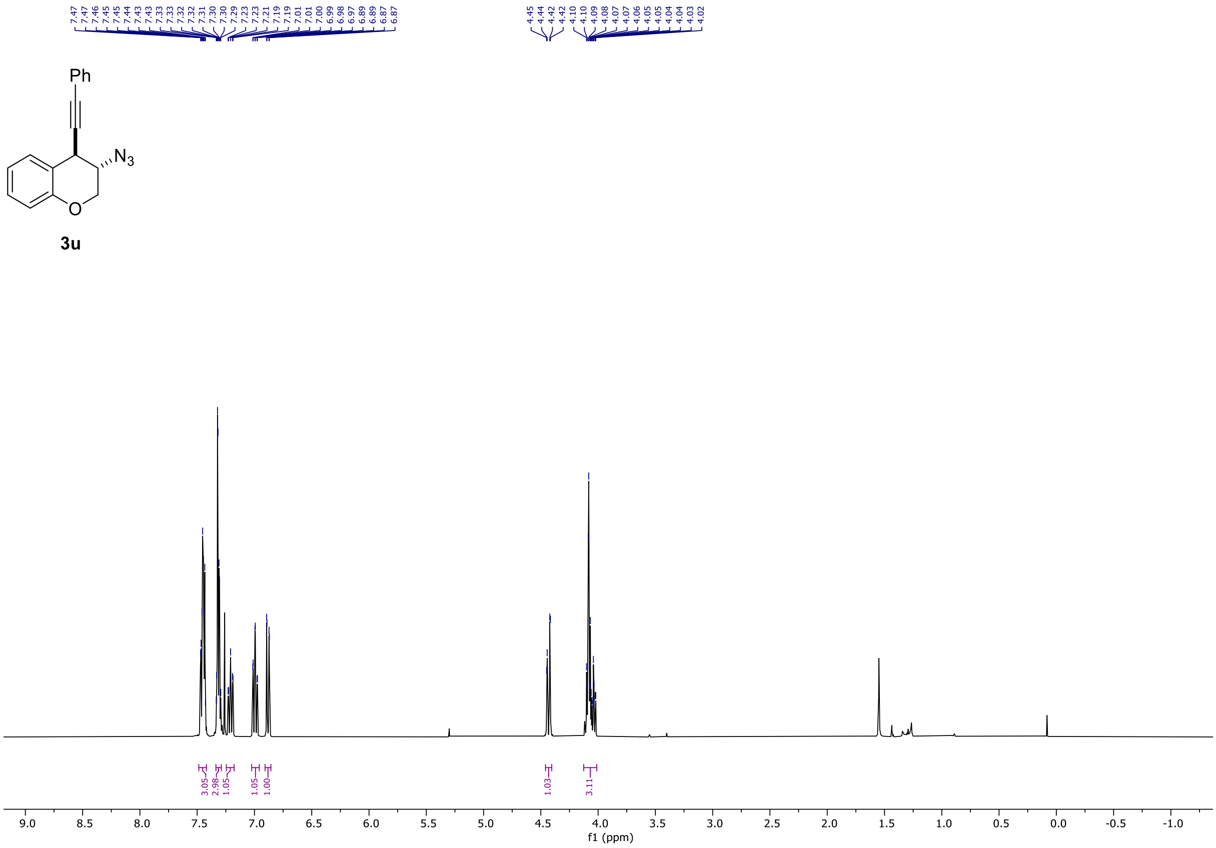

^{13}C NMR (101 MHz, $CDCl_3$) of compound **3v**:

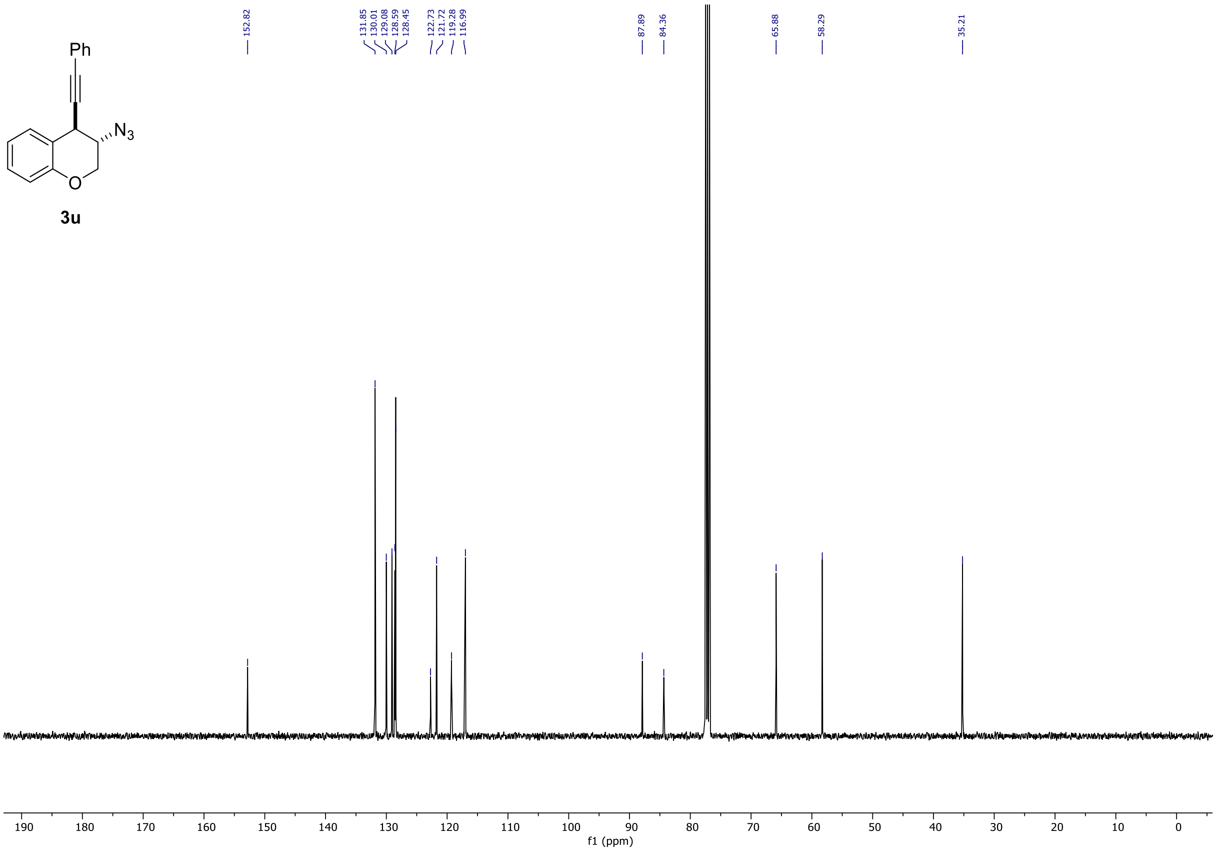

^{1}H NMR (400 MHz, $CDCl_3$) of compound **3w**:

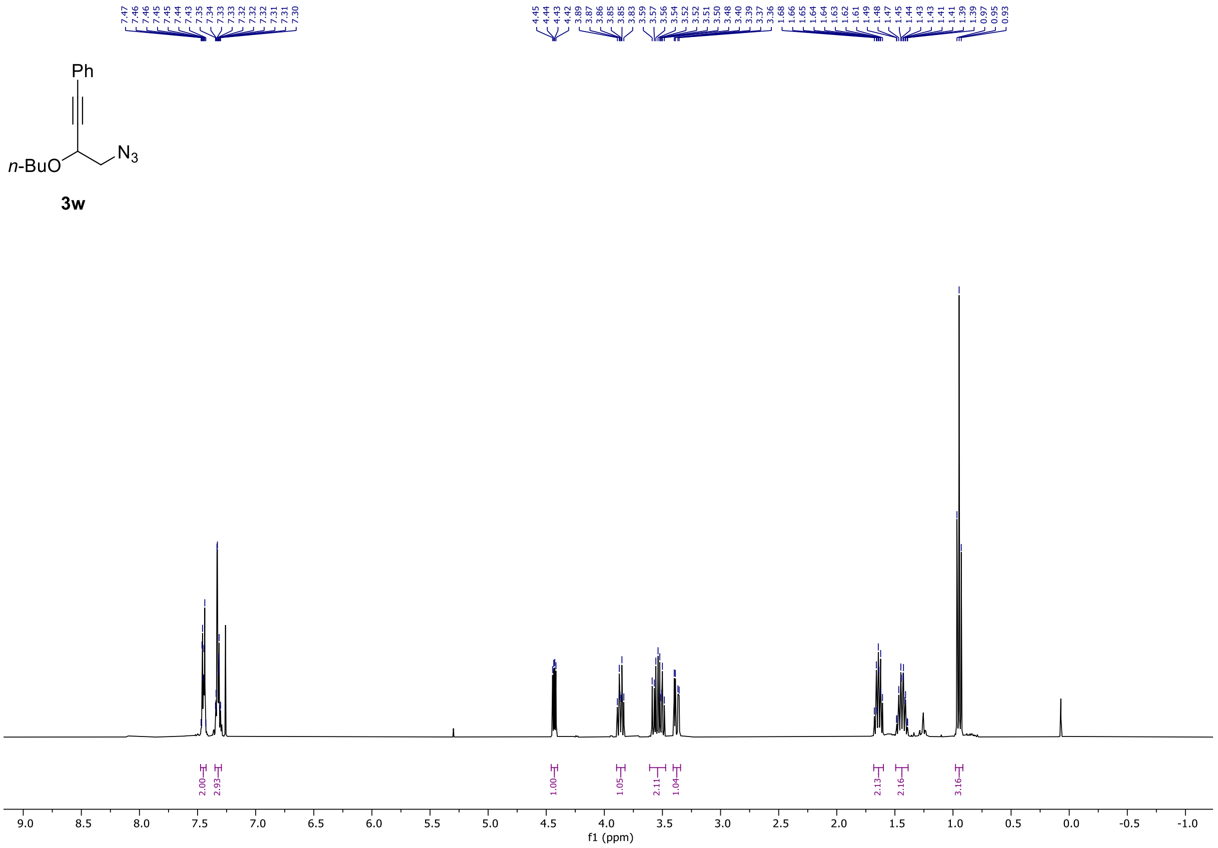

^{13}C NMR (101 MHz, $CDCl_3$) of compound **3w**:

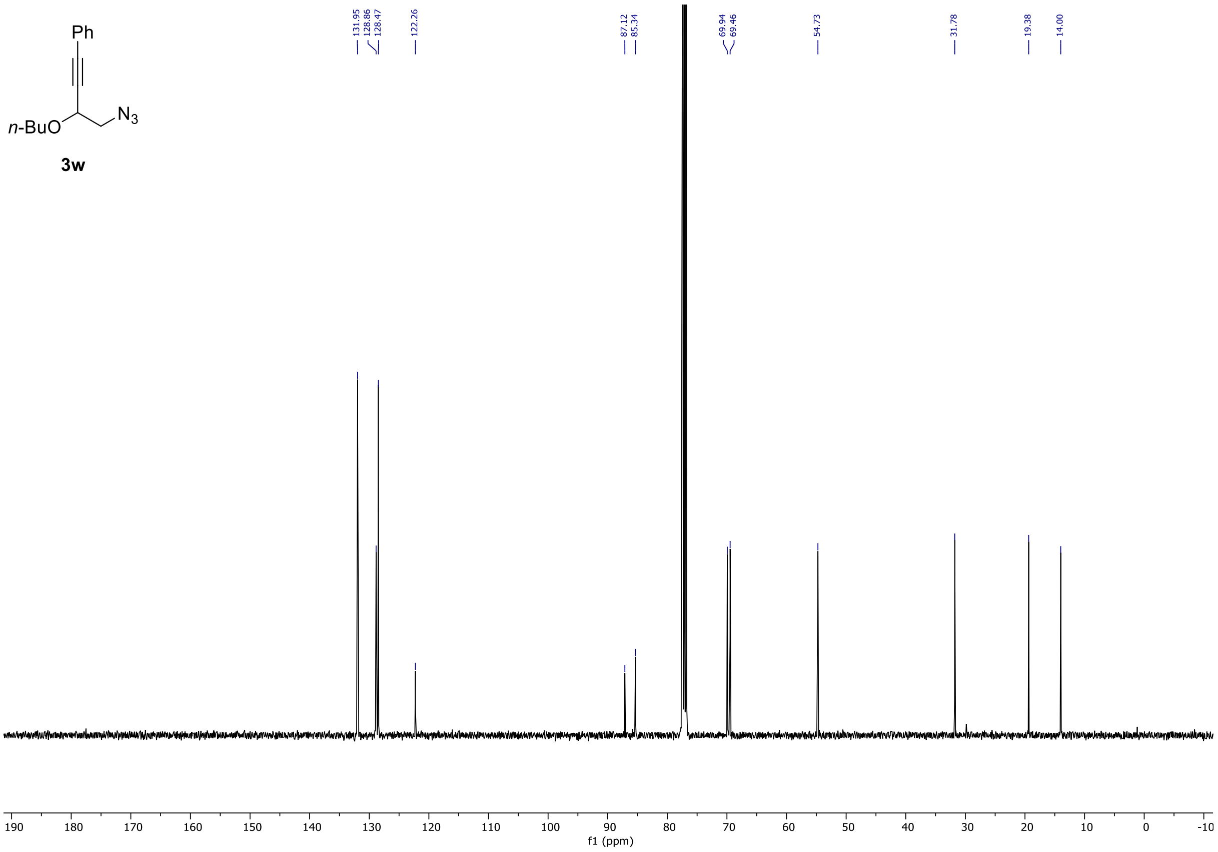

^{1}H NMR (400 MHz, $CDCl_3$) of compound **3x**:

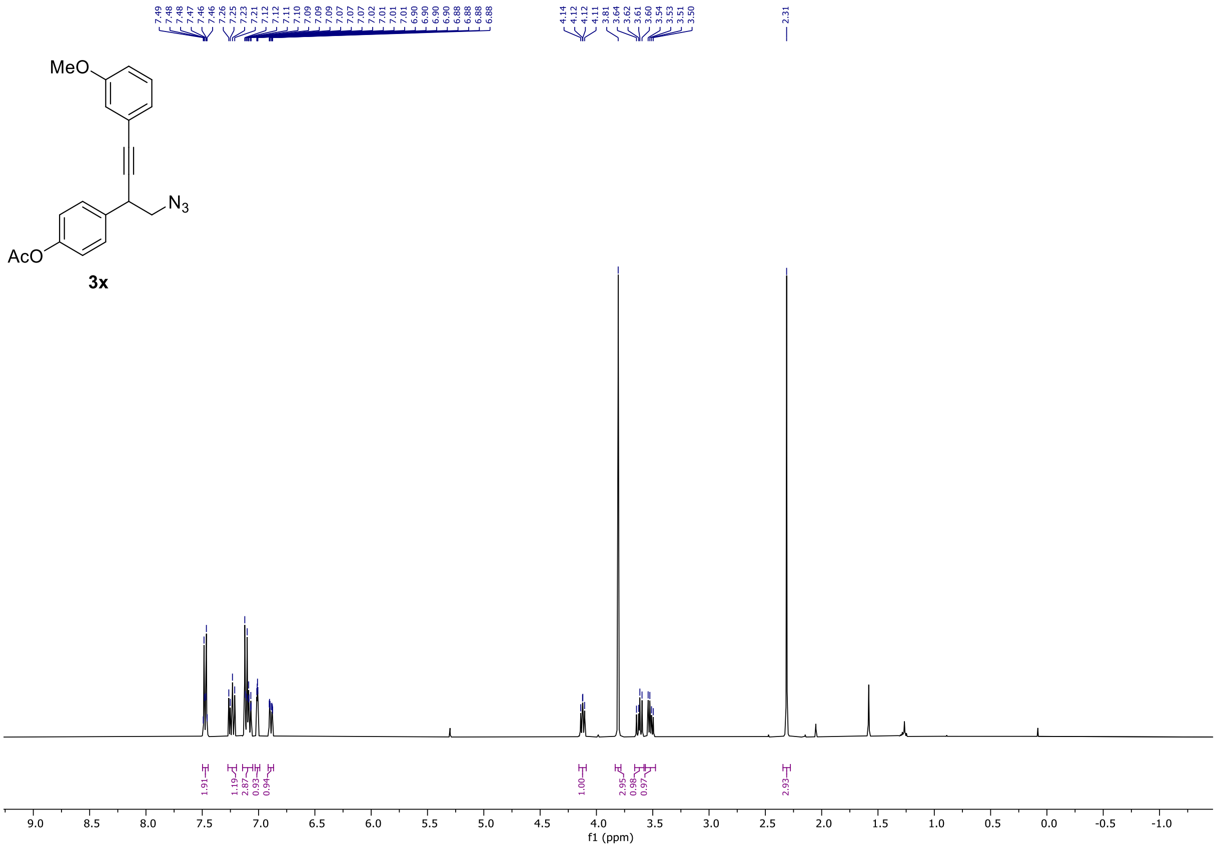

^{13}C NMR (101 MHz, $CDCl_3$) of compound **3x**:

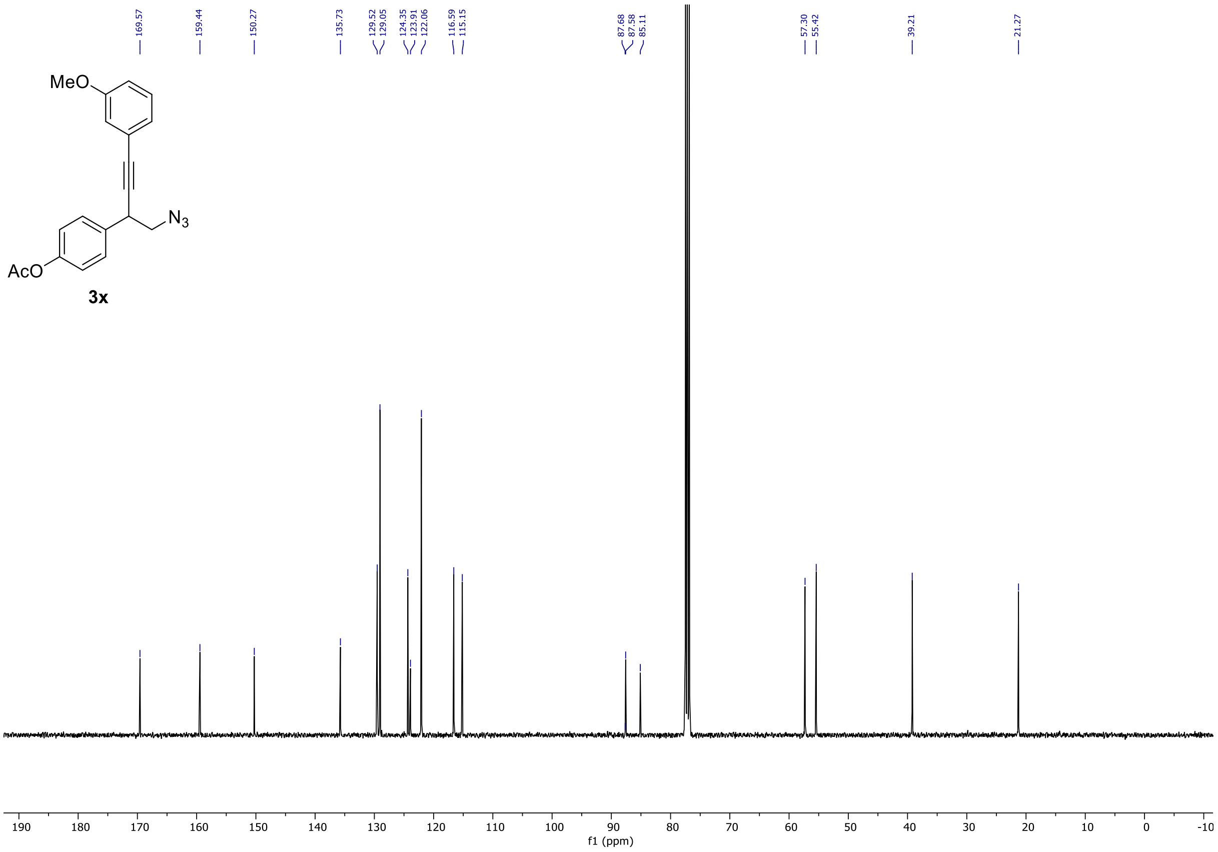

^{1}H NMR (400 MHz, $CDCl_3$) of compound **3y**:

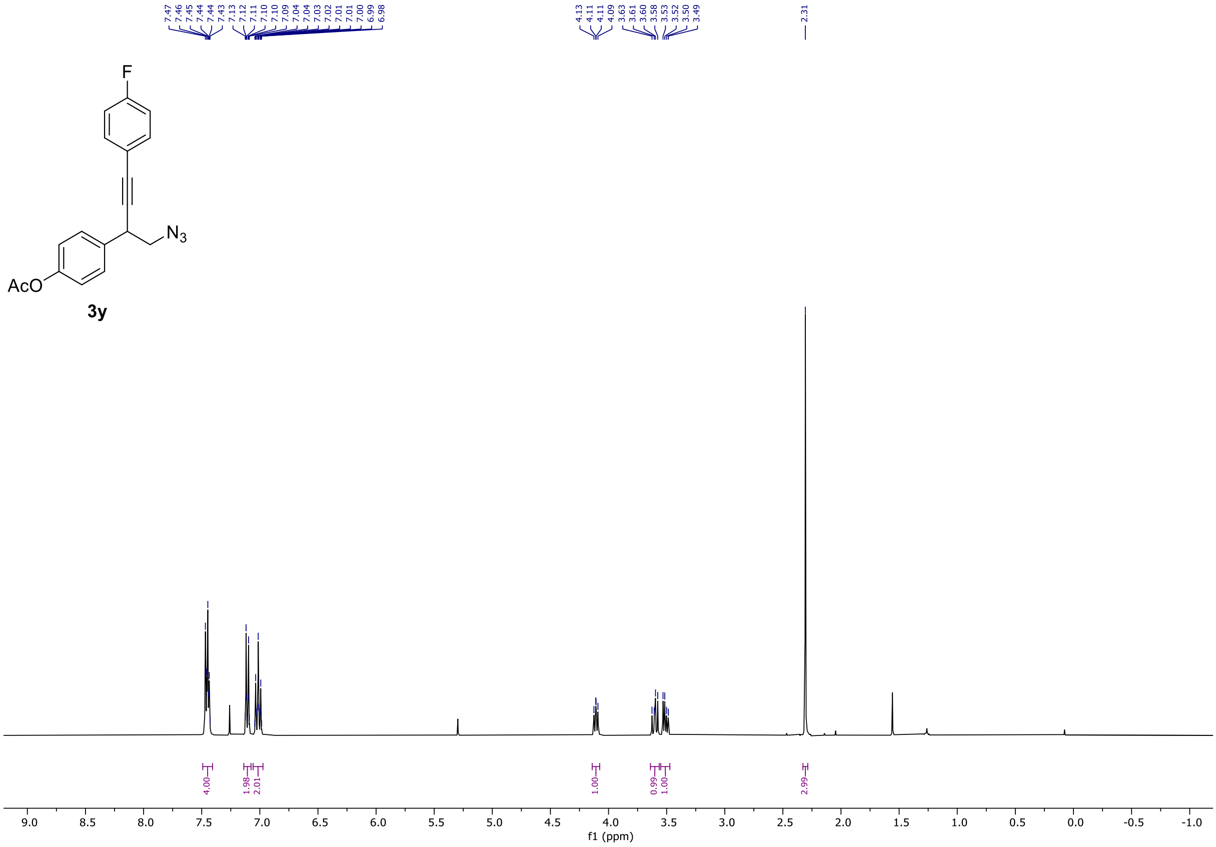

^{13}C NMR (101 MHz, $CDCl_3$) of compound **3y**:

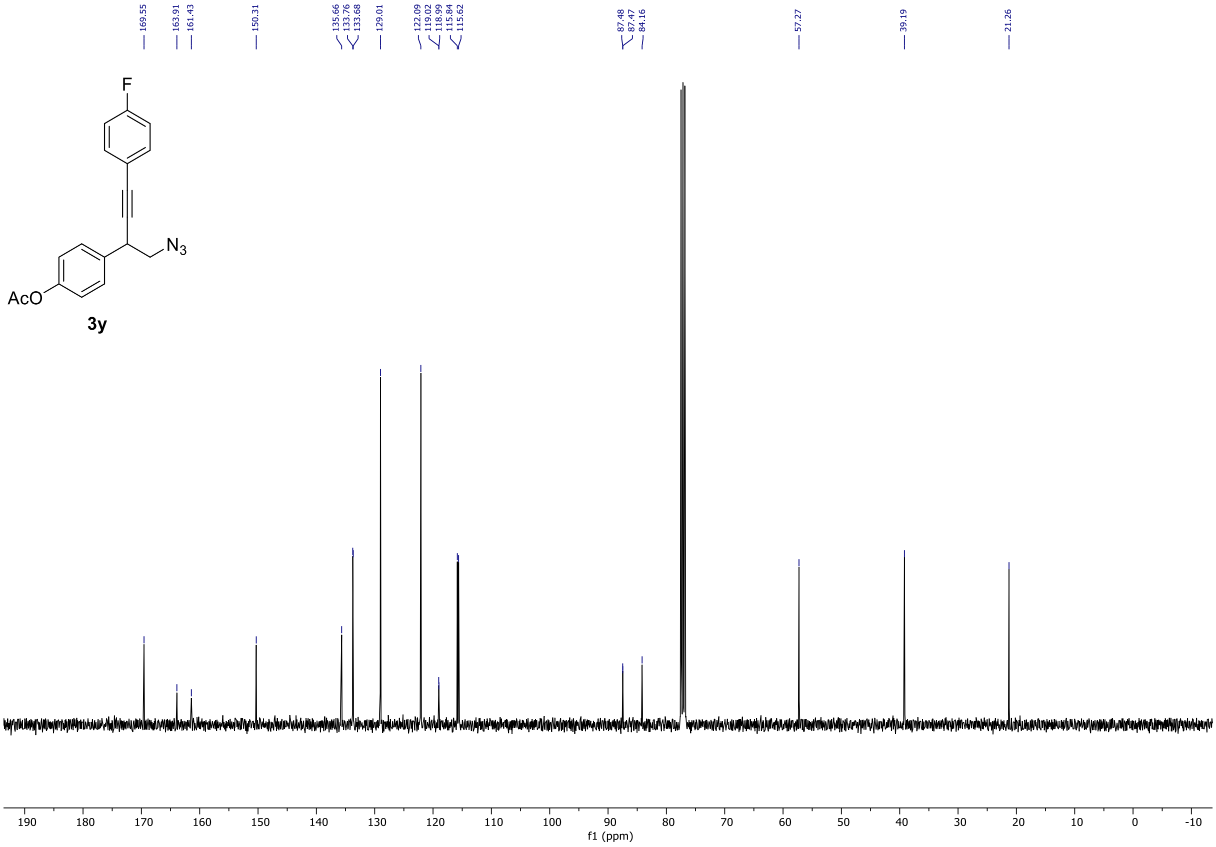

^{19}F NMR (376 MHz, $CDCl_3$) of compound **3y**:

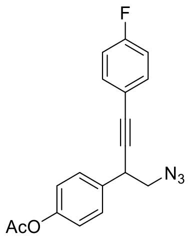

3y

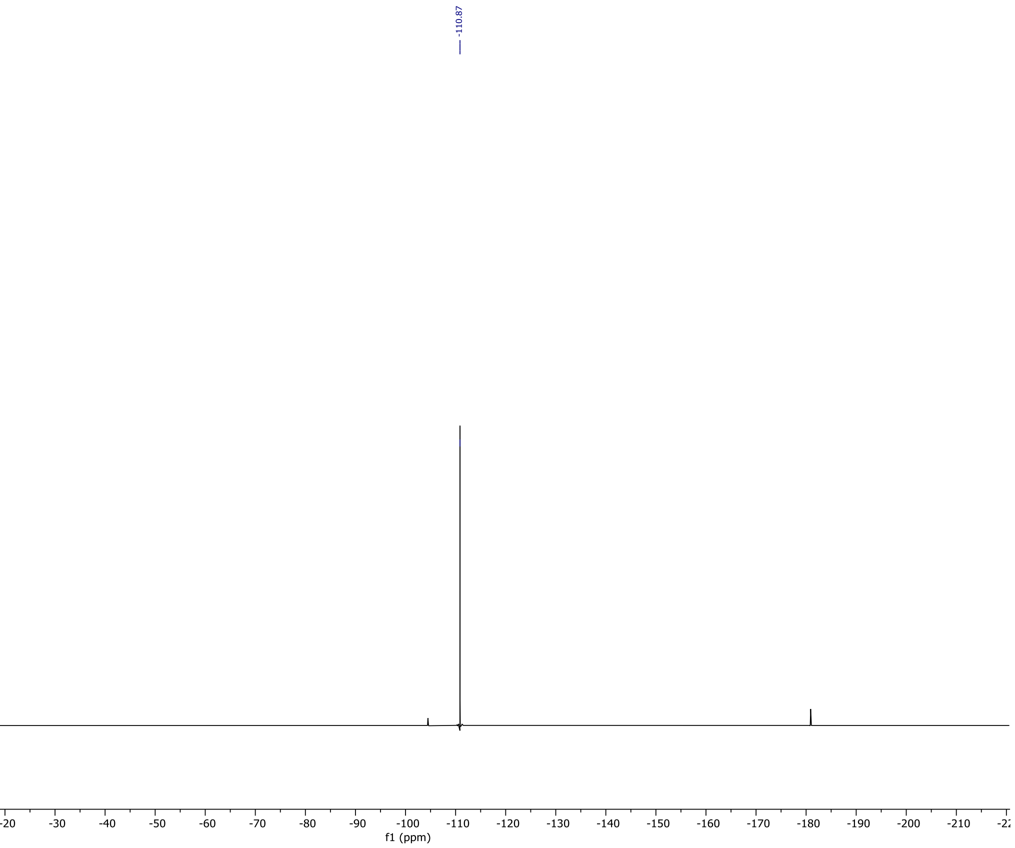

^{1}H NMR (400 MHz, $CDCl_3$) of compound **3z**:

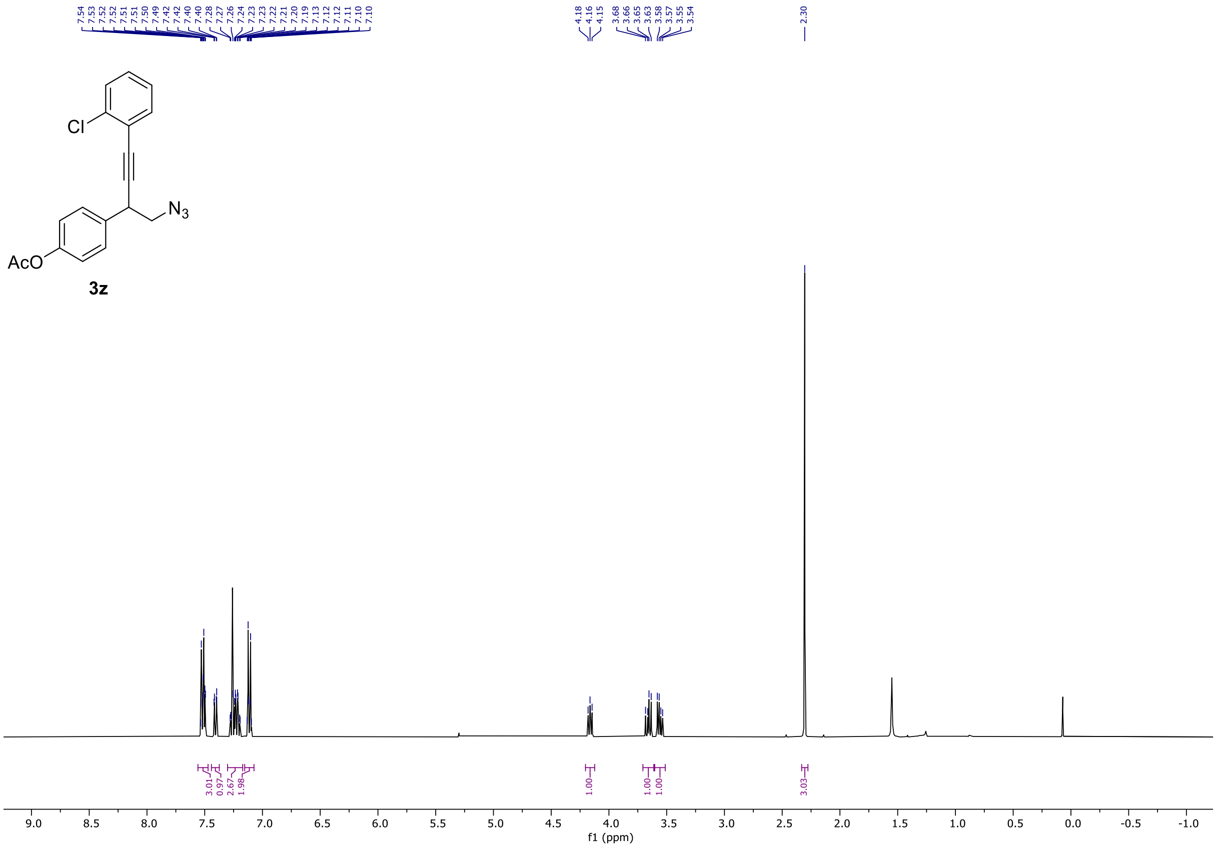

^{13}C NMR (101 MHz, $CDCl_3$) of compound **3z**:

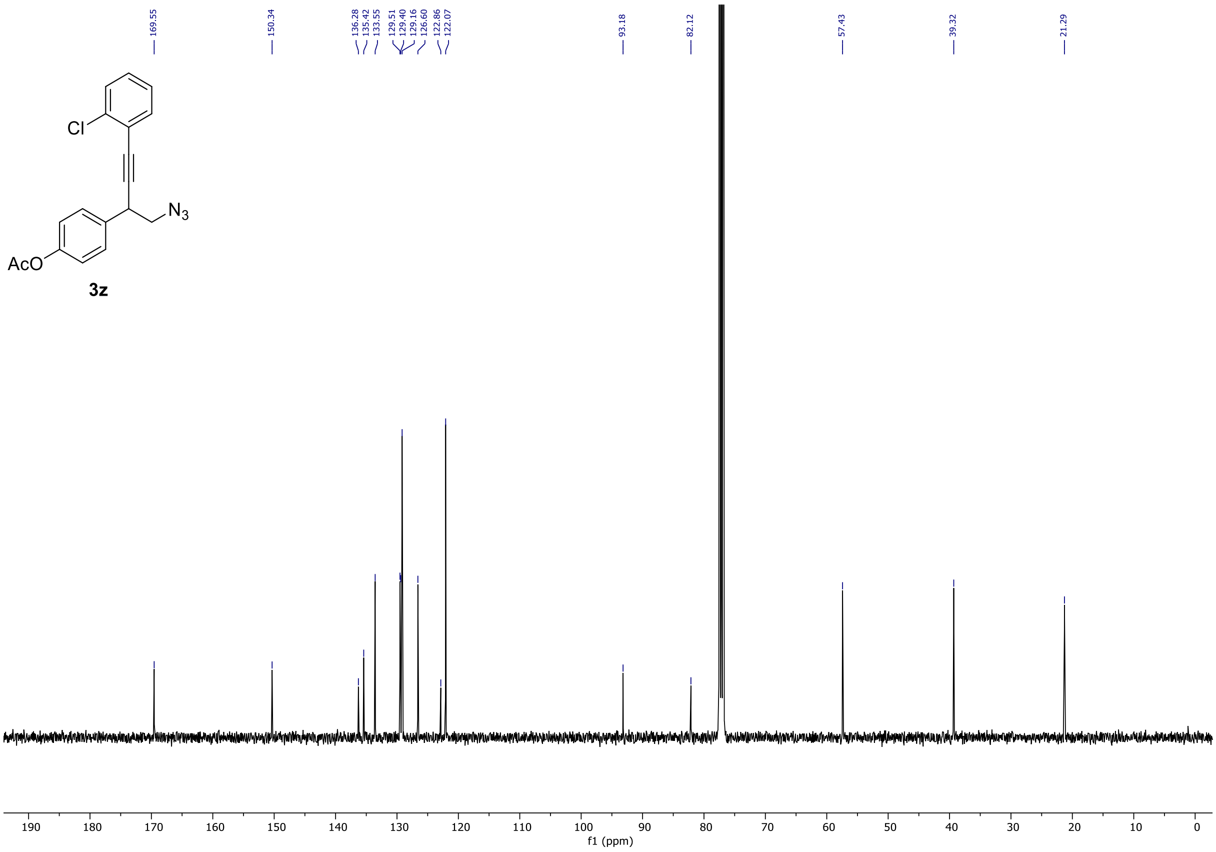

^{1}H NMR (400 MHz, $CDCl_3$) of compound **3aa**:

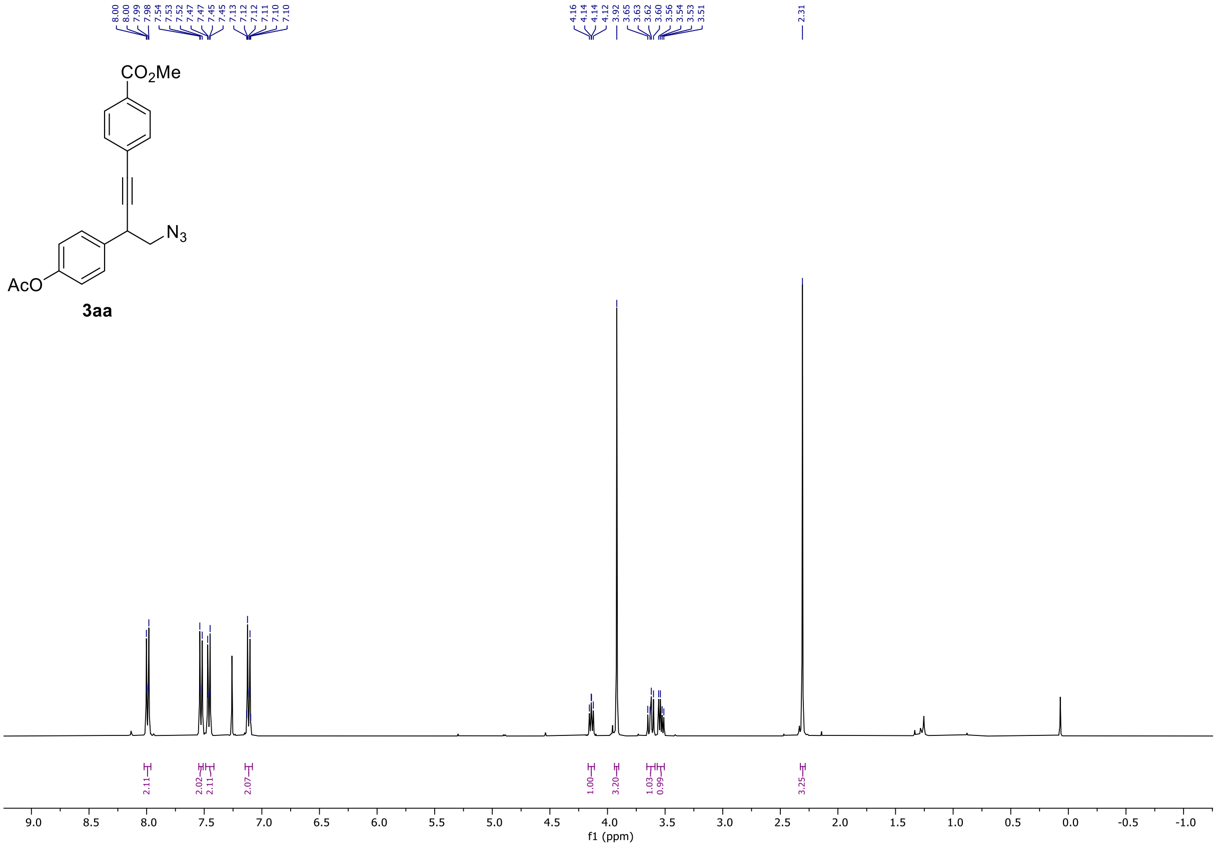

^{13}C NMR (101 MHz, $CDCl_3$) of compound **3aa**:

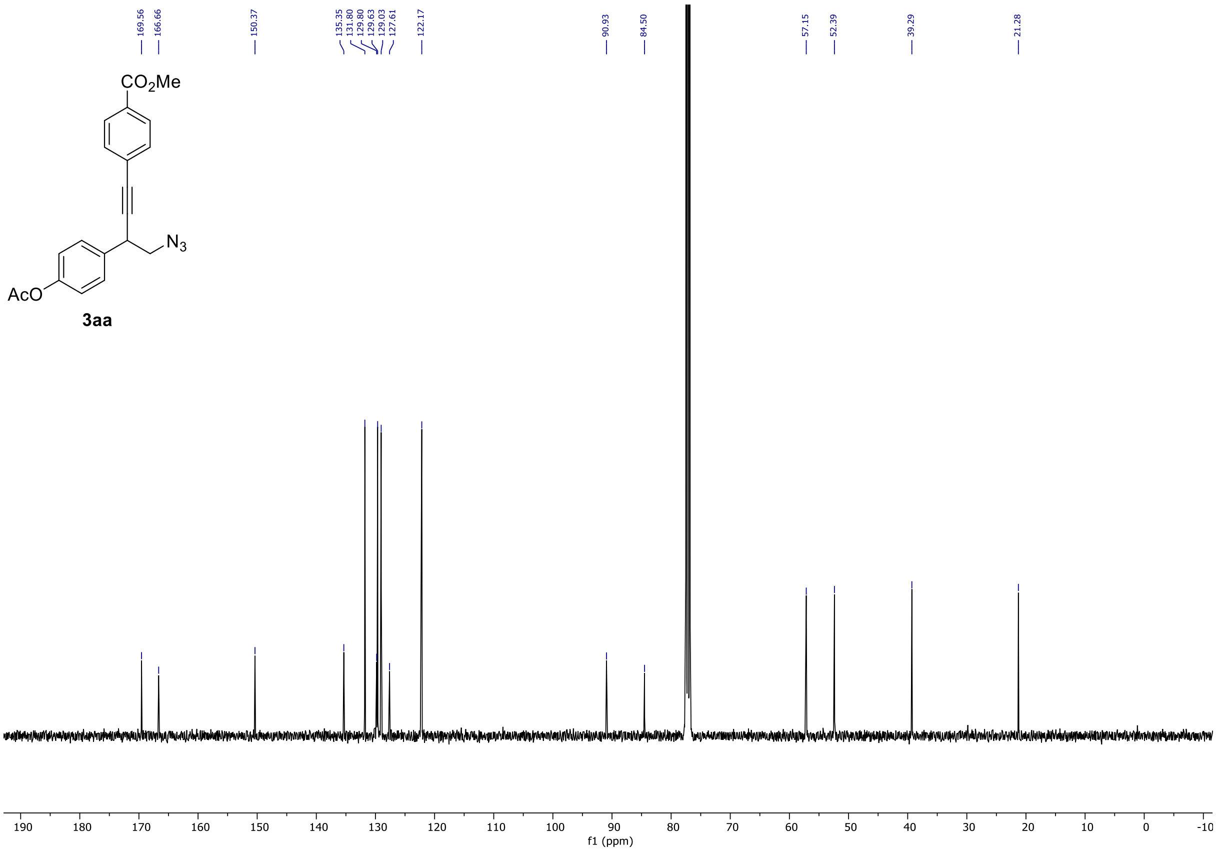

^{1}H NMR (400 MHz, $CDCl_3$) of compound **3ab**:

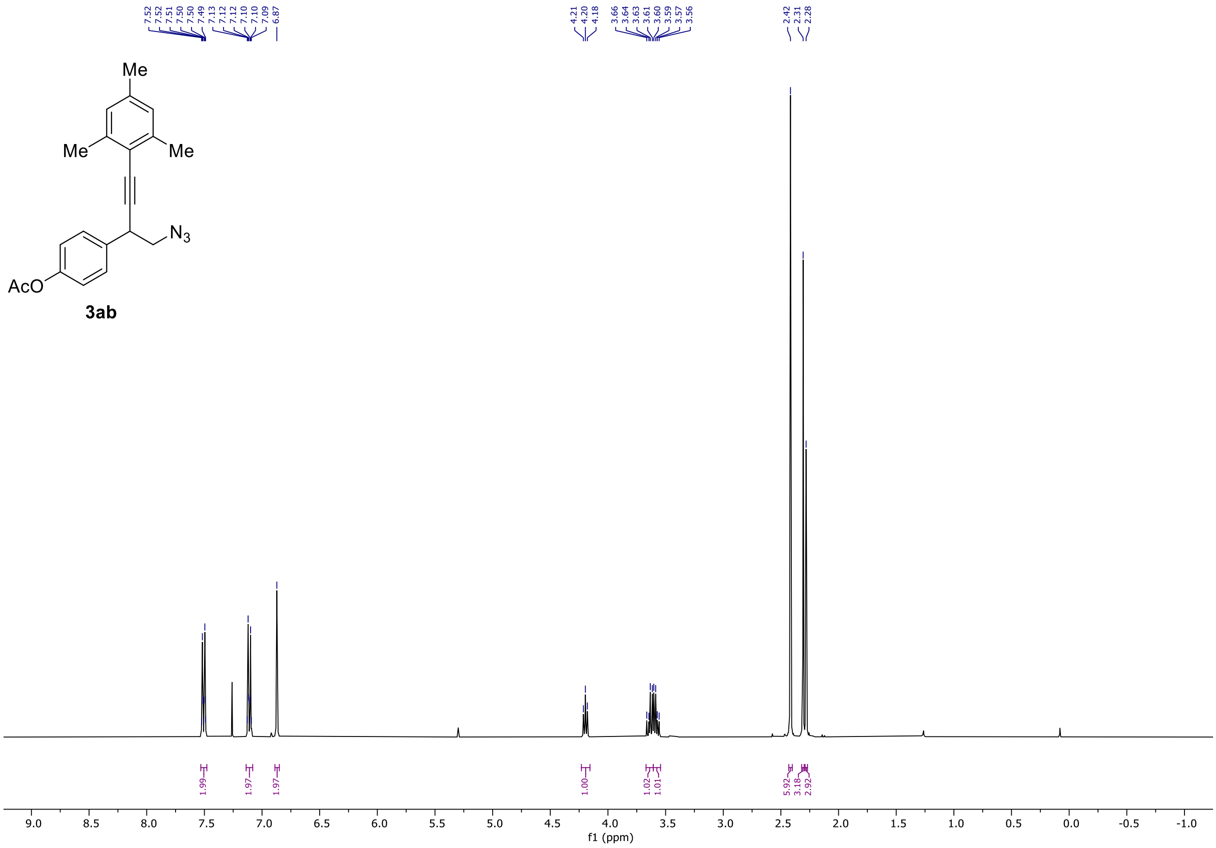

^{13}C NMR (101 MHz, $CDCl_3$) of compound **3ab**:

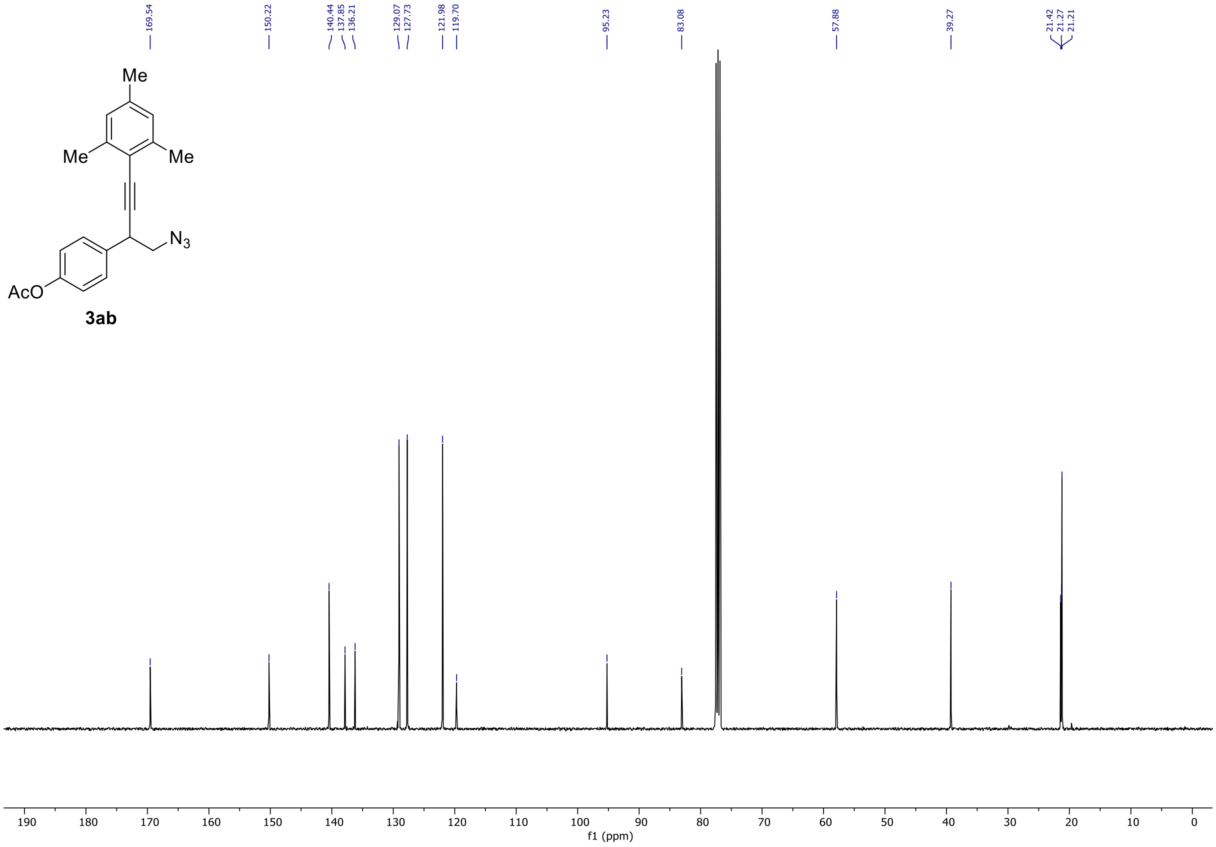

^{1}H NMR (400 MHz, $CDCl_3$) of compound **3ac**:

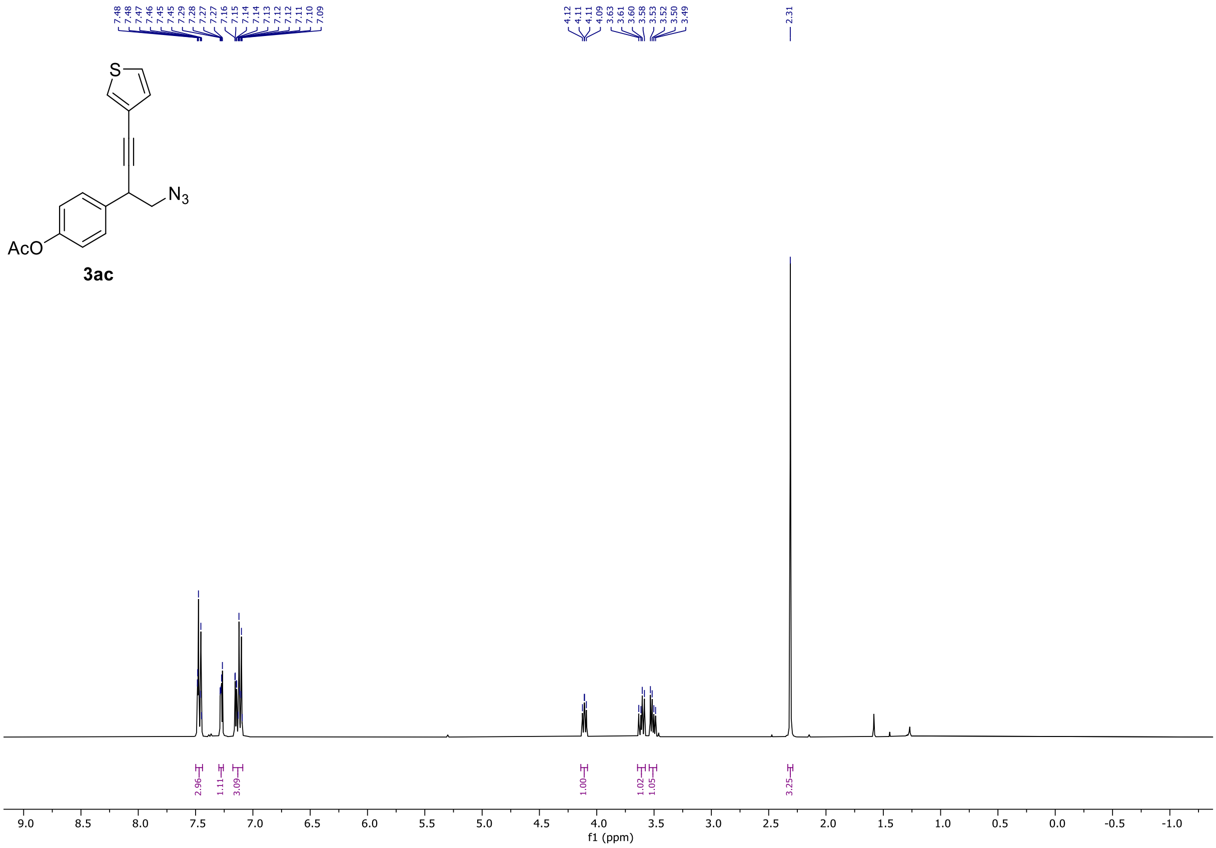

^{13}C NMR (101 MHz, $CDCl_3$) of compound **3ac**:

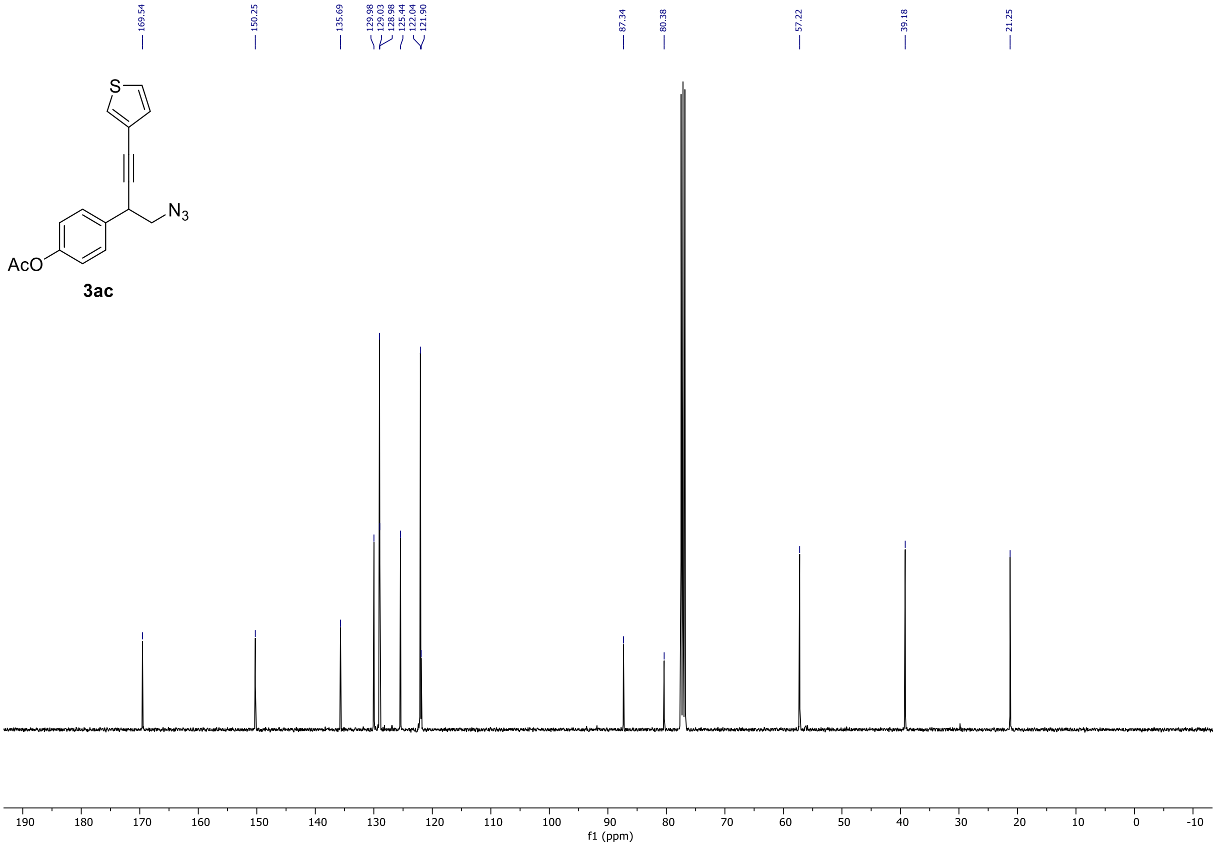

^{1}H NMR (400 MHz, $CDCl_3$) of compound **3ad**:

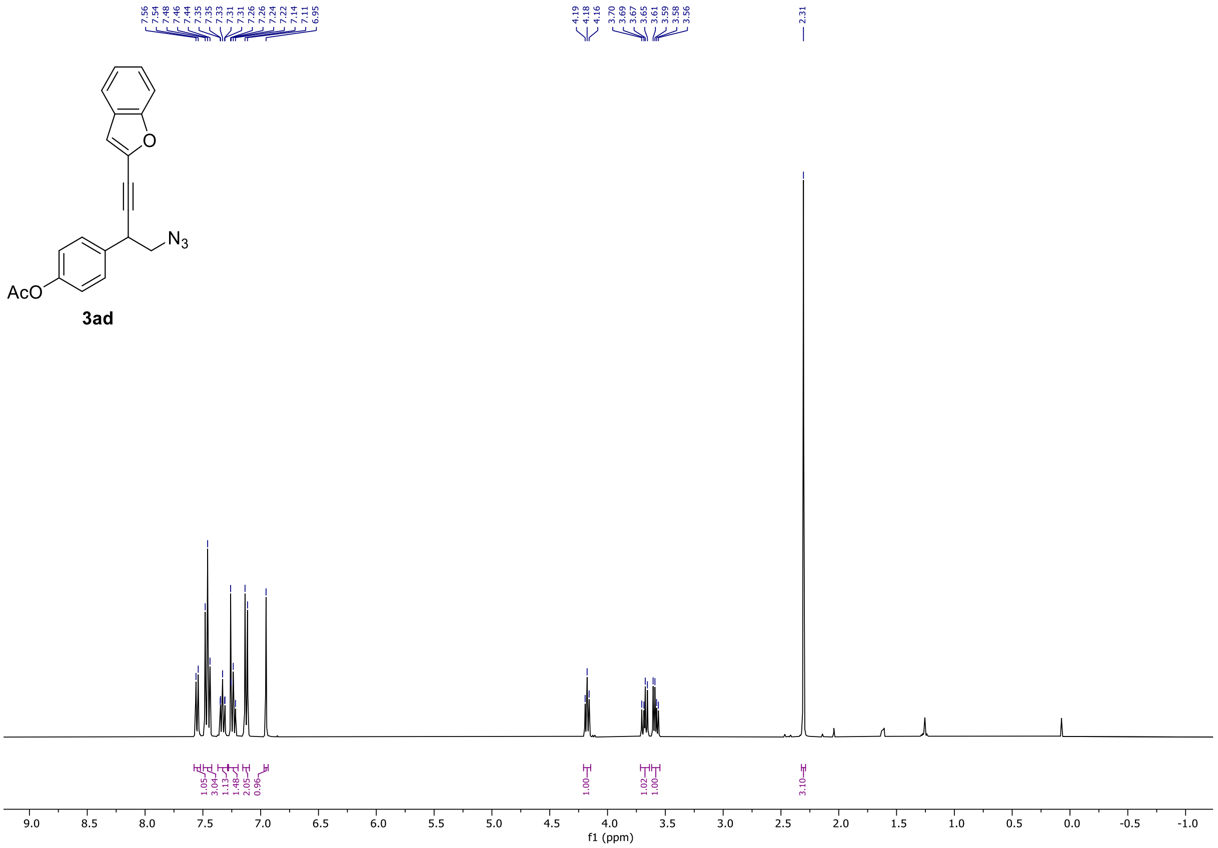

^{13}C NMR (101 MHz, $CDCl_3$) of compound **3ad**:

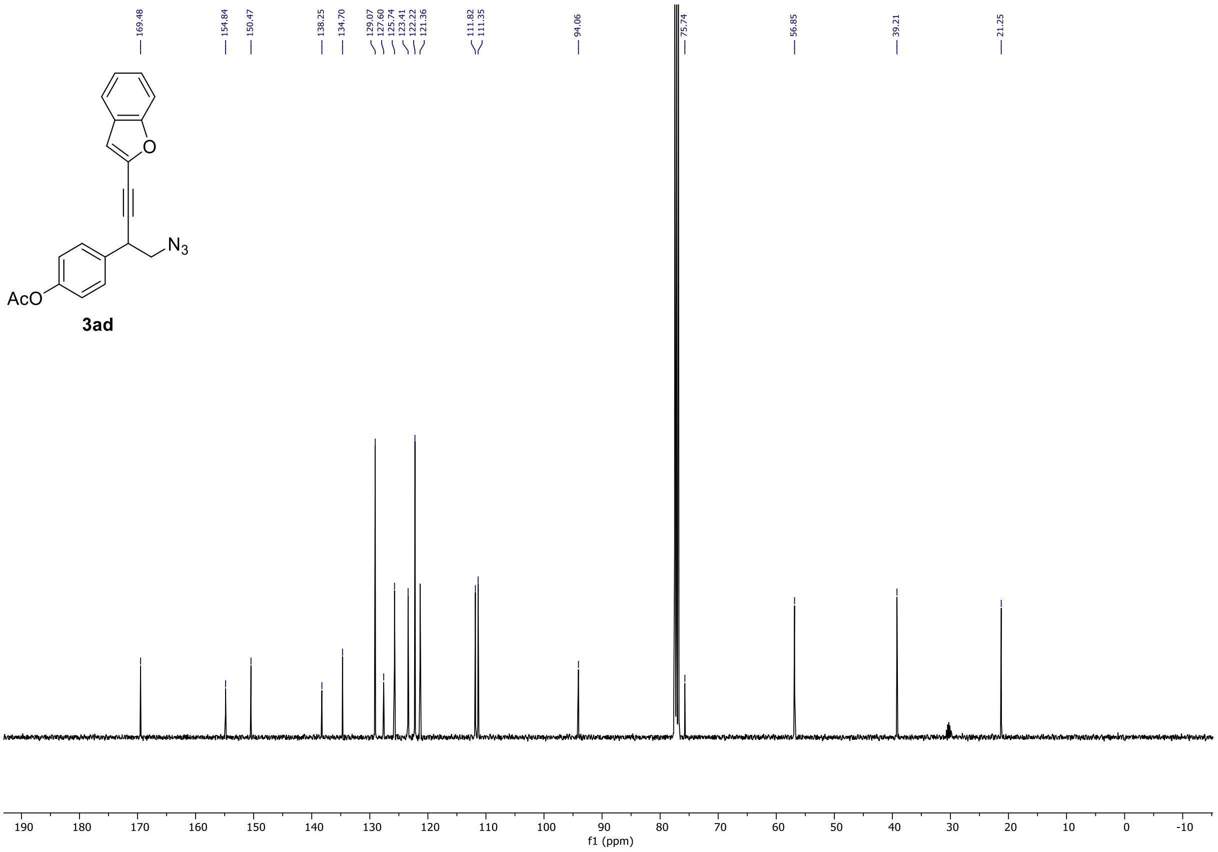

^{1}H NMR (400 MHz, $CDCl_3$) of compound **3ae**:

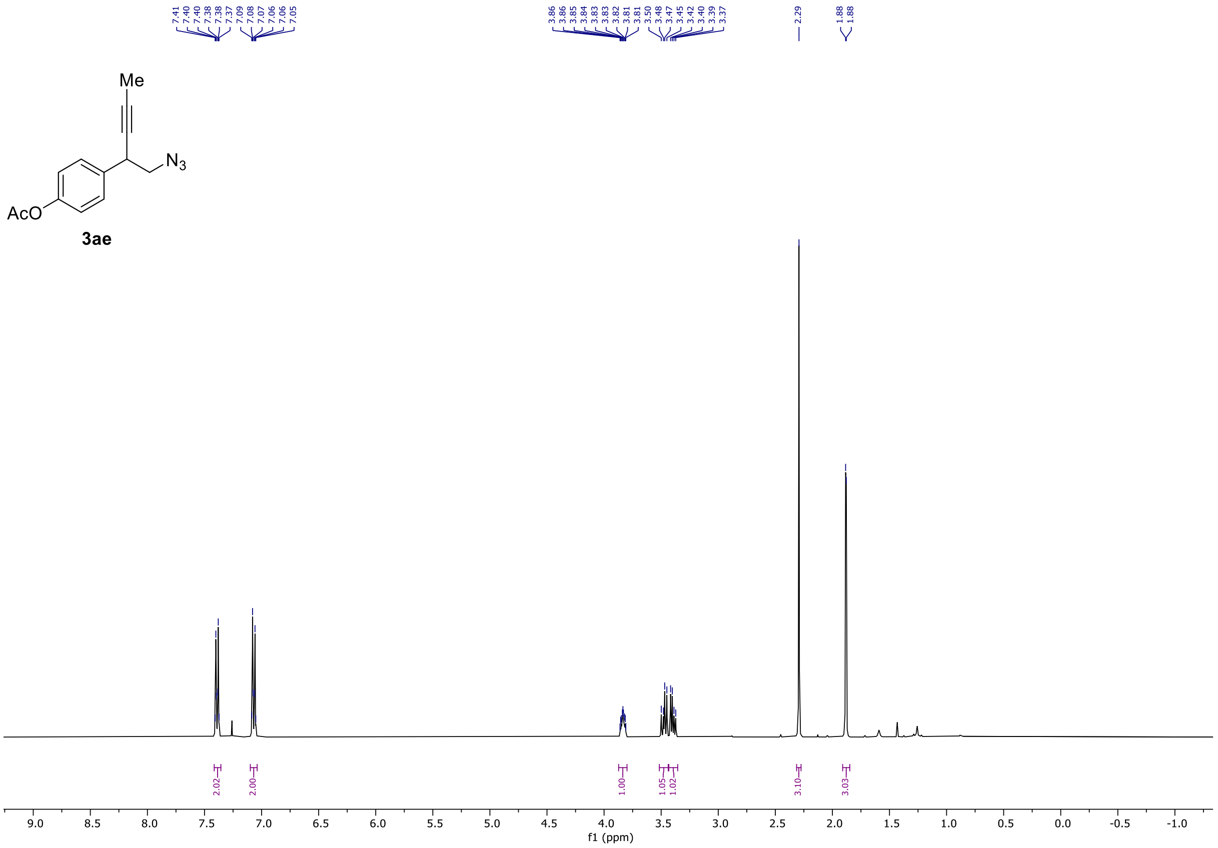

^{13}C NMR (101 MHz, $CDCl_3$) of compound **3ae**:

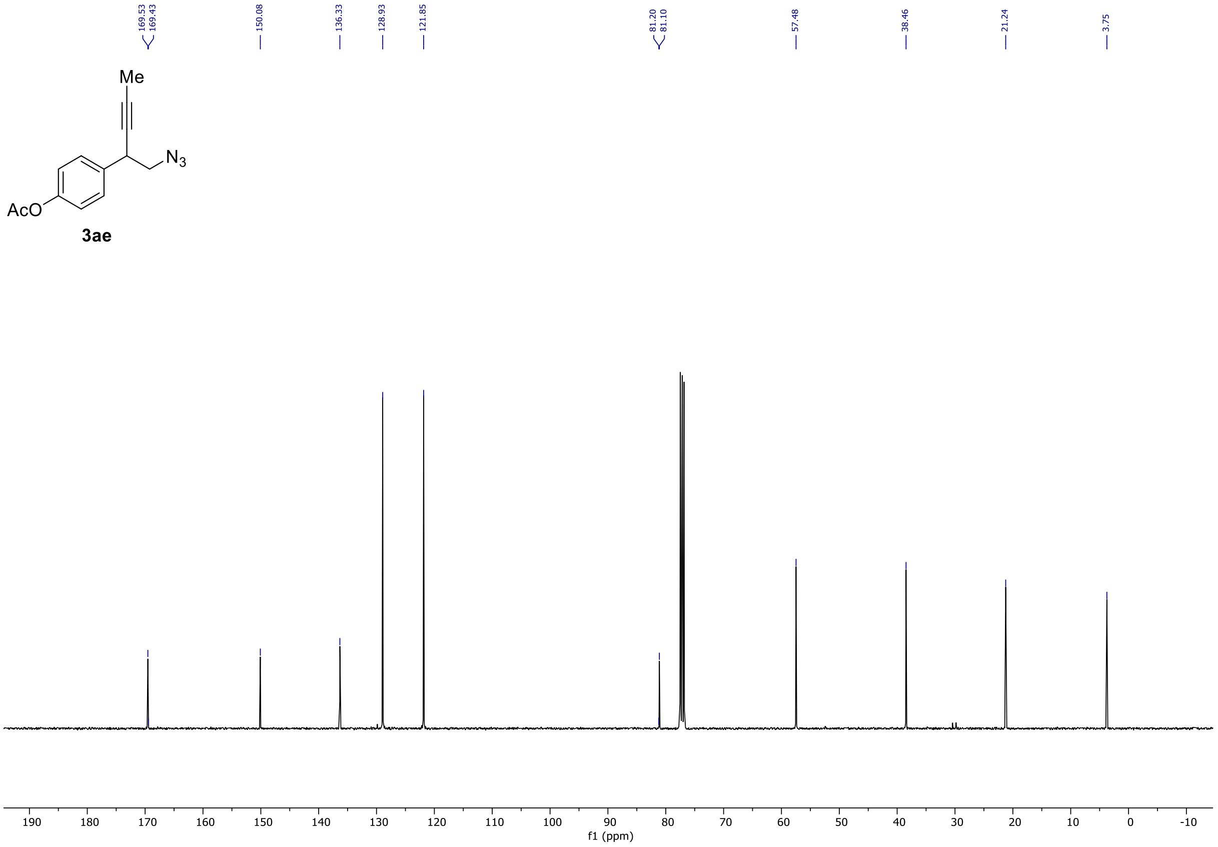

^{1}H NMR (400 MHz, $CDCl_3$) of compound **3af**:

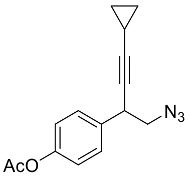

3af

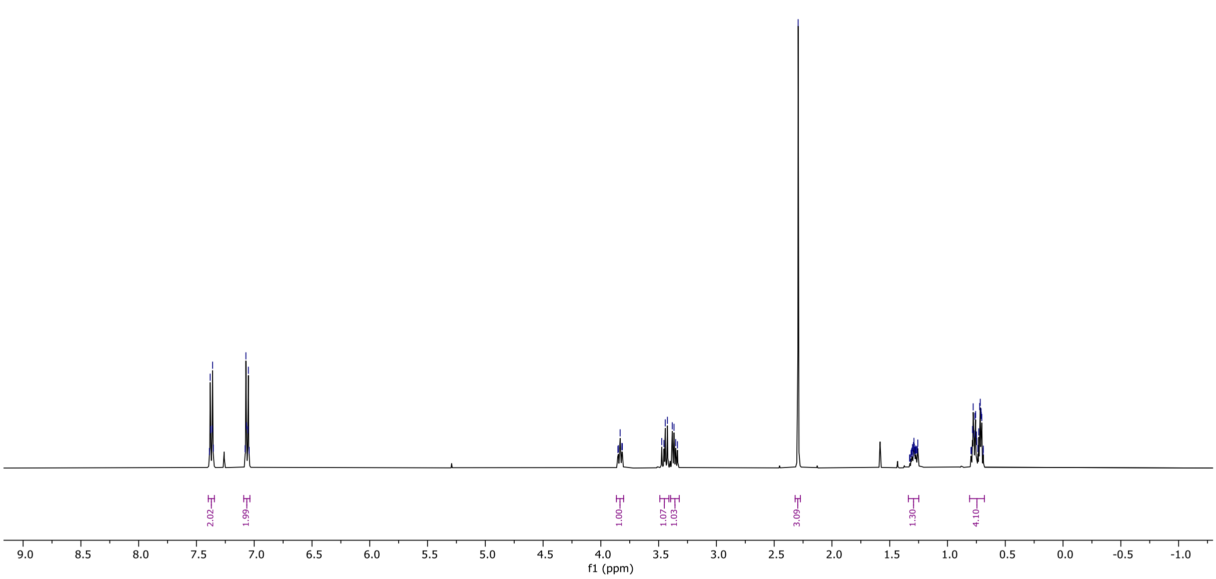

^{13}C NMR (101 MHz, $CDCl_3$) of compound **3af**:

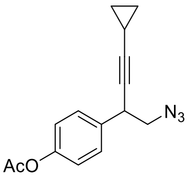

3af

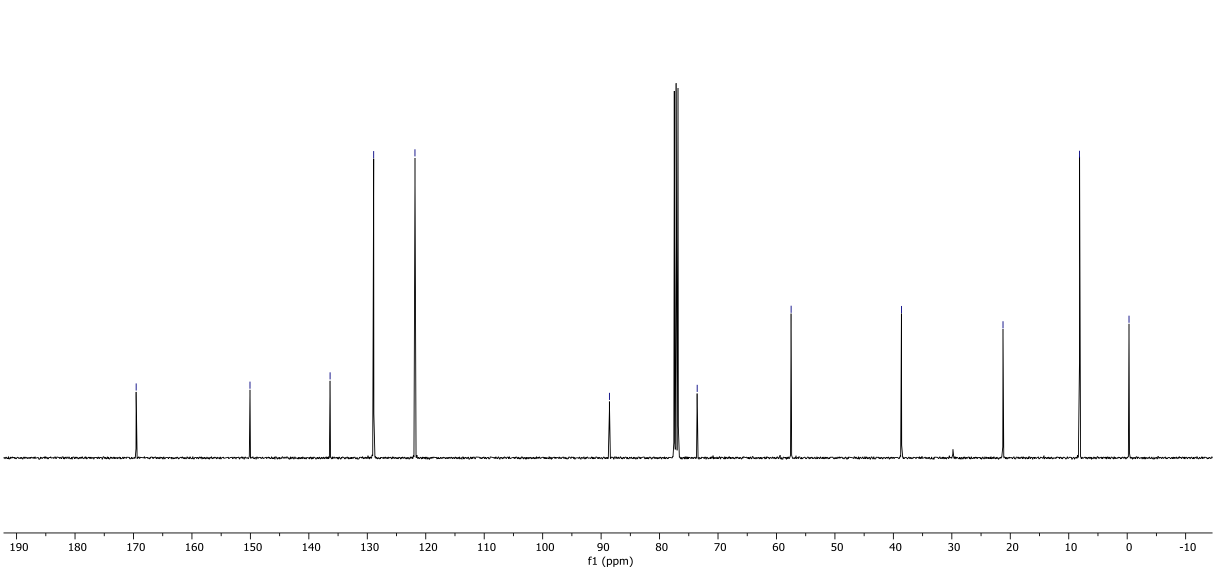

^{1}H NMR (400 MHz, $CDCl_3$) of compound **3ag**:

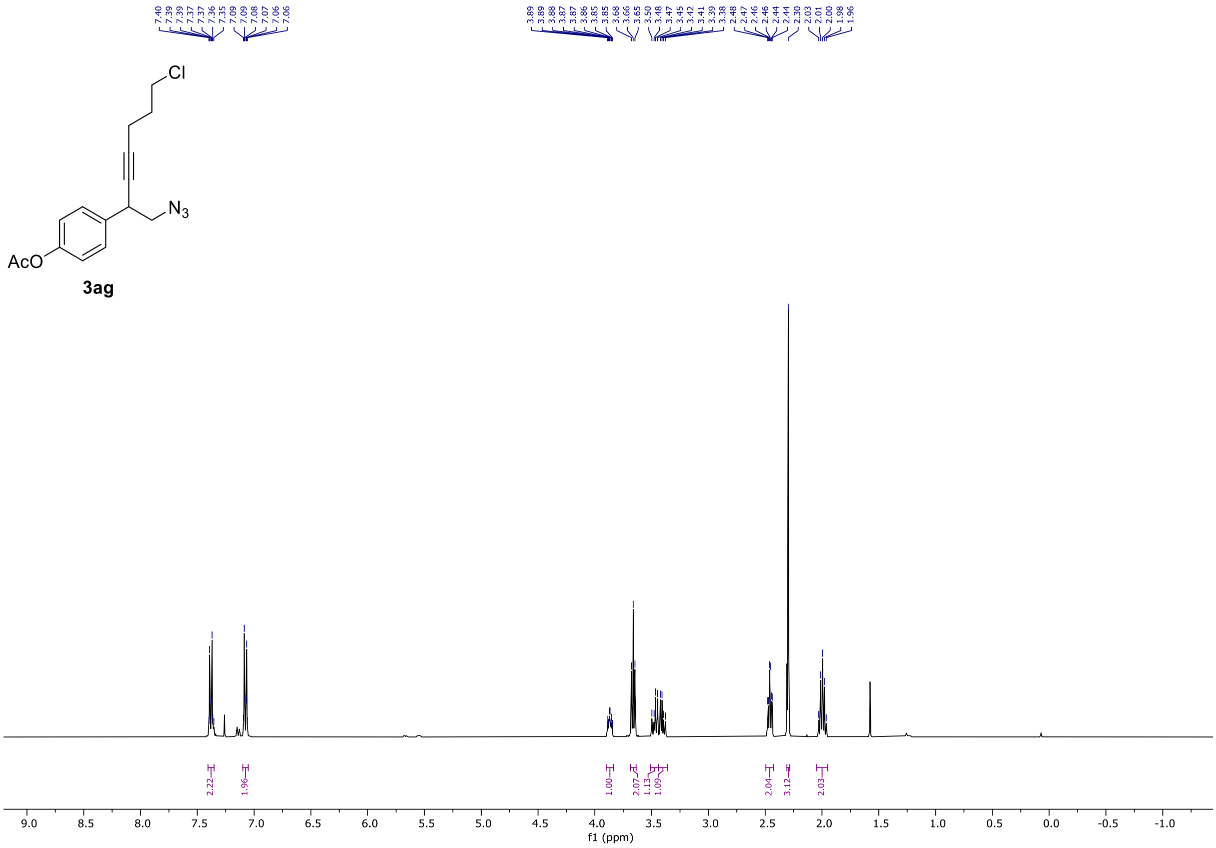

^{13}C NMR (101 MHz, $CDCl_3$) of compound **3ag**:

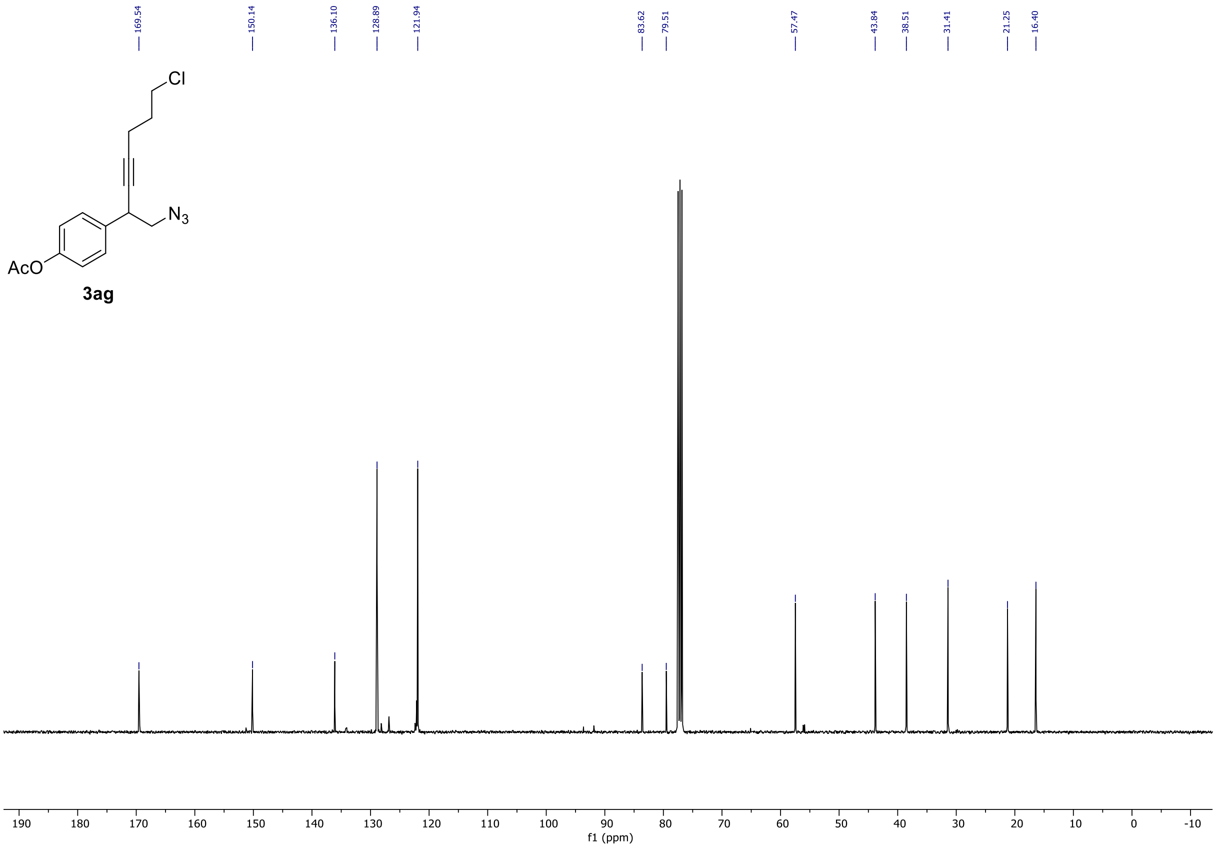

^{1}H NMR (400 MHz, $CDCl_3$) of compound **3ah**:

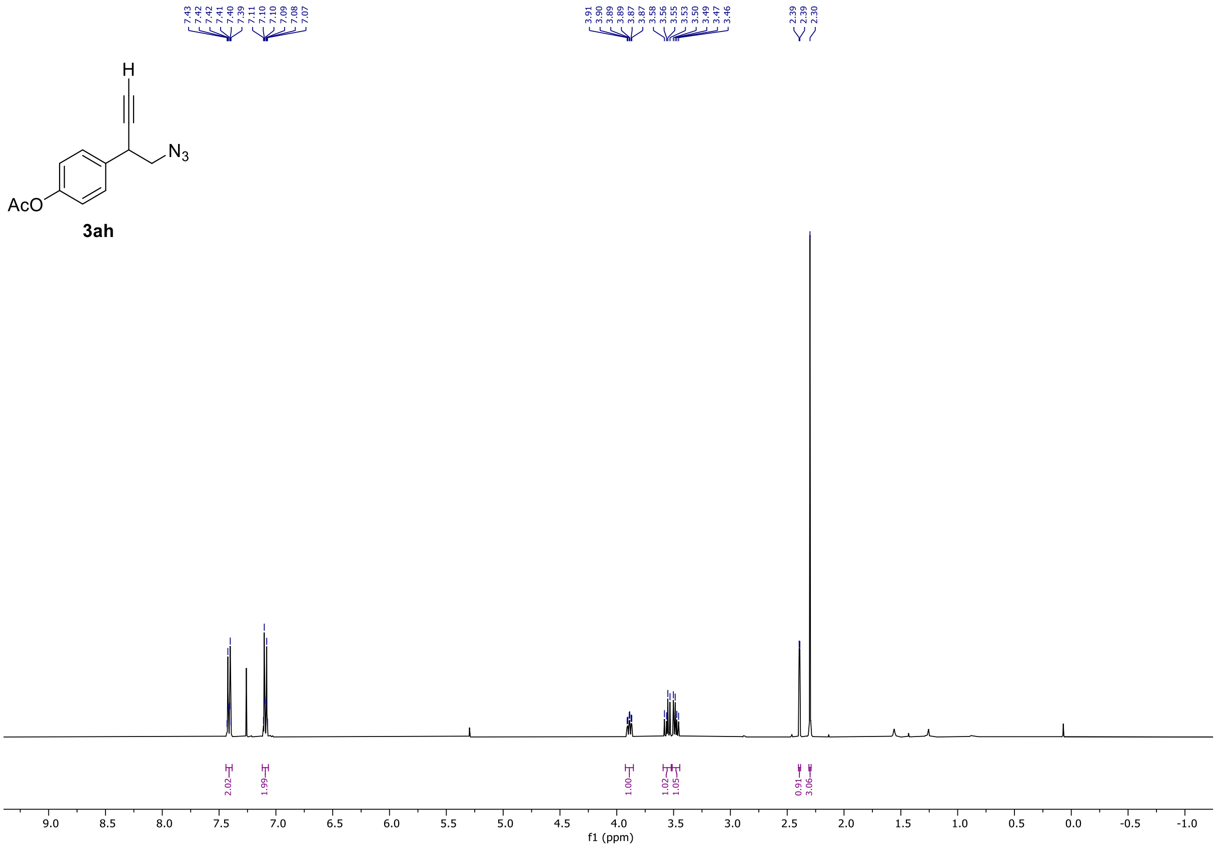

^{13}C NMR (101 MHz, $CDCl_3$) of compound **3ah**:

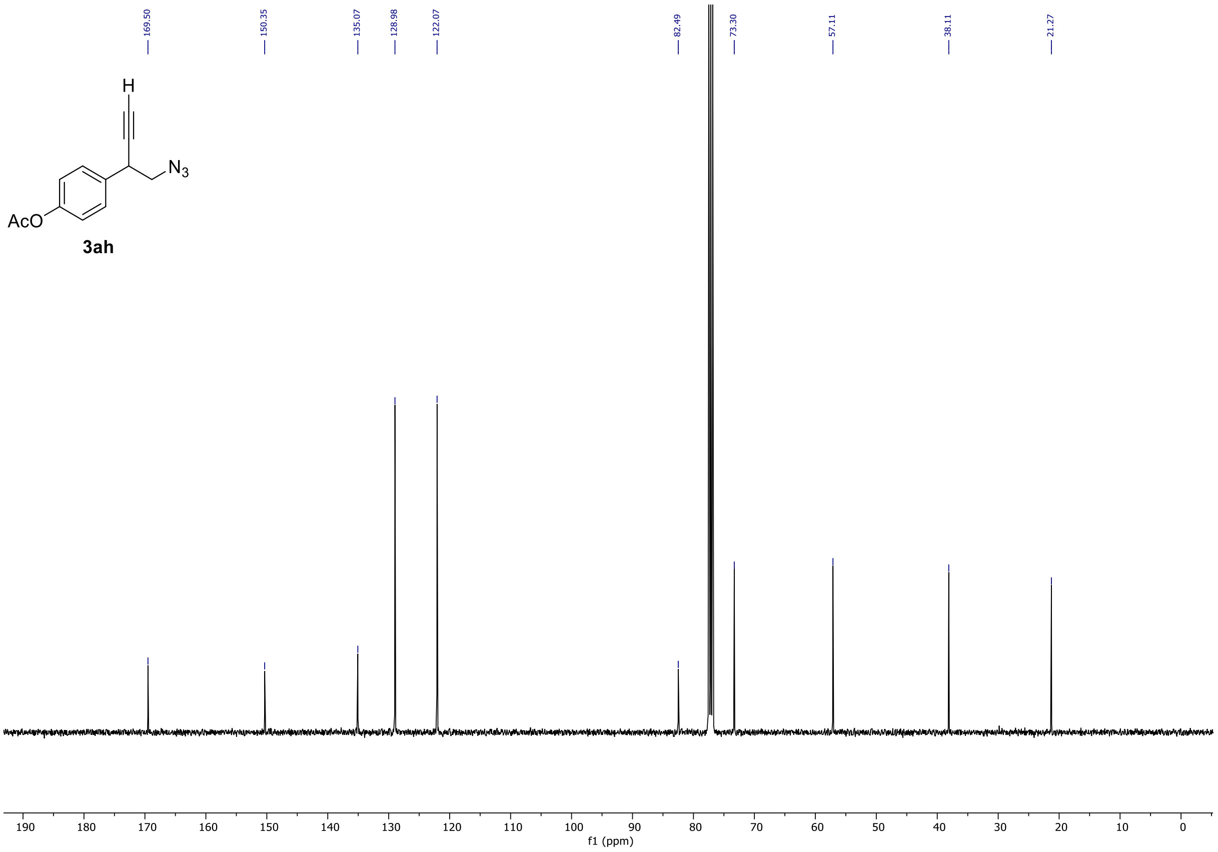

^{1}H NMR (400 MHz, $CDCl_3$) of compound **3ai**:

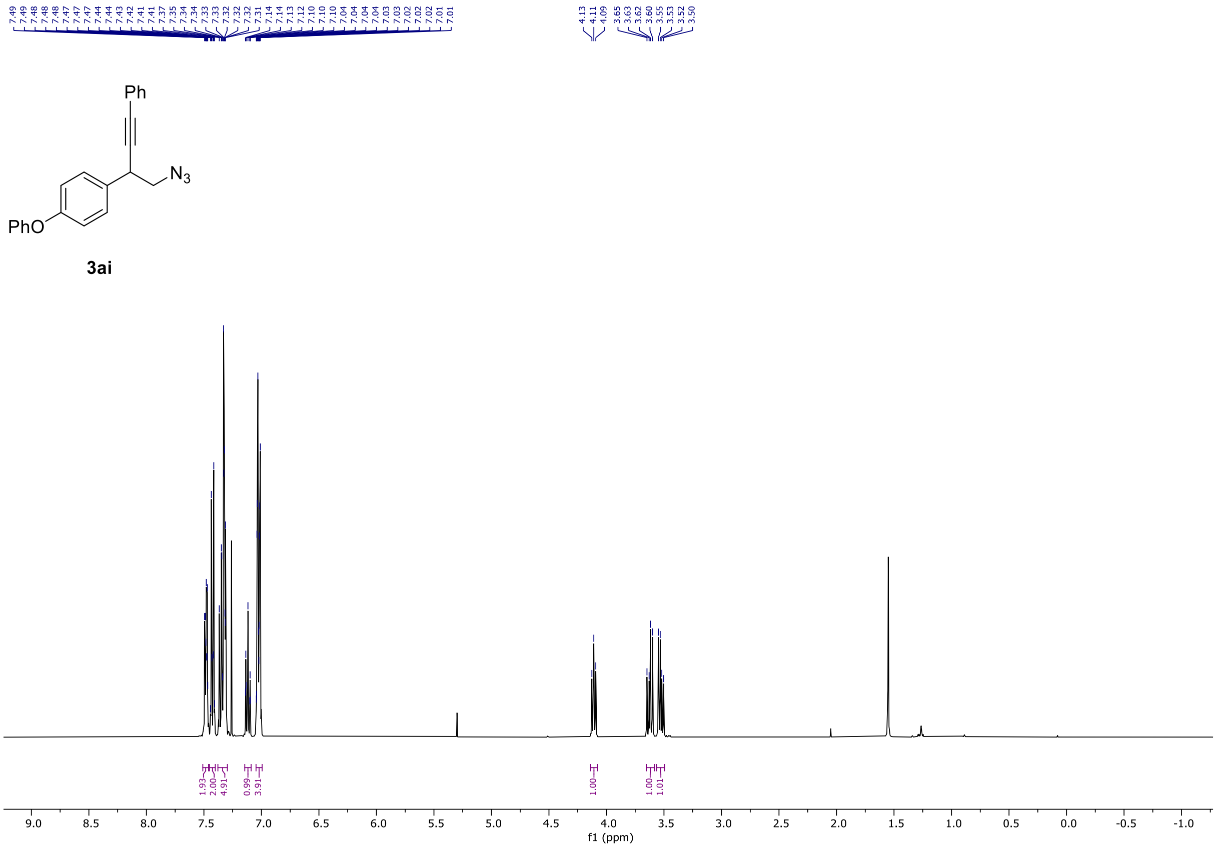

^{13}C NMR (101 MHz, $CDCl_3$) of compound **3ai**:

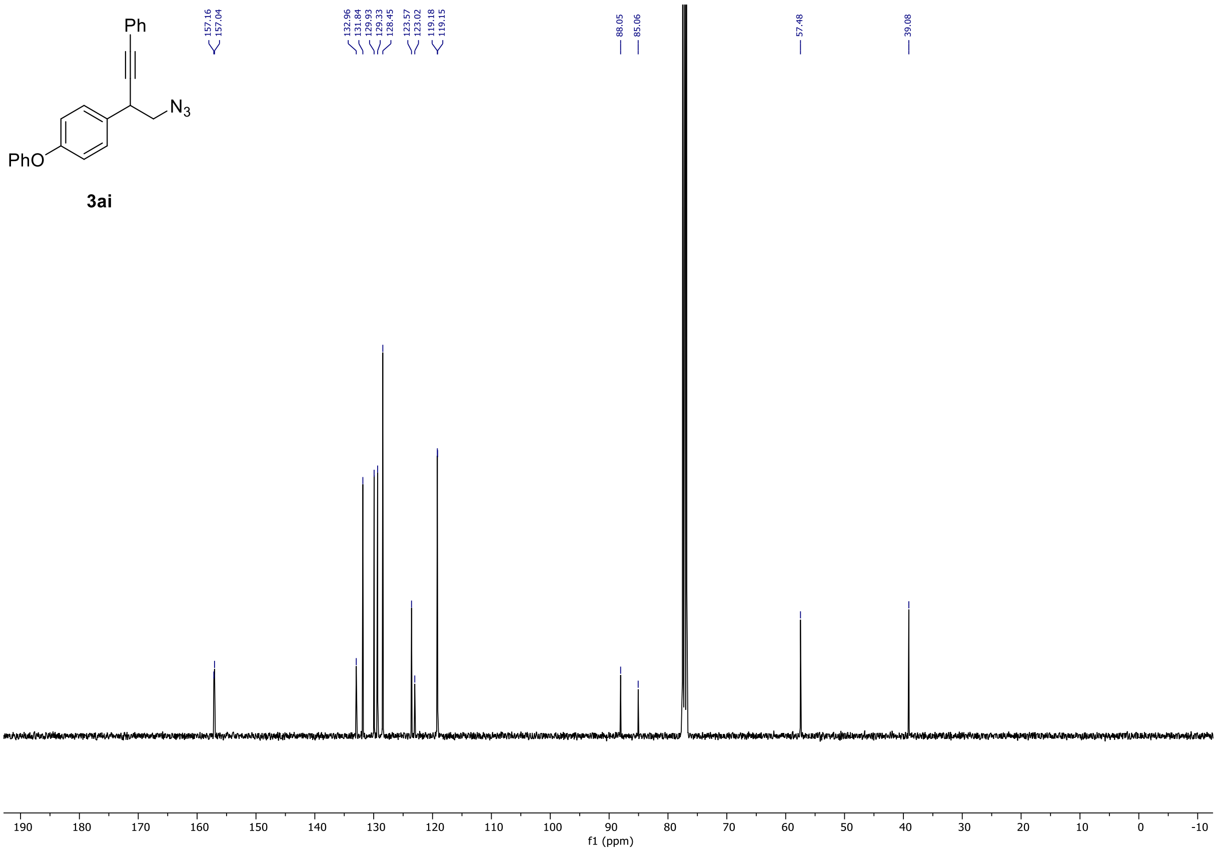

^1H NMR (400 MHz, $CDCl_3$) of compound **4**:

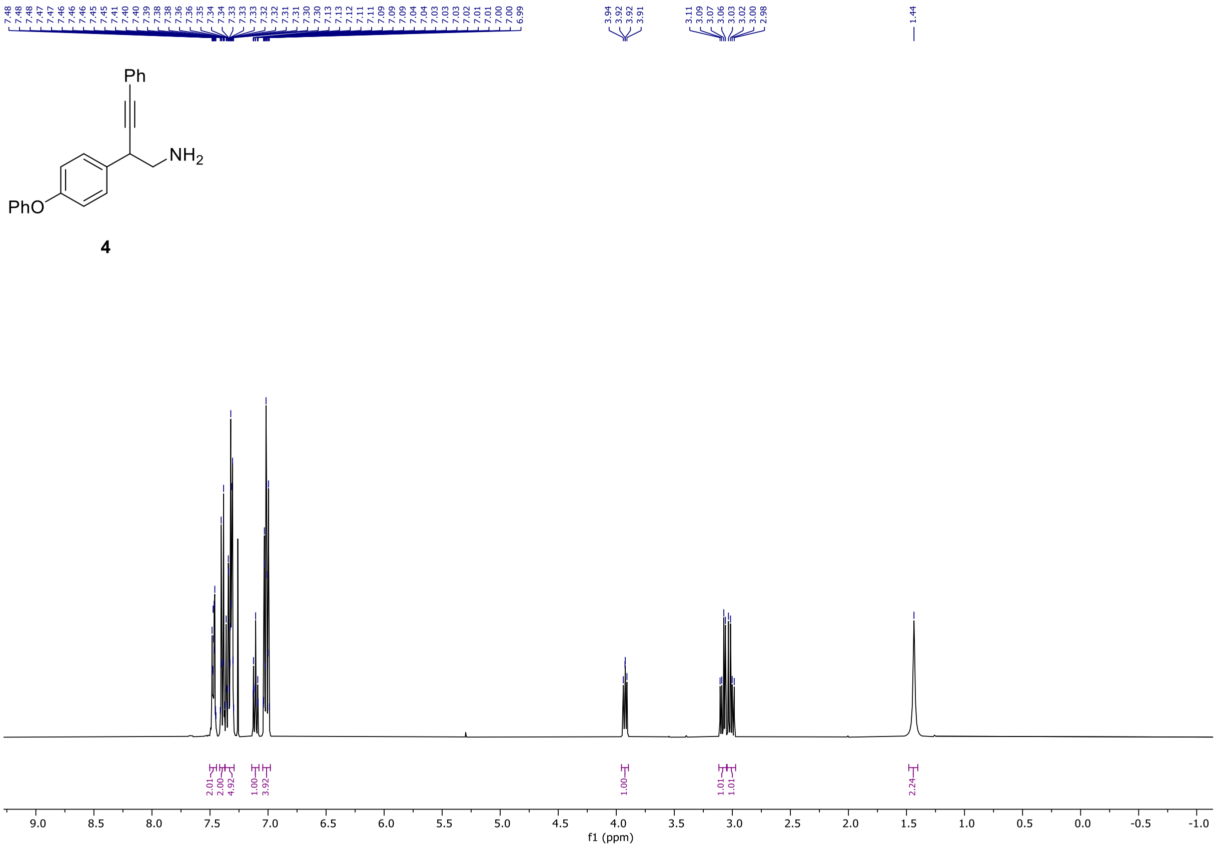

^{13}C NMR (101 MHz, $CDCl_3$) of compound **4**:

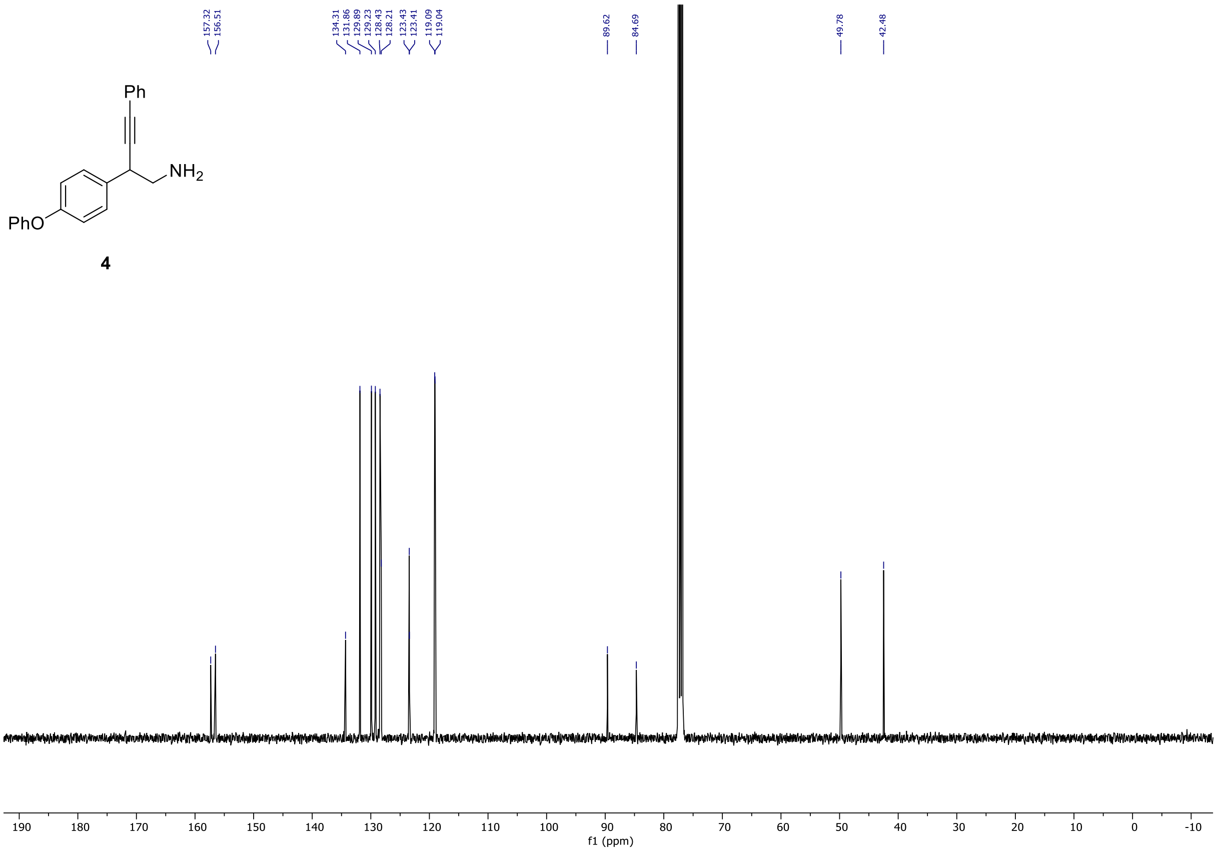

^{1}H NMR (400 MHz, $CDCl_3$) of compound **5**:

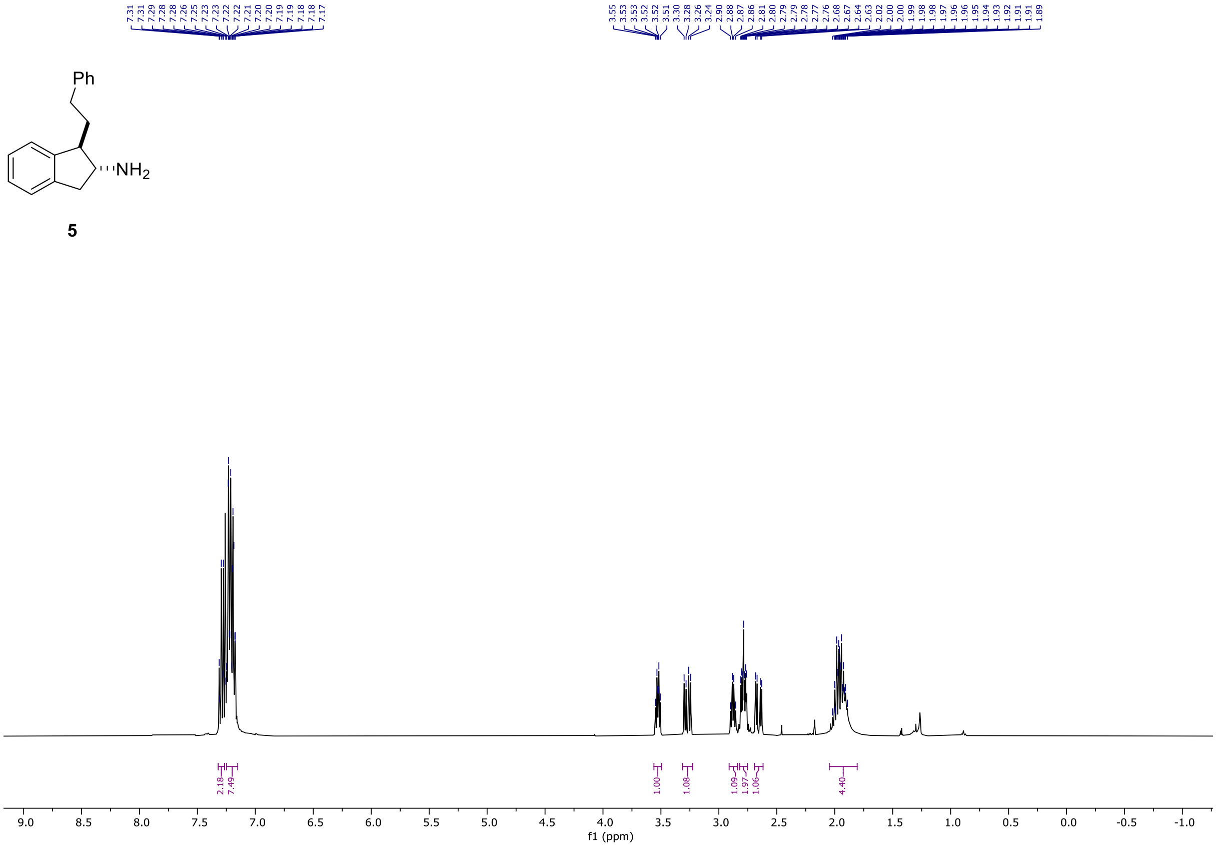

^{13}C NMR (101 MHz, $CDCl_3$) of compound **5**:

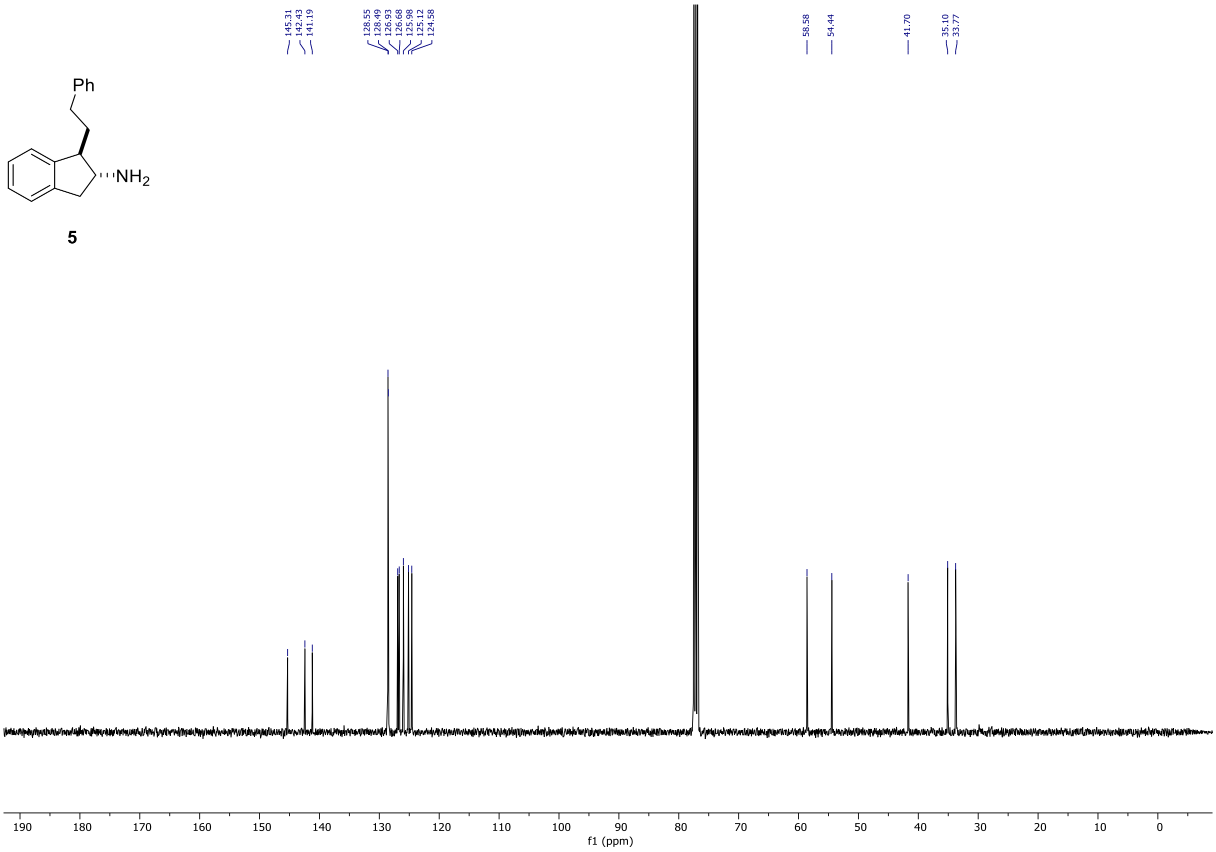

^{1}H-^{1}H NOESY NMR (400 MHz, $CDCl_3$) of compound **5**:

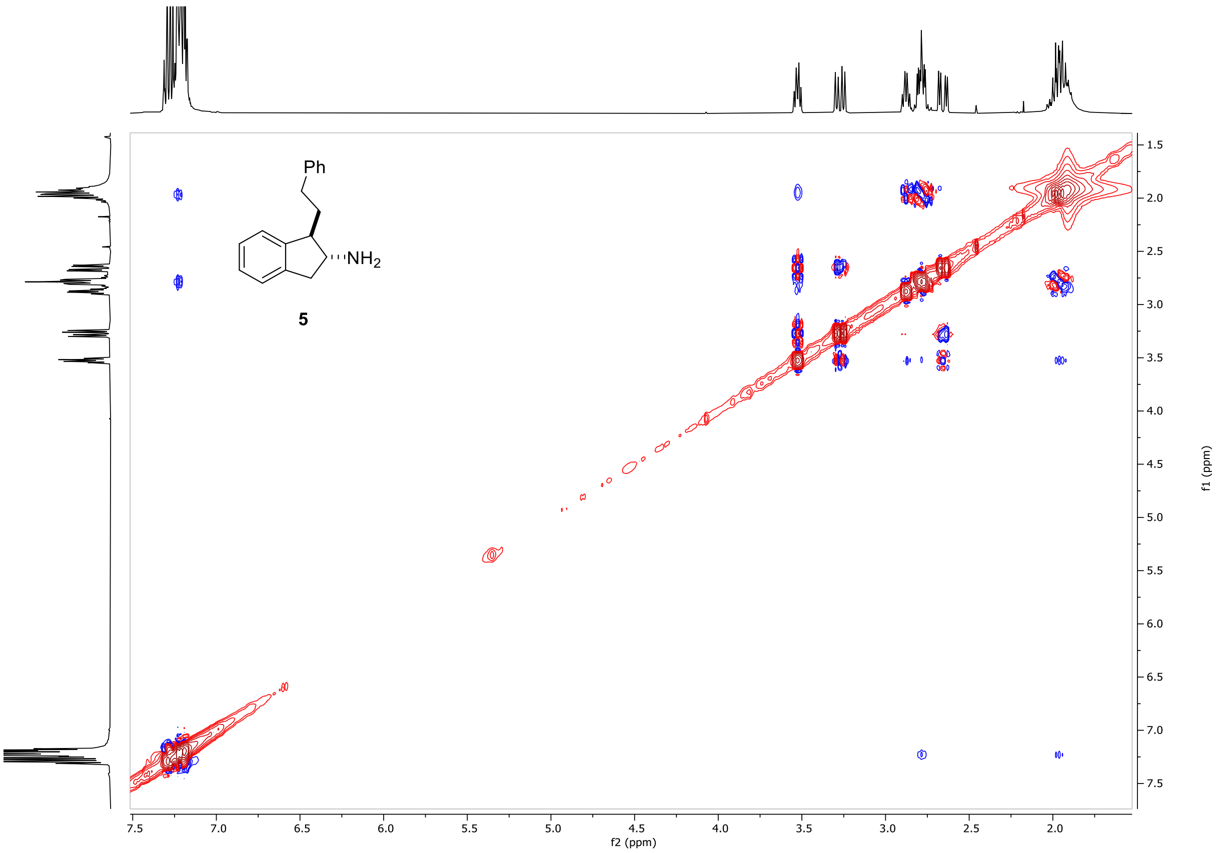

^{1}H-^{1}H NOESY NMR (400 MHz, $CDCl_3$) of compound **5** (zoom):

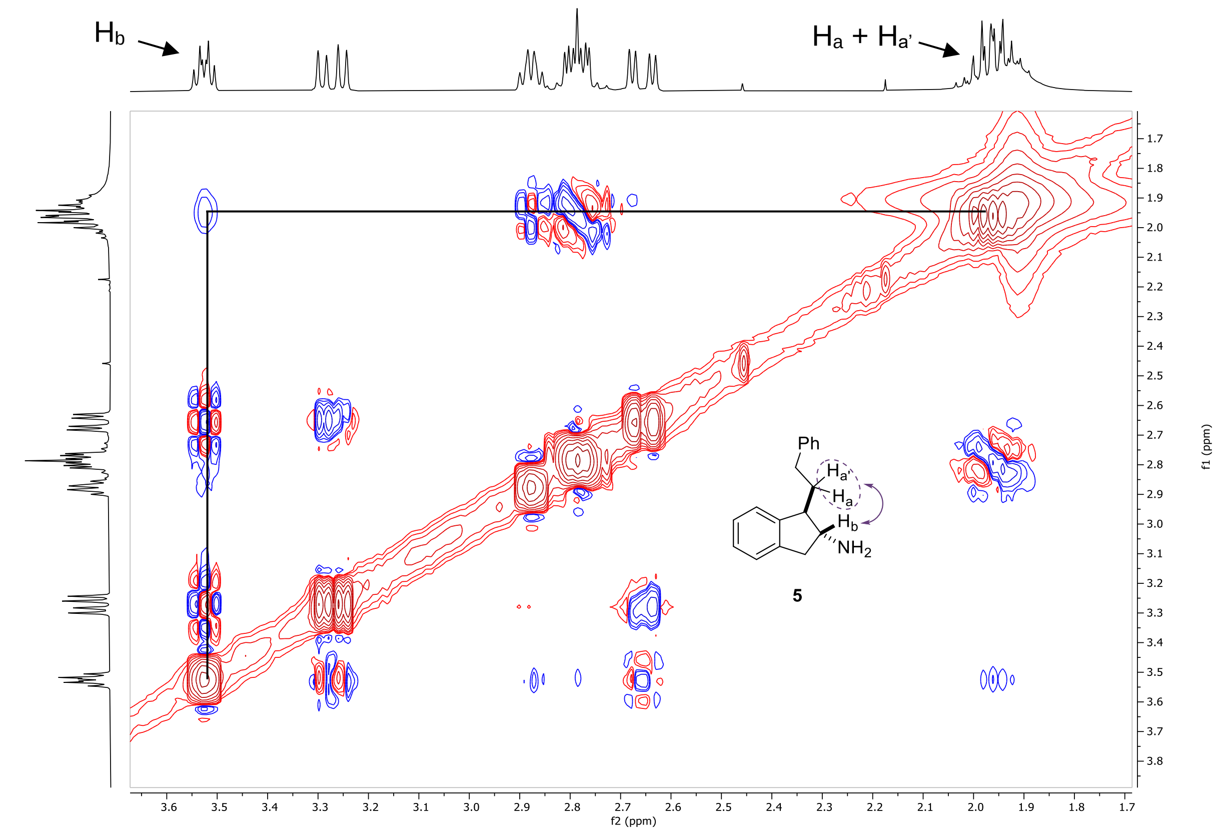

^{1}H NMR (400 MHz, $CDCl_3$) of compound **6**:

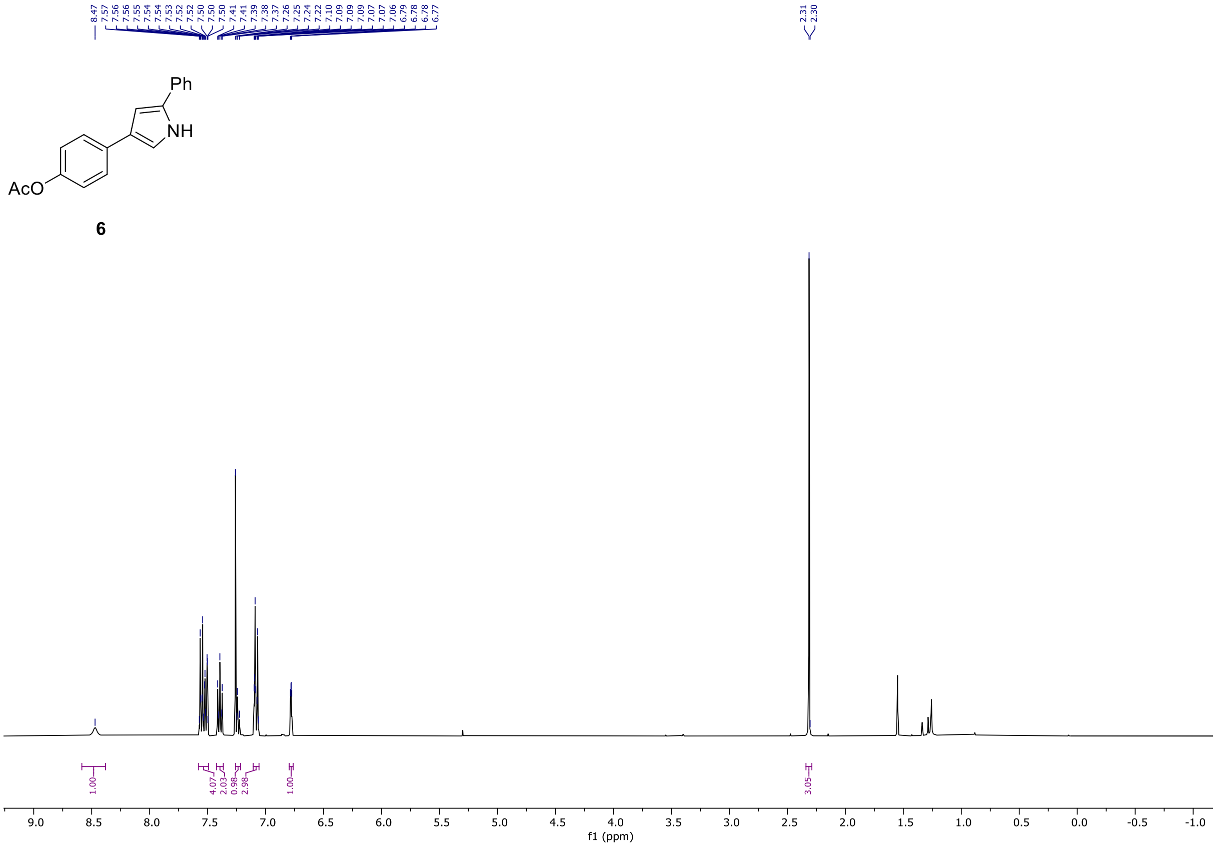

^{13}C NMR (101 MHz, $CDCl_3$) of compound **6**:

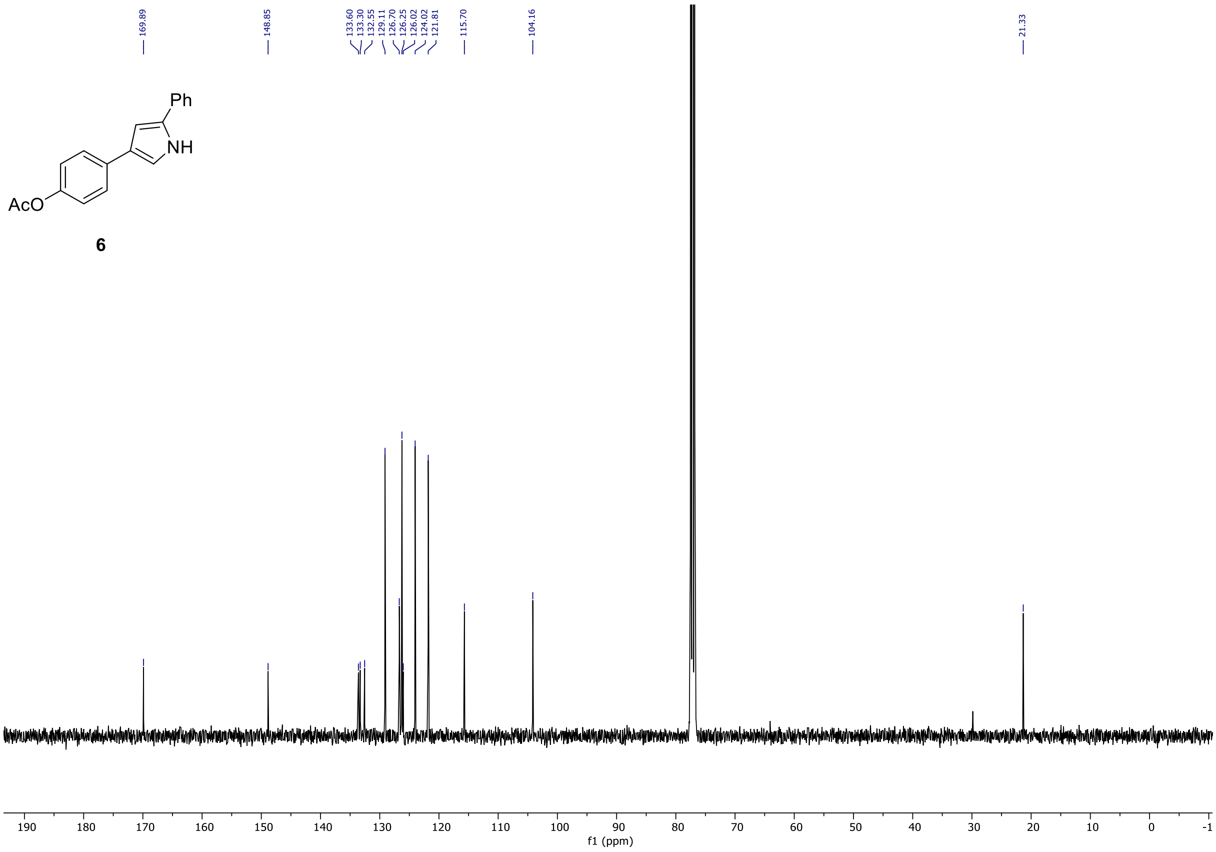

^{1}H NMR (400 MHz, $CDCl_3$) of compound **7**:

7

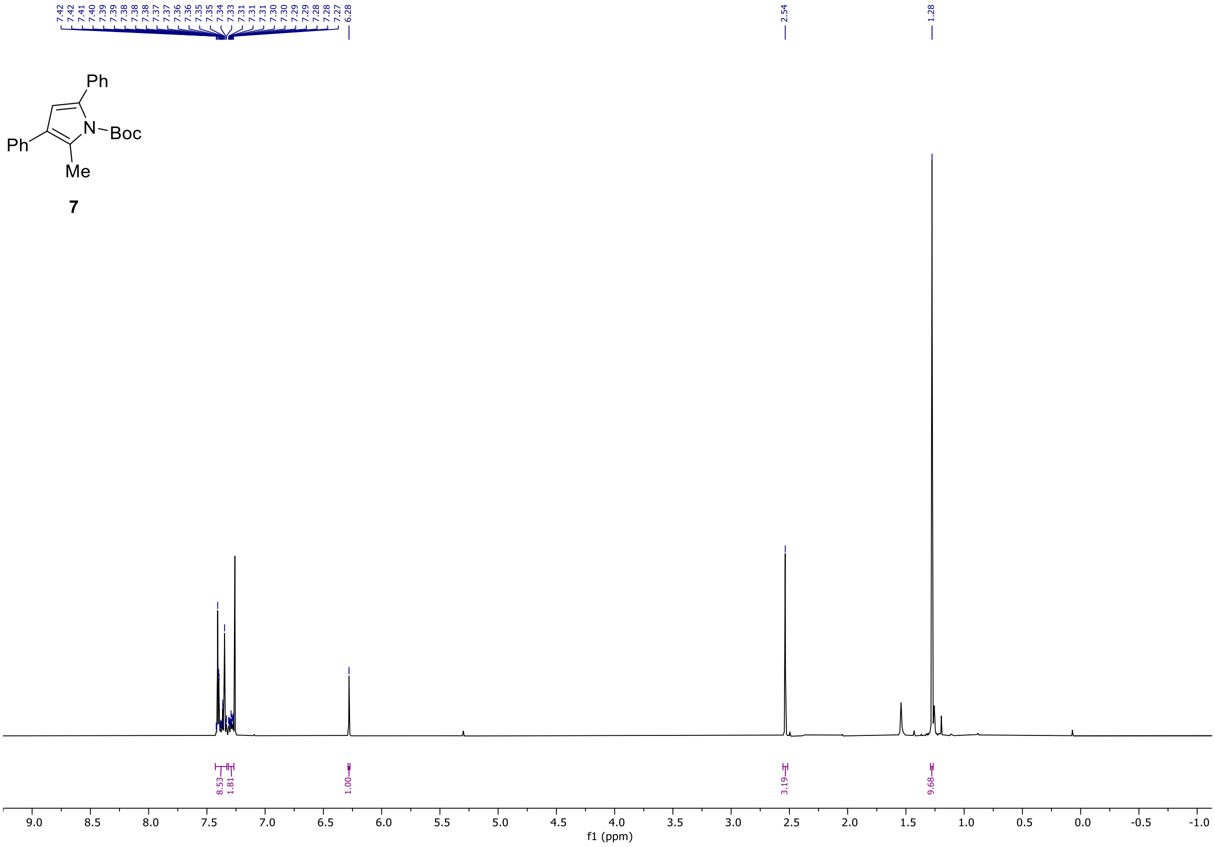

^{13}C NMR (101 MHz, $CDCl_3$) of compound **7**:

7

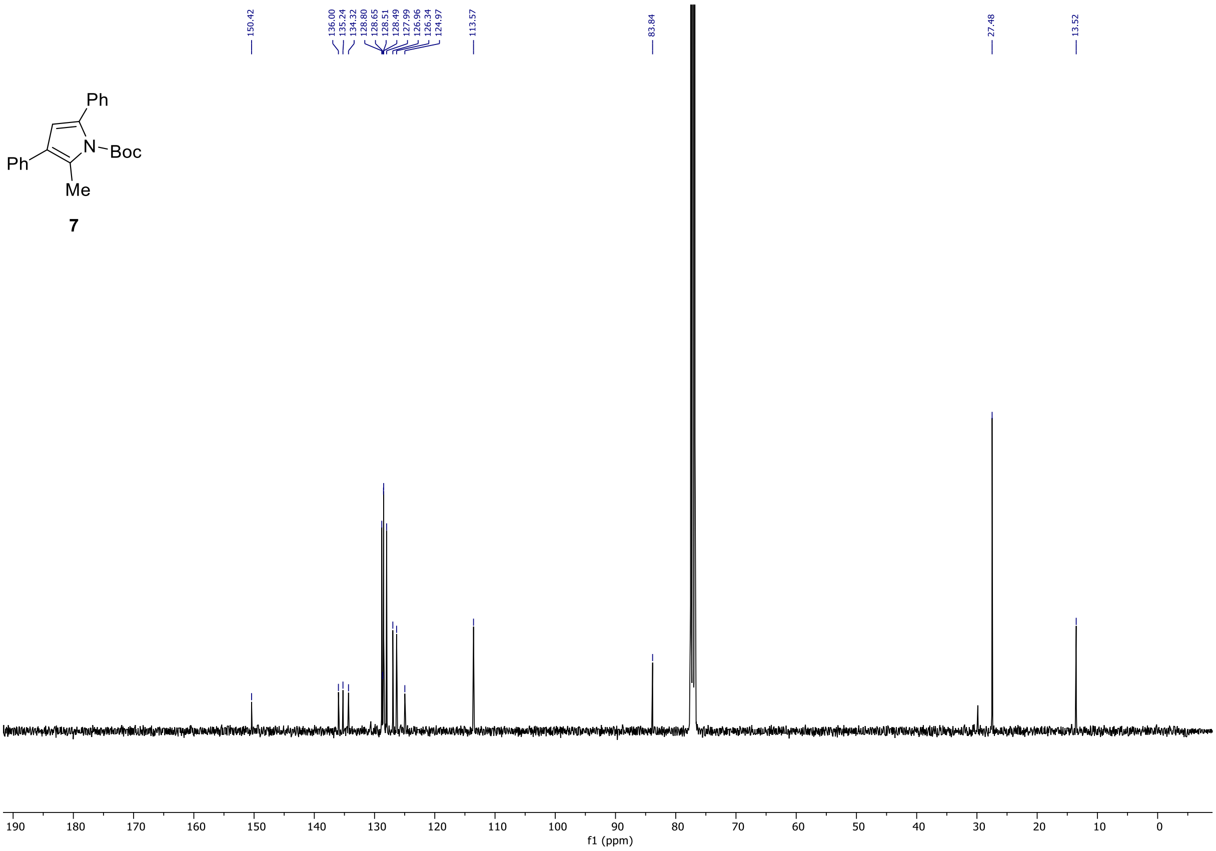

^{1}H NMR (400 MHz, $CDCl_3$) of compound **8**:

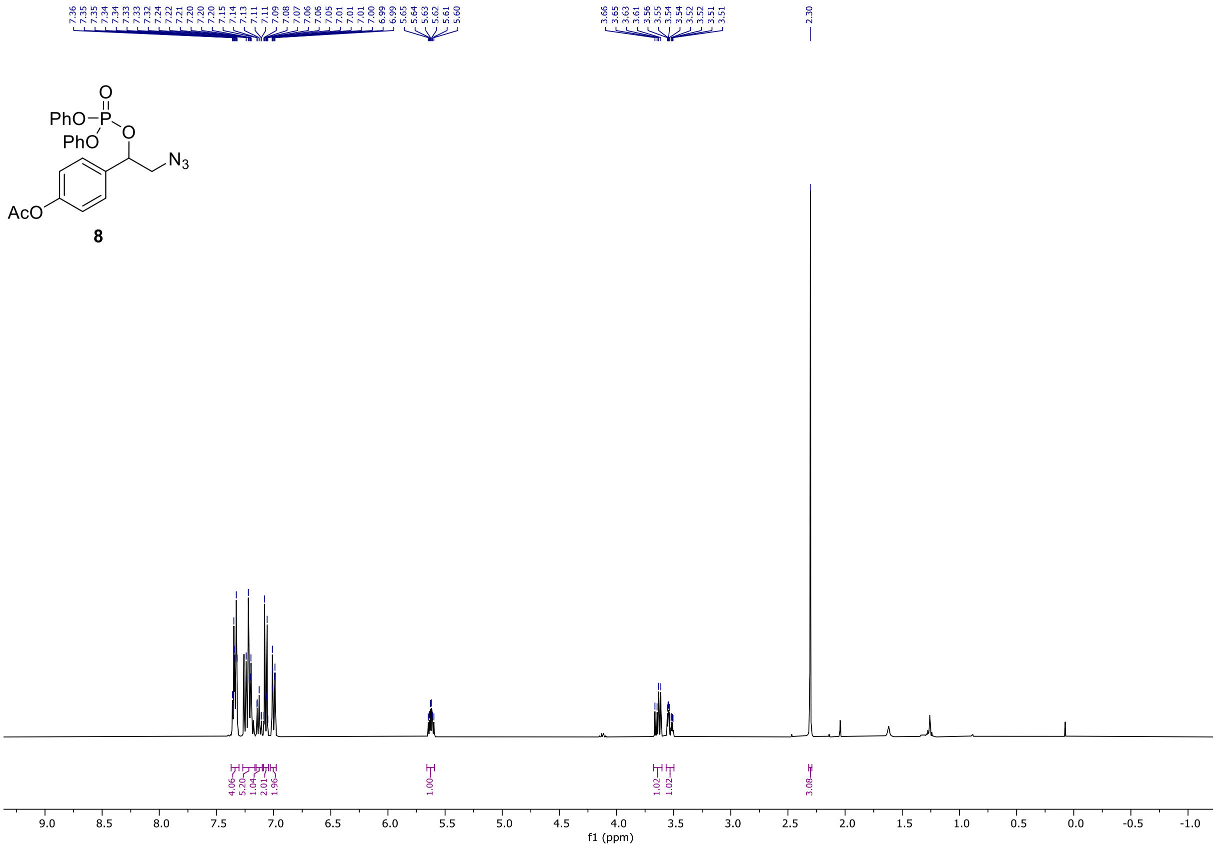

^{13}C NMR (101 MHz, $CDCl_3$) of compound **8**:

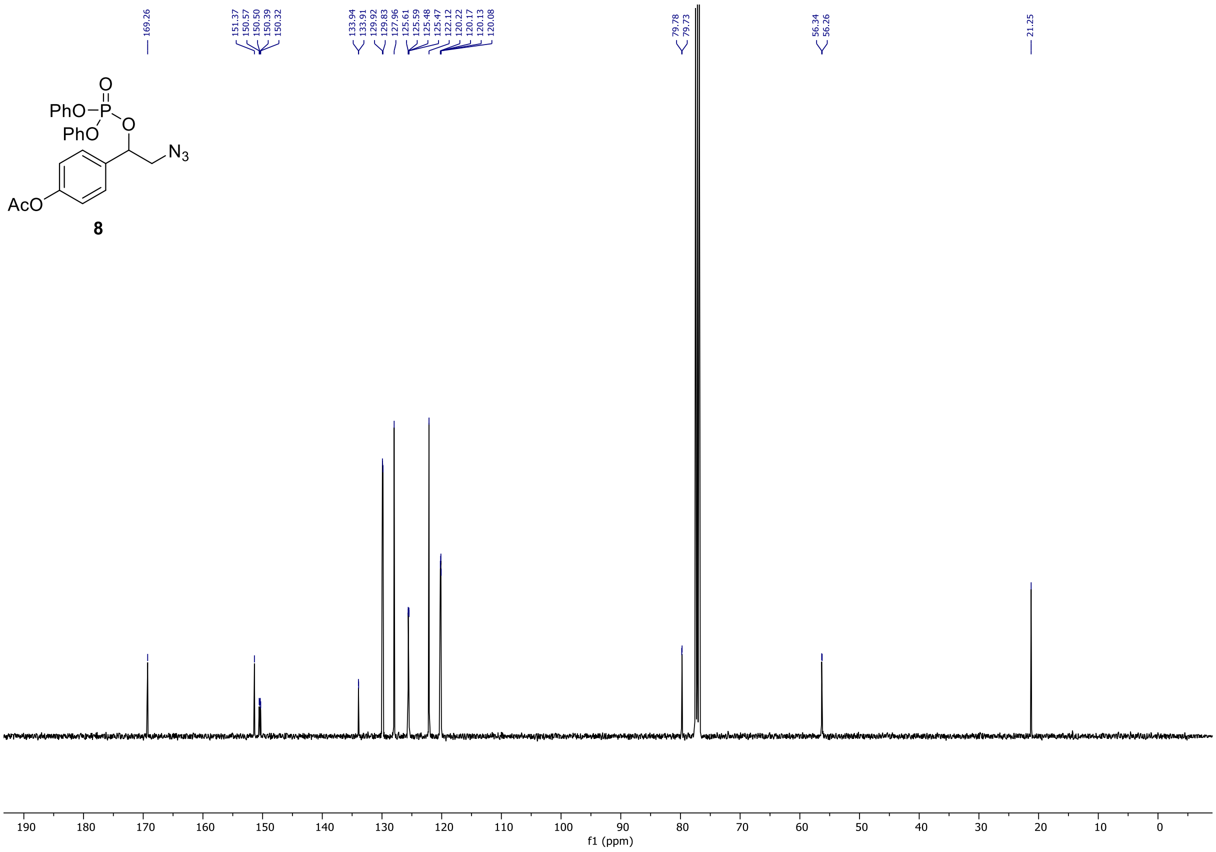

Supplement: SC-014-D3SC03309K-s001 [file SC-014-D3SC03309K-s001.pdf]
